# *In silico* investigation of binding affinities between human leukocyte antigen class I molecules and SARS-CoV-2 virus spike and ORF1ab proteins

**DOI:** 10.37349/ei.2021.00003

**Published:** 2021-04-30

**Authors:** Spyros A. Charonis, Effie-Photini Tsilibary, Apostolos P. Georgopoulos

**Affiliations:** 1 Brain Sciences Center, Department of Veterans Affairs Health Care System, Minneapolis, MN 55417, USA; 2 Department of Neuroscience, University of Minnesota Medical School, Minneapolis, MN 55455, USA

**Keywords:** ORF1ab, SARS-CoV-2, SARS-CoV-2 spike glycoprotein protein, human leukocyte antigen class I, *in silico* investigation

## Abstract

**Aim:**

The novel coronavirus severe acute respiratory syndrome coronavirus 2 (SARS-CoV-2) causes coronavirus disease 2019, a global pandemic. There is hence an urgent need for effective approaches to understand the mechanism of viral interaction with immune cells that lead to viral elimination and subsequent long-term immunity. The first, immediate response to the viral infection involves mobilization of native immunity and human leukocyte antigen (HLA) class I mechanisms to kill infected cells and eliminate the virus. The second line of defense involves the activation of HLA class II system for the production of antibodies against the virus which will add to the elimination of the virus and prevent future infections. In a previous study, investigated the relations between SARS-CoV-2 spike glycoprotein (S protein) and HLA class II alleles were investigaed; here report on the relations of the S protein and the open reading frame 1ab (ORF1ab) of SARS-CoV-2 to HLA class I alleles.

**Methods:**

An *in silico* sliding window approach was used to determine exhaustively the binding affinities of linear epitopes of 10 amino acid length (10-mers) to each of 61 common (global frequency ≥ 0.01) HLA class I molecules (17, 24 and 20 from gene loci *A*, *B* and *C*, respectively). A total of 8,354 epitopes were analyzed; 1,263 from the S protein and 7,091 from ORF1ab.

**Results:**

HLA-*A* genes were the most effective at binding SARS-CoV-2 epitopes for both spike and ORF1ab proteins. Good binding affinities were found for all three genes and were distributed throughout the length of the S protein and ORF1ab polyprotein sequence.

**Conclusions:**

Common HLA class I molecules, as a population, are very well suited to binding with high affinity to SARS-CoV-2 spike and ORF1ab proteins and hence should be effective in aiding the early elimination of the virus.

## Introduction

Severe acute respiratory syndrome coronavirus 2 (SARS-CoV-2) causes coronavirus disease 2019 (COVID-19), a disease that has now become a global pandemic. The steps following infection include a first phase during which native immunity mechanisms and human leukocyte antigen (HLA) class I molecules (of the *A*, *B*, *C* genes) combat the virus by recruiting cells entered by the virus which is then fragmented via proteasomal cleavage to 9–13 amino acid (AA) fragments. HLA class I genes code for cell-surface glycoproteins are expressed on nucleated cells and present on the cell surface those antigen peptides to CD8^+^ cytotoxic T cells to signal cell destruction, thus eliminating infected cells. Thus, HLA class I restricted processing and presentation alerts the immune system to any infectious processes unfolding intracellularly and provides potential targets for a cytotoxic T cell response. During the second phase of response to the viral infection, HLA class II molecules are involved to initiate the production of specific antibodies against the virus. HLA class II molecules (of the HLA-*DR*, *-DQ* and *-DP* genes) are expressed on professional antigen-presenting cells (e.g., macrophages, dendritic cells) and present endocytosed extracellular antigen peptides to CD4^+^ T cells to promote B-cell mediated antibody production and immune memory. The HLA region in chromosome 6 is the most highly polymorphic in the human genome resulting in considerable individual and population variability in HLA composition, reflecting the long evolutionary history of exposure to and dealing with elimination and ultimate protection from various pathogens [1, 2].

In a previous study [3], we reported on the relations between SARS-CoV-2 virus spike glycoprotein (S protein) and 66 common HLA class II alleles, investigated using an *in silico* approach [4] by assessing the binding affinity of epitopes of the S protein to these most commonly occurring alleles (frequency ≥ 0.01). In the current study, we employed the same sliding epitope window methodology to exhaustively scan the entire S protein and open reading frame 1ab (ORF1ab) protein and determine the binding affinity of each *n*-mers (*n* = 10) AA epitopes to 61 common HLA class I alleles.

## Materials and methods

The main objective of this study was to exhaustively assess the binding affinities of HLA class I molecules to the SARS-CoV-2 S protein and the ORF1ab polyprotein. For that purpose, we assessed the binding affinities of 61 common class I alleles as described below.

### HLA alleles

For this study, we selected the more frequent alleles of classical HLA class I genes (*A*, *B*, *C*), namely all alleles with global frequencies ≥ 0.01 (*n* = 61 total), an arbitrary but reasonable threshold. For that purpose, we obtained an Estimation of Global Allele Frequencies by querying the website http://www.allelefrequencies.net/ [5]. The alleles with frequencies ≥ 0.01 that we used are given in Table 1. They comprised 17, 24 and 20 alleles of *A*, *B* and *C* genes, respectively.

### SARS-CoV-2 S protein

**Figure 1.**
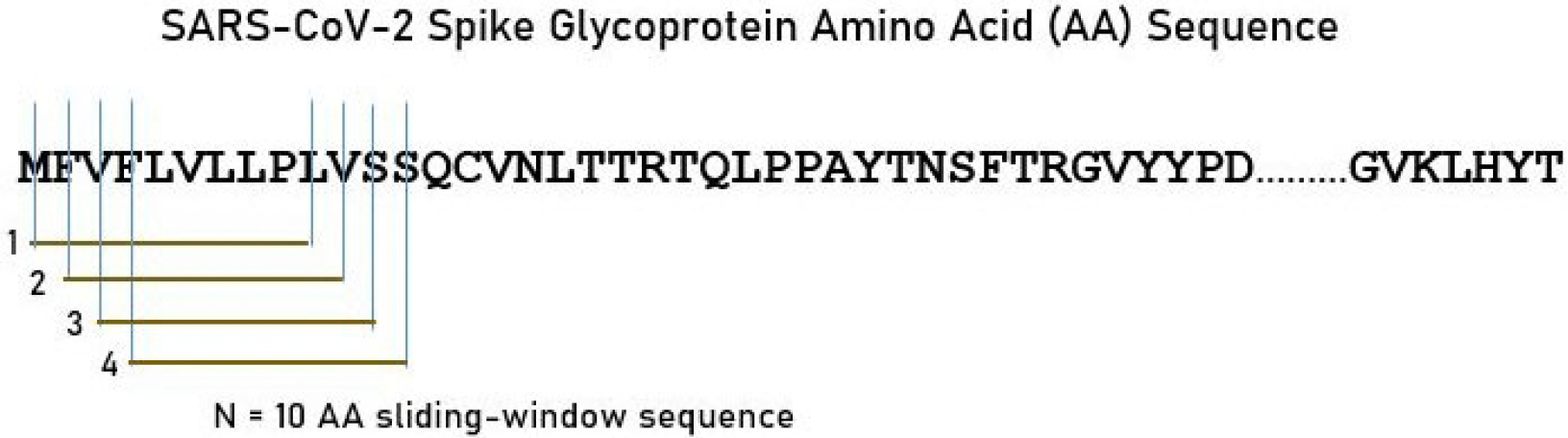
A sample of the sliding window approach for the SARS-CoV-2 S protein (see text for details)

The AA sequence of the SARS-CoV-2 S protein (“glycoprotein”) was retrieved from the UniprotKB database [6]. It consists of 1,273 AA residues. As mentioned above, the main objective of this study was to exhaustively assess the binding affinities of HLA class I and II molecules to the SARS-CoV-2 S protein. For that purpose, we used a sliding epitope window approach [3, 4] to partition the sequence of the S protein into subsequences of all possible consecutive 10-mers for (e.g., residues S1-S10, S2-S11, …, S1263-S1273) that covered the entire sequence length (1,273 AA). The method is illustrated in Figure 1.

### ORF1ab

The complete AA sequence of the ORF1ab polyprotein was retrieved on February 2, 2021 from the NCBI SARS-CoV-2 data hub resource (URL: www.ncbi.nlm.nih.gov/sars-cov-2). The retrieved sequence (GenBank Acc. number: QQX03240) was matched by filtering the search for release date (most recent sequence) and protein (ORF1ab polyprotein). The ORF1ab encodes numerous viral proteins including a leader protein, non-structural protein (nsp)2–9, 3C-like proteinase, RNA-dependent RNA polymerase, helicase, exonuclease, endoRNAse and methyltransferase. The ORF1ab polyprotein consists of 7,101 AA residues. The sliding epitope window approach illustrated in Figure 1 was applied to analyze the binding affinity of all possible linear epitopes.

Specifically, a set of 10-AA-length subsequences (considered in this analysis as putative epitopes) was generated (number of subsequences = length of the protein sequence - 10) and FASTA-formatted for input. The number of subsequences was hence 1,273 – 10 = 1,263 for the S protein and 7,101 – 10 = 7,091 for the ORF1ab polyprotein (“polyprotein”). The FASTA-formatted subsequences were then queried in the Immune Epitope Database (IEDB) (www.iedb.org) [7] in order to determine their binding affinity to a specific HLA class I molecule. Binding affinity predictions were obtained using the NetMHCpan eluted ligands (EL) method [8]. For each 10-mer, a binding affinity score was predicted and reported as a percentile rank by comparing the peptide’s score against the scores of 5 million random 10-mers (not limited to any one species or other taxonomic rank) selected from the SwissProt database [7]. For each gene and protein, all alleles and epitopes were entered as a single query and, thus, the same set of 5 million random 10-mers was employed to rank all queried alleles. Smaller percentile ranks indicate higher binding affinity. Next, the lowest (minimum) percentile rank (LPR) for each allele and 10-mer of the S protein and ORF1ab polyprotein was retrieved and retained (N = 1,263 epitopes × 61 alleles = 77,043 values for glycoprotein; N = 7,091 epitopes × 61 alleles = 432,551 for ORF1ab polyprotein). Finally, for various analyses (see below) we employed a conservative threshold of LPR = 1 and performed analyses on the percentage of cases with LPR < 1 (“good” binding affinities).

### Data analysis

Initially, we analyzed the data to assess the effect of protein, gene, and protein × gene interaction on the percentage of cases with LPR < 1, where this percentage was calculated for each allele across all epitopes of each protein. Subsequently, for each epitope, we calculated the percentage of alleles for which LPR < 1 in order to evaluate the distribution of allele affinity across the sequence of the two proteins. Standard statistical methods were employed in these analyses, including analysis of variance (ANOVA) and linear regression, using the IBM-SPSS statistical package (version 27).

## Results

### Overall estimated affinities

For the S protein, there were 981/77,043 (1.273%) cases with LPR < 1, and for the ORF1ab polyprotein 5,767/432,551 (1.333%). These proportions did not differ significantly (Wald H0 test of two proportions, two-sided *P* = 0.180). All values for which LPR < 1 for each allele (with associated epitope sequences) are given in Supplemental Table A for the S protein and Supplemental Table B for the ORF1ab polyprotein.

### Affinities of individual alleles

The percentage of LPR < 1 (across all epitopes of a protein) for each allele and protein, and their average are shown in Table 2.

### Effect of protein and gene

**Figure 2.**
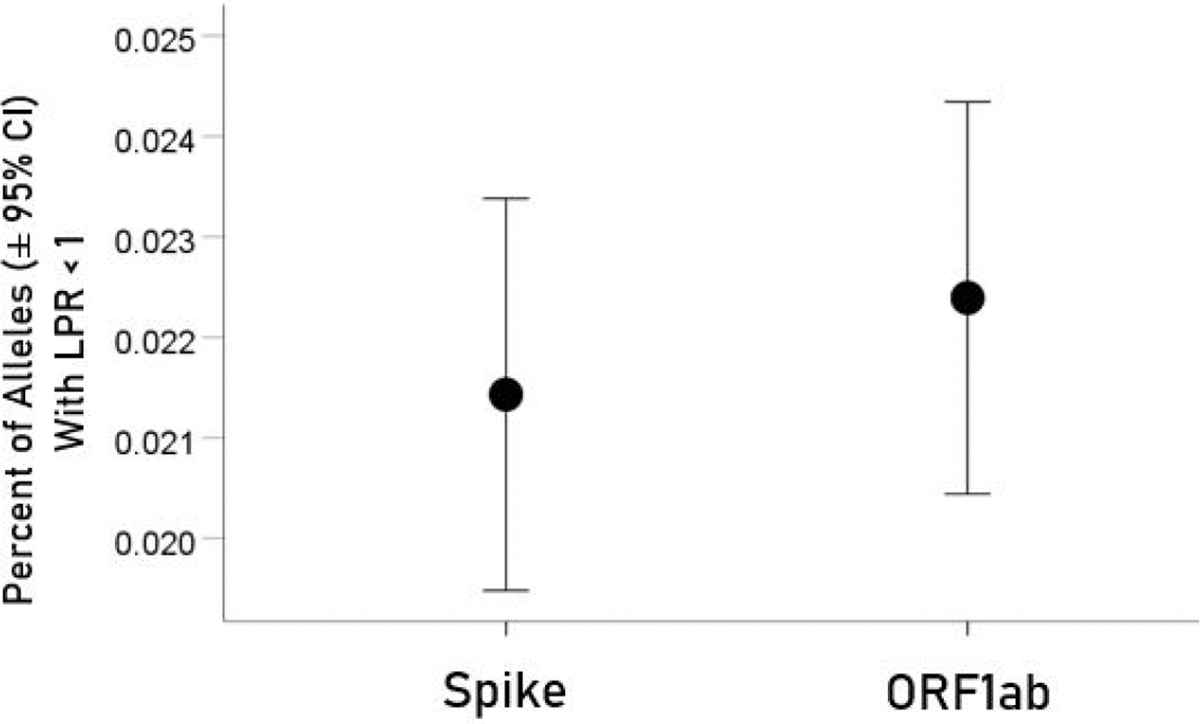
Mean percentages [± 95% confidence interval (CI)] of alleles with LPR < 1 for the two proteins. See text for details of statistical comparisons

**Figure 3.**
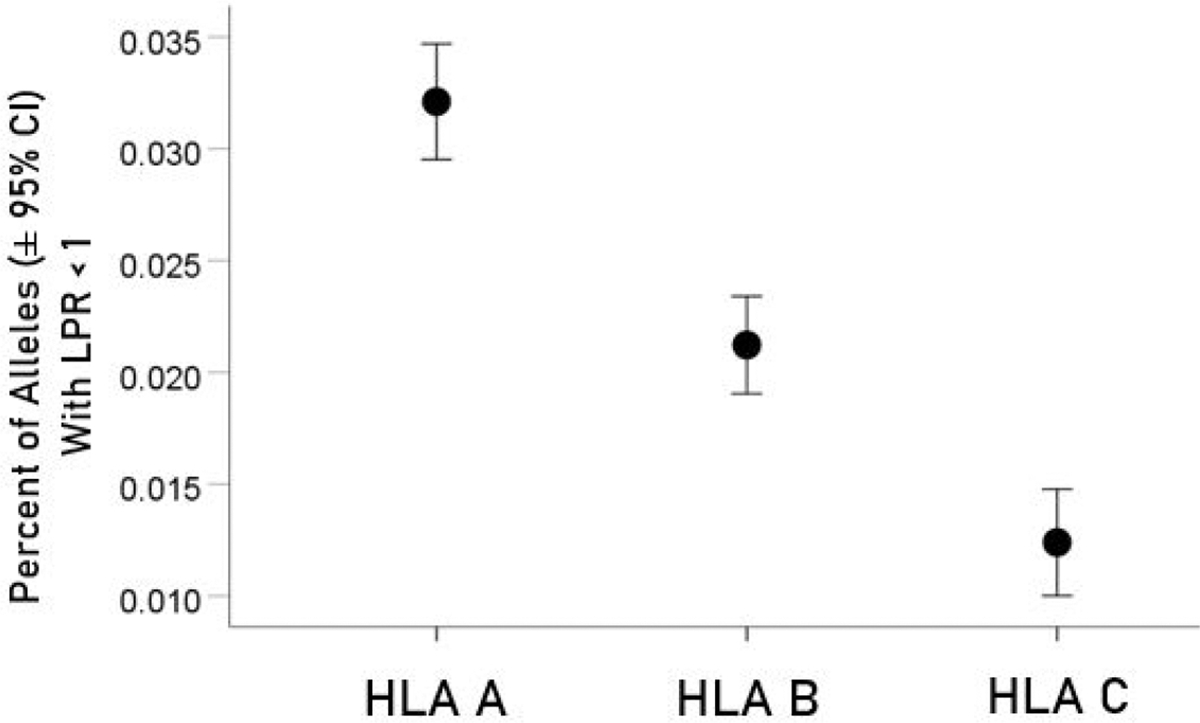
Mean percentages (± 95% CI) of alleles with LPR < 1 for the 3 genes. See text for details of statistical comparisons

**Figure 4.**
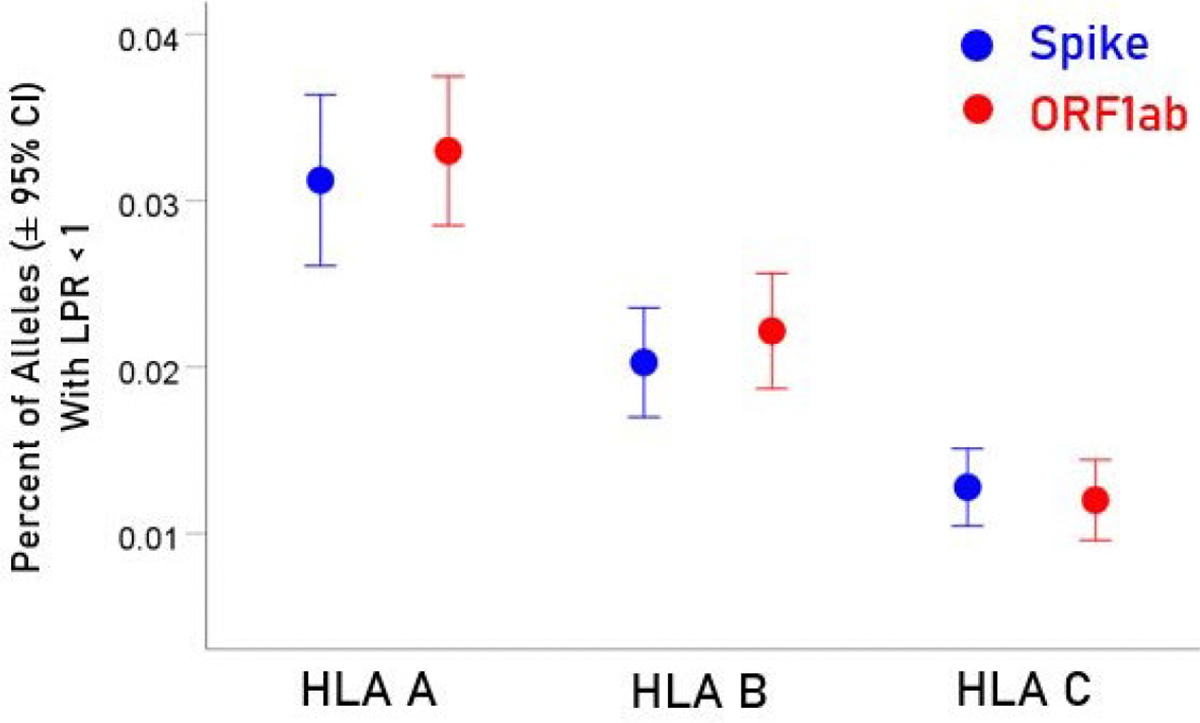
Mean percentages (± 95% CI) of alleles with LPR < 1 for the 3 genes and 2 proteins. See text for details of statistical comparisons

For each allele, the percentage of LPR < 1 across all protein epitopes was calculated and the effect of protein, gene and their interaction was evaluated using an ANOVA where protein and gene were fixed factors. The effect of protein was not statistically significant [Figure 2; *F*_(1,116)_ = 0.476, *P* = 0.476] but the effect of gene was highly statistically significant [Figure 3; *F*_(2,116)_ = 61.599, *P* = 5.90 × 10^−19^]. Specifically, the percentage of LPR < 1 above was significantly higher in gene *A* than gene *B* (*P* = 3.84 × 10^−9^, ANOVA) and gene *C* (*P* = 6.07 × 10^−20^), and in gene *B* than in gene *C* (*P* = 3.33 × 10^−7^). Finally, the protein × gene interaction term was not statistically significant [Figure 4; *F*_(2,116)_ = 0.397, *P* = 0.673].

### Distribution of affinities across protein sequences

**Figure 5.**
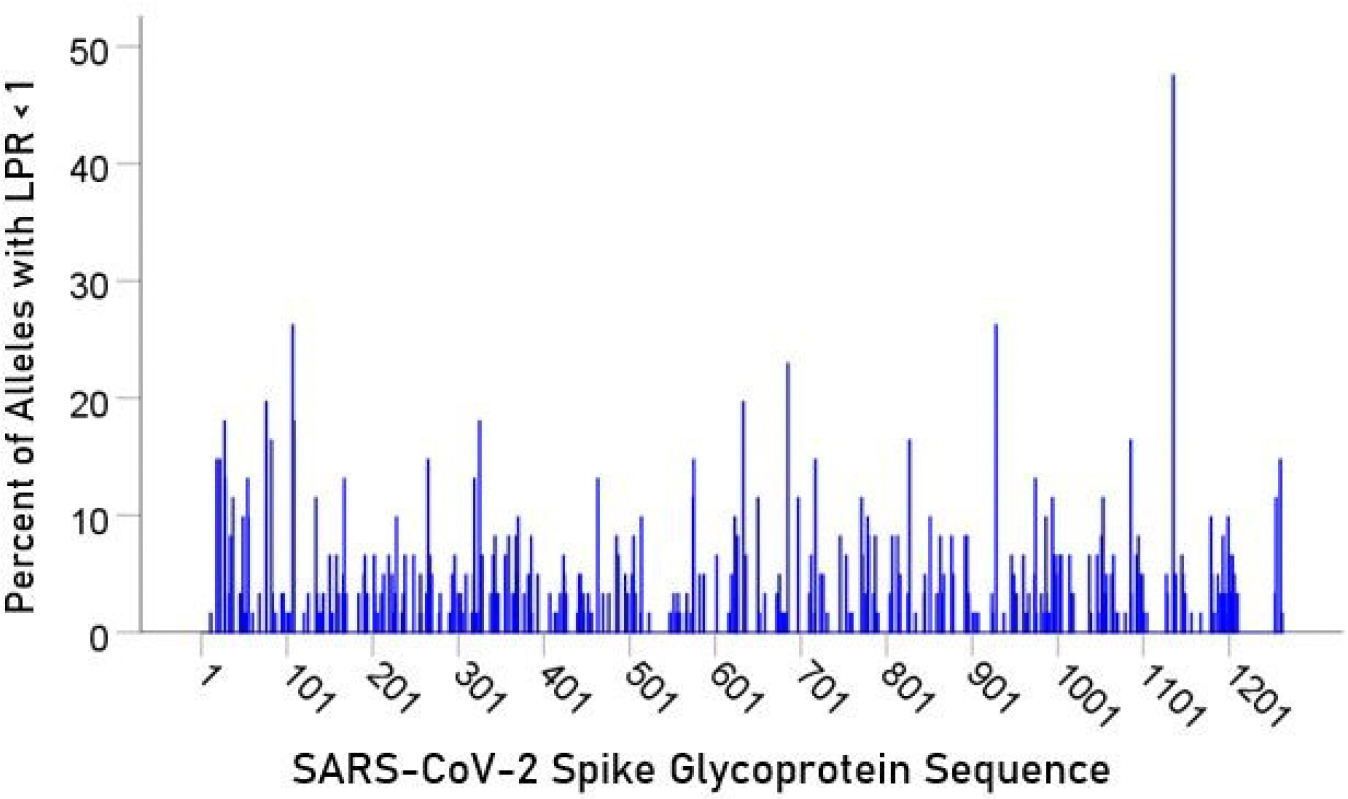
Distribution of alleles with LPR < 1 for an epitope along the S protein sequence

**Figure 6.**
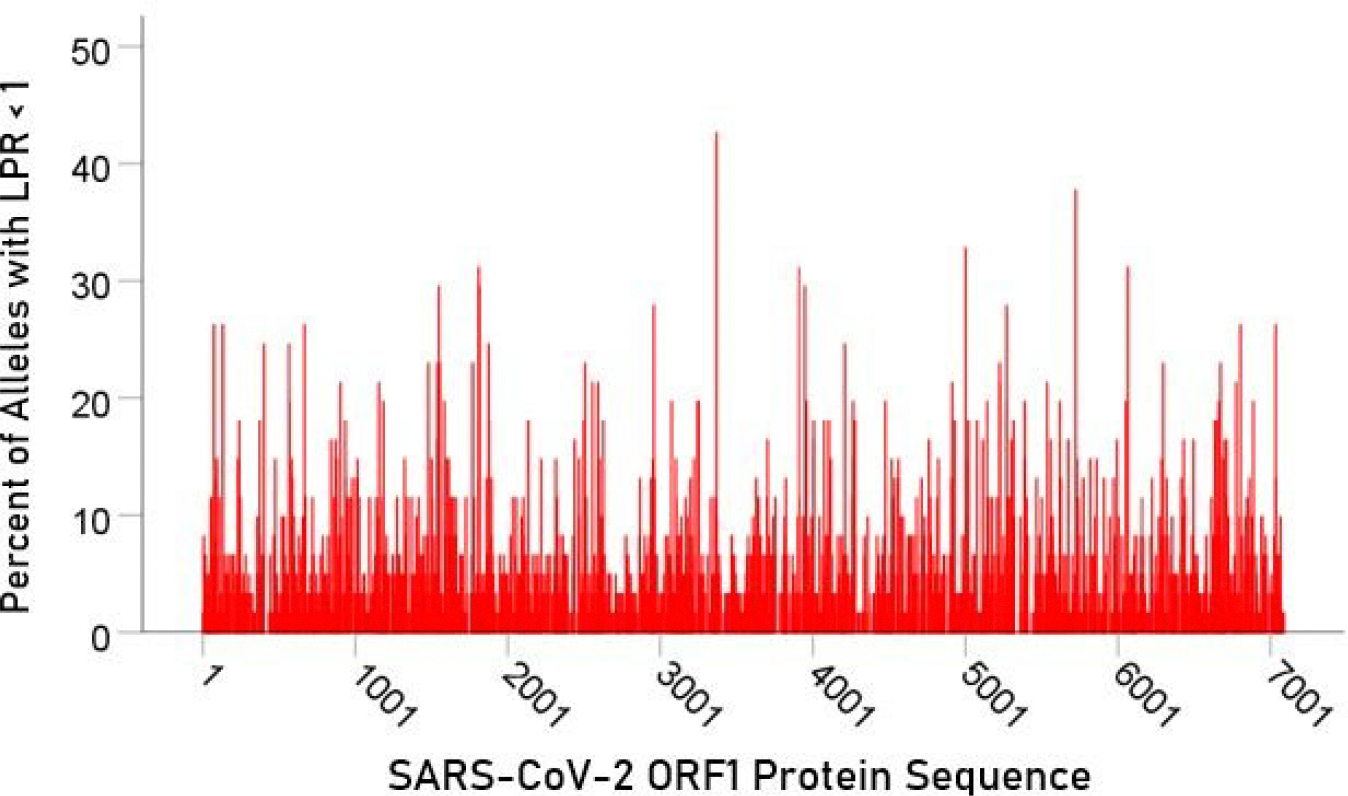
Distribution of alleles with LPR < 1 for an epitope along the S protein sequence

For each epitope tested, the percentage of alleles with LPR < 1 was calculated and plotted along the sequence of the S protein (Figure 5) and ORF1ab polyprotein (Figure 6). It was observed that high affinity scores exist throughout the entire protein sequence for both the S protein and ORF1ab.

### Application to individuals

Each individual carries 6 HLA class I alleles, 2 from each of the 3 classical genes (*A*, *B*, *C*). The average “goodness” of HLA affinities (LPR < 1 across epitopes) for each allele is given in Table 2, from which the average LPR < 1 percent across the 6 alleles carried by an individual can be calculated. (Since this study was focused on 61 common alleles, the LPR < 1 value for other alleles will need to be calculated.)

### Application to populations

The overall “goodness” of HLA class I affinities for a population comprising a set of specific alleles can be calculated as the mean of the products of the percent LPR < 1 value (Table 2) times the corresponding allele frequency (Table 1). (Since this study was focused on 61 common alleles, the LPR < 1 value for other alleles will need to be calculated for specific populations.) For a particular gene, the formula is:

(1) $$Q^{P}=\frac{1}{N}\sum_{i}^{i=1,N}a_{i}f_{i}^{P}$$

where $Q^{P}$ is the overall affinity for population P consisting of N alleles i of that gene, with percent LPR < 1 $a_{i}$ and frequency in the population $f_{i}^{P}$. For the global set of alleles analyzed in this study, and using the allele frequencies from Table 1 and the average (of the two proteins) percent LPR < 1 (last column of Table 2), we obtained the following $Q^{P}$ for the 3 genes. For genes A,B and $C,\;Q^{P}=0.001571,\;0.000678$, and 0.000608, respectively.

## Discussion

In the current study, we investigated the relations between two important proteins of the SARS-CoV-2 proteome (the S protein, which is the main antigenic molecule of the virus [9] and ORF1ab polyprotein), and 61 common HLA class I alleles. There were three major aims in this *in silico* study, as follows. First, we sought to quantify the binding affinity of suitable fragments (linear epitopes) of the S protein and ORF1ab polyprotein to the 61 HLA class I alleles in an exhaustive manner using the sliding epitope window approach [3, 4]; second, we aimed to assess differences in allele binding affinities between the two proteins, among the three HLA class I genes, and their interaction; and third, we sought to assess the distribution of binding affinities across the sequences of the two proteins. The results obtained were clear cut. First, all 61 alleles exhibited good binding affinities to epitopes of both the spike and ORF1ab proteins; second, the percentage of good binding affinities (LPR < 1) did not differ significantly between the two proteins (Figure 2) but did so among the genes, with gene *A* having the best binding performance, followed by gene *B* and gene *C* (*A* > *B* > *C*) (Figure 3); third, there was no significant interaction between proteins and genes (ANOVA), indicating a similar effect of the genes in both proteins (Figure 4); and fourth, good binding affinities were distributed throughout the two protein sequences (Figure 5 and 6). These findings indicate that the HLA class I system is well suited to contribute effectively to the elimination of SARS-CoV-2 at the early phase of its entry to the body in otherwise immunocompetent individuals. The fact that good binding affinities are distributed throughout both protein sequences provides for robustness in the fight against the virus, since it almost guarantees a good possibility that linear epitopes of the proteins can bind to class I molecules irrespective of the location of glycoprotein cleavage by cell proteases.

With respect to the ORF1ab polyprotein, it should be mentioned that CD8^+^ T cells recognize peptides presented by class I HLA surface receptors, and such peptides most commonly originate from cytosolic viral proteins following proteasomal degradation by host intracellular antigen processing pathways. Since T cells do not recognize surface antigens, in contrast to B cells and the antibodies that are produced and secreted by them, ORF1ab provides intracellular peptides that originate from essential enzymes and are thus more conserved. Proteins encoded by SARS-CoV-2 ORF1ab are more conserved among coronaviruses relative to the S protein, which has lower homology [10, 11]. ORF1ab was included in this study because it is the largest viral gene in the *Coronaviridae* family and encodes both non-structural and accessory proteins that are less susceptible to evolutionary pressure and hence mutational changes, in contrast to the S protein.

Nguyen and colleagues [12] have published a similar *in silico* analysis of viral peptide binding affinities to HLA*-A*, *-B* and *-C* loci. In their study, the entire SARS-CoV-2 proteome was investigated, whereas our study focused on the ORF1ab polyprotein and S protein, which together comprise a considerably smaller number of potential *n*-mers epitopes. Furthermore, sequence homology was used to identify highly conserved sequences in SARS-CoV-2 from other common coronaviruses that could function as epitopes. Here, we did not specifically investigate for sequence homology of the S protein or ORF1ab polyprotein with other coronaviruses as this has been documented in previous studies [10, 11]. Interestingly, Nguyen and colleagues [12] report that the HLA*-A* and HLA*-C* alleles exhibit the highest and lowest capacities to present SARS-CoV-2 antigens, respectively. This ranking is corroborated in our study (Figure 3) focusing on multiple proteomic elements (the S protein and the ensemble of intracellular proteins encoded in ORF1ab) as opposed to the entire viral proteome. With respect to specific alleles with the highest and lowest binding affinities to SARS-CoV-2 antigens, our results differ from those of Nguyen et al. [12] who report that B*15:03 is a better binder than B*46:01. This difference could be due to the smaller number of *n*-mers that we used (8,354 10-mers peptides that linearly partitioned the ORF1ab polyprotein and S protein *vs.* 32,257 peptide samples from the full SARS-CoV-2 proteome used by Nguyen and colleagues [12]).

Nelde and colleagues [13] recently published a study in which they identified HLA class I and HLA-DR binding peptides using experimental and computational methods. Their prediction workflow incorporates the NetMHCpan algorithm which was used in our study and reports immunogenic SARS-CoV-2-derived class I T cell epitopes. Among these are two 9-mers, LTDEMIAQY (HLA-A*01) and QYIKWPWYI (HLA-A*24), both of which have very high binding affinity scores in our analysis as well. The authors [13] used ELISA-type assays with *in vitro* amplified T cells from patients convalescing from SARS-CoV-2 infection and donors who were never exposed to the virus. Nelde et al. [13] reported that 29% of SARS-CoV-2-derived HLA class I binding peptides were validated as naturally occurring T cell epitopes, showing that NetMHCpan is useful as a predictive algorithm for class I immunogenicity.

To the best of our knowledge, there have been only a limited number of published studies (by Nguyen et al. [12] and Nelde et al. [13]) that investigated viral protein binding affinity across a wide range of HLA alleles on a per-allele basis using predictive algorithms. Our present study builds on previous work [3, 4] and examines the relationship between two viral proteins of the SARS-CoV-2 proteome and HLA class I antigen presentation, with the results discussed above. Finally, we devised metric ($Q^{P}$) to calculate the overall binding affinity of a gene in a specific population by taking into account both the goodness of binding affinities of alleles of a gene and their frequency of occurrence in the population. Using the global allele frequencies of HLA class I alleles (Table 1), we found that the HLA class I *A* gene had the best overall binding performance, followed by genes B and C. It is possible that this result could differ for different populations (e.g., ethnicities, locations on Earth, etc.), thus providing a population-specific measure of how well the HLA class I makeup of that population could contribute to SARS-CoV-2 elimination at the early stage of fighting the viral infection.

We acknowledge, however, an important limitation to our work. This study was performed exclusively *in silico* and thus the data presented are subject to any constraints that may affect HLA binding affinity predictive tools (NetMHCpan EL in this case). Although important to consider, this limitation is mitigated by the fact that NetMHCpan and other binding affinity prediction algorithms included in the IEDB suite are trained using experimental datasets. In absence of clinical and patient data regarding actual Covid-19 cases, we are unable to assess the impact of disease-modifying risk factors such as age and clinical comorbidities [14, 15] on HLA class I-conferred neutralization of the SARS-CoV-2 virus. In spite of this limitation, we believe that computational studies centered around binding affinity prediction can offer important insights as to how HLA genotype affects viral susceptibility, which can hopefully guide vaccination strategies.

## Supplementary Material

Supplementary Material

## Figures and Tables

**Table 1.** HLA class I alleles used, ordered by their global frequencies in descending order (see text for details)

Gene *A*		Gene *B*		Gene *C*	

Allele	Frequency	Allele	Frequency	Allele	Frequency
A*02:01	0.19257	B*07:02	0.08271	C*04:01	0.13265
A*01:01	0.10933	B*08:01	0.06555	C*07:02	0.12442
A*24:02	0.09998	B*35:01	0.05815	C*07:01	0.12235
A*03:01	0.09324	B*44:02	0.05244	C*06:02	0.08360
A*11:01	0.07282	B*44:03	0.04972	C*04:43	0.08053
A*23:01	0.03373	B*51:01	0.04963	C*03:04	0.06754
A*68:01	0.03305	B*40:01	0.04177	C*05:01	0.05914
A*26:01	0.03199	B*15:01	0.03966	C*01:02	0.04659
A*29:02	0.02847	B*18:01	0.03814	C*02:02	0.04371
A*32:01	0.02784	B*14:02	0.02643	C*16:01	0.04129
A*31:01	0.02769	B*57:01	0.02553	C*12:03	0.03938
A*68:02	0.01941	B*53:01	0.02361	C*08:02	0.03282
A*02:06	0.01365	B*58:01	0.02326	C*15:02	0.02997
A*33:01	0.01282	B*27:05	0.02240	C*17:01	0.01957
A*25:01	0.01255	B*13:02	0.02061	C*14:02	0.01949
A*02:02	0.01097	B*40:02	0.01981	C*08:01	0.01833
A*02:05	0.01065	B*38:01	0.01872	C*12:02	0.01678
		B*49:01	0.01844	C*03:02	0.01492
		B*35:03	0.01644	C*07:04	0.01196
		B*45:01	0.01291	C*17:03	0.01037
		B*15:03	0.01247		
		B*46:01	0.01204		
		B*37:01	0.01181		
		B*39:01	0.01011		

**Table 2.** Percentages of LPR < 1 (across all epitopes of a protein) for all alleles studied, ranked from highest to lowest

Allele	Spike	ORF1ab	Average

A*29:02	0.0493	0.0497	0.0495
A*01:01	0.0441	0.0442	0.0441
A*26:01	0.0415	0.0418	0.0417
A*25:01	0.0428	0.0398	0.0413
A*24:02	0.0402	0.0363	0.0383
A*23:01	0.0415	0.0347	0.0381
B*15:01	0.0350	0.0412	0.0381
A*11:01	0.0337	0.0375	0.0356
A*03:01	0.0286	0.0386	0.0336
A*69:01	0.0299	0.0356	0.0327
B*46:01	0.0312	0.0340	0.0326
B*53:01	0.0324	0.0280	0.0302
A*02:02	0.0247	0.0331	0.0289
B*35:01	0.0286	0.0268	0.0277
B*57:01	0.0221	0.0331	0.0276
B*58:01	0.0208	0.0319	0.0263
A*68:01	0.0221	0.0291	0.0256
C*14:02	0.0234	0.0277	0.0256
B*44:03	0.0234	0.0270	0.0252
B*44:02	0.0234	0.0266	0.0250
A*32:01	0.0208	0.0275	0.0241
A*02:05	0.0234	0.0238	0.0236
B*07:02	0.0247	0.0217	0.0232
A*02:01	0.0195	0.0266	0.0230
A*31:01	0.0260	0.0197	0.0228
B*35:03	0.0234	0.0222	0.0228
A*33:01	0.0221	0.0213	0.0217
B*49:01	0.0221	0.0206	0.0213
B*15:03	0.0195	0.0231	0.0213
A*02:06	0.0208	0.0217	0.0212
B*40:01	0.0169	0.0254	0.0212
B*45:01	0.0234	0.0185	0.0209
B*27:05	0.0234	0.0183	0.0208
B*40:02	0.0182	0.0208	0.0195
B*51:01	0.0195	0.0192	0.0193
B*38:01	0.0182	0.0199	0.0190
C*02:02	0.0182	0.0183	0.0182
C*03:02	0.0195	0.0160	0.0177
B*13:02	0.0182	0.0160	0.0171
C*07:02	0.0195	0.0146	0.0170
C*01:02	0.0182	0.0106	0.0144
B*18:01	0.0130	0.0153	0.0141
C*12:02	0.0169	0.0113	0.0141
C*17:01	0.0143	0.0125	0.0134
C*17:03	0.0143	0.0125	0.0134
B*39:01	0.0091	0.0169	0.0130
C*05:01	0.0078	0.0169	0.0123
C*08:02	0.0091	0.0139	0.0115
C*07:01	0.0130	0.0088	0.0109
C*04:01	0.0104	0.0113	0.0109
C*04:43	0.0104	0.0113	0.0109
C*15:02	0.0104	0.0095	0.0099
C*03:04	0.0078	0.0120	0.0099
B*08:01	0.0091	0.0104	0.0097
B*37:01	0.0091	0.0097	0.0094
C*16:01	0.0104	0.0081	0.0092
C*12:03	0.0104	0.0065	0.0084
C*07:04	0.0091	0.0069	0.0080
C*06:02	0.0078	0.0053	0.0066
C*08:01	0.0052	0.0062	0.0057
B*14:02	0.0026	0.0058	0.0042

## Data Availability

Not applicable.

## Abbreviations

AA: amino acid
ANOVA: analysis of variance
CI: confidence interval
COVID-19: coronavirus disease 2019
HLA: human leukocyte antigen
LPR: lowest (minimum) percentile rank
ORF1ab: open reading frame 1ab
S protein: spike glycoprotein
SARS-CoV-2: severe acute respiratory syndrome coronavirus 2
