# Supplementary material for: In silico investigation of binding affinities between human leukocyte antigen class I molecules and SARS-CoV-2 virus spike and ORF1ab proteins
Source: Explor Immunol. Author manuscript; Available in PMC 2025 Jul 31. (PMC12311910; doi:10.37349/ei.2021.00003)
Supplement: Supplementary Material [file NIHMS2079217-supplement-Supplementary_Material.pdf]

Supplemental

Table A. LPR < 1 values for all alleles and associated epitope sequences for the spike glycoprotein. LPR values are ranked from lowest (highest affinity) to highest (lowest affinity)

Allele	Start position	End position	Epitope sequence	Lowest Percentile Rank
A*01:01	864	874	LLTDEMIAQY	0.01
B*27:05	77	87	KRFDNPVLPF	0.01
B*44:02	95	105	TEKSNIIRGW	0.01
A*23:01	1,066	1,076	TYVPAQEKNF	0.02
A*24:02	1,066	1,076	TYVPAQEKNF	0.02
A*26:01	780	790	EVFAQVKQIY	0.02
B*44:03	95	105	TEKSNIIRGW	0.02
B*53:01	55	65	FLPFFSNVTW	0.02
C*07:01	77	87	KRFDNPVLPF	0.02
A*03:01	408	418	RQIAPGQTGK	0.03
A*03:01	786	796	KQIYKTPPIK	0.03
A*23:01	788	798	IYKTPPIKDF	0.03
A*24:02	159	169	VYSSANNCTF	0.03
A*24:02	368	378	LYNSASFSTF	0.03
A*24:02	788	798	IYKTPPIKDF	0.03
A*25:01	780	790	EVFAQVKQIY	0.03
B*53:01	229	239	LPIGINITRF	0.03
A*68:01	394	404	NVYADSFVIR	0.04
B*15:01	634	644	RVYSTGSNVF	0.04
A*01:01	828	838	LADAGFIKQY	0.05
A*23:01	368	378	LYNSASFSTF	0.05
A*26:01	191	201	EFVFKNIDGY	0.05
B*18:01	464	474	FERDISTEIY	0.05
B*44:02	297	307	SETKCTLKSF	0.05
C*04:01	1,136	1,146	TVYDPLQPEL	0.05
C*04:43	1,136	1,146	TVYDPLQPEL	0.05
A*23:01	159	169	VYSSANNCTF	0.06
A*26:01	360	370	NCVADYSVLY	0.06
A*26:01	686	696	SVASQSIIAY	0.06
A*31:01	35	45	GVYYPDKVFR	0.06
A*32:01	634	644	RVYSTGSNVF	0.06

A*68:02	1,136	1,146	TVYDPLQPEL	0.06
B*38:01	624	634	IHADQLTPTW	0.06
B*57:01	344	354	ATRFASVYAW	0.06
A*01:01	1,197	1,207	LIDLQELGKY	0.07
A*31:01	558	568	KFLPFQQFGR	0.07
A*33:01	394	404	NVYADSFVIR	0.07
A*68:01	637	647	STGSNVFQTR	0.07
B*35:01	229	239	LPIGINITRF	0.07
B*44:03	297	307	SETKCTLKSF	0.07
B*45:01	1,016	1,026	AEIRASANLA	0.07
B*53:01	83	93	VLPFNDGVYF	0.07
B*53:01	320	330	VQPTESIVRF	0.07
B*58:01	624	634	IHADQLTPTW	0.07
C*07:02	77	87	KRFDNPVLPF	0.07
A*01:01	603	613	NTSNQVAVLY	0.08
A*02:02	975	985	SVLNDILSRL	0.08
A*11:01	724	734	TEILPVSMTK	0.08
A*25:01	191	201	EFVFKNIDGY	0.08
A*25:01	583	593	EILDITPCSF	0.08
A*31:01	319	329	RVQPTESIVR	0.08
B*27:05	814	824	KRSFIEDLLF	0.08
B*44:03	747	757	TECSNLLLQY	0.08
C*01:02	214	224	RDLPQGFSAL	0.08
A*02:05	1,136	1,146	TVYDPLQPEL	0.09
A*23:01	488	498	CYFPLQSYGF	0.09
B*40:01	987	997	VEAEVQIDRL	0.09
B*45:01	1,181	1,191	KEIDRLNEVA	0.09
B*51:01	1,052	1,062	FPQSAPHGVV	0.09
C*06:02	77	87	KRFDNPVLPF	0.09
A*01:01	28	38	YTNSFTRGVY	0.1
A*01:01	161	171	SSANNCTFEY	0.1
A*01:01	440	450	NLDSKVGGNY	0.1
A*03:01	1,064	1,074	HVTYVPAQEK	0.1
A*23:01	1,208	1,218	QYIKWPWYIW	0.1
A*25:01	686	696	SVASQSIIAY	0.1
A*32:01	344	354	ATRFASVYAW	0.1
B*15:01	698	708	SLGAENSVAY	0.1

B*53:01	56	66	LPFFSNVTWF	0.1
B*53:01	249	259	LTPGDSSSGW	0.1
C*07:04	576	586	VRDPQTLEIL	0.1
A*23:01	143	153	VYYHKNNKSW	0.11
B*07:02	1,261	1,271	SEPVLKGVKL	0.11
B*13:02	852	862	AQKFNGLTVL	0.11
B*27:05	1,038	1,048	KRVDFCGKGY	0.11
A*02:06	1,136	1,146	TVYDPLQPEL	0.12
A*03:01	269	279	YLQPRTFLLK	0.12
A*03:01	724	734	TEILPVSMTK	0.12
A*26:01	583	593	EILDITPCSF	0.12
A*26:01	603	613	NTSNQVAVLY	0.12
B*15:01	686	696	SVASQSIIAY	0.12
B*35:01	895	905	QIPFAMQMAY	0.12
B*44:02	747	757	TECSNLLLQY	0.12
B*45:01	1,071	1,081	QEKNFTTAPA	0.12
B*53:01	1,139	1,149	DPLQPELDSF	0.12
C*06:02	326	336	IVRFPNITNL	0.12
A*11:01	408	418	RQIAPGQTGK	0.13
A*11:01	1,064	1,074	HVTYVPAQEK	0.13
A*24:02	143	153	VYYHKNNKSW	0.13
A*24:02	203	213	IYSKHTPINL	0.13
A*24:02	488	498	CYFPLQSYGF	0.13
A*29:02	496	506	GFQPTNGVGY	0.13
A*33:01	448	458	NYNYLYRLFR	0.13
B*46:01	686	696	SVASQSIIAY	0.13
C*01:02	860	870	VLPPLLTDEM	0.13
C*17:01	1,136	1,146	TVYDPLQPEL	0.13
C*17:03	1,136	1,146	TVYDPLQPEL	0.13
A*24:02	1,208	1,218	QYIKWPWYIW	0.14
A*29:02	151	161	SWMESEFRVY	0.14
A*31:01	637	647	STGSNVFQTR	0.14
A*33:01	228	238	DLPIGINITR	0.14
A*68:02	717	727	NFTISVTTEI	0.14
A*68:02	1,188	1,198	EVAKNLNESL	0.14
B*13:02	626	636	ADQLTPTWRV	0.14
B*35:01	56	66	LPFFSNVTWF	0.14

B*45:01	1,070	1,080	AQEKNFTTAP	0.14
A*23:01	268	278	GYLQPRTFLL	0.15
A*31:01	346	356	RFASVYAWNR	0.15
A*01:01	865	875	LTDEMIAQYT	0.16
A*23:01	1,094	1,104	VFVSNGTHWF	0.16
B*35:01	83	93	VLPFNDGVYF	0.16
B*35:03	229	239	LPIGINITRF	0.16
B*40:01	1,256	1,266	FDEDDSEPVL	0.16
B*53:01	329	339	FPNITNLCPF	0.16
B*57:01	624	634	IHADQLTPTW	0.16
B*58:01	344	354	ATRFASVYAW	0.16
C*08:02	1,136	1,146	TVYDPLQPEL	0.16
A*23:01	1,211	1,221	KWPWYIWLGF	0.17
A*24:02	268	278	GYLQPRTFLL	0.17
A*29:02	686	696	SVASQSIIAY	0.17
B*40:01	1,261	1,271	SEPVLKGVKL	0.17
B*40:02	987	997	VEAEVQIDRL	0.17
B*58:01	814	824	KRSFIEDLLF	0.17
C*01:02	23	33	QLPPAYTNSF	0.17
A*03:01	1,196	1,206	SLIDLQELGK	0.18
A*24:02	634	644	RVYSTGSNVF	0.18
A*24:02	1,094	1,104	VFVSNGTHWF	0.18
A*26:01	28	38	YTNSFTRGVY	0.18
A*29:02	360	370	NCVADYSVLY	0.18
A*31:01	237	247	RFQTLLALHR	0.18
B*27:05	326	336	IVRFPNITNL	0.18
B*27:05	998	1,008	TGRLQSLQTY	0.18
B*35:01	329	339	FPNITNLCPF	0.18
B*53:01	624	634	IHADQLTPTW	0.18
C*03:04	929	939	SAIGKIQDSL	0.18
C*05:01	108	118	TTLDSKTQSL	0.18
C*08:02	108	118	TTLDSKTQSL	0.18
A*02:05	975	985	SVLNDILSRL	0.19
A*68:01	1,064	1,074	HVTYVPAQEK	0.19
B*07:02	38	48	YPDKVFRSSV	0.19
B*46:01	266	276	YVGYLQPRTF	0.19
C*04:01	1,137	1,147	VYDPLQPELD	0.19

C*04:43	1,137	1,147	VYDPLQPELD	0.19
C*05:01	109	119	TLDSKTQSLL	0.19
C*05:01	1,136	1,146	TVYDPLQPEL	0.19
C*14:02	144	154	YYHKNNKSWM	0.19
A*01:01	651	661	IGAEHVNNSY	0.2
A*01:01	1,146	1,156	DSFKEELDKY	0.2
A*02:02	386	396	KLNDLCFTNV	0.2
A*02:02	424	434	KLPDDFTGCV	0.2
A*02:05	424	434	KLPDDFTGCV	0.2
A*23:01	203	213	IYSKHTPINL	0.2
A*24:02	1,211	1,221	KWPWYIWLGF	0.2
A*25:01	360	370	NCVADYSVLY	0.2
A*68:01	777	787	NTQEVFAQVK	0.2
B*15:03	464	474	FERDISTEIY	0.2
B*44:03	464	474	FERDISTEIY	0.2
B*57:01	814	824	KRSFIEDLLF	0.2
A*02:06	424	434	KLPDDFTGCV	0.21
A*03:01	805	815	ILPDPSKPSK	0.21
A*25:01	1,188	1,198	EVAKNLNESL	0.21
A*26:01	442	452	DSKVGGNYNY	0.21
A*29:02	1,101	1,111	HWFVTQRNFY	0.21
A*31:01	264	274	AYYVGYLQPR	0.21
A*31:01	310	320	KGIYQTSNFR	0.21
A*68:01	69	79	HVSGTNGTKR	0.21
A*68:01	228	238	DLPIGINITR	0.21
A*68:01	568	578	DIADTTDAVR	0.21
A*68:01	1,098	1,108	NGTHWFVTQR	0.21
B*13:02	964	974	KQLSSNFGAI	0.21
B*18:01	747	757	TECSNLLLQY	0.21
B*35:03	1,052	1,062	FPQSAPHGVV	0.21
B*39:01	576	586	VRDPQTLEIL	0.21
B*40:01	1,015	1,025	AAEIRASANL	0.21
B*49:01	772	782	VEQDKNTQEV	0.21
C*05:01	575	585	AVRDPQTLEI	0.21
C*14:02	159	169	VYSSANNCTF	0.21
C*14:02	368	378	LYNSASFSTF	0.21
A*24:02	23	33	QLPPAYTNSF	0.22

A*33:01	1,098	1,108	NGTHWFVTQR	0.22
B*15:01	852	862	AQKFNGLTVL	0.22
B*35:01	686	696	SVASQSIIAY	0.22
B*45:01	470	480	TEIYQAGSTP	0.22
C*08:02	109	119	TLDSKTQSLL	0.22
A*02:01	424	434	KLPDDFTGCV	0.23
A*11:01	35	45	GVYYPDKVFR	0.23
A*11:01	826	836	VTLADAGFIK	0.23
B*38:01	576	586	VRDPQTLEIL	0.23
B*40:02	1,261	1,271	SEPVLKGVKL	0.23
B*45:01	659	669	SYECDIPIGA	0.23
B*49:01	779	789	QEVFAQVKQI	0.23
C*03:04	1,136	1,146	TVYDPLQPEL	0.23
C*07:01	576	586	VRDPQTLEIL	0.23
A*01:01	414	424	QTGKIADYNY	0.24
A*02:01	386	396	KLNDLCFTNV	0.24
A*25:01	1,136	1,146	TVYDPLQPEL	0.24
A*26:01	1,146	1,156	DSFKEELDKY	0.24
A*68:02	718	728	FTISVTTEIL	0.24
B*15:03	77	87	KRFDNPVLPF	0.24
B*38:01	47	57	VLHSTQDLFL	0.24
B*44:02	464	474	FERDISTEIY	0.24
C*01:02	1,054	1,064	QSAPHGVVFL	0.24
C*07:01	326	336	IVRFPNITNL	0.24
C*07:02	576	586	VRDPQTLEIL	0.24
A*03:01	453	463	YRLFRKSNLK	0.25
A*23:01	23	33	QLPPAYTNSF	0.25
B*35:01	1,139	1,149	DPLQPELDSF	0.25
B*44:02	553	563	TESNKKFLPF	0.25
B*46:01	634	644	RVYSTGSNVF	0.25
B*51:01	38	48	YPDKVFRSSV	0.25
B*53:01	383	393	SPTKLNDLCF	0.25
B*57:01	1,093	1,103	GVFVSNGTHW	0.25
A*01:01	135	145	FCNDPFLGVY	0.26
A*01:01	136	146	CNDPFLGVYY	0.26
A*02:01	515	525	FELLHAPATV	0.26
A*24:02	267	277	VGYLQPRTFL	0.26

A*31:01	348	358	ASVYAWNRKR	0.26
B*15:03	634	644	RVYSTGSNVF	0.26
B*46:01	28	38	YTNSFTRGVY	0.26
B*46:01	698	708	SLGAENSVAY	0.26
B*49:01	1,180	1,190	QKEIDRLNEV	0.26
B*58:01	1,093	1,103	GVFVSNGTHW	0.26
C*07:04	1,136	1,146	TVYDPLQPEL	0.26
A*01:01	732	742	TKTSVDCTMY	0.27
A*68:01	35	45	GVYYPDKVFR	0.27
A*68:01	1,005	1,015	QTYVTQQLIR	0.27
A*68:02	967	977	SSNFGAISSV	0.27
B*15:01	320	330	VQPTESIVRF	0.27
B*37:01	214	224	RDLPQGFSAL	0.27
B*58:01	49	59	HSTQDLFLPF	0.27
A*02:02	995	1,005	RLITGRLQSL	0.28
A*29:02	28	38	YTNSFTRGVY	0.28
A*29:02	603	613	NTSNQVAVLY	0.28
A*68:02	1,128	1,138	VVIGIVNNTV	0.28
B*15:03	852	862	AQKFNGLTVL	0.28
B*27:05	235	245	ITRFQTLLAL	0.28
B*35:03	620	630	VPVAIHADQL	0.28
B*38:01	1,087	1,097	AHFPREGVFV	0.28
A*01:01	257	267	GWTAGAAAYY	0.29
A*11:01	1,196	1,206	SLIDLQELGK	0.29
A*23:01	267	277	VGYLQPRTFL	0.29
A*29:02	257	267	GWTAGAAAYY	0.29
B*07:02	506	516	QPYRVVVLSF	0.29
B*15:01	270	280	LQPRTFLLKY	0.29
B*15:01	1,200	1,210	LQELGKYEQY	0.29
B*35:03	56	66	LPFFSNVTWF	0.29
B*35:03	294	304	DPLSETKCTL	0.29
B*57:01	97	107	KSNIIRGWIF	0.29
C*02:02	28	38	YTNSFTRGVY	0.29
C*08:01	1,136	1,146	TVYDPLQPEL	0.29
A*03:01	924	934	ANQFNSAIGK	0.3
A*23:01	634	644	RVYSTGSNVF	0.3
A*32:01	34	44	RGVYYPDKVF	0.3

A*68:01	88	98	DGVYFASTEK	0.3
A*68:02	258	268	WTAGAAAYYV	0.3
A*68:02	1,095	1,105	FVSNGTHWFV	0.3
B*13:02	925	935	NQFNSAIGKI	0.3
B*27:05	344	354	ATRFASVYAW	0.3
B*46:01	135	145	FCNDPFLGVY	0.3
B*57:01	1,203	1,213	LGKYEQYIKW	0.3
B*58:01	249	259	LTPGDSSSGW	0.3
C*07:02	788	798	IYKTPPIKDF	0.3
C*07:02	1,136	1,146	TVYDPLQPEL	0.3
C*14:02	788	798	IYKTPPIKDF	0.3
A*01:01	360	370	NCVADYSVLY	0.31
A*01:01	499	509	PTNGVGYQPY	0.31
A*25:01	603	613	NTSNQVAVLY	0.31
A*31:01	676	686	TQTNSPRRAR	0.31
A*31:01	991	1,001	VQIDRLITGR	0.31
A*32:01	1,093	1,103	GVFVSNGTHW	0.31
B*37:01	1,261	1,271	SEPVLKGVKL	0.31
B*38:01	1,100	1,110	THWFVTQRNF	0.31
B*40:01	1,194	1,204	NESLIDLQEL	0.31
B*45:01	339	349	GEVFNATRFA	0.31
B*49:01	515	525	FELLHAPATV	0.31
B*53:01	506	516	QPYRVVVLSF	0.31
B*57:01	879	889	AGTITSGWTF	0.31
C*03:02	135	145	FCNDPFLGVY	0.31
C*04:01	77	87	KRFDNPVLPF	0.31
C*04:43	77	87	KRFDNPVLPF	0.31
C*14:02	634	644	RVYSTGSNVF	0.31
A*02:01	1,047	1,057	YHLMSFPQSA	0.32
A*03:01	310	320	KGIYQTSNFR	0.32
B*27:05	999	1,009	GRLQSLQTYV	0.32
B*35:01	55	65	FLPFFSNVTW	0.32
B*35:03	38	48	YPDKVFRSSV	0.32
B*45:01	867	877	DEMIAQYTSA	0.32
B*51:01	727	737	LPVSMTKTSV	0.32
C*02:02	266	276	YVGYLQPRTF	0.32
C*03:02	266	276	YVGYLQPRTF	0.32

C*12:03	929	939	SAIGKIQDSL	0.32
A*11:01	369	379	YNSASFSTFK	0.33
A*11:01	1,019	1,029	RASANLAATK	0.33
A*23:01	320	330	VQPTESIVRF	0.33
A*29:02	29	39	TNSFTRGVYY	0.33
A*68:02	1,168	1,178	DISGINASVV	0.33
B*39:01	1,047	1,057	YHLMSFPQSA	0.33
B*44:03	553	563	TESNKKFLPF	0.33
C*01:02	524	534	VCGPKKSTNL	0.33
C*02:02	828	838	LADAGFIKQY	0.33
C*08:02	575	585	AVRDPQTLEI	0.33
A*02:02	1,003	1,013	SLQTYVTQQL	0.34
A*26:01	257	267	GWTAGAAAYY	0.34
A*26:01	895	905	QIPFAMQMAY	0.34
B*18:01	1,206	1,216	YEQYIKWPWY	0.34
B*27:05	356	366	KRISNCVADY	0.34
B*44:02	779	789	QEVFAQVKQI	0.34
B*44:03	779	789	QEVFAQVKQI	0.34
B*57:01	49	59	HSTQDLFLPF	0.34
B*57:01	304	314	KSFTVEKGIY	0.34
B*58:01	879	889	AGTITSGWTF	0.34
A*01:01	29	39	TNSFTRGVYY	0.35
A*02:01	947	957	KLQDVVNQNA	0.35
A*02:01	975	985	SVLNDILSRL	0.35
A*02:01	995	1,005	RLITGRLQSL	0.35
A*02:01	1,136	1,146	TVYDPLQPEL	0.35
A*02:02	947	957	KLQDVVNQNA	0.35
A*02:02	1,136	1,146	TVYDPLQPEL	0.35
A*11:01	924	934	ANQFNSAIGK	0.35
A*24:02	320	330	VQPTESIVRF	0.35
A*33:01	637	647	STGSNVFQTR	0.35
B*15:01	464	474	FERDISTEIY	0.35
B*38:01	773	783	EQDKNTQEVF	0.35
B*49:01	987	997	VEAEVQIDRL	0.35
B*53:01	808	818	DPSKPSKRSF	0.35
B*57:01	249	259	LTPGDSSSGW	0.35
C*02:02	1,136	1,146	TVYDPLQPEL	0.35

C*03:02	686	696	SVASQSIIAY	0.35
C*14:02	1,094	1,104	VFVSNGTHWF	0.35
A*23:01	1,100	1,110	THWFVTQRNF	0.36
A*24:02	504	514	GYQPYRVVVL	0.36
A*26:01	1,136	1,146	TVYDPLQPEL	0.36
A*29:02	195	205	KNIDGYFKIY	0.36
A*33:01	35	45	GVYYPDKVFR	0.36
B*07:02	620	630	VPVAIHADQL	0.36
B*18:01	627	637	DQLTPTWRVY	0.36
B*35:03	83	93	VLPFNDGVYF	0.36
B*38:01	77	87	KRFDNPVLPF	0.36
B*57:01	877	887	LLAGTITSGW	0.36
C*16:01	929	939	SAIGKIQDSL	0.36
A*02:05	995	1,005	RLITGRLQSL	0.37
A*02:06	975	985	SVLNDILSRL	0.37
A*03:01	35	45	GVYYPDKVFR	0.37
A*23:01	1,147	1,157	SFKEELDKYF	0.37
A*23:01	1,207	1,217	EQYIKWPWYI	0.37
A*24:02	1,147	1,157	SFKEELDKYF	0.37
A*26:01	1,058	1,068	HGVVFLHVTY	0.37
A*31:01	457	467	RKSNLKPFER	0.37
A*32:01	995	1,005	RLITGRLQSL	0.37
B*07:02	1,052	1,062	FPQSAPHGVV	0.37
B*13:02	893	903	ALQIPFAMQM	0.37
B*27:05	236	246	TRFQTLLALH	0.37
B*49:01	1,181	1,191	KEIDRLNEVA	0.37
C*07:04	575	585	AVRDPQTLEI	0.37
A*01:01	19	29	TTRTQLPPAY	0.38
A*02:06	626	636	ADQLTPTWRV	0.38
A*03:01	1,019	1,029	RASANLAATK	0.38
A*25:01	28	38	YTNSFTRGVY	0.38
A*25:01	442	452	DSKVGGNYNY	0.38
A*26:01	192	202	FVFKNIDGYF	0.38
B*08:01	929	939	SAIGKIQDSL	0.38
B*13:02	761	771	TQLNRALTGI	0.38
B*44:03	987	997	VEAEVQIDRL	0.38
B*58:01	877	887	LLAGTITSGW	0.38

C*03:02	28	38	YTNSFTRGVY	0.38
C*17:01	718	728	FTISVTTEIL	0.38
C*17:03	718	728	FTISVTTEIL	0.38
A*02:05	1,095	1,105	FVSNGTHWFV	0.39
A*23:01	168	178	FEYVSQPFLM	0.39
B*35:01	320	330	VQPTESIVRF	0.39
C*07:02	998	1,008	TGRLQSLQTY	0.39
C*14:02	151	161	SWMESEFRVY	0.39
A*02:06	852	862	AQKFNGLTVL	0.4
A*03:01	454	464	RLFRKSNLKP	0.4
A*11:01	786	796	KQIYKTPPIK	0.4
A*11:01	974	984	SSVLNDILSR	0.4
A*25:01	192	202	FVFKNIDGYF	0.4
A*25:01	1,093	1,103	GVFVSNGTHW	0.4
A*29:02	161	171	SSANNCTFEY	0.4
A*29:02	444	454	KVGGNYNYLY	0.4
B*15:01	893	903	ALQIPFAMQM	0.4
B*44:02	1,205	1,215	KYEQYIKWPW	0.4
B*46:01	828	838	LADAGFIKQY	0.4
C*02:02	929	939	SAIGKIQDSL	0.4
C*03:04	220	230	FSALEPLVDL	0.4
C*15:02	1,136	1,146	TVYDPLQPEL	0.4
A*25:01	249	259	LTPGDSSSGW	0.41
A*29:02	698	708	SLGAENSVAY	0.41
B*35:03	1,139	1,149	DPLQPELDSF	0.41
C*03:02	828	838	LADAGFIKQY	0.41
C*07:01	998	1,008	TGRLQSLQTY	0.41
C*12:02	135	145	FCNDPFLGVY	0.41
C*12:02	929	939	SAIGKIQDSL	0.41
C*14:02	1,066	1,076	TYVPAQEKNF	0.41
C*15:02	1,054	1,064	QSAPHGVVFL	0.41
A*11:01	319	329	RVQPTESIVR	0.42
A*24:02	1,136	1,146	TVYDPLQPEL	0.42
A*25:01	211	221	NLVRDLPQGF	0.42
A*29:02	486	496	FNCYFPLQSY	0.42
A*29:02	747	757	TECSNLLLQY	0.42
A*32:01	877	887	LLAGTITSGW	0.42

A*33:01	673	683	SYQTQTNSPR	0.42
B*40:01	168	178	FEYVSQPFLM	0.42
B*46:01	365	375	YSVLYNSASF	0.42
B*46:01	486	496	FNCYFPLQSY	0.42
A*01:01	686	696	SVASQSIIAY	0.43
A*11:01	637	647	STGSNVFQTR	0.43
A*23:01	504	514	GYQPYRVVVL	0.43
A*23:01	624	634	IHADQLTPTW	0.43
A*23:01	897	907	PFAMQMAYRF	0.43
A*25:01	1,146	1,156	DSFKEELDKY	0.43
A*29:02	864	874	LLTDEMIAQY	0.43
B*15:01	496	506	GFQPTNGVGY	0.43
B*40:02	168	178	FEYVSQPFLM	0.43
B*44:02	1,261	1,271	SEPVLKGVKL	0.43
B*51:01	229	239	LPIGINITRF	0.43
B*58:01	304	314	KSFTVEKGIY	0.43
C*07:02	326	336	IVRFPNITNL	0.43
C*12:02	28	38	YTNSFTRGVY	0.43
A*01:01	371	381	SASFSTFKCY	0.44
A*32:01	879	889	AGTITSGWTF	0.44
A*33:01	568	578	DIADTTDAVR	0.44
A*33:01	676	686	TQTNSPRRAR	0.44
B*07:02	680	690	SPRRARSVAS	0.44
B*15:01	83	93	VLPFNDGVYF	0.44
B*27:05	846	856	ARDLICAQKF	0.44
B*35:03	329	339	FPNITNLCPF	0.44
B*44:03	1,200	1,210	LQELGKYEQY	0.44
B*45:01	772	782	VEQDKNTQEV	0.44
B*45:01	1,180	1,190	QKEIDRLNEV	0.44
B*51:01	560	570	LPFQQFGRDI	0.44
C*01:02	713	723	AIPTNFTISV	0.44
C*02:02	686	696	SVASQSIIAY	0.44
C*03:02	342	352	FNATRFASVY	0.44
A*02:05	852	862	AQKFNGLTVL	0.45
A*11:01	805	815	ILPDPSKPSK	0.45
A*24:02	168	178	FEYVSQPFLM	0.45
A*29:02	895	905	QIPFAMQMAY	0.45

B*07:02	588	598	TPCSFGGVSV	0.45
B*13:02	754	764	LQYGSFCTQL	0.45
B*13:02	835	845	KQYGDCLGDI	0.45
B*15:01	23	33	QLPPAYTNSF	0.45
B*15:01	961	971	TLVKQLSSNF	0.45
B*35:01	506	516	QPYRVVVLSF	0.45
B*44:03	1,261	1,271	SEPVLKGVKL	0.45
B*45:01	154	164	ESEFRVYSSA	0.45
C*03:04	1,054	1,064	QSAPHGVVFL	0.45
C*12:02	686	696	SVASQSIIAY	0.45
C*12:02	828	838	LADAGFIKQY	0.45
C*12:02	1,136	1,146	TVYDPLQPEL	0.45
C*14:02	203	213	IYSKHTPINL	0.45
A*02:02	109	119	TLDSKTQSLL	0.46
A*11:01	301	311	CTLKSFTVEK	0.46
A*26:01	211	221	NLVRDLPQGF	0.46
A*26:01	1,188	1,198	EVAKNLNESL	0.46
A*32:01	266	276	YVGYLQPRTF	0.46
A*33:01	346	356	RFASVYAWNR	0.46
A*68:02	108	118	TTLDSKTQSL	0.46
B*46:01	192	202	FVFKNIDGYF	0.46
C*01:02	1,136	1,146	TVYDPLQPEL	0.46
C*03:04	718	728	FTISVTTEIL	0.46
A*24:02	1,207	1,217	EQYIKWPWYI	0.47
A*26:01	698	708	SLGAENSVAY	0.47
A*33:01	138	148	DPFLGVYYHK	0.47
A*68:02	1,054	1,064	QSAPHGVVFL	0.47
B*07:02	727	737	LPVSMTKTSV	0.47
B*15:01	1,080	1,090	AICHDGKAHF	0.47
B*37:01	987	997	VEAEVQIDRL	0.47
B*51:01	56	66	LPFFSNVTWF	0.47
B*57:01	50	60	STQDLFLPFF	0.47
C*02:02	135	145	FCNDPFLGVY	0.47
C*04:01	109	119	TLDSKTQSLL	0.47
C*04:01	576	586	VRDPQTLEIL	0.47
C*04:43	109	119	TLDSKTQSLL	0.47
C*04:43	576	586	VRDPQTLEIL	0.47

C*06:02	982	992	SRLDKVEAEV	0.47
C*12:02	266	276	YVGYLQPRTF	0.47
A*26:01	19	29	TTRTQLPPAY	0.48
A*29:02	780	790	EVFAQVKQIY	0.48
B*27:05	101	111	IRGWIFGTTL	0.48
B*27:05	453	463	YRLFRKSNLK	0.48
B*27:05	904	914	YRFNGIGVTQ	0.48
B*40:01	167	177	TFEYVSQPFL	0.48
B*44:02	987	997	VEAEVQIDRL	0.48
B*44:03	1,206	1,216	YEQYIKWPWY	0.48
B*46:01	342	352	FNATRFASVY	0.48
B*49:01	470	480	TEIYQAGSTP	0.48
C*14:02	1,136	1,146	TVYDPLQPEL	0.48
C*17:01	929	939	SAIGKIQDSL	0.48
C*17:03	929	939	SAIGKIQDSL	0.48
A*23:01	77	87	KRFDNPVLPF	0.49
B*35:03	425	435	LPDDFTGCVI	0.49
B*44:02	1,200	1,210	LQELGKYEQY	0.49
A*01:01	698	708	SLGAENSVAY	0.5
A*02:05	386	396	KLNDLCFTNV	0.5
A*24:02	1,205	1,215	KYEQYIKWPW	0.5
A*25:01	950	960	DVVNQNAQAL	0.5
A*29:02	371	381	SASFSTFKCY	0.5
A*33:01	264	274	AYYVGYLQPR	0.5
B*15:01	773	783	EQDKNTQEVF	0.5
B*15:03	998	1,008	TGRLQSLQTY	0.5
B*27:05	19	29	TTRTQLPPAY	0.5
B*44:03	988	998	EAEVQIDRLI	0.5
B*49:01	1,201	1,211	QELGKYEQYI	0.5
B*58:01	1,203	1,213	LGKYEQYIKW	0.5
C*03:02	634	644	RVYSTGSNVF	0.5
C*04:01	108	118	TTLDSKTQSL	0.5
C*04:43	108	118	TTLDSKTQSL	0.5
C*17:01	220	230	FSALEPLVDL	0.5
C*17:03	220	230	FSALEPLVDL	0.5
A*23:01	506	516	QPYRVVVLSF	0.51
A*29:02	135	145	FCNDPFLGVY	0.51

B*15:03	320	330	VQPTESIVRF	0.51
B*37:01	772	782	VEQDKNTQEV	0.51
B*40:02	1,015	1,025	AAEIRASANL	0.51
B*46:01	1,136	1,146	TVYDPLQPEL	0.51
C*07:04	77	87	KRFDNPVLPF	0.51
C*08:01	108	118	TTLDSKTQSL	0.51
C*14:02	267	277	VGYLQPRTFL	0.51
A*02:02	108	118	TTLDSKTQSL	0.52
A*02:06	386	396	KLNDLCFTNV	0.52
A*11:01	686	696	SVASQSIIAY	0.52
A*23:01	1,205	1,215	KYEQYIKWPW	0.52
A*68:02	975	985	SVLNDILSRL	0.52
B*44:02	1,206	1,216	YEQYIKWPWY	0.52
B*44:03	1,205	1,215	KYEQYIKWPW	0.52
B*46:01	651	661	IGAEHVNNSY	0.52
B*46:01	929	939	SAIGKIQDSL	0.52
B*57:01	239	249	QTLLALHRSY	0.52
B*58:01	97	107	KSNIIRGWIF	0.52
C*14:02	504	514	GYQPYRVVVL	0.52
A*02:02	1,047	1,057	YHLMSFPQSA	0.53
A*11:01	269	279	YLQPRTFLLK	0.53
A*24:02	1,100	1,110	THWFVTQRNF	0.53
B*08:01	995	1,005	RLITGRLQSL	0.53
B*13:02	995	1,005	RLITGRLQSL	0.53
B*15:03	893	903	ALQIPFAMQM	0.53
B*35:01	828	838	LADAGFIKQY	0.53
A*01:01	442	452	DSKVGGNYNY	0.54
A*02:06	995	1,005	RLITGRLQSL	0.54
A*03:01	319	329	RVQPTESIVR	0.54
A*11:01	549	559	TGVLTESNKK	0.54
A*29:02	270	280	LQPRTFLLKY	0.54
B*13:02	1,004	1,014	LQTYVTQQLI	0.54
B*44:02	988	998	EAEVQIDRLI	0.54
C*07:01	814	824	KRSFIEDLLF	0.54
C*16:01	28	38	YTNSFTRGVY	0.54
C*16:01	686	696	SVASQSIIAY	0.54
C*17:01	108	118	TTLDSKTQSL	0.54

C*17:03	108	118	TTLDSKTQSL	0.54
A*25:01	961	971	TLVKQLSSNF	0.55
A*25:01	1,058	1,068	HGVVFLHVTY	0.55
A*68:01	974	984	SSVLNDILSR	0.55
B*49:01	1,016	1,026	AEIRASANLA	0.55
B*51:01	224	234	EPLVDLPIGI	0.55
B*53:01	1,052	1,062	FPQSAPHGVV	0.55
C*07:04	108	118	TTLDSKTQSL	0.55
C*07:04	109	119	TLDSKTQSLL	0.55
C*12:03	828	838	LADAGFIKQY	0.55
A*01:01	239	249	QTLLALHRSY	0.56
A*11:01	777	787	NTQEVFAQVK	0.56
A*11:01	816	826	SFIEDLLFNK	0.56
A*25:01	55	65	FLPFFSNVTW	0.56
A*25:01	266	276	YVGYLQPRTF	0.56
A*68:02	61	71	NVTWFHAIHV	0.56
B*15:01	864	874	LLTDEMIAQY	0.56
B*35:01	698	708	SLGAENSVAY	0.56
B*35:01	808	818	DPSKPSKRSF	0.56
B*40:02	1,181	1,191	KEIDRLNEVA	0.56
A*01:01	1,200	1,210	LQELGKYEQY	0.57
A*02:02	1,095	1,105	FVSNGTHWFV	0.57
A*02:06	1,095	1,105	FVSNGTHWFV	0.57
A*31:01	394	404	NVYADSFVIR	0.57
A*32:01	55	65	FLPFFSNVTW	0.57
B*40:02	1,180	1,190	QKEIDRLNEV	0.57
B*44:02	1,201	1,211	QELGKYEQYI	0.57
B*49:01	168	178	FEYVSQPFLM	0.57
B*53:01	84	94	LPFNDGVYFA	0.57
C*01:02	207	217	HTPINLVRDL	0.57
C*02:02	634	644	RVYSTGSNVF	0.57
C*12:03	28	38	YTNSFTRGVY	0.57
C*12:03	1,136	1,146	TVYDPLQPEL	0.57
B*39:01	1,087	1,097	AHFPREGVFV	0.58
C*04:01	1,255	1,265	KFDEDDSEPV	0.58
C*04:43	1,255	1,265	KFDEDDSEPV	0.58
A*01:01	464	474	FERDISTEIY	0.59

A*23:01	1,136	1,146	TVYDPLQPEL	0.59
A*29:02	239	249	QTLLALHRSY	0.59
A*33:01	896	906	IPFAMQMAYR	0.59
B*38:01	1,157	1,167	KNHTSPDVDL	0.59
B*46:01	23	33	QLPPAYTNSF	0.59
B*46:01	371	381	SASFSTFKCY	0.59
B*53:01	895	905	QIPFAMQMAY	0.59
C*01:02	929	939	SAIGKIQDSL	0.59
C*07:01	1,038	1,048	KRVDFCGKGY	0.59
C*15:02	929	939	SAIGKIQDSL	0.59
A*02:01	109	119	TLDSKTQSLL	0.6
A*24:02	378	388	KCYGVSPTKL	0.6
B*15:01	34	44	RGVYYPDKVF	0.6
B*44:03	1,201	1,211	QELGKYEQYI	0.6
C*01:02	83	93	VLPFNDGVYF	0.6
C*07:02	1,066	1,076	TYVPAQEKNF	0.6
C*16:01	1,086	1,096	KAHFPREGVF	0.6
A*24:02	624	634	IHADQLTPTW	0.61
A*26:01	23	33	QLPPAYTNSF	0.61
A*68:01	676	686	TQTNSPRRAR	0.61
B*08:01	108	118	TTLDSKTQSL	0.61
B*35:03	320	330	VQPTESIVRF	0.61
B*40:02	1,194	1,204	NESLIDLQEL	0.61
C*02:02	192	202	FVFKNIDGYF	0.61
C*17:01	170	180	YVSQPFLMDL	0.61
C*17:01	1,054	1,064	QSAPHGVVFL	0.61
C*17:03	170	180	YVSQPFLMDL	0.61
C*17:03	1,054	1,064	QSAPHGVVFL	0.61
A*02:02	1,191	1,201	KNLNESLIDL	0.62
A*02:06	1,128	1,138	VVIGIVNNTV	0.62
A*03:01	826	836	VTLADAGFIK	0.62
A*11:01	291	301	CALDPLSETK	0.62
A*23:01	420	430	DYNYKLPDDF	0.62
A*24:02	897	907	PFAMQMAYRF	0.62
A*25:01	929	939	SAIGKIQDSL	0.62
A*31:01	673	683	SYQTQTNSPR	0.62
A*31:01	1,005	1,015	QTYVTQQLIR	0.62

B*15:03	1,200	1,210	LQELGKYEQY	0.62
B*37:01	1,256	1,266	FDEDDSEPVL	0.62
B*38:01	1,086	1,096	KAHFPREGVF	0.62
B*49:01	280	290	NENGTITDAV	0.62
B*51:01	55	65	FLPFFSNVTW	0.62
C*03:02	929	939	SAIGKIQDSL	0.62
C*03:02	1,136	1,146	TVYDPLQPEL	0.62
C*06:02	576	586	VRDPQTLEIL	0.62
A*02:02	515	525	FELLHAPATV	0.63
A*25:01	23	33	QLPPAYTNSF	0.63
A*32:01	46	56	SVLHSTQDLF	0.63
B*39:01	953	963	NQNAQALNTL	0.63
C*03:02	365	375	YSVLYNSASF	0.63
A*01:01	747	757	TECSNLLLQY	0.64
A*02:01	108	118	TTLDSKTQSL	0.64
A*03:01	104	114	WIFGTTLDSK	0.64
B*07:02	1,056	1,066	APHGVVFLHV	0.64
B*35:01	360	370	NCVADYSVLY	0.64
B*40:02	214	224	RDLPQGFSAL	0.64
B*45:01	280	290	NENGTITDAV	0.64
B*58:01	50	60	STQDLFLPFF	0.64
A*01:01	387	397	LNDLCFTNVY	0.65
A*02:02	416	426	GKIADYNYKL	0.65
A*02:05	1,128	1,138	VVIGIVNNTV	0.65
A*03:01	301	311	CTLKSFTVEK	0.65
A*24:02	77	87	KRFDNPVLPF	0.65
A*25:01	877	887	LLAGTITSGW	0.65
B*44:02	1,015	1,025	AAEIRASANL	0.65
B*53:01	38	48	YPDKVFRSSV	0.65
B*57:01	1,086	1,096	KAHFPREGVF	0.65
A*02:01	1,191	1,201	KNLNESLIDL	0.66
A*24:02	420	430	DYNYKLPDDF	0.66
A*26:01	627	637	DQLTPTWRVY	0.66
A*29:02	488	498	CYFPLQSYGF	0.66
A*33:01	558	568	KFLPFQQFGR	0.66
B*08:01	109	119	TLDSKTQSLL	0.66
B*15:01	1,053	1,063	PQSAPHGVVF	0.66

B*15:03	1,086	1,096	KAHFPREGVF	0.66
B*37:01	1,040	1,050	VDFCGKGYHL	0.66
B*40:01	772	782	VEQDKNTQEV	0.66
B*40:01	779	789	QEVFAQVKQI	0.66
B*49:01	1,256	1,266	FDEDDSEPVL	0.66
B*53:01	620	630	VPVAIHADQL	0.66
C*16:01	29	39	TNSFTRGVYY	0.66
A*26:01	29	39	TNSFTRGVYY	0.67
A*68:01	724	734	TEILPVSMTK	0.67
B*18:01	168	178	FEYVSQPFLM	0.67
B*35:01	896	906	IPFAMQMAYR	0.67
B*35:03	929	939	SAIGKIQDSL	0.67
B*40:02	167	177	TFEYVSQPFL	0.67
B*44:02	1,194	1,204	NESLIDLQEL	0.67
B*45:01	989	999	AEVQIDRLIT	0.67
C*02:02	29	39	TNSFTRGVYY	0.67
C*03:02	486	496	FNCYFPLQSY	0.67
C*03:02	651	661	IGAEHVNNSY	0.67
C*12:03	266	276	YVGYLQPRTF	0.67
C*15:02	967	977	SSNFGAISSV	0.67
A*23:01	447	457	GNYNYLYRLF	0.68
A*29:02	1,058	1,068	HGVVFLHVTY	0.68
B*40:02	779	789	QEVFAQVKQI	0.68
B*40:02	1,256	1,266	FDEDDSEPVL	0.68
B*44:03	1,015	1,025	AAEIRASANL	0.68
B*53:01	625	635	HADQLTPTWR	0.68
B*58:01	625	635	HADQLTPTWR	0.68
C*01:02	108	118	TTLDSKTQSL	0.68
C*02:02	1,086	1,096	KAHFPREGVF	0.68
C*07:02	159	169	VYSSANNCTF	0.68
C*14:02	496	506	GFQPTNGVGY	0.68
A*24:02	506	516	QPYRVVVLSF	0.69
A*29:02	19	29	TTRTQLPPAY	0.69
A*68:02	224	234	EPLVDLPIGI	0.69
B*46:01	1,086	1,096	KAHFPREGVF	0.69
C*07:02	368	378	LYNSASFSTF	0.69
A*01:01	151	161	SWMESEFRVY	0.7

A*02:01	1,003	1,013	SLQTYVTQQL	0.7
A*24:02	83	93	VLPFNDGVYF	0.7
B*07:02	679	689	NSPRRARSVA	0.7
B*15:01	754	764	LQYGSFCTQL	0.7
B*35:01	383	393	SPTKLNDLCF	0.7
A*31:01	12	22	SSQCVNLTTR	0.71
A*68:01	369	379	YNSASFSTFK	0.71
B*07:02	229	239	LPIGINITRF	0.71
B*07:02	808	818	DPSKPSKRSF	0.71
B*07:02	1,051	1,061	SFPQSAPHGV	0.71
B*08:01	1,261	1,271	SEPVLKGVKL	0.71
B*38:01	1,136	1,146	TVYDPLQPEL	0.71
B*44:03	1,194	1,204	NESLIDLQEL	0.71
B*51:01	320	330	VQPTESIVRF	0.71
B*53:01	791	801	TPPIKDFGGF	0.71
C*02:02	342	352	FNATRFASVY	0.71
C*05:01	226	236	LVDLPIGINI	0.71
C*15:02	204	214	YSKHTPINLV	0.71
A*01:01	108	118	TTLDSKTQSL	0.72
A*02:05	326	336	IVRFPNITNL	0.72
A*02:06	893	903	ALQIPFAMQM	0.72
A*24:02	447	457	GNYNYLYRLF	0.72
A*29:02	627	637	DQLTPTWRVY	0.72
A*29:02	908	918	GIGVTQNVLY	0.72
B*15:01	627	637	DQLTPTWRVY	0.72
B*58:01	55	65	FLPFFSNVTW	0.72
A*02:06	947	957	KLQDVVNQNA	0.73
A*29:02	1,263	1,273	PVLKGVKLHY	0.73
B*15:01	28	38	YTNSFTRGVY	0.73
B*18:01	867	877	DEMIAQYTSA	0.73
B*35:03	588	598	TPCSFGGVSV	0.73
B*44:02	427	437	DDFTGCVIAW	0.73
B*44:02	1,180	1,190	QKEIDRLNEV	0.73
B*44:03	427	437	DDFTGCVIAW	0.73
B*46:01	718	728	FTISVTTEIL	0.73
C*12:02	634	644	RVYSTGSNVF	0.73
C*16:01	828	838	LADAGFIKQY	0.73

A*02:05	718	728	FTISVTTEIL	0.74
A*68:02	204	214	YSKHTPINLV	0.74
B*15:01	151	161	SWMESEFRVY	0.74
B*18:01	1,200	1,210	LQELGKYEQY	0.74
B*35:03	55	65	FLPFFSNVTW	0.74
B*35:03	1,261	1,271	SEPVLKGVKL	0.74
B*38:01	953	963	NQNAQALNTL	0.74
C*08:02	772	782	VEQDKNTQEV	0.74
A*02:01	1,095	1,105	FVSNGTHWFV	0.75
A*68:02	121	131	NNATNVVIKV	0.75
B*07:02	329	339	FPNITNLCPF	0.75
B*15:01	266	276	YVGYLQPRTF	0.75
B*27:05	508	518	YRVVVLSFEL	0.75
B*35:01	780	790	EVFAQVKQIY	0.75
C*05:01	772	782	VEQDKNTQEV	0.75
C*16:01	1,054	1,064	QSAPHGVVFL	0.75
A*02:01	999	1,009	GRLQSLQTYV	0.76
A*03:01	141	151	LGVYYHKNNK	0.76
A*25:01	718	728	FTISVTTEIL	0.76
A*25:01	868	878	EMIAQYTSAL	0.76
A*26:01	166	176	CTFEYVSQPF	0.76
A*32:01	50	60	STQDLFLPFF	0.76
B*27:05	327	337	VRFPNITNLC	0.76
C*14:02	758	768	SFCTQLNRAL	0.76
A*26:01	950	960	DVVNQNAQAL	0.77
A*26:01	961	971	TLVKQLSSNF	0.77
A*32:01	444	454	KVGGNYNYLY	0.77
B*13:02	1,003	1,013	SLQTYVTQQL	0.77
B*18:01	1,150	1,160	EELDKYFKNH	0.77
B*35:01	135	145	FCNDPFLGVY	0.77
C*14:02	168	178	FEYVSQPFLM	0.77
C*14:02	1,147	1,157	SFKEELDKYF	0.77
C*15:02	108	118	TTLDSKTQSL	0.77
A*25:01	344	354	ATRFASVYAW	0.78
A*26:01	864	874	LLTDEMIAQY	0.78
A*29:02	256	266	SGWTAGAAAY	0.78
B*13:02	1,105	1,115	TQRNFYEPQI	0.78

B*15:03	270	280	LQPRTFLLKY	0.78
B*49:01	339	349	GEVFNATRFA	0.78
C*01:02	1,055	1,065	SAPHGVVFLH	0.78
C*08:01	929	939	SAIGKIQDSL	0.78
C*14:02	488	498	CYFPLQSYGF	0.78
C*17:01	326	336	IVRFPNITNL	0.78
C*17:03	326	336	IVRFPNITNL	0.78
A*01:01	1,038	1,048	KRVDFCGKGY	0.79
A*11:01	310	320	KGIYQTSNFR	0.79
A*33:01	1,005	1,015	QTYVTQQLIR	0.79
B*53:01	23	33	QLPPAYTNSF	0.79
B*53:01	294	304	DPLSETKCTL	0.79
B*58:01	1,086	1,096	KAHFPREGVF	0.79
C*15:02	575	585	AVRDPQTLEI	0.79
A*25:01	895	905	QIPFAMQMAY	0.8
A*31:01	193	203	VFKNIDGYFK	0.8
A*31:01	974	984	SSVLNDILSR	0.8
A*68:02	306	316	FTVEKGIYQT	0.8
B*18:01	297	307	SETKCTLKSF	0.8
B*27:05	576	586	VRDPQTLEIL	0.8
B*40:02	772	782	VEQDKNTQEV	0.8
B*44:03	1,180	1,190	QKEIDRLNEV	0.8
B*45:01	653	663	AEHVNNSYEC	0.8
B*49:01	167	177	TFEYVSQPFL	0.8
B*51:01	83	93	VLPFNDGVYF	0.8
B*53:01	224	234	EPLVDLPIGI	0.8
B*57:01	55	65	FLPFFSNVTW	0.8
B*57:01	126	136	VVIKVCEFQF	0.8
C*02:02	718	728	FTISVTTEIL	0.8
A*29:02	464	474	FERDISTEIY	0.81
A*68:02	711	721	SIAIPTNFTI	0.81
B*07:02	683	693	RARSVASQSI	0.81
B*39:01	317	327	NFRVQPTESI	0.81
B*53:01	877	887	LLAGTITSGW	0.81
C*01:02	109	119	TLDSKTQSLL	0.81
C*03:02	1,086	1,096	KAHFPREGVF	0.81
C*07:02	464	474	FERDISTEIY	0.81

C*12:03	29	39	TNSFTRGVYY	0.81
A*03:01	845	855	AARDLICAQK	0.82
A*32:01	814	824	KRSFIEDLLF	0.82
A*68:02	902	912	MAYRFNGIGV	0.82
B*14:02	326	336	IVRFPNITNL	0.82
B*35:03	860	870	VLPPLLTDEM	0.82
B*45:01	279	289	YNENGTITDA	0.82
C*12:02	486	496	FNCYFPLQSY	0.82
A*25:01	19	29	TTRTQLPPAY	0.83
A*29:02	191	201	EFVFKNIDGY	0.83
B*40:02	515	525	FELLHAPATV	0.83
C*01:02	477	487	STPCNGVEGF	0.83
C*06:02	318	328	FRVQPTESIV	0.83
A*02:02	976	986	VLNDILSRLD	0.84
A*02:06	326	336	IVRFPNITNL	0.84
A*29:02	168	178	FEYVSQPFLM	0.84
B*15:01	651	661	IGAEHVNNSY	0.84
B*35:01	23	33	QLPPAYTNSF	0.84
B*35:03	727	737	LPVSMTKTSV	0.84
C*04:01	38	48	YPDKVFRSSV	0.84
C*04:43	38	48	YPDKVFRSSV	0.84
A*11:01	1,005	1,015	QTYVTQQLIR	0.85
A*26:01	828	838	LADAGFIKQY	0.85
A*29:02	499	509	PTNGVGYQPY	0.85
A*29:02	828	838	LADAGFIKQY	0.85
B*08:01	808	818	DPSKPSKRSF	0.85
B*35:01	651	661	IGAEHVNNSY	0.85
C*03:02	29	39	TNSFTRGVYY	0.85
C*07:01	19	29	TTRTQLPPAY	0.85
C*12:02	342	352	FNATRFASVY	0.85
A*01:01	50	60	STQDLFLPFF	0.86
A*02:05	929	939	SAIGKIQDSL	0.86
A*02:06	515	525	FELLHAPATV	0.86
A*02:06	817	827	FIEDLLFNKV	0.86
A*32:01	83	93	VLPFNDGVYF	0.86
A*68:02	950	960	DVVNQNAQAL	0.86
B*38:01	1,256	1,266	FDEDDSEPVL	0.86

B*40:01	1,180	1,190	QKEIDRLNEV	0.86
B*44:02	773	783	EQDKNTQEVF	0.86
B*46:01	83	93	VLPFNDGVYF	0.86
B*49:01	1,261	1,271	SEPVLKGVKL	0.86
B*58:01	634	644	RVYSTGSNVF	0.86
C*06:02	327	337	VRFPNITNLC	0.86
C*17:01	975	985	SVLNDILSRL	0.86
C*17:03	975	985	SVLNDILSRL	0.86
A*01:01	195	205	KNIDGYFKIY	0.87
A*02:02	999	1,009	GRLQSLQTYV	0.87
A*02:05	1,003	1,013	SLQTYVTQQL	0.87
A*23:01	83	93	VLPFNDGVYF	0.87
A*26:01	477	487	STPCNGVEGF	0.87
B*07:02	326	336	IVRFPNITNL	0.87
B*07:02	713	723	AIPTNFTISV	0.87
B*14:02	1,184	1,194	DRLNEVAKNL	0.87
B*15:03	627	637	DQLTPTWRVY	0.87
B*35:01	84	94	LPFNDGVYFA	0.87
B*35:01	1,052	1,062	FPQSAPHGVV	0.87
B*38:01	846	856	ARDLICAQKF	0.87
C*03:02	371	381	SASFSTFKCY	0.87
C*14:02	268	278	GYLQPRTFLL	0.87
A*23:01	56	66	LPFFSNVTWF	0.88
A*24:02	185	195	NFKNLREFVF	0.88
A*26:01	239	249	QTLLALHRSY	0.88
A*29:02	1,146	1,156	DSFKEELDKY	0.88
B*15:01	19	29	TTRTQLPPAY	0.88
B*15:03	754	764	LQYGSFCTQL	0.88
B*40:01	852	862	AQKFNGLTVL	0.88
B*51:01	294	304	DPLSETKCTL	0.88
B*53:01	583	593	EILDITPCSF	0.88
C*07:01	788	798	IYKTPPIKDF	0.88
C*08:02	773	783	EQDKNTQEVF	0.88
C*08:02	1,256	1,266	FDEDDSEPVL	0.88
A*02:05	817	827	FIEDLLFNKV	0.89
A*11:01	548	558	GTGVLTESNK	0.89
A*25:01	808	818	DPSKPSKRSF	0.89

A*26:01	868	878	EMIAQYTSAL	0.89
A*31:01	986	996	KVEAEVQIDR	0.89
B*49:01	659	669	SYECDIPIGA	0.89
B*51:01	425	435	LPDDFTGCVI	0.89
C*07:01	846	856	ARDLICAQKF	0.89
C*12:03	1,086	1,096	KAHFPREGVF	0.89
C*16:01	651	661	IGAEHVNNSY	0.89
C*17:01	575	585	AVRDPQTLEI	0.89
C*17:03	575	585	AVRDPQTLEI	0.89
A*02:02	47	57	VLHSTQDLFL	0.9
A*02:05	947	957	KLQDVVNQNA	0.9
A*02:06	1,047	1,057	YHLMSFPQSA	0.9
A*03:01	369	379	YNSASFSTFK	0.9
A*31:01	1,098	1,108	NGTHWFVTQR	0.9
B*08:01	38	48	YPDKVFRSSV	0.9
B*15:03	556	566	NKKFLPFQQF	0.9
B*46:01	29	39	TNSFTRGVYY	0.9
C*03:04	108	118	TTLDSKTQSL	0.9
C*07:02	203	213	IYSKHTPINL	0.9
C*08:01	220	230	FSALEPLVDL	0.9
C*17:01	109	119	TLDSKTQSLL	0.9
C*17:03	109	119	TLDSKTQSLL	0.9
A*02:01	982	992	SRLDKVEAEV	0.91
A*02:06	713	723	AIPTNFTISV	0.91
A*23:01	185	195	NFKNLREFVF	0.91
A*25:01	166	176	CTFEYVSQPF	0.91
A*26:01	266	276	YVGYLQPRTF	0.91
A*68:01	780	790	EVFAQVKQIY	0.91
A*68:02	868	878	EMIAQYTSAL	0.91
B*40:02	297	307	SETKCTLKSF	0.91
B*45:01	617	627	CTEVPVAIHA	0.91
B*45:01	779	789	QEVFAQVKQI	0.91
B*51:01	588	598	TPCSFGGVSV	0.91
C*12:03	686	696	SVASQSIIAY	0.91
C*15:02	718	728	FTISVTTEIL	0.91
A*02:02	268	278	GYLQPRTFLL	0.92
A*02:05	893	903	ALQIPFAMQM	0.92

A*11:01	938	948	LSSTASALGK	0.92
B*15:01	1,129	1,139	VIGIVNNTVY	0.92
B*18:01	1,194	1,204	NESLIDLQEL	0.92
B*46:01	864	874	LLTDEMIAQY	0.92
B*46:01	961	971	TLVKQLSSNF	0.92
C*07:01	356	366	KRISNCVADY	0.92
C*12:02	651	661	IGAEHVNNSY	0.92
A*01:01	444	454	KVGGNYNYLY	0.93
A*26:01	486	496	FNCYFPLQSY	0.93
A*33:01	69	79	HVSGTNGTKR	0.93
A*68:01	674	684	YQTQTNSPRR	0.93
B*15:03	698	708	SLGAENSVAY	0.93
B*39:01	508	518	YRVVVLSFEL	0.93
B*39:01	1,256	1,266	FDEDDSEPVL	0.93
B*44:03	1,150	1,160	EELDKYFKNH	0.93
C*02:02	371	381	SASFSTFKCY	0.93
C*07:02	1,038	1,048	KRVDFCGKGY	0.93
C*12:02	371	381	SASFSTFKCY	0.93
A*02:02	817	827	FIEDLLFNKV	0.94
A*03:01	348	358	ASVYAWNRKR	0.94
A*29:02	1,094	1,104	VFVSNGTHWF	0.94
A*32:01	77	87	KRFDNPVLPF	0.94
B*07:02	56	66	LPFFSNVTWF	0.94
B*46:01	1,129	1,139	VIGIVNNTVY	0.94
C*07:02	19	29	TTRTQLPPAY	0.94
A*02:05	713	723	AIPTNFTISV	0.95
A*02:05	754	764	LQYGSFCTQL	0.95
A*23:01	378	388	KCYGVSPTKL	0.95
A*25:01	229	239	LPIGINITRF	0.95
A*68:02	53	63	DLFLPFFSNV	0.95
A*68:02	326	336	IVRFPNITNL	0.95
B*15:03	356	366	KRISNCVADY	0.95
B*40:01	515	525	FELLHAPATV	0.95
B*40:01	1,201	1,211	QELGKYEQYI	0.95
C*07:02	144	154	YYHKNNKSWM	0.95
C*12:02	1,086	1,096	KAHFPREGVF	0.95
A*03:01	686	696	SVASQSIIAY	0.96

A*11:01	845	855	AARDLICAQK	0.96
A*32:01	126	136	VVIKVCEFQF	0.96
B*07:02	383	393	SPTKLNDLCF	0.96
B*13:02	386	396	KLNDLCFTNV	0.96
B*49:01	323	333	TESIVRFPNI	0.96
B*51:01	1,056	1,066	APHGVVFLHV	0.96
B*57:01	634	644	RVYSTGSNVF	0.96
C*07:02	356	366	KRISNCVADY	0.96
C*07:04	1,137	1,147	VYDPLQPELD	0.96
A*25:01	50	60	STQDLFLPFF	0.97
A*29:02	136	146	CNDPFLGVYY	0.97
A*33:01	237	247	RFQTLLALHR	0.97
B*13:02	1,207	1,217	EQYIKWPWYI	0.97
B*15:01	46	56	SVLHSTQDLF	0.97
B*46:01	19	29	TTRTQLPPAY	0.97
A*02:05	567	577	RDIADTTDAV	0.98
A*25:01	975	985	SVLNDILSRL	0.98
B*37:01	573	583	TDAVRDPQTL	0.98
B*38:01	575	585	AVRDPQTLEI	0.98
B*45:01	514	524	SFELLHAPAT	0.98
B*51:01	711	721	SIAIPTNFTI	0.98
A*26:01	50	60	STQDLFLPFF	0.99
A*29:02	368	378	LYNSASFSTF	0.99
B*35:03	1,136	1,146	TVYDPLQPEL	0.99
B*53:01	425	435	LPDDFTGCVI	0.99

Table B. LPR < 1 values for all alleles and associated epitope sequences for the ORF1ab polyprotein. LPR values are ranked from lowest (highest affinity) to highest (lowest affinity)

Allele	Start position	End position	Epitope sequence	Lowest Percentile Rank
A*01:01	1, 325	1, 335	VPTDNYITTY	0. 01
A*01:01	1, 642	1, 652	TTDPSFLGRY	0. 01
A*01:01	3, 441	3, 451	AGTDLEGNFY	0. 01
A*01:01	4, 009	4, 019	MADQAMTQMY	0. 01
A*01:01	4, 087	4, 097	NTCDGTTFTY	0. 01

A*01:01	4, 167	4, 177	ACTDDNALAY	0. 01
A*01:01	4, 168	4, 178	CTDDNALAYY	0. 01
A*01:01	5, 469	5, 479	ATEETFKLSY	0. 01
A*01:01	5, 861	5, 871	TVDSSQGSEY	0. 01
A*03:01	1, 181	1, 191	AVFDKNLYDK	0. 01
A*03:01	5, 538	5, 548	VVYRGTTTYK	0. 01
A*11:01	1, 181	1, 191	AVFDKNLYDK	0. 01
A*11:01	2, 604	2, 614	SSTFNVPMEK	0. 01
A*23:01	1, 540	1, 550	VYYTSNPTTF	0. 01
A*24:02	1, 540	1, 550	VYYTSNPTTF	0. 01
A*25:01	2, 310	2, 320	ETIQITISSF	0. 01
A*26:01	2, 310	2, 320	ETIQITISSF	0. 01
A*32:01	2, 594	2, 604	KMFDAYVNTF	0. 01
A*68:01	2, 055	2, 065	EVVENPTIQK	0. 01
B*15:01	1, 817	1, 827	AQYELKHGTF	0. 01
B*15:01	5, 390	5, 400	TQLYLGGMSY	0. 01
B*18:01	5, 589	5, 599	DEFSSNVANY	0. 01
B*27:05	6, 713	6, 723	KRFKESPFEL	0. 01
B*38:01	1, 549	1, 559	FHLDGEVITF	0. 01
B*44:02	5, 272	5, 282	QEYADVFHLY	0. 01
B*44:02	6, 007	6, 017	EEAIRHVRAW	0. 01
B*44:03	938	948	EEEFEPSTQY	0. 01
B*44:03	1, 895	1, 905	TEIDPKLDNY	0. 01
B*44:03	5, 272	5, 282	QEYADVFHLY	0. 01
B*49:01	1, 862	1, 872	SEYKGPITDV	0. 01
B*53:01	6, 799	6, 809	YPKLQSSQAW	0. 01
B*57:01	1, 510	1, 520	ISLAGSYKDW	0. 01
B*57:01	3, 578	3, 588	KRTIKGTHHW	0. 01
B*57:01	4, 897	4, 907	KSAGFPFNKW	0. 01
B*57:01	6, 970	6, 980	VAIKITEHSW	0. 01
B*58:01	3, 578	3, 588	KRTIKGTHHW	0. 01
B*58:01	4, 897	4, 907	KSAGFPFNKW	0. 01
B*58:01	6, 970	6, 980	VAIKITEHSW	0. 01
A*01:01	3, 246	3, 256	FSNSGSDVLY	0. 02
A*01:01	5, 134	5, 144	VDTDFVNEFY	0. 02
A*03:01	2, 604	2, 614	SSTFNVPMEK	0. 02
A*11:01	282	292	KTIQPRVEKK	0. 02
A*11:01	1, 879	1, 889	TTTIKPVTYK	0. 02
A*11:01	2, 196	2, 206	KASMPTTIAK	0. 02
A*23:01	4, 465	4, 475	SYFVVKRHTF	0. 02
A*24:02	3, 816	3, 826	YDYLVSTQEF	0. 02
A*25:01	670	680	EIKESVQTFF	0. 02
A*25:01	2, 473	2, 483	EVARDLSLQF	0. 02

A*25:01	4, 537	4, 547	DTLKEILVTY	0. 02
A*25:01	6, 570	6, 580	ETICAPLTVF	0. 02
A*32:01	4, 678	4, 688	KLFDRYFKYW	0. 02
A*68:01	5, 880	5, 890	ETAHSCNVNR	0. 02
B*15:01	5, 537	5, 547	AVVYRGTTTY	0. 02
B*15:03	1, 817	1, 827	AQYELKHGTF	0. 02
B*15:03	5, 390	5, 400	TQLYLGGMSY	0. 02
B*18:01	938	948	EEEFEPSTQY	0. 02
B*35:01	1, 325	1, 335	VPTDNYITTY	0. 02
B*35:01	6, 726	6, 736	IPMDSTVKNY	0. 02
B*38:01	4, 478	4, 488	QHEETIYNLL	0. 02
B*38:01	5, 288	5, 298	LHDELTGHML	0. 02
B*40:01	5, 775	5, 785	AEIVDTVSAL	0. 02
B*44:02	669	679	KEIKESVQTF	0. 02
B*44:02	938	948	EEEFEPSTQY	0. 02
B*44:02	1, 895	1, 905	TEIDPKLDNY	0. 02
B*44:02	4, 563	4, 573	VENPDILRVY	0. 02
B*44:03	669	679	KEIKESVQTF	0. 02
B*44:03	4, 563	4, 573	VENPDILRVY	0. 02
B*46:01	3, 371	3, 381	FVRIQPGQTF	0. 02
B*51:01	5, 225	5, 235	YLPYPDPSRI	0. 02
B*53:01	152	162	DPYEDFQENW	0. 02
B*53:01	891	901	TPLGIDLDEW	0. 02
B*53:01	6, 168	6, 178	NPFMIDVQQW	0. 02
B*53:01	6, 506	6, 516	LPVNVAFELW	0. 02
B*57:01	5, 487	5, 497	LSDRELHLSW	0. 02
B*58:01	1, 510	1, 520	ISLAGSYKDW	0. 02
A*01:01	1, 896	1, 906	EIDPKLDNYY	0. 03
A*01:01	3, 313	3, 323	TSEDMLNPNY	0. 03
A*01:01	3, 950	3, 960	ASEFSSLPSY	0. 03
A*01:01	5, 777	5, 787	IVDTVSALVY	0. 03
A*03:01	282	292	KTIQPRVEKK	0. 03
A*11:01	5, 538	5, 548	VVYRGTTTYK	0. 03
A*25:01	1, 418	1, 428	EGVVDYGARF	0. 03
A*25:01	3, 360	3, 370	DTANPKTPKY	0. 03
A*25:01	5, 005	5, 015	DVENPHLMGW	0. 03
A*25:01	7, 036	7, 046	NTNPIQLSSY	0. 03
A*26:01	2, 137	2, 147	SVPWDTIANY	0. 03
A*26:01	2, 473	2, 483	EVARDLSLQF	0. 03
A*26:01	3, 360	3, 370	DTANPKTPKY	0. 03
A*26:01	4, 537	4, 547	DTLKEILVTY	0. 03
A*29:02	5, 272	5, 282	QEYADVFHLY	0. 03
A*29:02	5, 619	5, 629	HFAIGLALYY	0. 03

A*68:01	1,508	1,518	ETISLAGSYK	0.03
A*68:01	1,641	1,651	HTTDPSFLGR	0.03
B*13:02	6,474	6,484	GQQGEVPVSI	0.03
B*13:02	6,700	6,710	SQLGGLHLLI	0.03
B*15:01	1,586	1,596	TQVVDMSMTY	0.03
B*15:01	1,772	1,782	AVMYMGTLSY	0.03
B*15:01	1,810	1,820	VMMSAPPAQY	0.03
B*15:01	2,594	2,604	KMFDAYVNTF	0.03
B*35:01	5,972	5,982	IPGIPKDMTY	0.03
B*38:01	387	397	YHNESGLKTI	0.03
B*38:01	1,841	1,851	YKHITSKETL	0.03
B*38:01	4,777	4,787	MHAASGNLLL	0.03
B*38:01	6,698	6,708	SHSQLGGLHL	0.03
B*44:02	1,369	1,379	KQEILGTVSW	0.03
B*44:02	3,950	3,960	ASEFSSLPSY	0.03
B*44:02	5,005	5,015	DVENPHLMGW	0.03
B*44:03	912	922	GEFKLASHMY	0.03
B*44:03	3,950	3,960	ASEFSSLPSY	0.03
B*44:03	6,007	6,017	EEAIRHVRAW	0.03
B*53:01	6,271	6,281	VPQADVEWKF	0.03
B*57:01	5,304	5,314	LTNDNTSRYW	0.03
B*58:01	5,487	5,497	LSDRELHLSW	0.03
A*01:01	4,846	4,856	AISDYDYYRY	0.04
A*01:01	5,303	5,313	MLTNDNTSRY	0.04
A*02:01	4,729	4,739	KIFVDGVPFV	0.04
A*03:01	2,196	2,206	KASMPTTIAK	0.04
A*03:01	2,516	2,526	KTYERHSLSH	0.04
A*03:01	3,984	3,994	KLKKSLNVAK	0.04
A*11:01	6,764	6,774	KSQDLSVVSK	0.04
A*23:01	2,598	2,608	AYVNTFSSTF	0.04
A*23:01	3,816	3,826	YDYLVSTQEF	0.04
A*24:02	2,598	2,608	AYVNTFSSTF	0.04
A*24:02	4,465	4,475	SYFVVKRHTF	0.04
A*29:02	2,509	2,519	YFDKAGQKTY	0.04
A*32:01	6,503	6,513	KTTLPVNVAF	0.04
A*68:01	110	120	HVGEIPVAYR	0.04
A*68:01	1,215	1,225	EVKPFITESK	0.04
A*68:02	102	112	ETLGVLVPHV	0.04
A*68:02	2,971	2,981	NTYLEGSVRV	0.04
B*07:02	79	89	APHGHVMVEL	0.04
B*07:02	4,224	4,234	TPKGPKVKYL	0.04
B*13:02	14	24	VQLSLPVLQV	0.04
B*13:02	1,202	1,212	KQVEQKIAEI	0.04

B*13:02	6, 889	6, 899	RQWLPTGTLL	0. 04
B*18:01	5, 272	5, 282	QEYADVFHLY	0. 04
B*27:05	6, 582	6, 592	GRVDGQVDLF	0. 04
B*38:01	5, 361	5, 371	DHVISTSHKL	0. 04
B*39:01	1, 841	1, 851	YKHITSKETL	0. 04
B*39:01	5, 288	5, 298	LHDELTGHML	0. 04
B*40:01	6, 497	6, 507	VELFENKTTL	0. 04
B*44:02	5, 826	5, 836	REFLTRNPAW	0. 04
B*44:03	632	642	EEKFKEGVEF	0. 04
B*44:03	1, 369	1, 379	KQEILGTVSW	0. 04
B*44:03	5, 005	5, 015	DVENPHLMGW	0. 04
B*45:01	376	386	SEVGPEHSLA	0. 04
B*51:01	275	285	FPLNSIIKTI	0. 04
B*51:01	5, 226	5, 236	LPYPDPSRIL	0. 04
B*53:01	61	71	LPQLEQPYVF	0. 04
B*53:01	5, 582	5, 592	YPTLNISDEF	0. 04
B*53:01	6, 322	6, 332	YPANSIVCRF	0. 04
B*57:01	1, 652	1, 662	MSALNHTKKW	0. 04
B*57:01	3, 466	3, 476	TTITVNVLAW	0. 04
B*58:01	5, 304	5, 314	LTNDNTSRYW	0. 04
C*01:02	2, 870	2, 880	VVPGLPGTIL	0. 04
C*04:01	907	917	LFDESGEFKL	0. 04
C*04:43	907	917	LFDESGEFKL	0. 04
C*14:02	1, 540	1, 550	VYYTSNPTTF	0. 04
A*01:01	4, 653	4, 663	HVDTDLTKPY	0. 05
A*01:01	7, 036	7, 046	NTNPIQLSSY	0. 05
A*02:06	4, 729	4, 739	KIFVDGVPFV	0. 05
A*03:01	1, 190	1, 200	KLVSSFLEMK	0. 05
A*03:01	1, 392	1, 402	KLMPVCVETK	0. 05
A*03:01	2, 753	2, 763	RQVVNVVTTK	0. 05
A*03:01	4, 438	4, 448	KVAGFAKFLK	0. 05
A*26:01	567	577	ITILDGISQY	0. 05
A*26:01	4, 223	4, 233	DTPKGPKVKY	0. 05
A*26:01	6, 570	6, 580	ETICAPLTVF	0. 05
A*26:01	7, 036	7, 046	NTNPIQLSSY	0. 05
A*31:01	634	644	KFKEGVEFLR	0. 05
A*32:01	3, 578	3, 588	KRTIKGTHHW	0. 05
A*32:01	4, 897	4, 907	KSAGFPFNKW	0. 05
A*68:01	2, 025	2, 035	ETSNSFDVLK	0. 05
B*07:02	2, 113	2, 123	KKPNELSRVL	0. 05
B*07:02	3, 399	3, 409	RPNFTIKGSF	0. 05
B*15:01	1, 532	1, 542	FLKRGDKSVY	0. 05
B*35:01	2, 021	2, 031	TKPVETSNSF	0. 05

B*35:01	3, 645	3, 655	LLPSLATVAY	0. 05
B*35:01	5, 410	5, 420	FPLCANGQVF	0. 05
B*35:03	5, 226	5, 236	LPYPDPSRIL	0. 05
B*38:01	1, 617	1, 627	SHEGKTFYVL	0. 05
B*40:02	3, 910	3, 920	TEAFEKMVSL	0. 05
B*40:02	6, 497	6, 507	VELFENKTTL	0. 05
B*44:02	632	642	EEKFKEGVEF	0. 05
B*44:02	912	922	GEFKLASHMY	0. 05
B*44:02	6, 943	6, 953	TKENDSKEGF	0. 05
B*44:02	6, 944	6, 954	KENDSKEGFF	0. 05
B*44:03	5, 826	5, 836	REFLTRNPAW	0. 05
B*45:01	3, 951	3, 961	SEFSSLPSYA	0. 05
B*49:01	2, 057	2, 067	VENPTIQKDV	0. 05
B*53:01	4, 265	4, 275	VPANSTVLSF	0. 05
B*53:01	5, 972	5, 982	IPGIPKDMTY	0. 05
B*53:01	6, 726	6, 736	IPMDSTVKNY	0. 05
B*57:01	2, 323	2, 333	LTAFGLVAEW	0. 05
B*57:01	6, 503	6, 513	KTTLPVNVAF	0. 05
B*58:01	3, 466	3, 476	TTITVNVLAW	0. 05
C*04:01	4, 530	4, 540	HFDEGNCDTL	0. 05
C*04:43	4, 530	4, 540	HFDEGNCDTL	0. 05
C*05:01	5, 003	5, 013	YSDVENPHLM	0. 05
C*07:01	3, 371	3, 381	FVRIQPGQTF	0. 05
C*08:01	5, 003	5, 013	YSDVENPHLM	0. 05
A*01:01	4, 202	4, 212	PKSDGTGTIY	0. 06
A*01:01	6, 673	6, 683	LAMDEFIERY	0. 06
A*03:01	1, 879	1, 889	TTTIKPVTYK	0. 06
A*03:01	4, 720	4, 730	LTSFGPLVRK	0. 06
A*11:01	1, 525	1, 535	STQLGIEFLK	0. 06
A*11:01	1, 777	1, 787	GTLSYEQFKK	0. 06
A*11:01	4, 077	4, 087	VVIPDYNTYK	0. 06
A*24:02	834	844	QGYKSVNITF	0. 06
A*24:02	5, 273	5, 283	EYADVFHLYL	0. 06
A*24:02	5, 545	5, 555	TYKLNVGDYF	0. 06
A*25:01	1, 732	1, 742	DVRETMSYLF	0. 06
A*25:01	4, 076	4, 086	MVVIPDYNTY	0. 06
A*26:01	670	680	EIKESVQTFF	0. 06
A*26:01	1, 418	1, 428	EGVVDYGARF	0. 06
A*26:01	1, 586	1, 596	TQVVDMSMTY	0. 06
A*26:01	2, 500	2, 510	TVKNGSIHLY	0. 06
A*26:01	4, 871	4, 881	EVVDKYFDCY	0. 06
A*26:01	6, 627	6, 637	EAVKTQFNYY	0. 06
A*29:02	1, 772	1, 782	AVMYMGTLSY	0. 06

A*29:02	1,810	1,820	VMMSAPPAQY	0.06
A*33:01	2,722	2,732	DFMSLSEQLR	0.06
A*33:01	4,506	4,516	DMVPHISRQR	0.06
A*68:01	2,467	2,477	STFISDEVAR	0.06
B*07:02	5,226	5,236	LPYPDPSRIL	0.06
B*07:02	5,831	5,841	RNPAWRKAVF	0.06
B*15:01	669	679	KEIKESVQTF	0.06
B*15:01	3,904	3,914	LLAKDTTEAF	0.06
B*18:01	4,457	4,467	DEDDNLIDSY	0.06
B*35:01	5,582	5,592	YPTLNISDEF	0.06
B*38:01	4,477	4,487	YQHEETIYNL	0.06
B*39:01	1,503	1,513	EEHFIETISL	0.06
B*39:01	1,549	1,559	FHLDGEVITF	0.06
B*39:01	1,617	1,627	SHEGKTFYVL	0.06
B*40:01	1,140	1,150	YENFNQHEVL	0.06
B*40:01	3,910	3,920	TEAFEKMVSL	0.06
B*40:01	4,263	4,273	TEVPANSTVL	0.06
B*40:02	669	679	KEIKESVQTF	0.06
B*44:03	5,469	5,479	ATEETFKLSY	0.06
B*46:01	404	414	IAFGGCVFSY	0.06
B*49:01	4,262	4,272	ATEVPANSTV	0.06
B*53:01	1,325	1,335	VPTDNYITTY	0.06
B*53:01	3,730	3,740	NALDQAISMW	0.06
B*53:01	5,410	5,420	FPLCANGQVF	0.06
B*57:01	3,662	3,672	ASWVMRIMTW	0.06
B*57:01	4,092	4,102	TTFTYASALW	0.06
B*58:01	1,652	1,662	MSALNHTKKW	0.06
B*58:01	6,269	6,279	KCVPQADVEW	0.06
C*01:02	1,812	1,822	MSAPPAQYEL	0.06
C*03:02	3,371	3,381	FVRIQPGQTF	0.06
C*04:01	6,693	6,703	VYGDFSHSQL	0.06
C*04:43	6,693	6,703	VYGDFSHSQL	0.06
C*07:01	6,713	6,723	KRFKESPFEL	0.06
C*07:02	3,371	3,381	FVRIQPGQTF	0.06
A*01:01	1,895	1,905	TEIDPKLDNY	0.07
A*01:01	5,077	5,087	TSSGDATTAY	0.07
A*03:01	5,608	5,618	TLQGPPGTGK	0.07
A*23:01	834	844	QGYKSVNITF	0.07
A*23:01	905	915	YYLFDESGEF	0.07
A*23:01	3,171	3,181	VFNGVSFSTF	0.07
A*24:02	905	915	YYLFDESGEF	0.07
A*24:02	1,863	1,873	EYKGPITDVF	0.07
A*24:02	3,171	3,181	VFNGVSFSTF	0.07

A*24:02	7,044	7,054	SYSLFDMSKF	0.07
A*25:01	2,137	2,147	SVPWDTIANY	0.07
A*25:01	2,500	2,510	TVKNGSIHLY	0.07
A*25:01	4,223	4,233	DTPKGPKVKY	0.07
A*25:01	6,627	6,637	EAVKTQFNYY	0.07
A*26:01	1,448	1,458	ETLVTMPLGY	0.07
A*26:01	1,971	1,981	DVVAIDYKHY	0.07
A*26:01	4,076	4,086	MVVIPDYNTY	0.07
A*29:02	1,252	1,262	KFLTENLLLY	0.07
A*29:02	3,818	3,828	YLVSTQEFRY	0.07
A*29:02	5,391	5,401	QLYLGGMSYY	0.07
A*29:02	5,626	5,636	LYYPSARIVY	0.07
A*31:01	6,126	6,136	KYFVKIGPER	0.07
A*32:01	527	537	KSILSPLYAF	0.07
A*33:01	3,995	4,005	EFDRDAAMQR	0.07
A*68:01	162	172	NTKHSSGVTR	0.07
A*68:01	828	838	DTVIEVQGYK	0.07
A*68:01	1,029	1,039	EVNSFSGYLK	0.07
A*68:01	2,652	2,662	ETKDVVECLK	0.07
A*68:01	6,672	6,682	ELAMDEFIER	0.07
A*68:02	5,776	5,786	EIVDTVSALV	0.07
B*07:02	1,981	1,991	TPSFKKGAKL	0.07
B*13:02	1,781	1,791	YEQFKKGVQI	0.07
B*13:02	6,664	6,674	SQMEIDFLEL	0.07
B*13:02	6,765	6,775	SQDLSVVSKV	0.07
B*15:01	5,391	5,401	QLYLGGMSYY	0.07
B*18:01	632	642	EEKFKEGVEF	0.07
B*18:01	4,918	4,928	YEDQDALFAY	0.07
B*35:01	61	71	LPQLEQPYVF	0.07
B*35:03	7,038	7,048	NPIQLSSYSL	0.07
B*38:01	6,416	6,426	HHANEYRLYL	0.07
B*40:01	5,207	5,217	HEFCSQHTML	0.07
B*40:01	6,224	6,234	IEYPIIGDEL	0.07
B*40:02	1,140	1,150	YENFNQHEVL	0.07
B*40:02	5,775	5,785	AEIVDTVSAL	0.07
B*44:03	232	242	REHEHEIAWY	0.07
B*44:03	471	481	EEIAIILASF	0.07
B*44:03	6,943	6,953	TKENDSKEGF	0.07
C*04:01	4,816	4,826	FYDFAVSKGF	0.07
C*04:43	4,816	4,826	FYDFAVSKGF	0.07
C*05:01	6,054	6,064	YVDTPDNTDF	0.07
A*01:01	896	906	DLDEWSMATY	0.08
A*02:05	4,477	4,487	YQHEETIYNL	0.08

A*11:01	4,438	4,448	KVAGFAKFLK	0.08
A*11:01	4,720	4,730	LTSFGPLVRK	0.08
A*11:01	6,482	6,492	SIINNTVYTK	0.08
A*11:01	7,043	7,053	SSYSLFDMSK	0.08
A*23:01	1,638	1,648	EYYHTTDPSF	0.08
A*23:01	2,171	2,181	TNYMPYFFTL	0.08
A*23:01	5,273	5,283	EYADVFHLYL	0.08
A*24:02	1,903	1,913	NYYKKDNSYF	0.08
A*24:02	2,171	2,181	TNYMPYFFTL	0.08
A*25:01	567	577	ITILDGISQY	0.08
A*25:01	3,371	3,381	FVRIQPGQTF	0.08
A*25:01	4,433	4,443	DIYNDKVAGF	0.08
A*26:01	1,732	1,742	DVRETMSYLF	0.08
A*26:01	2,590	2,600	EVAVKMFDAY	0.08
A*26:01	2,669	2,679	EVTGDSCNNY	0.08
A*26:01	4,433	4,443	DIYNDKVAGF	0.08
A*26:01	4,983	4,993	TVVIGTSKFY	0.08
A*33:01	3,817	3,827	DYLVSTQEFR	0.08
A*68:02	1,880	1,890	TTIKPVTYKL	0.08
B*07:02	3,037	3,047	TPLIQPIGAL	0.08
B*08:01	2,608	2,618	NVPMEKLKTL	0.08
B*08:01	5,250	5,260	TLMIERFVSL	0.08
B*13:02	5,732	5,742	AQLPAPRTLL	0.08
B*13:02	7,068	7,078	GQINDMILSL	0.08
B*15:01	2,776	2,786	KQLIKVTLVF	0.08
B*15:01	3,740	3,750	ALIISVTSNY	0.08
B*15:01	4,580	4,590	RQALLKTVQF	0.08
B*15:01	4,762	4,772	RLSFKELLVY	0.08
B*15:01	5,819	5,829	RPQIGVVREF	0.08
B*15:01	6,805	6,815	SQAWQPGVAM	0.08
B*15:03	1,414	1,424	IKIQEGVVDY	0.08
B*27:05	4,909	4,919	ARLYYDSMSY	0.08
B*35:01	4,265	4,275	VPANSTVLSF	0.08
B*35:01	5,269	5,279	HPNQEYADVF	0.08
B*35:01	6,322	6,332	YPANSIVCRF	0.08
B*39:01	12	22	THVQLSLPVL	0.08
B*39:01	387	397	YHNESGLKTI	0.08
B*39:01	4,777	4,787	MHAASGNLLL	0.08
B*39:01	5,361	5,371	DHVISTSHKL	0.08
B*40:01	1,728	1,738	GELGDVRETM	0.08
B*40:02	1,728	1,738	GELGDVRETM	0.08
B*40:02	1,862	1,872	SEYKGPITDV	0.08
B*40:02	5,207	5,217	HEFCSQHTML	0.08

B*44:02	5,469	5,479	ATEETFKLSY	0.08
B*44:03	6,944	6,954	KENDSKEGFF	0.08
B*45:01	1,805	1,815	QESPFVMMSA	0.08
B*49:01	1,019	1,029	MELTPVVQTI	0.08
B*53:01	2,021	2,031	TKPVETSNSF	0.08
B*57:01	527	537	KSILSPLYAF	0.08
B*58:01	4,092	4,102	TTFTYASALW	0.08
B*58:01	6,148	6,158	STASDTYACW	0.08
B*58:01	6,503	6,513	KTTLPVNVAF	0.08
C*04:01	2,509	2,519	YFDKAGQKTY	0.08
C*04:43	2,509	2,519	YFDKAGQKTY	0.08
C*08:02	5,003	5,013	YSDVENPHLM	0.08
C*14:02	5,626	5,636	LYYPSARIVY	0.08
A*01:01	1,170	1,180	CVDTVRTNVY	0.09
A*01:01	1,482	1,492	VSSPDAVTAY	0.09
A*01:01	2,557	2,567	SSAKSASVYY	0.09
A*01:01	5,003	5,013	YSDVENPHLM	0.09
A*02:02	3,334	3,344	FLVQAGNVQL	0.09
A*02:05	4,729	4,739	KIFVDGVPFV	0.09
A*11:01	4,805	4,815	QTVKPGNFNK	0.09
A*23:01	1,903	1,913	NYYKKDNSYF	0.09
A*23:01	5,545	5,555	TYKLNVGDYF	0.09
A*24:02	1,638	1,648	EYYHTTDPSF	0.09
A*25:01	1,665	1,675	QVNGLTSIKW	0.09
A*25:01	5,133	5,143	DVDTDFVNEF	0.09
A*26:01	5,062	5,072	EMVMCGGSLY	0.09
A*26:01	5,303	5,313	MLTNDNTSRY	0.09
A*29:02	6,075	6,085	QFKHLIPLMY	0.09
A*29:02	7,036	7,046	NTNPIQLSSY	0.09
A*31:01	5,475	5,485	KLSYGIATVR	0.09
A*68:01	1,879	1,889	TTTIKPVTYK	0.09
A*68:01	3,223	3,233	SGAMDTTSYR	0.09
B*07:02	5,819	5,829	RPQIGVVREF	0.09
B*13:02	6,615	6,625	KQASLNGVTL	0.09
B*15:03	6,805	6,815	SQAWQPGVAM	0.09
B*18:01	939	949	EEFEPSTQYE	0.09
B*18:01	1,895	1,905	TEIDPKLDNY	0.09
B*18:01	4,563	4,573	VENPDILRVY	0.09
B*35:03	3,617	3,627	LPFAMGIIAM	0.09
B*35:03	5,410	5,420	FPLCANGQVF	0.09
B*38:01	12	22	THVQLSLPVL	0.09
B*38:01	1,503	1,513	EEHFIETISL	0.09
B*38:01	1,639	1,649	YYHTTDPSFL	0.09

B*38:01	4, 776	4, 786	AMHAASGNLL	0. 09
B*40:01	524	534	GEQKSILSPL	0. 09
B*40:02	524	534	GEQKSILSPL	0. 09
B*44:02	232	242	REHEHEIAWY	0. 09
B*45:01	1, 200	1, 210	SEKQVEQKIA	0. 09
B*46:01	3, 960	3, 970	AAFATAQEAY	0. 09
B*49:01	112	122	GEIPVAYRKV	0. 09
B*53:01	5, 269	5, 279	HPNQEYADVF	0. 09
B*57:01	2, 709	2, 719	AKSHNIALIW	0. 09
B*58:01	2, 323	2, 333	LTAFGLVAEW	0. 09
B*58:01	6, 107	6, 117	LSDRVVFVLW	0. 09
C*01:02	2, 952	2, 962	SLRPDTRYVL	0. 09
C*01:02	4, 263	4, 273	TEVPANSTVL	0. 09
C*01:02	5, 560	5, 570	TVMPLSAPTL	0. 09
C*02:02	3, 371	3, 381	FVRIQPGQTF	0. 09
C*02:02	6, 067	6, 077	SAKPPPGDQF	0. 09
C*07:02	1, 540	1, 550	VYYTSNPTTF	0. 09
C*08:02	6, 054	6, 064	YVDTPDNTDF	0. 09
A*01:01	487	497	FVETVKGLDY	0. 1
A*01:01	1, 878	1, 888	YTTTIKPVTY	0. 1
A*01:01	3, 222	3, 232	FSGAMDTTSY	0. 1
A*02:06	6, 664	6, 674	SQMEIDFLEL	0. 1
A*03:01	2, 507	2, 517	HLYFDKAGQK	0. 1
A*03:01	6, 606	6, 616	KGLQPSVGPK	0. 1
A*03:01	6, 764	6, 774	KSQDLSVVSK	0. 1
A*11:01	3, 264	3, 274	SAVLQSGFRK	0. 1
A*23:01	1, 863	1, 873	EYKGPITDVF	0. 1
A*23:01	2, 172	2, 182	NYMPYFFTLL	0. 1
A*23:01	7, 044	7, 054	SYSLFDMSKF	0. 1
A*24:02	1, 800	1, 810	KYLVQQESPF	0. 1
A*24:02	3, 253	3, 263	VLYQPPQTSI	0. 1
A*24:02	6, 693	6, 703	VYGDFSHSQL	0. 1
A*25:01	1, 586	1, 596	TQVVDMSMTY	0. 1
A*25:01	3, 124	3, 134	DVSFLAHIQW	0. 1
A*26:01	827	837	DDTVIEVQGY	0. 1
A*31:01	5, 028	5, 038	RIMASLVLAR	0. 1
A*32:01	1, 369	1, 379	KQEILGTVSW	0. 1
A*33:01	162	172	NTKHSSGVTR	0. 1
A*33:01	2, 007	2, 017	TYKPNTWCIR	0. 1
A*33:01	5, 710	5, 720	NYDLSVVNAR	0. 1
A*33:01	7, 049	7, 059	DMSKFPLKLR	0. 1
A*68:01	2, 604	2, 614	SSTFNVPMEK	0. 1
B*08:01	5, 143	5, 153	YAYLRKHFSM	0. 1

B*13:02	3, 336	3, 346	VQAGNVQLRV	0. 1
B*13:02	3, 459	3, 469	AQAAGTDTTI	0. 1
B*15:01	4, 909	4, 919	ARLYYDSMSY	0. 1
B*15:01	5, 929	5, 939	LQAENVTGLF	0. 1
B*18:01	6, 419	6, 429	NEYRLYLDAY	0. 1
B*27:05	2, 475	2, 485	ARDLSLQFKR	0. 1
B*27:05	3, 168	3, 178	RRVVFNGVSF	0. 1
B*38:01	44	54	QHLKDGTCGL	0. 1
B*38:01	3, 080	3, 090	SHVVAFNTLL	0. 1
B*39:01	4, 478	4, 488	QHEETIYNLL	0. 1
B*39:01	6, 416	6, 426	HHANEYRLYL	0. 1
B*39:01	6, 698	6, 708	SHSQLGGLHL	0. 1
B*40:01	669	679	KEIKESVQTF	0. 1
B*44:02	471	481	EEIAIILASF	0. 1
B*44:02	621	631	YEKLKPVLDW	0. 1
B*46:01	6, 673	6, 683	LAMDEFIERY	0. 1
B*53:01	3, 646	3, 656	LPSLATVAYF	0. 1
B*57:01	3, 151	3, 161	ICISTKHFYW	0. 1
B*57:01	6, 107	6, 117	LSDRVVFVLW	0. 1
B*58:01	527	537	KSILSPLYAF	0. 1
C*04:01	1, 556	1, 566	ITFDNLKTLL	0. 1
C*04:43	1, 556	1, 566	ITFDNLKTLL	0. 1
A*11:01	252	262	QTPFEIKLAK	0. 11
A*23:01	1, 800	1, 810	KYLVQQESPF	0. 11
A*23:01	3, 118	3, 128	TFYLTNDVSF	0. 11
A*24:02	2, 172	2, 182	NYMPYFFTLL	0. 11
A*24:02	3, 118	3, 128	TFYLTNDVSF	0. 11
A*24:02	5, 002	5, 012	VYSDVENPHL	0. 11
A*29:02	2, 388	2, 398	MYIFFASFYY	0. 11
A*33:01	4, 561	4, 571	DFVENPDILR	0. 11
A*68:01	4, 421	4, 431	TGTSTDVVYR	0. 11
A*68:02	3, 103	3, 113	YSFLPGVYSV	0. 11
A*68:02	5, 385	5, 395	DVTDVTQLYL	0. 11
B*07:02	4, 639	4, 649	MPILTLTRAL	0. 11
B*13:02	1, 761	1, 771	GQQQTTLKGV	0. 11
B*15:01	1, 766	1, 776	TLKGVEAVMY	0. 11
B*15:01	3, 371	3, 381	FVRIQPGQTF	0. 11
B*15:03	232	242	REHEHEIAWY	0. 11
B*18:01	3, 076	3, 086	FGEYSHVVAF	0. 11
B*27:05	3, 007	3, 017	GRWVLNNDYY	0. 11
B*27:05	3, 371	3, 381	FVRIQPGQTF	0. 11
B*27:05	5, 887	5, 897	VNRFNVAITR	0. 11
B*35:03	196	206	YPLECIKDLL	0. 11

B*35:03	884	894	QPVSELLTPL	0. 11
B*35:03	4, 639	4, 649	MPILTLTRAL	0. 11
B*35:03	5, 582	5, 592	YPTLNISDEF	0. 11
B*37:01	669	679	KEIKESVQTF	0. 11
B*38:01	164	174	KHSSGVTREL	0. 11
B*40:01	6, 028	6, 038	REAVGTNLPL	0. 11
B*44:03	6, 626	6, 636	GEAVKTQFNY	0. 11
B*45:01	1, 209	1, 219	AEIPKEEVKP	0. 11
B*45:01	6, 665	6, 675	QMEIDFLELA	0. 11
B*46:01	567	577	ITILDGISQY	0. 11
B*51:01	4, 201	4, 211	FPKSDGTGTI	0. 11
B*51:01	4, 718	4, 728	FPLTSFGPLV	0. 11
B*53:01	6, 970	6, 980	VAIKITEHSW	0. 11
B*57:01	6, 148	6, 158	STASDTYACW	0. 11
B*57:01	6, 269	6, 279	KCVPQADVEW	0. 11
B*58:01	4, 189	4, 199	LLSDLQDLKW	0. 11
C*01:02	4, 066	4, 076	IIPLTTAAKL	0. 11
C*04:01	824	834	TFGDDTVIEV	0. 11
C*04:01	1, 187	1, 197	LYDKLVSSFL	0. 11
C*04:01	5, 910	5, 920	LYDKLQFTSL	0. 11
C*04:43	824	834	TFGDDTVIEV	0. 11
C*04:43	1, 187	1, 197	LYDKLVSSFL	0. 11
C*04:43	5, 910	5, 920	LYDKLQFTSL	0. 11
A*01:01	88	98	LVAELEGIQY	0. 12
A*01:01	790	800	LLEIKDTEKY	0. 12
A*01:01	5, 219	5, 229	QGDDYVYLPY	0. 12
A*02:01	3, 334	3, 344	FLVQAGNVQL	0. 12
A*02:02	445	455	GLNDNLLEIL	0. 12
A*02:02	1, 207	1, 217	KIAEIPKEEV	0. 12
A*02:02	4, 729	4, 739	KIFVDGVPFV	0. 12
A*02:05	1, 207	1, 217	KIAEIPKEEV	0. 12
A*02:06	4, 477	4, 487	YQHEETIYNL	0. 12
A*03:01	555	565	AQNSVRVLQK	0. 12
A*11:01	2, 753	2, 763	RQVVNVVTTK	0. 12
A*25:01	4, 871	4, 881	EVVDKYFDCY	0. 12
A*26:01	1, 896	1, 906	EIDPKLDNYY	0. 12
A*26:01	5, 133	5, 143	DVDTDFVNEF	0. 12
A*26:01	5, 384	5, 394	CDVTDVTQLY	0. 12
A*29:02	567	577	ITILDGISQY	0. 12
A*29:02	2, 500	2, 510	TVKNGSIHLY	0. 12
A*29:02	5, 618	5, 628	SHFAIGLALY	0. 12
A*32:01	669	679	KEIKESVQTF	0. 12
A*33:01	5, 827	5, 837	EFLTRNPAWR	0. 12

A*68:02	1,004	1,014	TTTIQTIVEV	0.12
B*07:02	3,389	3,399	SPSGVYQCAM	0.12
B*15:01	3,207	3,217	TQYNRYLALY	0.12
B*15:03	669	679	KEIKESVQTF	0.12
B*15:03	3,207	3,217	TQYNRYLALY	0.12
B*27:05	3,711	3,721	RRVWTLMNVL	0.12
B*35:01	3,617	3,627	LPFAMGIIAM	0.12
B*35:01	3,646	3,656	LPSLATVAYF	0.12
B*35:01	6,357	6,367	HTPAFDKSAF	0.12
B*35:03	2,021	2,031	TKPVETSNSF	0.12
B*38:01	5,948	5,958	LHPTQAPTHL	0.12
B*39:01	4,477	4,487	YQHEETIYNL	0.12
B*39:01	5,403	5,413	SHKPPISFPL	0.12
B*40:01	375	385	NSEVGPEHSL	0.12
B*44:02	6,626	6,636	GEAVKTQFNY	0.12
B*45:01	245	255	SEKSYELQTP	0.12
B*45:01	1,130	1,140	GEDIQLLKSA	0.12
B*46:01	1,772	1,782	AVMYMGTLSY	0.12
B*46:01	3,904	3,914	LLAKDTTEAF	0.12
B*46:01	6,067	6,077	SAKPPPGDQF	0.12
B*49:01	4,212	4,222	TELEPPCRFV	0.12
B*53:01	2,359	2,369	FAVHFISNSW	0.12
B*53:01	5,225	5,235	YLPYPDPSRI	0.12
B*53:01	5,226	5,236	LPYPDPSRIL	0.12
B*57:01	2,629	2,639	VSLDNVLSTF	0.12
B*57:01	3,579	3,589	RTIKGTHHWL	0.12
B*57:01	3,987	3,997	KSLNVAKSEF	0.12
B*58:01	2,629	2,639	VSLDNVLSTF	0.12
C*04:01	5,002	5,012	VYSDVENPHL	0.12
C*04:43	5,002	5,012	VYSDVENPHL	0.12
C*05:01	4,107	4,117	VDADSKIVQL	0.12
A*02:02	5,225	5,235	YLPYPDPSRI	0.13
A*02:06	2,869	2,879	FVVPGLPGTI	0.13
A*03:01	1,856	1,866	ALLTKSSEYK	0.13
A*11:01	483	493	STSAFVETVK	0.13
A*11:01	3,528	3,538	AVLDMCASLK	0.13
A*24:02	1,924	1,934	PYPNASFDNF	0.13
A*24:02	6,298	6,308	YSYATHSDKF	0.13
A*25:01	2,141	2,151	DTIANYAKPF	0.13
A*25:01	2,590	2,600	EVAVKMFDAY	0.13
A*26:01	176	186	ELNGGAYTRY	0.13
A*29:02	404	414	IAFGGCVFSY	0.13
A*29:02	4,682	4,692	RYFKYWDQTY	0.13

A*31:01	4,971	4,981	KLLKSIAATR	0.13
A*31:01	5,652	5,662	KYLPIDKCSR	0.13
A*33:01	1,559	1,569	DNLKTLLSLR	0.13
A*33:01	3,658	3,668	VYMPASWVMR	0.13
A*33:01	6,446	6,456	TYNLWNTFTR	0.13
A*33:01	6,509	6,519	NVAFELWAKR	0.13
A*68:01	2	12	ESLVPGFNEK	0.13
A*68:01	2,814	2,824	YKAIDGGVTR	0.13
B*07:02	4,341	4,351	HPNPKGFCDL	0.13
B*15:01	1,321	1,331	ALRKVPTDNY	0.13
B*15:01	1,523	1,533	GQSTQLGIEF	0.13
B*15:03	2,594	2,604	KMFDAYVNTF	0.13
B*35:01	360	370	LPQNAVVKIY	0.13
B*35:01	404	414	IAFGGCVFSY	0.13
B*35:01	1,695	1,705	FNPPALQDAY	0.13
B*35:01	2,137	2,147	SVPWDTIANY	0.13
B*35:01	3,140	3,150	LVPFWITIAY	0.13
B*35:01	6,341	6,351	LPGCDGGSLY	0.13
B*35:03	4,628	4,638	VPVVDSYYSL	0.13
B*46:01	1,482	1,492	VSSPDAVTAY	0.13
B*46:01	5,537	5,547	AVVYRGTTTY	0.13
B*49:01	1,781	1,791	YEQFKKGVQI	0.13
B*51:01	1,663	1,673	YPQVNGLTSI	0.13
C*01:02	4,932	4,942	VIPTITQMNL	0.13
C*05:01	569	579	ILDGISQYSL	0.13
C*06:02	7,033	7,043	FWRNTNPIQL	0.13
C*08:02	4,107	4,117	VDADSKIVQL	0.13
C*12:02	3,371	3,381	FVRIQPGQTF	0.13
C*14:02	2,509	2,519	YFDKAGQKTY	0.13
C*14:02	4,825	4,835	FFKEGSSVEL	0.13
C*15:02	77	87	RTAPHGHVMV	0.13
A*01:01	3,360	3,370	DTANPKTPKY	0.14
A*01:01	5,863	5,873	DSSQGSEYDY	0.14
A*02:01	4,098	4,108	SALWEIQQVV	0.14
A*02:02	1,442	1,452	TLNDLNETLV	0.14
A*02:02	2,222	2,232	YLKSPNFSKL	0.14
A*03:01	710	720	VTHSKGLYRK	0.14
A*03:01	1,401	1,411	KAIVSTIQRK	0.14
A*03:01	1,772	1,782	AVMYMGTLSY	0.14
A*03:01	7,043	7,053	SSYSLFDMSK	0.14
A*11:01	555	565	AQNSVRVLQK	0.14
A*23:01	5,002	5,012	VYSDVENPHL	0.14
A*23:01	6,693	6,703	VYGDFSHSQL	0.14

A*25:01	1,971	1,981	DVVAIDYKHY	0.14
A*25:01	3,466	3,476	TTITVNVLAW	0.14
A*26:01	5,291	5,301	ELTGHMLDMY	0.14
A*29:02	1,586	1,596	TQVVDMSMTY	0.14
A*29:02	2,213	2,223	KFCLEASFNY	0.14
A*29:02	3,715	3,725	TLMNVLTLVY	0.14
A*32:01	915	925	KLASHMYCSF	0.14
A*33:01	110	120	HVGEIPVAYR	0.14
A*68:02	1,434	1,444	TTVASLINTL	0.14
B*07:02	4,507	4,517	MVPHISRQRL	0.14
B*08:01	4,224	4,234	TPKGPKVKYL	0.14
B*08:01	7,053	7,063	FPLKLRGTAV	0.14
B*15:03	1,586	1,596	TQVVDMSMTY	0.14
B*15:03	5,819	5,829	RPQIGVVREF	0.14
B*18:01	471	481	EEIAIILASF	0.14
B*18:01	3,950	3,960	ASEFSSLPSY	0.14
B*35:03	3,037	3,047	TPLIQPIGAL	0.14
B*38:01	2,834	2,844	NKHADFDTWF	0.14
B*40:01	2,035	2,045	SEDAQGMDNL	0.14
B*44:03	5,589	5,599	DEFSSNVANY	0.14
B*45:01	3,432	3,442	HMELPTGVHA	0.14
B*45:01	6,006	6,016	REEAIRHVRA	0.14
B*46:01	1,810	1,820	VMMSAPPAQY	0.14
B*49:01	5,318	5,328	YEAMYTPHTV	0.14
B*58:01	2,359	2,369	FAVHFISNSW	0.14
B*58:01	2,880	2,890	RTTNGDFLHF	0.14
B*58:01	3,579	3,589	RTIKGTHHWL	0.14
C*01:02	5,225	5,235	YLPYPDPSRI	0.14
C*01:02	5,732	5,742	AQLPAPRTLL	0.14
C*03:02	404	414	IAFGGCVFSY	0.14
C*03:04	1,812	1,822	MSAPPAQYEL	0.14
C*04:01	4,825	4,835	FFKEGSSVEL	0.14
C*04:01	6,360	6,370	AFDKSAFVNL	0.14
C*04:43	4,825	4,835	FFKEGSSVEL	0.14
C*04:43	6,360	6,370	AFDKSAFVNL	0.14
C*14:02	905	915	YYLFDESGEF	0.14
A*01:01	240	250	WYTERSEKSY	0.15
A*02:02	468	478	KLNEEIAIIL	0.15
A*03:01	3,898	3,908	QLHNDILLAK	0.15
A*03:01	3,925	3,935	SMQGAVDINK	0.15
A*03:01	4,486	4,496	LLKDCPAVAK	0.15
A*11:01	1,276	1,286	VSDIDITFLK	0.15
A*11:01	3,359	3,369	VDTANPKTPK	0.15

A*23:01	7, 024	7, 034	GYVMHANYIF	0. 15
A*24:02	181	191	AYTRYVDNNF	0. 15
A*24:02	2, 517	2, 527	TYERHSLSHF	0. 15
A*25:01	2, 587	2, 597	DSAEVAVKMF	0. 15
A*25:01	4, 983	4, 993	TVVIGTSKFY	0. 15
A*29:02	940	950	EFEPSTQYEY	0. 15
A*29:02	1, 878	1, 888	YTTTIKPVTY	0. 15
A*29:02	5, 390	5, 400	TQLYLGGMSY	0. 15
A*31:01	1, 562	1, 572	KTLLSLREVR	0. 15
A*32:01	4, 120	4, 130	SMDNSPNLAW	0. 15
A*68:02	3, 911	3, 921	EAFEKMVSLL	0. 15
B*07:02	5, 663	5, 673	IPARARVECF	0. 15
B*15:01	2, 960	2, 970	VLMDGSIIQF	0. 15
B*15:03	2, 776	2, 786	KQLIKVTLVF	0. 15
B*15:03	4, 580	4, 590	RQALLKTVQF	0. 15
B*35:03	1, 453	1, 463	MPLGYVTHGL	0. 15
B*35:03	2, 935	2, 945	VPYCYDTNVL	0. 15
B*38:01	5, 403	5, 413	SHKPPISFPL	0. 15
B*40:01	388	398	HNESGLKTIL	0. 15
B*40:01	724	734	REETGLLMPL	0. 15
B*40:01	749	759	TEVLTEEVVL	0. 15
B*40:01	960	970	LEFGATSAAL	0. 15
B*40:01	1, 862	1, 872	SEYKGPITDV	0. 15
B*40:01	2, 865	2, 875	REVGFVVPGL	0. 15
B*44:03	621	631	YEKLKPVLDW	0. 15
B*44:03	939	949	EEFEPSTQYE	0. 15
B*44:03	5, 525	5, 535	GEYTFEKGDY	0. 15
B*45:01	1, 862	1, 872	SEYKGPITDV	0. 15
B*45:01	2, 553	2, 563	KCEESSAKSA	0. 15
B*45:01	5, 918	5, 928	SLEIPRRNVA	0. 15
B*46:01	2, 594	2, 604	KMFDAYVNTF	0. 15
B*51:01	6, 479	6, 489	VPVSIINNTV	0. 15
B*57:01	1, 686	1, 696	LTLQQIELKF	0. 15
B*58:01	2, 709	2, 719	AKSHNIALIW	0. 15
B*58:01	3, 477	3, 487	YAAVINGDRW	0. 15
C*02:02	567	577	ITILDGISQY	0. 15
C*07:01	7, 033	7, 043	FWRNTNPIQL	0. 15
C*08:02	569	579	ILDGISQYSL	0. 15
C*12:02	6, 067	6, 077	SAKPPPGDQF	0. 15
C*14:02	1, 158	1, 168	IFGADPIHSL	0. 15
C*14:02	3, 816	3, 826	YDYLVSTQEF	0. 15
A*02:01	569	579	ILDGISQYSL	0. 16
A*02:02	4, 519	4, 529	YTMADLVYAL	0. 16

A*11:01	710	720	VTHSKGLYRK	0.16
A*11:01	1,062	1,072	VVNAANVYLK	0.16
A*23:01	3,253	3,263	VLYQPPQTSI	0.16
A*24:02	3,688	3,698	VMYASAVVLL	0.16
A*26:01	2,941	2,951	TNVLEGSVAY	0.16
A*31:01	5,123	5,133	RLYECLYRNR	0.16
A*68:01	750	760	EVLTEEVVLK	0.16
B*07:02	4,265	4,275	VPANSTVLSF	0.16
B*08:01	3,195	3,205	YLKLRSDVLL	0.16
B*15:01	2,500	2,510	TVKNGSIHLY	0.16
B*15:01	3,377	3,387	GQTFSVLACY	0.16
B*15:03	4,085	4,095	YKNTCDGTTF	0.16
B*15:03	4,517	4,527	TKYTMADLVY	0.16
B*35:01	4,009	4,019	MADQAMTQMY	0.16
B*35:01	5,566	5,576	APTLVPQEHY	0.16
B*38:01	5,792	5,802	AHKDKSAQCF	0.16
B*46:01	3,105	3,115	FLPGVYSVIY	0.16
B*46:01	3,953	3,963	FSSLPSYAAF	0.16
B*53:01	6,882	6,892	APGTAVLRQW	0.16
B*57:01	1,665	1,675	QVNGLTSIKW	0.16
B*57:01	2,359	2,369	FAVHFISNSW	0.16
B*57:01	4,678	4,688	KLFDRYFKYW	0.16
B*58:01	4,916	4,926	MSYEDQDALF	0.16
B*58:01	6,289	6,299	KAYKIEELFY	0.16
C*01:02	6,505	6,515	TLPVNVAFEL	0.16
C*06:02	6,713	6,723	KRFKESPFEL	0.16
C*07:01	76	86	ARTAPHGHVM	0.16
C*07:02	5,626	5,636	LYYPSARIVY	0.16
C*08:01	4,107	4,117	VDADSKIVQL	0.16
C*14:02	1,639	1,649	YYHTTDPSFL	0.16
C*17:01	4,107	4,117	VDADSKIVQL	0.16
C*17:01	5,003	5,013	YSDVENPHLM	0.16
C*17:03	4,107	4,117	VDADSKIVQL	0.16
C*17:03	5,003	5,013	YSDVENPHLM	0.16
A*02:01	5,225	5,235	YLPYPDPSRI	0.17
A*02:02	569	579	ILDGISQYSL	0.17
A*02:02	5,928	5,938	TLQAENVTGL	0.17
A*02:05	5,725	5,735	YVYIGDPAQL	0.17
A*02:05	6,664	6,674	SQMEIDFLEL	0.17
A*03:01	2,081	2,091	ILKPANNSLK	0.17
A*23:01	2,362	2,372	HFISNSWLMW	0.17
A*24:02	2,305	2,315	TYPSLETIQI	0.17
A*24:02	6,440	6,450	VYKQFDTYNL	0.17

A*24:02	7,024	7,034	GYVMHANYIF	0.17
A*25:01	539	549	EAARVVRSIF	0.17
A*25:01	1,448	1,458	ETLVTMPLGY	0.17
A*26:01	1,507	1,517	IETISLAGSY	0.17
A*26:01	1,878	1,888	YTTTIKPVTY	0.17
A*26:01	3,371	3,381	FVRIQPGQTF	0.17
A*29:02	88	98	LVAELEGIQY	0.17
A*29:02	1,642	1,652	TTDPSFLGRY	0.17
A*29:02	2,137	2,147	SVPWDTIANY	0.17
A*29:02	2,893	2,903	VFSAVGNICY	0.17
A*29:02	5,275	5,285	ADVFHLYLQY	0.17
A*29:02	5,597	5,607	NYQKVGMQKY	0.17
A*33:01	3,159	3,169	YWFFSNYLKR	0.17
A*68:02	586	596	FTSDLATNNL	0.17
A*68:02	2,881	2,891	TTNGDFLHFL	0.17
A*68:02	4,519	4,529	YTMADLVYAL	0.17
A*68:02	6,358	6,368	TPAFDKSAFV	0.17
B*15:01	567	577	ITILDGISQY	0.17
B*15:03	2,021	2,031	TKPVETSNSF	0.17
B*15:03	5,994	6,004	YQVNGYPNMF	0.17
B*35:01	6,799	6,809	YPKLQSSQAW	0.17
B*35:03	2,572	2,582	QPILLLDQAL	0.17
B*38:01	2,802	2,812	SKHTDFSSEI	0.17
B*38:01	6,259	6,269	LHDIGNPKAI	0.17
B*39:01	3,080	3,090	SHVVAFNTLL	0.17
B*44:02	1,370	1,380	QEILGTVSWN	0.17
B*44:02	2,950	2,960	YESLRPDTRY	0.17
B*44:03	2,950	2,960	YESLRPDTRY	0.17
B*44:03	5,775	5,785	AEIVDTVSAL	0.17
B*44:03	6,716	6,726	KESPFELEDF	0.17
B*49:01	669	679	KEIKESVQTF	0.17
B*49:01	3,951	3,961	SEFSSLPSYA	0.17
B*49:01	4,562	4,572	FVENPDILRV	0.17
B*49:01	5,775	5,785	AEIVDTVSAL	0.17
B*49:01	6,497	6,507	VELFENKTTL	0.17
B*51:01	2,609	2,619	VPMEKLKTLV	0.17
B*53:01	196	206	YPLECIKDLL	0.17
B*57:01	2,880	2,890	RTTNGDFLHF	0.17
B*57:01	3,135	3,145	VMFTPLVPFW	0.17
B*58:01	3,151	3,161	ICISTKHFYW	0.17
B*58:01	3,662	3,672	ASWVMRIMTW	0.17
B*58:01	6,306	6,316	KFTDGVCLFW	0.17
C*06:02	3,371	3,381	FVRIQPGQTF	0.17

C*07:01	6, 217	6, 227	VKRVDWTIEY	0. 17
C*07:02	905	915	YYLFDESGEF	0. 17
C*07:02	6, 217	6, 227	VKRVDWTIEY	0. 17
C*14:02	5, 226	5, 236	LPYPDPSRIL	0. 17
A*01:01	567	577	ITILDGISQY	0. 18
A*01:01	1, 916	1, 926	PIDLVPNQPY	0. 18
A*01:01	2, 297	2, 307	LSGLDSLDTY	0. 18
A*02:02	1, 473	1, 483	SLKVPATVSV	0. 18
A*02:02	1, 625	1, 635	VLPNDDTLRV	0. 18
A*02:06	4, 562	4, 572	FVENPDILRV	0. 18
A*02:06	7, 068	7, 078	GQINDMILSL	0. 18
A*11:01	2, 605	2, 615	STFNVPMEKL	0. 18
A*11:01	5, 168	5, 178	ASQGLVASIK	0. 18
A*23:01	1, 924	1, 934	PYPNASFDNF	0. 18
A*23:01	2, 594	2, 604	KMFDAYVNTF	0. 18
A*23:01	4, 816	4, 826	FYDFAVSKGF	0. 18
A*24:02	4, 816	4, 826	FYDFAVSKGF	0. 18
A*24:02	5, 115	5, 125	KYVRNLQHRL	0. 18
A*25:01	1, 210	1, 220	EIPKEEVKPF	0. 18
A*25:01	2, 359	2, 369	FAVHFISNSW	0. 18
A*25:01	6, 007	6, 017	EEAIRHVRAW	0. 18
A*26:01	1, 642	1, 652	TTDPSFLGRY	0. 18
A*26:01	5, 158	5, 168	DAVVCFNSTY	0. 18
A*29:02	5, 537	5, 547	AVVYRGTTTY	0. 18
A*32:01	2, 776	2, 786	KQLIKVTLVF	0. 18
A*32:01	2, 880	2, 890	RTTNGDFLHF	0. 18
A*68:02	553	563	ETAQNSVRVL	0. 18
A*68:02	1, 542	1, 552	YTSNPTTFHL	0. 18
A*68:02	5, 362	5, 372	HVISTSHKLV	0. 18
B*13:02	1, 817	1, 827	AQYELKHGTF	0. 18
B*15:01	6, 455	6, 465	RLQSLENVAF	0. 18
B*18:01	669	679	KEIKESVQTF	0. 18
B*35:01	2, 941	2, 951	TNVLEGSVAY	0. 18
B*35:01	6, 271	6, 281	VPQADVEWKF	0. 18
B*35:01	6, 673	6, 683	LAMDEFIERY	0. 18
B*35:03	61	71	LPQLEQPYVF	0. 18
B*37:01	4, 107	4, 117	VDADSKIVQL	0. 18
B*39:01	44	54	QHLKDGTCGL	0. 18
B*40:01	242	252	TERSEKSYEL	0. 18
B*40:02	6, 224	6, 234	IEYPIIGDEL	0. 18
B*44:02	231	241	CREHEHEIAW	0. 18
B*44:02	3, 937	3, 947	EEMLDNRATL	0. 18
B*44:02	6, 716	6, 726	KESPFELEDF	0. 18

B*44:03	1, 370	1, 380	QEILGTVSWN	0. 18
B*57:01	2, 228	2, 238	FSKLINIIIW	0. 18
C*14:02	2, 598	2, 608	AYVNTFSSTF	0. 18
C*15:02	2, 605	2, 615	STFNVPMEKL	0. 18
C*16:01	5, 143	5, 153	YAYLRKHFSM	0. 18
C*17:01	5, 725	5, 735	YVYIGDPAQL	0. 18
C*17:03	5, 725	5, 735	YVYIGDPAQL	0. 18
A*01:01	591	601	ATNNLVVMAY	0. 19
A*01:01	1, 730	1, 740	LGDVRETMSY	0. 19
A*01:01	1, 832	1, 842	YTGNYQCGHY	0. 19
A*01:01	2, 509	2, 519	YFDKAGQKTY	0. 19
A*02:01	445	455	GLNDNLLEIL	0. 19
A*03:01	2, 794	2, 804	LIIPVHVMSK	0. 19
A*03:01	5, 509	5, 519	YVFTGYRVTK	0. 19
A*11:01	281	291	IKTIQPRVEK	0. 19
A*11:01	625	635	KPVLDWLEEK	0. 19
A*11:01	5, 509	5, 519	YVFTGYRVTK	0. 19
A*11:01	6, 628	6, 638	AVKTQFNYYK	0. 19
A*23:01	1, 975	1, 985	IDYKHYTPSF	0. 19
A*23:01	5, 115	5, 125	KYVRNLQHRL	0. 19
A*24:02	599	609	AYITGGVVQL	0. 19
A*24:02	1, 975	1, 985	IDYKHYTPSF	0. 19
A*24:02	3, 705	3, 715	VYDDGARRVW	0. 19
A*25:01	2, 669	2, 679	EVTGDSCNNY	0. 19
A*25:01	2, 975	2, 985	EGSVRVVTTF	0. 19
A*25:01	3, 911	3, 921	EAFEKMVSLL	0. 19
A*25:01	4, 868	4, 878	FVVEVVDKYF	0. 19
A*25:01	6, 148	6, 158	STASDTYACW	0. 19
A*26:01	1, 027	1, 037	TIEVNSFSGY	0. 19
A*26:01	4, 087	4, 097	NTCDGTTFTY	0. 19
A*26:01	5, 385	5, 395	DVTDVTQLYL	0. 19
A*26:01	5, 537	5, 547	AVVYRGTTTY	0. 19
A*29:02	1, 594	1, 604	TYGQQFGPTY	0. 19
A*31:01	110	120	HVGEIPVAYR	0. 19
A*31:01	3, 658	3, 668	VYMPASWVMR	0. 19
A*33:01	223	233	DTKRGVYCCR	0. 19
A*33:01	3, 557	3, 567	DEFTPFDVVR	0. 19
A*68:01	4, 737	4, 747	FVVSTGYHFR	0. 19
A*68:02	3, 704	3, 714	TVYDDGARRV	0. 19
B*07:02	6, 567	6, 577	KPTETICAPL	0. 19
B*07:02	6, 661	6, 671	KPRSQMEIDF	0. 19
B*13:02	4, 477	4, 487	YQHEETIYNL	0. 19
B*14:02	5, 361	5, 371	DHVISTSHKL	0. 19

B*15:01	173	183	LMRELNGGAY	0. 19
B*15:01	5, 994	6, 004	YQVNGYPNMF	0. 19
B*15:03	3, 950	3, 960	ASEFSSLPSY	0. 19
B*15:03	5, 618	5, 628	SHFAIGLALY	0. 19
B*15:03	5, 675	5, 685	FKVNSTLEQY	0. 19
B*18:01	912	922	GEFKLASHMY	0. 19
B*18:01	5, 702	5, 712	FDEISMATNY	0. 19
B*27:05	541	551	ARVVRSIFSR	0. 19
B*27:05	3, 489	3, 499	NRFTTTLNDF	0. 19
B*27:05	6, 217	6, 227	VKRVDWTIEY	0. 19
B*35:03	18	28	LPVLQVRDVL	0. 19
B*35:03	1, 663	1, 673	YPQVNGLTSI	0. 19
B*38:01	1, 385	1, 395	AHAEETRKLM	0. 19
B*40:01	2, 651	2, 661	VETKDVVECL	0. 19
B*40:02	2, 865	2, 875	REVGFVVPGL	0. 19
B*40:02	4, 263	4, 273	TEVPANSTVL	0. 19
B*44:02	5, 775	5, 785	AEIVDTVSAL	0. 19
B*44:03	4, 674	4, 684	EERLKLFDRY	0. 19
B*44:03	6, 975	6, 985	TEHSWNADLY	0. 19
B*46:01	1, 878	1, 888	YTTTIKPVTY	0. 19
B*46:01	4, 265	4, 275	VPANSTVLSF	0. 19
B*49:01	656	666	ACEIVGGQIV	0. 19
B*51:01	6, 226	6, 236	YPIIGDELKI	0. 19
B*53:01	3, 477	3, 487	YAAVINGDRW	0. 19
B*53:01	6, 226	6, 236	YPIIGDELKI	0. 19
B*57:01	134	144	HSYGADLKSF	0. 19
B*57:01	3, 262	3, 272	ITSAVLQSGF	0. 19
B*57:01	4, 189	4, 199	LLSDLQDLKW	0. 19
B*57:01	6, 306	6, 316	KFTDGVCLFW	0. 19
C*02:02	404	414	IAFGGCVFSY	0. 19
C*04:01	1, 182	1, 192	VFDKNLYDKL	0. 19
C*04:43	1, 182	1, 192	VFDKNLYDKL	0. 19
C*06:02	3, 825	3, 835	FRYMNSQGLL	0. 19
C*14:02	2, 440	2, 450	YVYANGGKGF	0. 19
C*14:02	3, 067	3, 077	YYFMRFRRAF	0. 19
C*17:01	1, 542	1, 552	YTSNPTTFHL	0. 19
C*17:03	1, 542	1, 552	YTSNPTTFHL	0. 19
A*02:01	468	478	KLNEEIAIIL	0. 2
A*02:05	874	884	VVADAVIKTL	0. 2
A*02:05	4, 519	4, 529	YTMADLVYAL	0. 2
A*02:05	4, 562	4, 572	FVENPDILRV	0. 2
A*03:01	6, 482	6, 492	SIINNTVYTK	0. 2
A*23:01	181	191	AYTRYVDNNF	0. 2

A*23:01	599	609	AYITGGVVQL	0. 2
A*23:01	3, 014	3, 024	DYYRSLPGVF	0. 2
A*23:01	6, 440	6, 450	VYKQFDTYNL	0. 2
A*24:02	3, 393	3, 403	VYQCAMRPNF	0. 2
A*24:02	5, 539	5, 549	VYRGTTTYKL	0. 2
A*25:01	5, 303	5, 313	MLTNDNTSRY	0. 2
A*26:01	88	98	LVAELEGIQY	0. 2
A*26:01	2, 556	2, 566	ESSAKSASVY	0. 2
A*26:01	5, 391	5, 401	QLYLGGMSYY	0. 2
A*29:02	3, 246	3, 256	FSNSGSDVLY	0. 2
A*29:02	6, 118	6, 128	HGFELTSMKY	0. 2
A*32:01	1, 665	1, 675	QVNGLTSIKW	0. 2
A*32:01	3, 135	3, 145	VMFTPLVPFW	0. 2
A*32:01	6, 430	6, 440	MMISAGFSLW	0. 2
A*33:01	6, 672	6, 682	ELAMDEFIER	0. 2
A*68:01	6, 460	6, 470	ENVAFNVVNK	0. 2
A*68:01	6, 509	6, 519	NVAFELWAKR	0. 2
A*68:02	2, 605	2, 615	STFNVPMEKL	0. 2
B*07:02	5, 502	5, 512	RPPLNRNYVF	0. 2
B*13:02	2, 377	2, 387	VQMAPISAMV	0. 2
B*14:02	5, 403	5, 413	SHKPPISFPL	0. 2
B*18:01	940	950	EFEPSTQYEY	0. 2
B*18:01	6, 666	6, 676	MEIDFLELAM	0. 2
B*35:03	3, 389	3, 399	SPSGVYQCAM	0. 2
B*35:03	4, 201	4, 211	FPKSDGTGTI	0. 2
B*35:03	5, 269	5, 279	HPNQEYADVF	0. 2
B*35:03	6, 226	6, 236	YPIIGDELKI	0. 2
B*37:01	6, 497	6, 507	VELFENKTTL	0. 2
B*38:01	2, 798	2, 808	VHVMSKHTDF	0. 2
B*40:01	3, 993	4, 003	KSEFDRDAAM	0. 2
B*44:03	231	241	CREHEHEIAW	0. 2
B*44:03	3, 937	3, 947	EEMLDNRATL	0. 2
B*44:03	4, 918	4, 928	YEDQDALFAY	0. 2
B*44:03	7, 005	7, 015	SEAFLIGCNY	0. 2
B*45:01	3, 965	3, 975	AQEAYEQAVA	0. 2
B*46:01	905	915	YYLFDESGEF	0. 2
B*46:01	4, 076	4, 086	MVVIPDYNTY	0. 2
B*51:01	5, 841	5, 851	ISPYNSQNAV	0. 2
B*57:01	4, 762	4, 772	RLSFKELLVY	0. 2
B*58:01	1, 482	1, 492	VSSPDAVTAY	0. 2
B*58:01	1, 665	1, 675	QVNGLTSIKW	0. 2
B*58:01	3, 730	3, 740	NALDQAISMW	0. 2
B*58:01	4, 762	4, 772	RLSFKELLVY	0. 2

C*01:02	2,608	2,618	NVPMEKLKTL	0.2
C*01:02	4,507	4,517	MVPHISRQRL	0.2
C*01:02	6,067	6,077	SAKPPPGDQF	0.2
C*01:02	6,395	6,405	YVPLKSATCI	0.2
C*02:02	1,878	1,888	YTTTIKPVTY	0.2
C*02:02	6,298	6,308	YSYATHSDKF	0.2
C*08:01	6,054	6,064	YVDTPDNTDF	0.2
C*12:02	404	414	IAFGGCVFSY	0.2
A*01:01	1,419	1,429	GVVDYGARFY	0.21
A*01:01	2,556	2,566	ESSAKSASVY	0.21
A*01:01	3,442	3,452	GTDLEGNFYG	0.21
A*02:01	1,473	1,483	SLKVPATVSV	0.21
A*02:01	5,369	5,379	KLVLSVNPYV	0.21
A*02:05	2,869	2,879	FVVPGLPGTI	0.21
A*03:01	3,359	3,369	VDTANPKTPK	0.21
A*11:01	2,074	2,084	TEVVGDIILK	0.21
A*11:01	5,106	5,116	STDGNKIADK	0.21
A*23:01	6,298	6,308	YSYATHSDKF	0.21
A*23:01	6,780	6,790	DYTEISFMLW	0.21
A*24:02	953	963	DDYQGKPLEF	0.21
A*24:02	1,186	1,196	NLYDKLVSSF	0.21
A*24:02	5,725	5,735	YVYIGDPAQL	0.21
A*24:02	6,780	6,790	DYTEISFMLW	0.21
A*25:01	5,465	5,475	ETLKATEETF	0.21
A*26:01	1,419	1,429	GVVDYGARFY	0.21
A*26:01	1,508	1,518	ETISLAGSYK	0.21
A*26:01	2,141	2,151	DTIANYAKPF	0.21
A*26:01	3,740	3,750	ALIISVTSNY	0.21
A*26:01	4,653	4,663	HVDTDLTKPY	0.21
A*29:02	708	718	TFVTHSKGLY	0.21
A*29:02	1,179	1,189	YLAVFDKNLY	0.21
A*29:02	1,333	1,343	TYPGQGLNGY	0.21
A*32:01	1,817	1,827	AQYELKHGTF	0.21
A*32:01	2,960	2,970	VLMDGSIIQF	0.21
A*32:01	4,762	4,772	RLSFKELLVY	0.21
A*33:01	4,520	4,530	TMADLVYALR	0.21
A*33:01	5,997	6,007	NGYPNMFITR	0.21
A*68:01	252	262	QTPFEIKLAK	0.21
A*68:01	6,880	6,890	GVAPGTAVLR	0.21
B*13:02	4,864	4,874	RQLLFVVEVV	0.21
B*13:02	6,699	6,709	HSQLGGLHLL	0.21
B*15:01	88	98	LVAELEGIQY	0.21
B*15:01	525	535	EQKSILSPLY	0.21

B*15:01	2,979	2,989	RVVTTFDSEY	0.21
B*15:01	3,960	3,970	AAFATAQEAY	0.21
B*18:01	232	242	REHEHEIAWY	0.21
B*35:03	3,276	3,286	FPSGKVEGCM	0.21
B*35:03	4,265	4,275	VPANSTVLSF	0.21
B*35:03	4,341	4,351	HPNPKGFCDL	0.21
B*35:03	5,948	5,958	LHPTQAPTHL	0.21
B*35:03	6,322	6,332	YPANSIVCRF	0.21
B*38:01	1,145	1,155	QHEVLLAPLL	0.21
B*38:01	1,384	1,394	LAHAEETRKL	0.21
B*38:01	4,705	4,715	LHCANFNVLF	0.21
B*40:01	1,691	1,701	IELKFNPPAL	0.21
B*46:01	4,276	4,286	AFAVDAAKAY	0.21
B*58:01	134	144	HSYGADLKSF	0.21
B*58:01	3,987	3,997	KSLNVAKSEF	0.21
C*01:02	6,547	6,557	RDAPAHISTI	0.21
C*02:02	3,246	3,256	FSNSGSDVLY	0.21
C*03:04	3,371	3,381	FVRIQPGQTF	0.21
C*03:04	6,805	6,815	SQAWQPGVAM	0.21
C*04:01	2,594	2,604	KMFDAYVNTF	0.21
C*04:01	3,670	3,680	TWLDMVDTSL	0.21
C*04:01	6,054	6,064	YVDTPDNTDF	0.21
C*04:43	2,594	2,604	KMFDAYVNTF	0.21
C*04:43	3,670	3,680	TWLDMVDTSL	0.21
C*04:43	6,054	6,064	YVDTPDNTDF	0.21
C*08:02	586	596	FTSDLATNNL	0.21
C*14:02	5,725	5,735	YVYIGDPAQL	0.21
C*17:01	1,812	1,822	MSAPPAQYEL	0.21
C*17:03	1,812	1,822	MSAPPAQYEL	0.21
A*02:01	3,644	3,654	FLLPSLATVA	0.22
A*02:02	1,441	1,451	NTLNDLNETL	0.22
A*02:02	2,469	2,479	FISDEVARDL	0.22
A*03:01	3,528	3,538	AVLDMCASLK	0.22
A*03:01	4,077	4,087	VVIPDYNTYK	0.22
A*03:01	6,518	6,528	RNIKPVPEVK	0.22
A*11:01	807	817	MVTNNTFTLK	0.22
A*11:01	1,193	1,203	SSFLEMKSEK	0.22
A*23:01	2,517	2,527	TYERHSLSHF	0.22
A*23:01	3,067	3,077	YYFMRFRRAF	0.22
A*23:01	3,393	3,403	VYQCAMRPNF	0.22
A*23:01	3,688	3,698	VMYASAVVLL	0.22
A*23:01	5,725	5,735	YVYIGDPAQL	0.22
A*24:02	6,864	6,874	VPYNMRVIHF	0.22

A*26:01	5, 675	5, 685	FKVNSTLEQY	0. 22
A*29:02	2, 335	2, 345	AYILFTRFFY	0. 22
A*29:02	2, 805	2, 815	TDFSSEIIGY	0. 22
A*29:02	3, 207	3, 217	TQYNRYLALY	0. 22
A*31:01	2, 111	2, 121	TIKKPNELSR	0. 22
A*68:01	1, 400	1, 410	TKAIVSTIQR	0. 22
A*68:01	4, 896	4, 906	DKSAGFPFNK	0. 22
A*68:01	5, 509	5, 519	YVFTGYRVTK	0. 22
A*68:02	707	717	ETFVTHSKGL	0. 22
A*68:02	1, 008	1, 018	QTIVEVQPQL	0. 22
A*68:02	1, 735	1, 745	ETMSYLFQHA	0. 22
B*07:02	2, 608	2, 618	NVPMEKLKTL	0. 22
B*07:02	2, 870	2, 880	VVPGLPGTIL	0. 22
B*07:02	4, 201	4, 211	FPKSDGTGTI	0. 22
B*07:02	7, 038	7, 048	NPIQLSSYSL	0. 22
B*13:02	1, 663	1, 673	YPQVNGLTSI	0. 22
B*15:01	478	488	ASFSASTSAF	0. 22
B*15:01	2, 258	2, 268	LMSNLGMPSY	0. 22
B*15:01	2, 964	2, 974	GSIIQFPNTY	0. 22
B*15:01	3, 950	3, 960	ASEFSSLPSY	0. 22
B*15:01	4, 076	4, 086	MVVIPDYNTY	0. 22
B*15:01	6, 641	6, 651	GVVQQLPETY	0. 22
B*35:01	3, 960	3, 970	AAFATAQEAY	0. 22
B*35:03	5, 225	5, 235	YLPYPDPSRI	0. 22
B*40:02	1, 781	1, 791	YEQFKKGVQI	0. 22
B*44:03	790	800	LLEIKDTEKY	0. 22
B*45:01	2, 093	2, 103	EEVGHTDLMA	0. 22
B*46:01	2, 557	2, 567	SSAKSASVYY	0. 22
B*46:01	4, 714	4, 724	FSTVFPLTSF	0. 22
B*46:01	6, 170	6, 180	FMIDVQQWGF	0. 22
B*49:01	538	548	SEAARVVRSI	0. 22
B*49:01	1, 873	1, 883	YKENSYTTTI	0. 22
B*51:01	1, 211	1, 221	IPKEEVKPFI	0. 22
B*57:01	2, 317	2, 327	SSFKWDLTAF	0. 22
B*57:01	2, 539	2, 549	GSLPINVIVF	0. 22
B*57:01	3, 596	3, 606	LLVLVQSTQW	0. 22
B*57:01	6, 289	6, 299	KAYKIEELFY	0. 22
C*02:02	2, 557	2, 567	SSAKSASVYY	0. 22
C*03:02	1, 482	1, 492	VSSPDAVTAY	0. 22
C*03:02	3, 953	3, 963	FSSLPSYAAF	0. 22
C*03:02	6, 673	6, 683	LAMDEFIERY	0. 22
C*04:01	1, 186	1, 196	NLYDKLVSSF	0. 22
C*04:01	2, 103	2, 113	AYVDNSSLTI	0. 22

C*04:01	6,330	6,340	RFDTRVLSNL	0.22
C*04:43	1,186	1,196	NLYDKLVSSF	0.22
C*04:43	2,103	2,113	AYVDNSSLTI	0.22
C*04:43	6,330	6,340	RFDTRVLSNL	0.22
C*05:01	1,603	1,613	YLDGADVTKI	0.22
C*08:02	4,008	4,018	KMADQAMTQM	0.22
C*14:02	599	609	AYITGGVVQL	0.22
C*14:02	1,594	1,604	TYGQQFGPTY	0.22
C*14:02	4,465	4,475	SYFVVKRHTF	0.22
C*14:02	5,553	5,563	YFVLTSHTVM	0.22
C*14:02	6,693	6,703	VYGDFSHSQL	0.22
A*01:01	6,824	6,834	LLEKCDLQNY	0.23
A*02:01	843	853	FELDERIDKV	0.23
A*02:01	1,625	1,635	VLPNDDTLRV	0.23
A*02:02	6,105	6,115	KNLSDRVVFV	0.23
A*02:06	3,103	3,113	YSFLPGVYSV	0.23
A*02:06	6,700	6,710	SQLGGLHLLI	0.23
A*03:01	5,537	5,547	AVVYRGTTTY	0.23
A*11:01	2,493	2,503	SYIVDSVTVK	0.23
A*11:01	2,826	2,836	ASTDTCFANK	0.23
A*23:01	3,705	3,715	VYDDGARRVW	0.23
A*23:01	4,722	4,732	SFGPLVRKIF	0.23
A*23:01	5,272	5,282	QEYADVFHLY	0.23
A*24:02	2,362	2,372	HFISNSWLMW	0.23
A*24:02	2,594	2,604	KMFDAYVNTF	0.23
A*24:02	3,014	3,024	DYYRSLPGVF	0.23
A*26:01	1,869	1,879	TDVFYKENSY	0.23
A*26:01	4,868	4,878	FVVEVVDKYF	0.23
A*29:02	2,784	2,794	VFLFVAAIFY	0.23
A*29:02	4,087	4,097	NTCDGTTFTY	0.23
A*29:02	4,909	4,919	ARLYYDSMSY	0.23
A*29:02	5,116	5,126	YVRNLQHRLY	0.23
A*29:02	5,303	5,313	MLTNDNTSRY	0.23
A*33:01	1,641	1,651	HTTDPSFLGR	0.23
A*68:02	1,812	1,822	MSAPPAQYEL	0.23
A*68:02	2,461	2,471	DTFCAGSTFI	0.23
A*68:02	6,648	6,658	ETYFTQSRNL	0.23
B*07:02	114	124	IPVAYRKVLL	0.23
B*07:02	5,500	5,510	KPRPPLNRNY	0.23
B*07:02	7,053	7,063	FPLKLRGTAV	0.23
B*13:02	3,373	3,383	RIQPGQTFSV	0.23
B*13:02	6,475	6,485	QQGEVPVSII	0.23
B*15:01	1,035	1,045	GYLKLTDNVY	0.23

B*15:01	2,941	2,951	TNVLEGSVAY	0.23
B*15:03	5,520	5,530	SKVQIGEYTF	0.23
B*27:05	2,701	2,711	ARHINAQVAK	0.23
B*35:01	1,696	1,706	NPPALQDAYY	0.23
B*35:01	4,076	4,086	MVVIPDYNTY	0.23
B*40:01	2,471	2,481	SDEVARDLSL	0.23
B*45:01	773	783	AVEAPLVGTP	0.23
B*45:01	2,908	2,918	IEYTDFATSA	0.23
B*46:01	1,532	1,542	FLKRGDKSVY	0.23
B*49:01	843	853	FELDERIDKV	0.23
B*49:01	2,272	2,282	REGYLNSTNV	0.23
B*53:01	360	370	LPQNAVVKIY	0.23
B*57:01	7,025	7,035	YVMHANYIFW	0.23
C*01:02	3,374	3,384	IQPGQTFSVL	0.23
C*03:02	3,960	3,970	AAFATAQEAY	0.23
C*04:01	5,003	5,013	YSDVENPHLM	0.23
C*04:43	5,003	5,013	YSDVENPHLM	0.23
C*05:01	586	596	FTSDLATNNL	0.23
C*05:01	1,160	1,170	GADPIHSLRV	0.23
C*07:01	5,500	5,510	KPRPPLNRNY	0.23
C*07:04	7,033	7,043	FWRNTNPIQL	0.23
C*12:02	567	577	ITILDGISQY	0.23
C*14:02	1,333	1,343	TYPGQGLNGY	0.23
C*14:02	3,171	3,181	VFNGVSFSTF	0.23
A*01:01	1,027	1,037	TIEVNSFSGY	0.24
A*01:01	2,500	2,510	TVKNGSIHLY	0.24
A*01:01	2,673	2,683	DSCNNYMLTY	0.24
A*01:01	2,950	2,960	YESLRPDTRY	0.24
A*01:01	3,818	3,828	YLVSTQEFRY	0.24
A*01:01	6,432	6,442	ISAGFSLWVY	0.24
A*01:01	6,726	6,736	IPMDSTVKNY	0.24
A*02:01	6,105	6,115	KNLSDRVVFV	0.24
A*02:06	5,853	5,863	KILGLPTQTV	0.24
A*23:01	899	909	EWSMATYYLF	0.24
A*24:02	6,779	6,789	IDYTEISFML	0.24
A*25:01	22	32	QVRDVLVRGF	0.24
A*25:01	827	837	DDTVIEVQGY	0.24
A*25:01	5,062	5,072	EMVMCGGSLY	0.24
A*25:01	5,361	5,371	DHVISTSHKL	0.24
A*26:01	5,544	5,554	TTYKLNVGDY	0.24
A*26:01	5,619	5,629	HFAIGLALYY	0.24
A*29:02	2,941	2,951	TNVLEGSVAY	0.24
A*29:02	5,777	5,787	IVDTVSALVY	0.24

A*68:01	2,806	2,816	DFSSEIIGYK	0.24
A*68:01	4,720	4,730	LTSFGPLVRK	0.24
A*68:01	6,056	6,066	DTPDNTDFSR	0.24
A*68:02	3,051	3,061	SIVAGGIVAI	0.24
B*15:01	3,564	3,574	VVRQCSGVTF	0.24
B*15:03	3,371	3,381	FVRIQPGQTF	0.24
B*18:01	2,950	2,960	YESLRPDTRY	0.24
B*18:01	5,290	5,300	DELTGHMLDM	0.24
B*35:01	2,380	2,390	APISAMVRMY	0.24
B*35:01	3,834	3,844	LPPKNSIDAF	0.24
B*35:01	6,809	6,819	QPGVAMPNLY	0.24
B*38:01	6,793	6,803	GHVETFYPKL	0.24
B*39:01	3,079	3,089	YSHVVAFNTL	0.24
B*40:01	2,024	2,034	VETSNSFDVL	0.24
B*44:02	4,674	4,684	EERLKLFDRY	0.24
B*44:02	6,975	6,985	TEHSWNADLY	0.24
B*44:02	7,005	7,015	SEAFLIGCNY	0.24
B*44:03	4,211	4,221	YTELEPPCRF	0.24
B*45:01	2,589	2,599	AEVAVKMFDA	0.24
B*49:01	774	784	VEAPLVGTPV	0.24
B*49:01	6,224	6,234	IEYPIIGDEL	0.24
B*51:01	5,654	5,664	LPIDKCSRII	0.24
B*53:01	275	285	FPLNSIIKTI	0.24
B*53:01	6,080	6,090	IPLMYKGLPW	0.24
B*57:01	881	891	KTLQPVSELL	0.24
B*57:01	3,730	3,740	NALDQAISMW	0.24
B*58:01	2,317	2,327	SSFKWDLTAF	0.24
B*58:01	7,025	7,035	YVMHANYIFW	0.24
C*01:02	1,813	1,823	SAPPAQYELK	0.24
C*03:02	6,067	6,077	SAKPPPGDQF	0.24
C*03:04	5,003	5,013	YSDVENPHLM	0.24
C*04:01	3,704	3,714	TVYDDGARRV	0.24
C*04:43	3,704	3,714	TVYDDGARRV	0.24
C*07:02	2,952	2,962	SLRPDTRYVL	0.24
C*07:02	5,500	5,510	KPRPPLNRNY	0.24
A*01:01	4,871	4,881	EVVDKYFDCY	0.25
A*02:01	3,643	3,653	LFLLPSLATV	0.25
A*02:02	4,477	4,487	YQHEETIYNL	0.25
A*02:06	1,207	1,217	KIAEIPKEEV	0.25
A*03:01	252	262	QTPFEIKLAK	0.25
A*03:01	3,264	3,274	SAVLQSGFRK	0.25
A*11:01	1,772	1,782	AVMYMGTLSY	0.25
A*11:01	4,779	4,789	AASGNLLLDK	0.25

A*11:01	6, 880	6, 890	GVAPGTAVLR	0. 25
A*23:01	953	963	DDYQGKPLEF	0. 25
A*23:01	1, 186	1, 196	NLYDKLVSSF	0. 25
A*23:01	2, 390	2, 400	IFFASFYYVW	0. 25
A*23:01	2, 637	2, 647	TFISAARQGF	0. 25
A*23:01	4, 233	4, 243	LYFIKGLNNL	0. 25
A*23:01	4, 682	4, 692	RYFKYWDQTY	0. 25
A*23:01	6, 779	6, 789	IDYTEISFML	0. 25
A*24:02	1, 541	1, 551	YYTSNPTTFH	0. 25
A*24:02	5, 272	5, 282	QEYADVFHLY	0. 25
A*25:01	1, 878	1, 888	YTTTIKPVTY	0. 25
A*25:01	3, 730	3, 740	NALDQAISMW	0. 25
A*26:01	1, 402	1, 412	AIVSTIQRKY	0. 25
A*26:01	4, 867	4, 877	LFVVEVVDKY	0. 25
A*29:02	2, 841	2, 851	TWFSQRGGSY	0. 25
A*29:02	3, 212	3, 222	YLALYNKYKY	0. 25
A*29:02	4, 076	4, 086	MVVIPDYNTY	0. 25
A*31:01	1, 949	1, 959	LTGYKKPASR	0. 25
A*32:01	1, 988	1, 998	AKLLHKPIVW	0. 25
A*32:01	2, 317	2, 327	SSFKWDLTAF	0. 25
A*33:01	4, 191	4, 201	SDLQDLKWAR	0. 25
A*68:01	1, 159	1, 169	FGADPIHSLR	0. 25
A*68:01	2, 074	2, 084	TEVVGDIILK	0. 25
A*68:01	4, 805	4, 815	QTVKPGNFNK	0. 25
B*13:02	3, 253	3, 263	VLYQPPQTSI	0. 25
B*15:01	851	861	KVLNEKCSAY	0. 25
B*15:01	4, 395	4, 405	MLQSADAQSF	0. 25
B*15:01	5, 303	5, 313	MLTNDNTSRY	0. 25
B*15:03	4, 909	4, 919	ARLYYDSMSY	0. 25
B*18:01	898	908	DEWSMATYYL	0. 25
B*27:05	5, 576	5, 586	VRITGLYPTL	0. 25
B*35:01	3, 105	3, 115	FLPGVYSVIY	0. 25
B*35:03	6, 520	6, 530	IKPVPEVKIL	0. 25
B*37:01	3, 910	3, 920	TEAFEKMVSL	0. 25
B*38:01	2, 803	2, 813	KHTDFSSEII	0. 25
B*40:01	951	961	TEDDYQGKPL	0. 25
B*40:01	1, 503	1, 513	EEHFIETISL	0. 25
B*40:01	1, 781	1, 791	YEQFKKGVQI	0. 25
B*40:01	3, 937	3, 947	EEMLDNRATL	0. 25
B*40:02	1, 691	1, 701	IELKFNPPAL	0. 25
B*40:02	3, 993	4, 003	KSEFDRDAAM	0. 25
B*44:02	5, 525	5, 535	GEYTFEKGDY	0. 25
B*44:02	6, 213	6, 223	HECFVKRVDW	0. 25

B*44:03	940	950	EFEPSTQYEY	0.25
B*44:03	1,027	1,037	TIEVNSFSGY	0.25
B*46:01	478	488	ASFSASTSAF	0.25
B*57:01	676	686	QTFFKLVNKF	0.25
B*57:01	3,477	3,487	YAAVINGDRW	0.25
B*57:01	4,830	4,840	SSVELKHFFF	0.25
B*58:01	6,430	6,440	MMISAGFSLW	0.25
C*03:02	1,878	1,888	YTTTIKPVTY	0.25
C*03:02	4,276	4,286	AFAVDAAKAY	0.25
C*03:04	5,143	5,153	YAYLRKHFSM	0.25
C*04:01	1,549	1,559	FHLDGEVITF	0.25
C*04:43	1,549	1,559	FHLDGEVITF	0.25
C*07:02	1,549	1,559	FHLDGEVITF	0.25
C*14:02	1,903	1,913	NYYKKDNSYF	0.25
A*01:01	2,277	2,287	NSTNVTIATY	0.26
A*02:01	984	994	WLDDDSQQTV	0.26
A*02:01	1,207	1,217	KIAEIPKEEV	0.26
A*02:01	1,603	1,613	YLDGADVTKI	0.26
A*02:01	1,801	1,811	YLVQQESPFV	0.26
A*02:01	6,113	6,123	FVLWAHGFEL	0.26
A*02:01	6,250	6,260	ALLADKFPVL	0.26
A*02:02	6,250	6,260	ALLADKFPVL	0.26
A*02:05	1,275	1,285	LVSDIDITFL	0.26
A*02:05	3,052	3,062	IVAGGIVAIV	0.26
A*02:05	5,928	5,938	TLQAENVTGL	0.26
A*03:01	38	48	VLSEARQHLK	0.26
A*03:01	807	817	MVTNNTFTLK	0.26
A*03:01	5,802	5,812	KMFYKGVIMH	0.26
A*11:01	1,343	1,353	TVEEAKTVLK	0.26
A*11:01	1,392	1,402	KLMPVCVETK	0.26
A*11:01	2,311	2,321	TIQITISSFK	0.26
A*11:01	3,570	3,580	GVTFQSAVKR	0.26
A*23:01	5,539	5,549	VYRGTTTYKL	0.26
A*23:01	6,365	6,375	AFVNLKQLPF	0.26
A*23:01	6,864	6,874	VPYNMRVIHF	0.26
A*24:02	248	258	SYELQTPFEI	0.26
A*24:02	4,233	4,243	LYFIKGLNNL	0.26
A*25:01	2,941	2,951	TNVLEGSVAY	0.26
A*25:01	4,092	4,102	TTFTYASALW	0.26
A*25:01	4,714	4,724	FSTVFPLTSF	0.26
A*25:01	5,304	5,314	LTNDNTSRYW	0.26
A*25:01	5,537	5,547	AVVYRGTTTY	0.26
A*26:01	5,861	5,871	TVDSSQGSEY	0.26

A*29:02	591	601	ATNNLVVMAY	0.26
A*29:02	4,677	4,687	LKLFDRYFKY	0.26
A*31:01	3,703	3,713	RTVYDDGARR	0.26
A*32:01	4,580	4,590	RQALLKTVQF	0.26
A*33:01	2,987	2,997	EYCRHGTCER	0.26
A*33:01	5,114	5,124	DKYVRNLQHR	0.26
A*68:01	1,243	1,253	EVTTTLEETK	0.26
B*13:02	95	105	IQYGRSGETL	0.26
B*13:02	1,862	1,872	SEYKGPITDV	0.26
B*15:01	3,715	3,725	TLMNVLTLVY	0.26
B*15:03	4,728	4,738	RKIFVDGVPF	0.26
B*35:03	79	89	APHGHVMVEL	0.26
B*44:03	6,119	6,129	GFELTSMKYF	0.26
B*46:01	1,586	1,596	TQVVDMSMTY	0.26
B*46:01	6,298	6,308	YSYATHSDKF	0.26
B*49:01	1,016	1,026	QLEMELTPVV	0.26
B*49:01	5,207	5,217	HEFCSQHTML	0.26
B*51:01	359	369	YLPQNAVVKI	0.26
B*51:01	747	757	LPTEVLTEEV	0.26
B*51:01	3,138	3,148	TPLVPFWITI	0.26
B*53:01	6,864	6,874	VPYNMRVIHF	0.26
B*57:01	4,916	4,926	MSYEDQDALF	0.26
C*01:02	5,226	5,236	LPYPDPSRIL	0.26
C*03:02	567	577	ITILDGISQY	0.26
C*03:04	5,725	5,735	YVYIGDPAQL	0.26
C*07:02	76	86	ARTAPHGHVM	0.26
C*07:02	2,509	2,519	YFDKAGQKTY	0.26
C*12:03	5,143	5,153	YAYLRKHFSM	0.26
C*12:03	6,067	6,077	SAKPPPGDQF	0.26
C*15:02	1,812	1,822	MSAPPAQYEL	0.26
A*02:01	5,853	5,863	KILGLPTQTV	0.27
A*02:02	881	891	KTLQPVSELL	0.27
A*02:02	5,725	5,735	YVYIGDPAQL	0.27
A*02:05	1,473	1,483	SLKVPATVSV	0.27
A*02:06	1,295	1,305	VVQEGVLTAV	0.27
A*02:06	3,052	3,062	IVAGGIVAIV	0.27
A*11:01	614	624	TNIFGTVYEK	0.27
A*11:01	1,401	1,411	KAIVSTIQRK	0.27
A*11:01	5,171	5,181	GLVASIKNFK	0.27
A*11:01	5,714	5,724	SVVNARLRAK	0.27
A*11:01	6,433	6,443	SAGFSLWVYK	0.27
A*11:01	6,537	6,547	AANTVIWDYK	0.27
A*23:01	676	686	QTFFKLVNKF	0.27

A*23:01	1, 178	1, 188	VYLAVFDKNL	0. 27
A*23:01	2, 305	2, 315	TYPSLETIQI	0. 27
A*23:01	4, 736	4, 746	PFVVSTGYHF	0. 27
A*24:02	899	909	EWSMATYYLF	0. 27
A*25:01	55	65	EVEKGVLPQL	0. 27
A*25:01	1, 896	1, 906	EIDPKLDNYY	0. 27
A*26:01	525	535	EQKSILSPLY	0. 27
A*26:01	1, 772	1, 782	AVMYMGTLSY	0. 27
A*26:01	3, 111	3, 121	SVIYLYLTFY	0. 27
A*26:01	4, 426	4, 436	DVVYRAFDIY	0. 27
A*29:02	1, 419	1, 429	GVVDYGARFY	0. 27
A*29:02	4, 762	4, 772	RLSFKELLVY	0. 27
A*29:02	5, 136	5, 146	TDFVNEFYAY	0. 27
A*32:01	3, 662	3, 672	ASWVMRIMTW	0. 27
A*68:01	6, 354	6, 364	HAFHTPAFDK	0. 27
A*68:02	1, 012	1, 022	EVQPQLEMEL	0. 27
A*68:02	5, 361	5, 371	DHVISTSHKL	0. 27
B*07:02	6, 357	6, 367	HTPAFDKSAF	0. 27
B*07:02	6, 864	6, 874	VPYNMRVIHF	0. 27
B*15:01	905	915	YYLFDESGEF	0. 27
B*15:01	1, 482	1, 492	VSSPDAVTAY	0. 27
B*15:01	4, 756	4, 766	VNLHSSRLSF	0. 27
B*15:01	5, 216	5, 226	LVKQGDDYVY	0. 27
B*15:03	1, 772	1, 782	AVMYMGTLSY	0. 27
B*15:03	6, 291	6, 301	YKIEELFYSY	0. 27
B*35:01	5, 619	5, 629	HFAIGLALYY	0. 27
B*35:03	2, 379	2, 389	MAPISAMVRM	0. 27
B*38:01	2, 361	2, 371	VHFISNSWLM	0. 27
B*38:01	6, 470	6, 480	GHFDGQQGEV	0. 27
B*44:02	4, 918	4, 928	YEDQDALFAY	0. 27
B*46:01	1, 186	1, 196	NLYDKLVSSF	0. 27
B*46:01	2, 440	2, 450	YVYANGGKGF	0. 27
B*46:01	5, 390	5, 400	TQLYLGGMSY	0. 27
B*51:01	7, 053	7, 063	FPLKLRGTAV	0. 27
B*57:01	1, 956	1, 966	ASRELKVTFF	0. 27
B*57:01	2, 312	2, 322	IQITISSFKW	0. 27
B*57:01	3, 124	3, 134	DVSFLAHIQW	0. 27
B*57:01	6, 882	6, 892	APGTAVLRQW	0. 27
B*58:01	2, 539	2, 549	GSLPINVIVF	0. 27
C*03:02	6, 805	6, 815	SQAWQPGVAM	0. 27
C*04:01	3, 179	3, 189	TFEEAALCTF	0. 27
C*04:43	3, 179	3, 189	TFEEAALCTF	0. 27
C*05:01	1, 549	1, 559	FHLDGEVITF	0. 27

C*05:01	2,960	2,970	VLMDGSIIQF	0.27
C*05:01	4,338	4,348	HIDHPNPKGF	0.27
C*06:02	76	86	ARTAPHGHVM	0.27
C*07:02	7,033	7,043	FWRNTNPIQL	0.27
C*08:02	6,490	6,500	TKVDGVDVEL	0.27
C*16:01	6,067	6,077	SAKPPPGDQF	0.27
A*01:01	4,918	4,928	YEDQDALFAY	0.28
A*01:01	5,309	5,319	TSRYWEPEFY	0.28
A*02:01	6,754	6,764	LLLDDFVEII	0.28
A*02:02	7,046	7,056	SLFDMSKFPL	0.28
A*02:05	2,469	2,479	FISDEVARDL	0.28
A*02:05	7,068	7,078	GQINDMILSL	0.28
A*02:06	6,699	6,709	HSQLGGLHLL	0.28
A*11:01	273	283	FVFPLNSIIK	0.28
A*11:01	1,190	1,200	KLVSSFLEMK	0.28
A*11:01	3,361	3,371	TANPKTPKYK	0.28
A*11:01	3,925	3,935	SMQGAVDINK	0.28
A*24:02	2,103	2,113	AYVDNSSLTI	0.28
A*24:02	4,553	4,563	YFNKKDWYDF	0.28
A*24:02	4,722	4,732	SFGPLVRKIF	0.28
A*25:01	2,556	2,566	ESSAKSASVY	0.28
A*29:02	2,327	2,337	GLVAEWFLAY	0.28
A*29:02	3,807	3,817	RYFRLTLGVY	0.28
A*29:02	4,846	4,856	AISDYDYYRY	0.28
A*29:02	5,255	5,265	RFVSLAIDAY	0.28
A*29:02	6,432	6,442	ISAGFSLWVY	0.28
A*31:01	1,641	1,651	HTTDPSFLGR	0.28
A*31:01	5,498	5,508	VGKPRPPLNR	0.28
A*68:01	1,222	1,232	ESKPSVEQRK	0.28
A*68:01	4,506	4,516	DMVPHISRQR	0.28
A*68:02	489	499	ETVKGLDYKA	0.28
B*15:01	360	370	LPQNAVVKIY	0.28
B*15:01	1,186	1,196	NLYDKLVSSF	0.28
B*15:03	1,810	1,820	VMMSAPPAQY	0.28
B*15:03	1,842	1,852	KHITSKETLY	0.28
B*35:01	3,389	3,399	SPSGVYQCAM	0.28
B*38:01	6,352	6,362	NKHAFHTPAF	0.28
B*40:01	672	682	KESVQTFFKL	0.28
B*40:02	242	252	TERSEKSYEL	0.28
B*40:02	3,076	3,086	FGEYSHVVAF	0.28
B*44:02	939	949	EEFEPSTQYE	0.28
B*44:02	2,330	2,340	AEWFLAYILF	0.28
B*44:02	4,211	4,221	YTELEPPCRF	0.28

B*44:02	5, 589	5, 599	DEFSSNVANY	0. 28
B*44:02	6, 119	6, 129	GFELTSMKYF	0. 28
B*44:03	1, 728	1, 738	GELGDVRETM	0. 28
B*46:01	134	144	HSYGADLKSF	0. 28
B*46:01	2, 960	2, 970	VLMDGSIIQF	0. 28
B*46:01	3, 610	3, 620	FLYENAFLPF	0. 28
B*49:01	4, 263	4, 273	TEVPANSTVL	0. 28
B*51:01	731	741	MPLKAPKEII	0. 28
B*53:01	6, 341	6, 351	LPGCDGGSLY	0. 28
B*57:01	3, 348	3, 358	HSMQNCVLKF	0. 28
B*57:01	6, 430	6, 440	MMISAGFSLW	0. 28
B*58:01	881	891	KTLQPVSELL	0. 28
B*58:01	1, 686	1, 696	LTLQQIELKF	0. 28
B*58:01	3, 135	3, 145	VMFTPLVPFW	0. 28
C*01:02	359	369	YLPQNAVVKI	0. 28
C*03:02	3, 246	3, 256	FSNSGSDVLY	0. 28
C*04:01	569	579	ILDGISQYSL	0. 28
C*04:43	569	579	ILDGISQYSL	0. 28
C*05:01	4, 008	4, 018	KMADQAMTQM	0. 28
C*05:01	6, 491	6, 501	KVDGVDVELF	0. 28
C*05:01	6, 637	6, 647	KKVDGVVQQL	0. 28
C*07:01	6, 582	6, 592	GRVDGQVDLF	0. 28
C*07:02	3, 067	3, 077	YYFMRFRRAF	0. 28
C*14:02	4, 682	4, 692	RYFKYWDQTY	0. 28
C*15:02	1, 542	1, 552	YTSNPTTFHL	0. 28
A*01:01	4, 211	4, 221	YTELEPPCRF	0. 29
A*01:01	4, 537	4, 547	DTLKEILVTY	0. 29
A*02:01	7, 046	7, 056	SLFDMSKFPL	0. 29
A*02:06	1, 473	1, 483	SLKVPATVSV	0. 29
A*03:01	281	291	IKTIQPRVEK	0. 29
A*03:01	625	635	KPVLDWLEEK	0. 29
A*03:01	1, 062	1, 072	VVNAANVYLK	0. 29
A*03:01	1, 650	1, 660	RYMSALNHTK	0. 29
A*03:01	4, 805	4, 815	QTVKPGNFNK	0. 29
A*03:01	5, 171	5, 181	GLVASIKNFK	0. 29
A*03:01	5, 475	5, 485	KLSYGIATVR	0. 29
A*23:01	1, 158	1, 168	IFGADPIHSL	0. 29
A*23:01	2, 334	2, 344	LAYILFTRFF	0. 29
A*23:01	4, 553	4, 563	YFNKKDWYDF	0. 29
A*24:02	1, 639	1, 649	YYHTTDPSFL	0. 29
A*24:02	3, 067	3, 077	YYFMRFRRAF	0. 29
A*24:02	4, 176	4, 186	YYNTTKGGRF	0. 29
A*25:01	2, 430	2, 440	TIVNGVRRSF	0. 29

A*26:01	22	32	QVRDVLVRGF	0.29
A*26:01	5,116	5,126	YVRNLQHRLY	0.29
A*29:02	295	305	GFMGRIRSVY	0.29
A*29:02	2,258	2,268	LMSNLGMPSY	0.29
A*29:02	2,387	2,397	RMYIFFASFY	0.29
A*29:02	3,111	3,121	SVIYLYLTFY	0.29
A*29:02	3,150	3,160	IICISTKHFY	0.29
A*29:02	3,468	3,478	ITVNVLAWLY	0.29
A*29:02	3,960	3,970	AAFATAQEAY	0.29
A*29:02	4,276	4,286	AFAVDAAKAY	0.29
A*29:02	4,325	4,335	SFGGASCCLY	0.29
A*29:02	6,313	6,323	LFWNCNVDRY	0.29
A*31:01	5,224	5,234	VYLPYPDPSR	0.29
A*32:01	560	570	RVLQKAAITI	0.29
A*32:01	6,455	6,465	RLQSLENVAF	0.29
A*33:01	709	719	FVTHSKGLYR	0.29
A*68:01	3,676	3,686	DTSLSGFKLK	0.29
A*68:02	37	47	EVLSEARQHL	0.29
A*68:02	55	65	EVEKGVLPQL	0.29
A*68:02	2,536	2,546	NTKGSLPINV	0.29
A*68:02	4,721	4,731	TSFGPLVRKI	0.29
A*68:02	6,486	6,496	NTVYTKVDGV	0.29
B*13:02	4,256	4,266	RLQAGNATEV	0.29
B*13:02	5,853	5,863	KILGLPTQTV	0.29
B*15:01	5,175	5,185	SIKNFKSVLY	0.29
B*15:03	1,523	1,533	GQSTQLGIEF	0.29
B*15:03	3,267	3,277	LQSGFRKMAF	0.29
B*15:03	6,217	6,227	VKRVDWTIEY	0.29
B*15:03	6,889	6,899	RQWLPTGTLL	0.29
B*18:01	862	872	VELGTEVNEF	0.29
B*18:01	4,674	4,684	EERLKLFDRY	0.29
B*18:01	5,703	5,713	DEISMATNYD	0.29
B*18:01	6,975	6,985	TEHSWNADLY	0.29
B*27:05	5,718	5,728	ARLRAKHYVY	0.29
B*27:05	5,982	5,992	RRLISMMGFK	0.29
B*35:01	6,282	6,292	DAQPCSDKAY	0.29
B*38:01	3,079	3,089	YSHVVAFNTL	0.29
B*39:01	1,504	1,514	EHFIETISLA	0.29
B*39:01	5,617	5,627	KSHFAIGLAL	0.29
B*39:01	6,470	6,480	GHFDGQQGEV	0.29
B*40:01	376	386	SEVGPEHSLA	0.29
B*40:01	5,572	5,582	QEHYVRITGL	0.29
B*40:01	6,666	6,676	MEIDFLELAM	0.29

B*40:02	388	398	HNESGLKTIL	0.29
B*40:02	6,028	6,038	REAVGTNLPL	0.29
B*45:01	2,621	2,631	AEAELAKNVS	0.29
B*46:01	669	679	KEIKESVQTF	0.29
B*46:01	3,222	3,232	FSGAMDTTSY	0.29
B*51:01	2,199	2,209	MPTTIAKNTV	0.29
B*58:01	3,596	3,606	LLVLVQSTQW	0.29
C*02:02	3,960	3,970	AAFATAQEAY	0.29
C*03:02	5,143	5,153	YAYLRKHFSM	0.29
C*05:01	984	994	WLDDDSQQTV	0.29
C*05:01	2,629	2,639	VSLDNVLSTF	0.29
C*05:01	4,221	4,231	VTDTPKGPKV	0.29
C*05:01	5,154	5,164	ILSDDAVVCF	0.29
C*05:01	6,490	6,500	TKVDGVDVEL	0.29
C*07:02	2,953	2,963	LRPDTRYVLM	0.29
C*07:02	4,816	4,826	FYDFAVSKGF	0.29
C*07:02	6,713	6,723	KRFKESPFEL	0.29
A*02:01	3,297	3,307	GLWLDDVVYC	0.3
A*02:02	2,126	2,136	TLATHGLAAV	0.3
A*02:02	5,287	5,297	KLHDELTGHM	0.3
A*03:01	5,029	5,039	IMASLVLARK	0.3
A*03:01	5,714	5,724	SVVNARLRAK	0.3
A*03:01	6,628	6,638	AVKTQFNYYK	0.3
A*11:01	2,055	2,065	EVVENPTIQK	0.3
A*23:01	248	258	SYELQTPFEI	0.3
A*23:01	2,354	2,364	LFFSYFAVHF	0.3
A*23:01	3,657	3,667	MVYMPASWVM	0.3
A*24:02	2,440	2,450	YVYANGGKGF	0.3
A*24:02	3,657	3,667	MVYMPASWVM	0.3
A*24:02	6,422	6,432	RLYLDAYNMM	0.3
A*25:01	3,027	3,037	DAVNLLTNMF	0.3
A*25:01	5,385	5,395	DVTDVTQLYL	0.3
A*26:01	1,210	1,220	EIPKEEVKPF	0.3
A*29:02	59	69	GVLPQLEQPY	0.3
A*29:02	2,557	2,567	SSAKSASVYY	0.3
A*29:02	3,603	3,613	TQWSLFFFLY	0.3
A*32:01	3,579	3,589	RTIKGTHHWL	0.3
A*32:01	5,537	5,547	AVVYRGTTTY	0.3
A*68:01	34	44	SVEEVLSEAR	0.3
A*68:01	614	624	TNIFGTVYEK	0.3
A*68:01	3,570	3,580	GVTFQSAVKR	0.3
A*68:02	5,471	5,481	EETFKLSYGI	0.3
B*07:02	5,920	5,930	EIPRRNVATL	0.3

B*07:02	5, 975	5, 985	IPKDMTYRRL	0. 3
B*15:01	2, 557	2, 567	SSAKSASVYY	0. 3
B*15:03	1, 533	1, 543	LKRGDKSVYY	0. 3
B*18:01	897	907	LDEWSMATYY	0. 3
B*18:01	1, 507	1, 517	IETISLAGSY	0. 3
B*35:03	308	318	SPNECNQMCL	0. 3
B*38:01	2, 520	2, 530	RHSLSHFVNL	0. 3
B*38:01	5, 617	5, 627	KSHFAIGLAL	0. 3
B*44:02	790	800	LLEIKDTEKY	0. 3
B*44:02	4, 120	4, 130	SMDNSPNLAW	0. 3
B*44:03	862	872	VELGTEVNEF	0. 3
B*44:03	2, 330	2, 340	AEWFLAYILF	0. 3
B*44:03	6, 213	6, 223	HECFVKRVDW	0. 3
B*45:01	5, 775	5, 785	AEIVDTVSAL	0. 3
B*46:01	2, 629	2, 639	VSLDNVLSTF	0. 3
B*46:01	3, 076	3, 086	FGEYSHVVAF	0. 3
B*49:01	1, 398	1, 408	VETKAIVSTI	0. 3
B*53:01	5, 566	5, 576	APTLVPQEHY	0. 3
B*57:01	6, 918	6, 928	CATVHTANKW	0. 3
B*58:01	3, 262	3, 272	ITSAVLQSGF	0. 3
B*58:01	3, 348	3, 358	HSMQNCVLKF	0. 3
C*03:04	1, 342	1, 352	YTVEEAKTVL	0. 3
C*07:01	4, 909	4, 919	ARLYYDSMSY	0. 3
A*01:01	1, 179	1, 189	YLAVFDKNLY	0. 31
A*01:01	2, 930	2, 940	ASGKPVPYCY	0. 31
A*01:01	5, 135	5, 145	DTDFVNEFYA	0. 31
A*02:01	967	977	AALQPENPHL	0. 31
A*02:02	843	853	FELDERIDKV	0. 31
A*02:02	967	977	AALQPENPHL	0. 31
A*02:02	1, 266	1, 276	GNLHPDSATL	0. 31
A*02:02	3, 866	3, 876	KMSDVKCTSV	0. 31
A*02:02	4, 044	4, 054	KLDNDALNNI	0. 31
A*02:05	2, 222	2, 232	YLKSPNFSKL	0. 31
A*11:01	1, 880	1, 890	TTIKPVTYKL	0. 31
A*11:01	2, 197	2, 207	ASMPTTIAKN	0. 31
A*23:01	4, 990	5, 000	KFYGGWHNML	0. 31
A*23:01	6, 427	6, 437	AYNMMISAGF	0. 31
A*23:01	6, 889	6, 899	RQWLPTGTLL	0. 31
A*24:02	1, 178	1, 188	VYLAVFDKNL	0. 31
A*24:02	4, 682	4, 692	RYFKYWDQTY	0. 31
A*25:01	246	256	EKSYELQTPF	0. 31
A*25:01	1, 186	1, 196	NLYDKLVSSF	0. 31
A*26:01	1, 186	1, 196	NLYDKLVSSF	0. 31

A*26:01	2, 277	2, 287	NSTNVTIATY	0. 31
A*26:01	2, 587	2, 597	DSAEVAVKMF	0. 31
A*26:01	4, 734	4, 744	GVPFVVSTGY	0. 31
A*29:02	905	915	YYLFDESGEF	0. 31
A*29:02	3, 105	3, 115	FLPGVYSVIY	0. 31
A*29:02	3, 200	3, 210	SDVLLPLTQY	0. 31
A*29:02	3, 740	3, 750	ALIISVTSNY	0. 31
A*29:02	5, 469	5, 479	ATEETFKLSY	0. 31
A*29:02	5, 675	5, 685	FKVNSTLEQY	0. 31
A*29:02	5, 985	5, 995	ISMMGFKMNY	0. 31
A*29:02	6, 289	6, 299	KAYKIEELFY	0. 31
A*31:01	6, 321	6, 331	RYPANSIVCR	0. 31
A*32:01	4, 189	4, 199	LLSDLQDLKW	0. 31
A*33:01	68	78	YVFIKRSDAR	0. 31
A*33:01	634	644	KFKEGVEFLR	0. 31
A*33:01	4, 504	4, 514	DGDMVPHISR	0. 31
A*33:01	4, 737	4, 747	FVVSTGYHFR	0. 31
A*68:01	273	283	FVFPLNSIIK	0. 31
A*68:01	278	288	NSIIKTIQPR	0. 31
A*68:02	328	338	ETSWQTGDFV	0. 31
A*68:02	4, 621	4, 631	QTTPGSGVPV	0. 31
A*68:02	5, 758	5, 768	MKTIGPDMFL	0. 31
B*07:02	884	894	QPVSELLTPL	0. 31
B*07:02	3, 362	3, 372	ANPKTPKYKF	0. 31
B*15:01	500	510	KQIVESCGNF	0. 31
B*15:01	1, 402	1, 412	AIVSTIQRKY	0. 31
B*15:01	2, 376	2, 386	LVQMAPISAM	0. 31
B*15:01	3, 267	3, 277	LQSGFRKMAF	0. 31
B*15:01	3, 645	3, 655	LLPSLATVAY	0. 31
B*15:03	95	105	IQYGRSGETL	0. 31
B*27:05	3, 197	3, 207	KLRSDVLLPL	0. 31
B*27:05	4, 760	4, 770	SSRLSFKELL	0. 31
B*35:01	5, 226	5, 236	LPYPDPSRIL	0. 31
B*35:03	3, 646	3, 656	LPSLATVAYF	0. 31
B*35:03	6, 505	6, 515	TLPVNVAFEL	0. 31
B*39:01	164	174	KHSSGVTREL	0. 31
B*39:01	4, 776	4, 786	AMHAASGNLL	0. 31
B*40:01	1, 010	1, 020	IVEVQPQLEM	0. 31
B*40:01	5, 496	5, 506	WEVGKPRPPL	0. 31
B*44:02	940	950	EFEPSTQYEY	0. 31
B*45:01	4, 262	4, 272	ATEVPANSTV	0. 31
B*46:01	3, 246	3, 256	FSNSGSDVLY	0. 31
B*46:01	3, 564	3, 574	VVRQCSGVTF	0. 31

B*46:01	5,619	5,629	HFAIGLALYY	0.31
B*58:01	1,497	1,507	SSSKTPEEHF	0.31
B*58:01	2,312	2,322	IQITISSFKW	0.31
C*02:02	134	144	HSYGADLKSF	0.31
C*03:02	6,298	6,308	YSYATHSDKF	0.31
C*05:01	6,906	6,916	FVSDADSTLI	0.31
C*07:02	3,816	3,826	YDYLVSTQEF	0.31
C*07:04	4,825	4,835	FFKEGSSVEL	0.31
C*08:02	1,342	1,352	YTVEEAKTVL	0.31
C*12:02	4,276	4,286	AFAVDAAKAY	0.31
C*12:03	404	414	IAFGGCVFSY	0.31
C*12:03	2,146	2,156	YAKPFLNKVV	0.31
C*15:02	3,103	3,113	YSFLPGVYSV	0.31
A*01:01	940	950	EFEPSTQYEY	0.32
A*01:01	6,291	6,301	YKIEELFYSY	0.32
A*02:01	4,519	4,529	YTMADLVYAL	0.32
A*02:05	586	596	FTSDLATNNL	0.32
A*02:05	3,334	3,344	FLVQAGNVQL	0.32
A*02:06	14	24	VQLSLPVLQV	0.32
A*02:06	5,725	5,735	YVYIGDPAQL	0.32
A*03:01	6,674	6,684	AMDEFIERYK	0.32
A*23:01	2,792	2,802	FYLIIPVHVM	0.32
A*23:01	6,306	6,316	KFTDGVCLFW	0.32
A*24:02	1,158	1,168	IFGADPIHSL	0.32
A*24:02	2,637	2,647	TFISAARQGF	0.32
A*24:02	6,427	6,437	AYNMMISAGF	0.32
A*25:01	2,440	2,450	YVYANGGKGF	0.32
A*25:01	3,673	3,683	DMVDTSLSGF	0.32
A*25:01	4,087	4,097	NTCDGTTFTY	0.32
A*26:01	896	906	DLDEWSMATY	0.32
A*26:01	1,060	1,070	TVVVNAANVY	0.32
A*29:02	3,496	3,506	NDFNLVAMKY	0.32
A*29:02	3,950	3,960	ASEFSSLPSY	0.32
A*29:02	5,861	5,871	TVDSSQGSEY	0.32
A*32:01	881	891	KTLQPVSELL	0.32
A*32:01	5,819	5,829	RPQIGVVREF	0.32
A*32:01	6,889	6,899	RQWLPTGTLL	0.32
A*32:01	6,970	6,980	VAIKITEHSW	0.32
A*33:01	5,138	5,148	FVNEFYAYLR	0.32
A*68:01	709	719	FVTHSKGLYR	0.32
A*68:01	6,060	6,070	NTDFSRVSAK	0.32
A*68:02	1,250	1,260	ETKFLTENLL	0.32
A*68:02	3,908	3,918	DTTEAFEKMV	0.32

A*68:02	5,560	5,570	TVMPLSAPTL	0.32
B*08:01	3,631	3,641	MMFVKHKHAF	0.32
B*08:01	4,341	4,351	HPNPKGFCDL	0.32
B*08:01	4,574	4,584	NLGERVRQAL	0.32
B*15:03	1,532	1,542	FLKRGDKSVY	0.32
B*18:01	7,005	7,015	SEAFLIGCNY	0.32
B*38:01	1,584	1,594	LHTQVVDMSM	0.32
B*38:01	5,287	5,297	KLHDELTGHM	0.32
B*39:01	4,593	4,603	MRNAGIVGVL	0.32
B*40:01	2,073	2,083	TTEVVGDIIL	0.32
B*40:01	4,262	4,272	ATEVPANSTV	0.32
B*40:02	112	122	GEIPVAYRKV	0.32
B*40:02	672	682	KESVQTFFKL	0.32
B*40:02	5,572	5,582	QEHYVRITGL	0.32
B*45:01	1,016	1,026	QLEMELTPVV	0.32
B*51:01	5,020	5,030	DRAMPNMLRI	0.32
B*53:01	2,137	2,147	SVPWDTIANY	0.32
B*53:01	3,124	3,134	DVSFLAHIQW	0.32
C*02:02	5,003	5,013	YSDVENPHLM	0.32
C*04:01	6,579	6,589	FFDGRVDGQV	0.32
C*04:43	6,579	6,589	FFDGRVDGQV	0.32
C*06:02	2,953	2,963	LRPDTRYVLM	0.32
C*14:02	1,541	1,551	YYTSNPTTFH	0.32
C*14:02	5,627	5,637	YYPSARIVYT	0.32
A*02:01	1,442	1,452	TLNDLNETLV	0.33
A*02:02	984	994	WLDDDSQQTV	0.33
A*02:02	4,915	4,925	SMSYEDQDAL	0.33
A*02:02	6,497	6,507	VELFENKTTL	0.33
A*02:02	6,861	6,871	TLAVPYNMRV	0.33
A*02:05	3,103	3,113	YSFLPGVYSV	0.33
A*02:05	5,225	5,235	YLPYPDPSRI	0.33
A*11:01	49	59	GTCGLVEVEK	0.33
A*11:01	813	823	FTLKGGAPTK	0.33
A*11:01	1,120	1,130	LHVVGPNVNK	0.33
A*11:01	1,641	1,651	HTTDPSFLGR	0.33
A*11:01	5,608	5,618	TLQGPPGTGK	0.33
A*23:01	6,422	6,432	RLYLDAYNMM	0.33
A*24:02	5,283	5,293	QYIRKLHDEL	0.33
A*24:02	6,889	6,899	RQWLPTGTLL	0.33
A*26:01	3,673	3,683	DMVDTSLSGF	0.33
A*29:02	4,867	4,877	LFVVEVVDKY	0.33
A*29:02	5,175	5,185	SIKNFKSVLY	0.33
A*29:02	6,367	6,377	VNLKQLPFFY	0.33

A*31:01	282	292	KTIQPRVEKK	0.33
A*32:01	1,420	1,430	VVDYGARFYF	0.33
A*33:01	278	288	NSIIKTIQPR	0.33
A*33:01	5,880	5,890	ETAHSCNVNR	0.33
A*68:01	5,138	5,148	FVNEFYAYLR	0.33
A*68:02	4,118	4,128	EISMDNSPNL	0.33
B*15:01	1,631	1,641	TLRVEAFEYY	0.33
B*15:01	3,631	3,641	MMFVKHKHAF	0.33
B*15:03	2,501	2,511	VKNGSIHLYF	0.33
B*15:03	6,455	6,465	RLQSLENVAF	0.33
B*27:05	3,809	3,819	FRLTLGVYDY	0.33
B*35:01	378	388	VGPEHSLAEY	0.33
B*38:01	3,371	3,381	FVRIQPGQTF	0.33
B*38:01	3,584	3,594	THHWLLLTIL	0.33
B*39:01	3,584	3,594	THHWLLLTIL	0.33
B*40:01	3,076	3,086	FGEYSHVVAF	0.33
B*45:01	656	666	ACEIVGGQIV	0.33
B*45:01	1,139	1,149	AYENFNQHEV	0.33
B*46:01	2,137	2,147	SVPWDTIANY	0.33
B*46:01	2,317	2,327	SSFKWDLTAF	0.33
B*46:01	5,675	5,685	FKVNSTLEQY	0.33
B*46:01	6,651	6,661	FTQSRNLQEF	0.33
B*46:01	6,777	6,787	VTIDYTEISF	0.33
B*49:01	2,053	2,063	SEEVVENPTI	0.33
B*49:01	2,518	2,528	YERHSLSHFV	0.33
B*49:01	2,908	2,918	IEYTDFATSA	0.33
B*51:01	6,087	6,097	LPWNVVRIKI	0.33
B*53:01	1,453	1,463	MPLGYVTHGL	0.33
B*53:01	5,819	5,829	RPQIGVVREF	0.33
B*57:01	4,030	4,040	VTSAMQIMLF	0.33
B*58:01	4,678	4,688	KLFDRYFKYW	0.33
B*58:01	4,830	4,840	SSVELKHFFF	0.33
C*07:02	4,465	4,475	SYFVVKRHTF	0.33
C*08:02	1,549	1,559	FHLDGEVITF	0.33
C*12:02	3,960	3,970	AAFATAQEAY	0.33
C*14:02	2,792	2,802	FYLIIPVHVM	0.33
C*14:02	3,748	3,758	NYSGVVTTVM	0.33
C*14:02	4,176	4,186	YYNTTKGGRF	0.33
C*14:02	5,539	5,549	VYRGTTTYKL	0.33
C*17:01	874	884	VVADAVIKTL	0.33
C*17:03	874	884	VVADAVIKTL	0.33
A*01:01	1,615	1,625	HNSHEGKTFY	0.34
A*02:01	4,044	4,054	KLDNDALNNI	0.34

A*02:06	4, 519	4, 529	YTMADLVYAL	0. 34
A*03:01	5, 982	5, 992	RRLISMMGFK	0. 34
A*11:01	5, 953	5, 963	APTHLSVDTK	0. 34
A*11:01	6, 390	6, 400	VSDIDYVPLK	0. 34
A*23:01	3, 135	3, 145	VMFTPLVPFW	0. 34
A*23:01	6, 983	6, 993	LYKLMGHFAW	0. 34
A*24:02	4, 916	4, 926	MSYEDQDALF	0. 34
A*29:02	3, 650	3, 660	ATVAYFNMVY	0. 34
A*29:02	4, 983	4, 993	TVVIGTSKFY	0. 34
A*32:01	3, 253	3, 263	VLYQPPQTSI	0. 34
A*32:01	6, 032	6, 042	GTNLPLQLGF	0. 34
A*33:01	1, 159	1, 169	FGADPIHSLR	0. 34
A*68:01	1, 221	1, 231	TESKPSVEQR	0. 34
A*68:01	6, 199	6, 209	VASCDAIMTR	0. 34
A*68:02	5, 815	5, 825	SAINRPQIGV	0. 34
B*07:02	18	28	LPVLQVRDVL	0. 34
B*07:02	1, 453	1, 463	MPLGYVTHGL	0. 34
B*08:01	2, 109	2, 119	SLTIKKPNEL	0. 34
B*08:01	3, 194	3, 204	MYLKLRSDVL	0. 34
B*15:01	6, 889	6, 899	RQWLPTGTLL	0. 34
B*15:03	5, 732	5, 742	AQLPAPRTLL	0. 34
B*27:05	3, 578	3, 588	KRTIKGTHHW	0. 34
B*35:03	275	285	FPLNSIIKTI	0. 34
B*39:01	5, 948	5, 958	LHPTQAPTHL	0. 34
B*40:01	7, 066	7, 076	KEGQINDMIL	0. 34
B*44:03	4, 457	4, 467	DEDDNLIDSY	0. 34
B*46:01	6, 805	6, 815	SQAWQPGVAM	0. 34
B*51:01	5, 322	5, 332	YTPHTVLQAV	0. 34
B*51:01	5, 561	5, 571	VMPLSAPTLV	0. 34
B*53:01	3, 645	3, 655	LLPSLATVAY	0. 34
B*57:01	4, 423	4, 433	TSTDVVYRAF	0. 34
B*57:01	4, 829	4, 839	GSSVELKHFF	0. 34
B*57:01	5, 796	5, 806	KSAQCFKMFY	0. 34
B*57:01	6, 032	6, 042	GTNLPLQLGF	0. 34
B*58:01	1, 369	1, 379	KQEILGTVSW	0. 34
B*58:01	4, 120	4, 130	SMDNSPNLAW	0. 34
C*02:02	7, 036	7, 046	NTNPIQLSSY	0. 34
C*04:01	5, 288	5, 298	LHDELTGHML	0. 34
C*04:43	5, 288	5, 298	LHDELTGHML	0. 34
C*05:01	1, 556	1, 566	ITFDNLKTLL	0. 34
C*08:02	2, 469	2, 479	FISDEVARDL	0. 34
C*08:02	6, 637	6, 647	KKVDGVVQQL	0. 34
C*16:01	1, 482	1, 492	VSSPDAVTAY	0. 34

A*02:01	5, 250	5, 260	TLMIERFVSL	0. 35
A*03:01	1, 777	1, 787	GTLSYEQFKK	0. 35
A*11:01	2, 104	2, 114	YVDNSSLTIK	0. 35
A*11:01	2, 516	2, 526	KTYERHSLSH	0. 35
A*11:01	2, 794	2, 804	LIIPVHVMSK	0. 35
A*11:01	3, 703	3, 713	RTVYDDGARR	0. 35
A*23:01	1, 910	1, 920	SYFTEQPIDL	0. 35
A*24:02	4, 690	4, 700	TYHPNCVNCL	0. 35
A*24:02	4, 990	5, 000	KFYGGWHNML	0. 35
A*24:02	6, 365	6, 375	AFVNLKQLPF	0. 35
A*25:01	2, 323	2, 333	LTAFGLVAEW	0. 35
A*26:01	2, 440	2, 450	YVYANGGKGF	0. 35
A*31:01	175	185	RELNGGAYTR	0. 35
A*68:01	3, 335	3, 345	LVQAGNVQLR	0. 35
A*68:02	4, 306	4, 316	HTGTGQAITV	0. 35
B*07:02	308	318	SPNECNQMCL	0. 35
B*44:02	862	872	VELGTEVNEF	0. 35
B*46:01	2, 324	2, 334	TAFGLVAEWF	0. 35
B*46:01	3, 645	3, 655	LLPSLATVAY	0. 35
B*49:01	248	258	SYELQTPFEI	0. 35
B*49:01	376	386	SEVGPEHSLA	0. 35
B*53:01	2, 323	2, 333	LTAFGLVAEW	0. 35
B*57:01	6, 777	6, 787	VTIDYTEISF	0. 35
B*58:01	6, 777	6, 787	VTIDYTEISF	0. 35
C*03:04	3, 910	3, 920	TEAFEKMVSL	0. 35
C*07:02	4, 909	4, 919	ARLYYDSMSY	0. 35
C*07:04	1, 158	1, 168	IFGADPIHSL	0. 35
C*08:02	874	884	VVADAVIKTL	0. 35
A*01:01	4, 420	4, 430	GTGTSTDVVY	0. 36
A*01:01	6, 678	6, 688	FIERYKLEGY	0. 36
A*02:01	1, 684	1, 694	ALLTLQQIEL	0. 36
A*02:02	1, 283	1, 293	FLKKDAPYIV	0. 36
A*03:01	692	702	SIIIGGAKLK	0. 36
A*23:01	2, 219	2, 229	SFNYLKSPNF	0. 36
A*23:01	2, 440	2, 450	YVYANGGKGF	0. 36
A*23:01	4, 176	4, 186	YYNTTKGGRF	0. 36
A*23:01	5, 283	5, 293	QYIRKLHDEL	0. 36
A*23:01	6, 423	6, 433	LYLDAYNMMI	0. 36
A*24:02	1, 910	1, 920	SYFTEQPIDL	0. 36
A*24:02	3, 163	3, 173	SNYLKRRVVF	0. 36
A*24:02	6, 306	6, 316	KFTDGVCLFW	0. 36
A*24:02	6, 423	6, 433	LYLDAYNMMI	0. 36
A*25:01	1, 274	1, 284	TLVSDIDITF	0. 36

A*29:02	1,713	1,723	ANFCALILAY	0.36
A*29:02	6,368	6,378	NLKQLPFFYY	0.36
A*31:01	3,202	3,212	VLLPLTQYNR	0.36
A*32:01	676	686	QTFFKLVNKF	0.36
A*68:01	2,945	2,955	EGSVAYESLR	0.36
A*68:02	5,322	5,332	YTPHTVLQAV	0.36
B*07:02	5,948	5,958	LHPTQAPTHL	0.36
B*08:01	2,222	2,232	YLKSPNFSKL	0.36
B*15:01	3,697	3,707	LILMTARTVY	0.36
B*27:05	1,424	1,434	GARFYFYTSK	0.36
B*27:05	3,546	3,556	GRTILGSALL	0.36
B*35:01	1,586	1,596	TQVVDMSMTY	0.36
B*35:01	2,379	2,389	MAPISAMVRM	0.36
B*38:01	1,111	1,121	SGHNLAKHCL	0.36
B*38:01	6,243	6,253	QHMVVKAALL	0.36
B*38:01	6,713	6,723	KRFKESPFEL	0.36
B*40:02	9	19	NEKTHVQLSL	0.36
B*40:02	375	385	NSEVGPEHSL	0.36
B*40:02	724	734	REETGLLMPL	0.36
B*40:02	960	970	LEFGATSAAL	0.36
B*44:02	1,027	1,037	TIEVNSFSGY	0.36
B*44:02	1,728	1,738	GELGDVRETM	0.36
B*44:03	3,951	3,961	SEFSSLPSYA	0.36
B*44:03	6,419	6,429	NEYRLYLDAY	0.36
B*46:01	2,941	2,951	TNVLEGSVAY	0.36
B*46:01	3,058	3,068	VAIVVTCLAY	0.36
B*46:01	7,036	7,046	NTNPIQLSSY	0.36
B*49:01	1,367	1,377	NEKQEILGTV	0.36
B*49:01	3,910	3,920	TEAFEKMVSL	0.36
B*49:01	6,569	6,579	TETICAPLTV	0.36
B*53:01	3,834	3,844	LPPKNSIDAF	0.36
B*53:01	4,201	4,211	FPKSDGTGTI	0.36
B*53:01	6,809	6,819	QPGVAMPNLY	0.36
B*57:01	2,205	2,215	KNTVKSVGKF	0.36
B*57:01	6,506	6,516	LPVNVAFELW	0.36
C*03:02	134	144	HSYGADLKSF	0.36
C*07:01	5,116	5,126	YVRNLQHRLY	0.36
C*08:02	984	994	WLDDDSQQTV	0.36
C*08:02	1,160	1,170	GADPIHSLRV	0.36
C*08:02	1,603	1,613	YLDGADVTKI	0.36
C*14:02	3,118	3,128	TFYLTNDVSF	0.36
C*14:02	5,597	5,607	NYQKVGMQKY	0.36
C*14:02	6,298	6,308	YSYATHSDKF	0.36

C*16:01	1,878	1,888	YTTTIKPVTY	0.36
A*01:01	3,650	3,660	ATVAYFNMVY	0.37
A*01:01	5,566	5,576	APTLVPQEHY	0.37
A*02:01	1,036	1,046	YLKLTDNVYI	0.37
A*02:01	1,253	1,263	FLTENLLLYI	0.37
A*02:01	2,230	2,240	KLINIIIWFL	0.37
A*02:01	3,875	3,885	VVLLSVLQQL	0.37
A*02:02	1,603	1,613	YLDGADVTKI	0.37
A*02:02	4,098	4,108	SALWEIQQVV	0.37
A*02:02	5,250	5,260	TLMIERFVSL	0.37
A*02:06	6,889	6,899	RQWLPTGTLL	0.37
A*03:01	116	126	VAYRKVLLRK	0.37
A*03:01	273	283	FVFPLNSIIK	0.37
A*11:01	6,583	6,593	RVDGQVDLFR	0.37
A*11:01	6,737	6,747	ITDAQTGSSK	0.37
A*23:01	1,541	1,551	YYTSNPTTFH	0.37
A*23:01	3,163	3,173	SNYLKRRVVF	0.37
A*24:02	4,736	4,746	PFVVSTGYHF	0.37
A*25:01	471	481	EEIAIILASF	0.37
A*25:01	553	563	ETAQNSVRVL	0.37
A*26:01	1,630	1,640	DTLRVEAFEY	0.37
A*26:01	3,081	3,091	HVVAFNTLLF	0.37
A*26:01	6,777	6,787	VTIDYTEISF	0.37
A*29:02	4,167	4,177	ACTDDNALAY	0.37
A*31:01	1,650	1,660	RYMSALNHTK	0.37
A*33:01	2,111	2,121	TIKKPNELSR	0.37
A*33:01	5,224	5,234	VYLPYPDPSR	0.37
A*68:02	6,761	6,771	EIIKSQDLSV	0.37
B*08:01	1,186	1,196	NLYDKLVSSF	0.37
B*13:02	3,866	3,876	KMSDVKCTSV	0.37
B*15:01	95	105	IQYGRSGETL	0.37
B*18:01	3,951	3,961	SEFSSLPSYA	0.37
B*18:01	4,211	4,221	YTELEPPCRF	0.37
B*18:01	5,313	5,323	WEPEFYEAMY	0.37
B*27:05	3,070	3,080	MRFRRAFGEY	0.37
B*35:01	4,639	4,649	MPILTLTRAL	0.37
B*35:03	3,834	3,844	LPPKNSIDAF	0.37
B*39:01	1,639	1,649	YYHTTDPSFL	0.37
B*45:01	5,272	5,282	QEYADVFHLY	0.37
B*46:01	3,740	3,750	ALIISVTSNY	0.37
B*46:01	6,847	6,857	VAKYTQLCQY	0.37
B*53:01	6,357	6,367	HTPAFDKSAF	0.37
B*57:01	603	613	GGVVQLTSQW	0.37

C*04:01	3, 705	3, 715	VYDDGARRVW	0. 37
C*04:43	3, 705	3, 715	VYDDGARRVW	0. 37
C*05:01	2, 469	2, 479	FISDEVARDL	0. 37
C*07:01	2, 952	2, 962	SLRPDTRYVL	0. 37
C*07:02	4, 682	4, 692	RYFKYWDQTY	0. 37
C*08:01	586	596	FTSDLATNNL	0. 37
C*08:02	4, 361	4, 371	CANDPVGFTL	0. 37
C*14:02	2, 637	2, 647	TFISAARQGF	0. 37
C*15:02	5, 003	5, 013	YSDVENPHLM	0. 37
A*01:01	1, 448	1, 458	ETLVTMPLGY	0. 38
A*01:01	5, 675	5, 685	FKVNSTLEQY	0. 38
A*02:01	3, 197	3, 207	KLRSDVLLPL	0. 38
A*02:01	6, 497	6, 507	VELFENKTTL	0. 38
A*02:02	5, 450	5, 460	ILANTCTERL	0. 38
A*02:02	5, 474	5, 484	FKLSYGIATV	0. 38
A*02:02	6, 106	6, 116	NLSDRVVFVL	0. 38
A*02:06	3, 644	3, 654	FLLPSLATVA	0. 38
A*03:01	5, 064	5, 074	VMCGGSLYVK	0. 38
A*03:01	5, 391	5, 401	QLYLGGMSYY	0. 38
A*23:01	4, 916	4, 926	MSYEDQDALF	0. 38
A*24:02	5, 226	5, 236	LPYPDPSRIL	0. 38
A*25:01	676	686	QTFFKLVNKF	0. 38
A*25:01	5, 158	5, 168	DAVVCFNSTY	0. 38
A*26:01	5, 005	5, 015	DVENPHLMGW	0. 38
A*26:01	5, 077	5, 087	TSSGDATTAY	0. 38
A*31:01	3, 008	3, 018	RWVLNNDYYR	0. 38
A*31:01	4, 191	4, 201	SDLQDLKWAR	0. 38
A*32:01	4, 930	4, 940	RNVIPTITQM	0. 38
A*68:01	1, 181	1, 191	AVFDKNLYDK	0. 38
A*68:01	2, 111	2, 121	TIKKPNELSR	0. 38
A*68:02	874	884	VVADAVIKTL	0. 38
B*15:03	6, 657	6, 667	LQEFKPRSQM	0. 38
B*27:05	6, 218	6, 228	KRVDWTIEYP	0. 38
B*27:05	6, 680	6, 690	ERYKLEGYAF	0. 38
B*38:01	6, 664	6, 674	SQMEIDFLEL	0. 38
B*39:01	2, 802	2, 812	SKHTDFSSEI	0. 38
B*45:01	668	678	AKEIKESVQT	0. 38
B*46:01	3, 950	3, 960	ASEFSSLPSY	0. 38
B*46:01	5, 391	5, 401	QLYLGGMSYY	0. 38
B*49:01	1, 140	1, 150	YENFNQHEVL	0. 38
B*51:01	1, 501	1, 511	TPEEHFIETI	0. 38
B*51:01	2, 486	2, 496	INPTDQSSYI	0. 38
B*58:01	1, 878	1, 888	YTTTIKPVTY	0. 38

B*58:01	4,423	4,433	TSTDVVYRAF	0.38
C*02:02	1,482	1,492	VSSPDAVTAY	0.38
C*03:02	4,714	4,724	FSTVFPLTSF	0.38
C*03:04	3,973	3,983	VANGDSEVVL	0.38
C*08:02	4,338	4,348	HIDHPNPKGF	0.38
C*12:02	2,557	2,567	SSAKSASVYY	0.38
C*14:02	240	250	WYTERSEKSY	0.38
A*01:01	1,943	1,953	ADDLNQLTGY	0.39
A*01:01	4,625	4,635	GSGVPVVDSY	0.39
A*02:01	4,126	4,136	NLAWPLIVTA	0.39
A*02:01	4,477	4,487	YQHEETIYNL	0.39
A*02:05	3,051	3,061	SIVAGGIVAI	0.39
A*02:05	6,699	6,709	HSQLGGLHLL	0.39
A*02:06	5,732	5,742	AQLPAPRTLL	0.39
A*03:01	675	685	VQTFFKLVNK	0.39
A*03:01	5,028	5,038	RIMASLVLAR	0.39
A*23:01	1,549	1,559	FHLDGEVITF	0.39
A*23:01	2,103	2,113	AYVDNSSLTI	0.39
A*24:02	3,689	3,699	MYASAVVLLI	0.39
A*25:01	1,880	1,890	TTIKPVTYKL	0.39
A*25:01	5,188	5,198	NVFMSEAKCW	0.39
A*26:01	591	601	ATNNLVVMAY	0.39
A*26:01	3,105	3,115	FLPGVYSVIY	0.39
A*26:01	3,200	3,210	SDVLLPLTQY	0.39
A*26:01	5,361	5,371	DHVISTSHKL	0.39
A*29:02	912	922	GEFKLASHMY	0.39
A*29:02	6,291	6,301	YKIEELFYSY	0.39
A*31:01	1,221	1,231	TESKPSVEQR	0.39
A*32:01	4,492	4,502	AVAKHDFFKF	0.39
A*33:01	3,223	3,233	SGAMDTTSYR	0.39
A*33:01	5,712	5,722	DLSVVNARLR	0.39
A*68:02	1,616	1,626	NSHEGKTFYV	0.39
A*68:02	4,771	4,781	YAADPAMHAA	0.39
A*68:02	5,319	5,329	EAMYTPHTVL	0.39
B*14:02	1,503	1,513	EEHFIETISL	0.39
B*15:01	6,615	6,625	KQASLNGVTL	0.39
B*15:03	4,563	4,573	VENPDILRVY	0.39
B*27:05	98	108	GRSGETLGVL	0.39
B*27:05	6,091	6,101	VVRIKIVQML	0.39
B*35:03	1,965	1,975	FPDLNGDVVA	0.39
B*35:03	5,319	5,329	EAMYTPHTVL	0.39
B*39:01	6,793	6,803	GHVETFYPKL	0.39
B*40:01	9	19	NEKTHVQLSL	0.39

B*40:01	6,716	6,726	KESPFELEDF	0.39
B*40:02	1,503	1,513	EEHFIETISL	0.39
B*46:01	6,432	6,442	ISAGFSLWVY	0.39
B*51:01	3,659	3,669	YMPASWVMRI	0.39
B*57:01	1,482	1,492	VSSPDAVTAY	0.39
B*58:01	1,812	1,822	MSAPPAQYEL	0.39
C*01:02	5,841	5,851	ISPYNSQNAV	0.39
C*02:02	2,440	2,450	YVYANGGKGF	0.39
C*02:02	4,265	4,275	VPANSTVLSF	0.39
C*03:02	3,222	3,232	FSGAMDTTSY	0.39
C*08:01	1,812	1,822	MSAPPAQYEL	0.39
C*14:02	5,002	5,012	VYSDVENPHL	0.39
C*14:02	5,272	5,282	QEYADVFHLY	0.39
A*01:01	145	155	LGDELGTDPY	0.4
A*01:01	1,630	1,640	DTLRVEAFEY	0.4
A*02:01	3,253	3,263	VLYQPPQTSI	0.4
A*02:02	3,253	3,263	VLYQPPQTSI	0.4
A*02:05	1,295	1,305	VVQEGVLTAV	0.4
A*03:01	728	738	GLLMPLKAPK	0.4
A*03:01	1,315	1,325	TEMLAKALRK	0.4
A*03:01	6,246	6,256	VVKAALLADK	0.4
A*11:01	692	702	SIIIGGAKLK	0.4
A*11:01	2,618	2,628	VATAEAELAK	0.4
A*11:01	4,981	4,991	GATVVIGTSK	0.4
A*11:01	6,354	6,364	HAFHTPAFDK	0.4
A*24:02	134	144	HSYGADLKSF	0.4
A*24:02	1,623	1,633	FYVLPNDDTL	0.4
A*24:02	5,910	5,920	LYDKLQFTSL	0.4
A*25:01	1,869	1,879	TDVFYKENSY	0.4
A*25:01	6,357	6,367	HTPAFDKSAF	0.4
A*25:01	6,777	6,787	VTIDYTEISF	0.4
A*25:01	6,970	6,980	VAIKITEHSW	0.4
A*26:01	938	948	EEEFEPSTQY	0.4
A*26:01	4,626	4,636	SGVPVVDSYY	0.4
A*26:01	4,714	4,724	FSTVFPLTSF	0.4
A*26:01	5,175	5,185	SIKNFKSVLY	0.4
A*26:01	5,465	5,475	ETLKATEETF	0.4
A*29:02	1,402	1,412	AIVSTIQRKY	0.4
A*29:02	1,482	1,492	VSSPDAVTAY	0.4
A*29:02	3,645	3,655	LLPSLATVAY	0.4
A*29:02	6,673	6,683	LAMDEFIERY	0.4
A*31:01	6,446	6,456	TYNLWNTFTR	0.4
A*68:01	2,185	2,195	CTFTRSTNSR	0.4

A*68:01	2,757	2,767	NVVTTKIALK	0.4
A*68:01	2,981	2,991	VTTFDSEYCR	0.4
A*68:02	4,793	4,803	FSVAALTNNV	0.4
A*68:02	5,365	5,375	STSHKLVLSV	0.4
B*08:01	5,978	5,988	DMTYRRLISM	0.4
B*15:01	404	414	IAFGGCVFSY	0.4
B*35:01	5,158	5,168	DAVVCFNSTY	0.4
B*35:01	7,038	7,048	NPIQLSSYSL	0.4
B*37:01	4,670	4,680	YDFTEERLKL	0.4
B*38:01	5,907	5,917	DRDLYDKLQF	0.4
B*39:01	242	252	TERSEKSYEL	0.4
B*40:01	268	278	GECPNFVFPL	0.4
B*40:01	340	350	TCEFCGTENL	0.4
B*40:01	743	753	EGETLPTEVL	0.4
B*40:02	6,666	6,676	MEIDFLELAM	0.4
B*44:02	538	548	SEAARVVRSI	0.4
B*51:01	6,358	6,368	TPAFDKSAFV	0.4
B*58:01	4,829	4,839	GSSVELKHFF	0.4
B*58:01	6,918	6,928	CATVHTANKW	0.4
C*01:02	5,561	5,571	VMPLSAPTLV	0.4
C*02:02	6,651	6,661	FTQSRNLQEF	0.4
C*02:02	6,673	6,683	LAMDEFIERY	0.4
C*03:04	1,542	1,552	YTSNPTTFHL	0.4
C*03:04	2,101	2,111	MAAYVDNSSL	0.4
C*04:01	4,668	4,678	LKYDFTEERL	0.4
C*04:43	4,668	4,678	LKYDFTEERL	0.4
C*07:01	3,825	3,835	FRYMNSQGLL	0.4
C*07:04	6,713	6,723	KRFKESPFEL	0.4
C*08:01	1,342	1,352	YTVEEAKTVL	0.4
C*08:01	4,361	4,371	CANDPVGFTL	0.4
C*12:02	1,878	1,888	YTTTIKPVTY	0.4
C*12:02	3,246	3,256	FSNSGSDVLY	0.4
C*12:03	3,103	3,113	YSFLPGVYSV	0.4
C*14:02	1,638	1,648	EYYHTTDPSF	0.4
C*14:02	1,910	1,920	SYFTEQPIDL	0.4
C*14:02	3,119	3,129	FYLTNDVSFL	0.4
C*14:02	4,816	4,826	FYDFAVSKGF	0.4
C*17:01	569	579	ILDGISQYSL	0.4
C*17:03	569	579	ILDGISQYSL	0.4
A*01:01	4,844	4,854	NAAISDYDYY	0.41
A*02:01	2,126	2,136	TLATHGLAAV	0.41
A*02:01	4,562	4,572	FVENPDILRV	0.41
A*02:01	6,082	6,092	LMYKGLPWNV	0.41

A*02:02	4, 008	4, 018	KMADQAMTQM	0. 41
A*03:01	3, 211	3, 221	RYLALYNKYK	0. 41
A*03:01	4, 676	4, 686	RLKLFDRYFK	0. 41
A*03:01	4, 762	4, 772	RLSFKELLVY	0. 41
A*11:01	5, 246	5, 256	KTDGTLMIER	0. 41
A*25:01	669	679	KEIKESVQTF	0. 41
A*26:01	2, 673	2, 683	DSCNNYMLTY	0. 41
A*29:02	2, 901	2, 911	CYTPSKLIEY	0. 41
A*29:02	6, 145	6, 155	TCFSTASDTY	0. 41
A*31:01	2, 007	2, 017	TYKPNTWCIR	0. 41
A*33:01	1, 976	1, 986	DYKHYTPSFK	0. 41
A*68:01	4, 520	4, 530	TMADLVYALR	0. 41
A*68:02	83	93	HVMVELVAEL	0. 41
A*68:02	5, 472	5, 482	ETFKLSYGIA	0. 41
B*07:02	2, 609	2, 619	VPMEKLKTLV	0. 41
B*07:02	3, 617	3, 627	LPFAMGIIAM	0. 41
B*15:01	790	800	LLEIKDTEKY	0. 41
B*15:01	3, 756	3, 766	VMFLARGIVF	0. 41
B*15:01	6, 067	6, 077	SAKPPPGDQF	0. 41
B*15:01	6, 657	6, 667	LQEFKPRSQM	0. 41
B*15:03	4, 395	4, 405	MLQSADAQSF	0. 41
B*15:03	5, 929	5, 939	LQAENVTGLF	0. 41
B*27:05	1, 706	1, 716	RARAGEAANF	0. 41
B*35:01	3, 660	3, 670	MPASWVMRIM	0. 41
B*35:03	3, 660	3, 670	MPASWVMRIM	0. 41
B*39:01	6, 243	6, 253	QHMVVKAALL	0. 41
B*40:01	5, 483	5, 493	VREVLSDREL	0. 41
B*40:02	2, 471	2, 481	SDEVARDLSL	0. 41
B*44:03	112	122	GEIPVAYRKV	0. 41
B*49:01	998	1, 008	GSEDNQTTTI	0. 41
B*49:01	5, 272	5, 282	QEYADVFHLY	0. 41
B*53:01	1, 663	1, 673	YPQVNGLTSI	0. 41
B*53:01	1, 665	1, 675	QVNGLTSIKW	0. 41
B*53:01	5, 663	5, 673	IPARARVECF	0. 41
B*53:01	5, 948	5, 958	LHPTQAPTHL	0. 41
B*57:01	3, 944	3, 954	ATLQAIASEF	0. 41
B*58:01	603	613	GGVVQLTSQW	0. 41
B*58:01	2, 228	2, 238	FSKLINIIIW	0. 41
B*58:01	4, 030	4, 040	VTSAMQIMLF	0. 41
B*58:01	6, 032	6, 042	GTNLPLQLGF	0. 41
B*58:01	6, 441	6, 451	YKQFDTYNLW	0. 41
C*01:02	1, 158	1, 168	IFGADPIHSL	0. 41
C*02:02	3, 904	3, 914	LLAKDTTEAF	0. 41

C*05:01	5, 002	5, 012	VYSDVENPHL	0. 41
C*06:02	2, 952	2, 962	SLRPDTRYVL	0. 41
C*08:02	5, 154	5, 164	ILSDDAVVCF	0. 41
C*12:02	1, 482	1, 492	VSSPDAVTAY	0. 41
C*12:02	6, 673	6, 683	LAMDEFIERY	0. 41
C*14:02	3, 426	3, 436	SFCYMHHMEL	0. 41
C*14:02	4, 276	4, 286	AFAVDAAKAY	0. 41
A*01:01	4, 934	4, 944	PTITQMNLKY	0. 42
A*02:01	3, 090	3, 100	FLMSFTVLCL	0. 42
A*02:02	4, 562	4, 572	FVENPDILRV	0. 42
A*03:01	614	624	TNIFGTVYEK	0. 42
A*11:01	3, 839	3, 849	SIDAFKLNIK	0. 42
A*23:01	1, 639	1, 649	YYHTTDPSFL	0. 42
A*23:01	3, 689	3, 699	MYASAVVLLI	0. 42
A*24:02	3, 179	3, 189	TFEEAALCTF	0. 42
A*24:02	7, 013	7, 023	NYLGKPCEQI	0. 42
A*25:01	134	144	HSYGADLKSF	0. 42
A*26:01	3, 911	3, 921	EAFEKMVSLL	0. 42
A*26:01	5, 272	5, 282	QEYADVFHLY	0. 42
A*26:01	6, 641	6, 651	GVVQQLPETY	0. 42
A*29:02	1, 631	1, 641	TLRVEAFEYY	0. 42
A*31:01	709	719	FVTHSKGLYR	0. 42
A*32:01	1, 772	1, 782	AVMYMGTLSY	0. 42
A*33:01	1, 215	1, 225	EVKPFITESK	0. 42
A*33:01	4, 235	4, 245	FIKGLNNLNR	0. 42
A*33:01	4, 818	4, 828	DFAVSKGFFK	0. 42
A*68:01	1, 314	1, 324	TTEMLAKALR	0. 42
A*68:01	4, 065	4, 075	NIIPLTTAAK	0. 42
A*68:02	1, 441	1, 451	NTLNDLNETL	0. 42
A*68:02	1, 927	1, 937	NASFDNFKFV	0. 42
A*68:02	3, 052	3, 062	IVAGGIVAIV	0. 42
A*68:02	5, 063	5, 073	MVMCGGSLYV	0. 42
B*07:02	2, 021	2, 031	TKPVETSNSF	0. 42
B*14:02	4, 025	4, 035	DKRAKVTSAM	0. 42
B*15:01	5, 597	5, 607	NYQKVGMQKY	0. 42
B*15:03	127	137	GNKGAGGHSY	0. 42
B*15:03	5, 537	5, 547	AVVYRGTTTY	0. 42
B*18:01	3, 612	3, 622	YENAFLPFAM	0. 42
B*18:01	5, 054	5, 064	NECAQVLSEM	0. 42
B*27:05	4, 429	4, 439	YRAFDIYNDK	0. 42
B*27:05	4, 593	4, 603	MRNAGIVGVL	0. 42
B*35:03	6, 357	6, 367	HTPAFDKSAF	0. 42
B*37:01	1, 862	1, 872	SEYKGPITDV	0. 42

B*38:01	6, 158	6, 168	HHSIGFDYVY	0. 42
B*44:02	4, 656	4, 666	TDLTKPYIKW	0. 42
B*44:03	911	921	SGEFKLASHM	0. 42
B*44:03	1, 503	1, 513	EEHFIETISL	0. 42
B*57:01	1, 369	1, 379	KQEILGTVSW	0. 42
B*58:01	3, 124	3, 134	DVSFLAHIQW	0. 42
C*02:02	5, 619	5, 629	HFAIGLALYY	0. 42
C*03:04	1, 519	1, 529	WSYSGQSTQL	0. 42
C*03:04	5, 319	5, 329	EAMYTPHTVL	0. 42
C*06:02	5, 500	5, 510	KPRPPLNRNY	0. 42
C*07:02	1, 186	1, 196	NLYDKLVSSF	0. 42
C*07:04	1, 549	1, 559	FHLDGEVITF	0. 42
C*08:01	3, 973	3, 983	VANGDSEVVL	0. 42
C*08:02	2, 960	2, 970	VLMDGSIIQF	0. 42
C*08:02	6, 491	6, 501	KVDGVDVELF	0. 42
C*14:02	3, 253	3, 263	VLYQPPQTSI	0. 42
C*15:02	3, 074	3, 084	RAFGEYSHVV	0. 42
C*16:01	1, 812	1, 822	MSAPPAQYEL	0. 42
A*01:01	2, 669	2, 679	EVTGDSCNNY	0. 43
A*01:01	5, 291	5, 301	ELTGHMLDMY	0. 43
A*01:01	5, 384	5, 394	CDVTDVTQLY	0. 43
A*02:01	1, 016	1, 026	QLEMELTPVV	0. 43
A*02:01	5, 725	5, 735	YVYIGDPAQL	0. 43
A*02:01	5, 928	5, 938	TLQAENVTGL	0. 43
A*02:01	6, 861	6, 871	TLAVPYNMRV	0. 43
A*02:02	3, 119	3, 129	FYLTNDVSFL	0. 43
A*02:02	3, 197	3, 207	KLRSDVLLPL	0. 43
A*02:06	4, 098	4, 108	SALWEIQQVV	0. 43
A*03:01	1, 120	1, 130	LHVVGPNVNK	0. 43
A*03:01	2, 493	2, 503	SYIVDSVTVK	0. 43
A*03:01	2, 605	2, 615	STFNVPMEKL	0. 43
A*03:01	2, 919	2, 929	VLAAECTIFK	0. 43
A*03:01	3, 703	3, 713	RTVYDDGARR	0. 43
A*24:02	95	105	IQYGRSGETL	0. 43
A*24:02	2, 219	2, 229	SFNYLKSPNF	0. 43
A*24:02	6, 983	6, 993	LYKLMGHFAW	0. 43
A*25:01	88	98	LVAELEGIQY	0. 43
A*25:01	2, 277	2, 287	NSTNVTIATY	0. 43
A*25:01	3, 081	3, 091	HVVAFNTLLF	0. 43
A*25:01	4, 653	4, 663	HVDTDLTKPY	0. 43
A*26:01	55	65	EVEKGVLPQL	0. 43
A*26:01	246	256	EKSYELQTPF	0. 43
A*26:01	471	481	EEIAIILASF	0. 43

A*29:02	2,393	2,403	ASFYYVWKSY	0.43
A*29:02	4,918	4,928	YEDQDALFAY	0.43
A*29:02	5,273	5,283	EYADVFHLYL	0.43
A*29:02	5,384	5,394	CDVTDVTQLY	0.43
A*29:02	6,726	6,736	IPMDSTVKNY	0.43
A*32:01	4,682	4,692	RYFKYWDQTY	0.43
A*33:01	6,002	6,012	MFITREEAIR	0.43
A*68:01	483	493	STSAFVETVK	0.43
A*68:01	4,077	4,087	VVIPDYNTYK	0.43
B*07:02	1,663	1,673	YPQVNGLTSI	0.43
B*15:01	2,137	2,147	SVPWDTIANY	0.43
B*15:01	3,818	3,828	YLVSTQEFRY	0.43
B*15:01	5,675	5,685	FKVNSTLEQY	0.43
B*15:03	3,631	3,641	MMFVKHKHAF	0.43
B*15:03	5,176	5,186	IKNFKSVLYY	0.43
B*27:05	3,210	3,220	NRYLALYNKY	0.43
B*35:01	3,276	3,286	FPSGKVEGCM	0.43
B*35:03	1,501	1,511	TPEEHFIETI	0.43
B*35:03	1,964	1,974	FFPDLNGDVV	0.43
B*35:03	7,053	7,063	FPLKLRGTAV	0.43
B*37:01	7,048	7,058	FDMSKFPLKL	0.43
B*40:01	1,019	1,029	MELTPVVQTI	0.43
B*40:02	5,318	5,328	YEAMYTPHTV	0.43
B*40:02	6,716	6,726	KESPFELEDF	0.43
B*46:01	4,795	4,805	VAALTNNVAF	0.43
B*58:01	322	332	KCDHCGETSW	0.43
B*58:01	3,884	3,894	LRVESSSKLW	0.43
C*01:02	2,379	2,389	MAPISAMVRM	0.43
C*01:02	5,745	5,755	TLEPEYFNSV	0.43
C*02:02	3,953	3,963	FSSLPSYAAF	0.43
C*02:02	4,276	4,286	AFAVDAAKAY	0.43
C*03:02	4,265	4,275	VPANSTVLSF	0.43
C*03:04	4,107	4,117	VDADSKIVQL	0.43
C*04:01	2,960	2,970	VLMDGSIIQF	0.43
C*04:01	5,273	5,283	EYADVFHLYL	0.43
C*04:43	2,960	2,970	VLMDGSIIQF	0.43
C*04:43	5,273	5,283	EYADVFHLYL	0.43
C*05:01	2,582	2,592	VSDVGDSAEV	0.43
C*07:02	5,272	5,282	QEYADVFHLY	0.43
C*07:02	5,725	5,735	YVYIGDPAQL	0.43
C*08:01	874	884	VVADAVIKTL	0.43
C*08:02	4,530	4,540	HFDEGNCDTL	0.43
C*08:02	6,693	6,703	VYGDFSHSQL	0.43

C*12:02	3, 904	3, 914	LLAKDTTEAF	0. 43
C*14:02	4, 990	5, 000	KFYGGWHNML	0. 43
C*16:01	76	86	ARTAPHGHVM	0. 43
C*16:01	6, 836	6, 846	SATLPKGIMM	0. 43
C*17:01	6, 637	6, 647	KKVDGVVQQL	0. 43
C*17:03	6, 637	6, 647	KKVDGVVQQL	0. 43
A*02:01	881	891	KTLQPVSELL	0. 44
A*02:01	1, 888	1, 898	KLDGVVCTEI	0. 44
A*02:01	5, 295	5, 305	HMLDMYSVML	0. 44
A*02:06	5, 369	5, 379	KLVLSVNPYV	0. 44
A*03:01	483	493	STSAFVETVK	0. 44
A*03:01	1, 525	1, 535	STQLGIEFLK	0. 44
A*03:01	2, 311	2, 321	TIQITISSFK	0. 44
A*03:01	3, 349	3, 359	SMQNCVLKFK	0. 44
A*03:01	4, 188	4, 198	ALLSDLQDLK	0. 44
A*03:01	4, 757	4, 767	NLHSSRLSFK	0. 44
A*11:01	4, 962	4, 972	TMTNRQFHQK	0. 44
A*11:01	5, 537	5, 547	AVVYRGTTTY	0. 44
A*26:01	2, 327	2, 337	GLVAEWFLAY	0. 44
A*26:01	2, 975	2, 985	EGSVRVVTTF	0. 44
A*26:01	3, 140	3, 150	LVPFWITIAY	0. 44
A*29:02	1, 823	1, 833	HGTFTCASEY	0. 44
A*29:02	2, 964	2, 974	GSIIQFPNTY	0. 44
A*29:02	4, 475	4, 485	SNYQHEETIY	0. 44
A*29:02	5, 176	5, 186	IKNFKSVLYY	0. 44
A*29:02	6, 158	6, 168	HHSIGFDYVY	0. 44
A*32:01	134	144	HSYGADLKSF	0. 44
A*32:01	478	488	ASFSASTSAF	0. 44
A*32:01	3, 096	3, 106	VLCLTPVYSF	0. 44
A*32:01	3, 987	3, 997	KSLNVAKSEF	0. 44
A*32:01	6, 148	6, 158	STASDTYACW	0. 44
A*33:01	3, 160	3, 170	WFFSNYLKRR	0. 44
B*07:02	2, 935	2, 945	VPYCYDTNVL	0. 44
B*07:02	3, 374	3, 384	IQPGQTFSVL	0. 44
B*08:01	1, 981	1, 991	TPSFKKGAKL	0. 44
B*08:01	3, 911	3, 921	EAFEKMVSLL	0. 44
B*15:01	59	69	GVLPQLEQPY	0. 44
B*15:01	2, 440	2, 450	YVYANGGKGF	0. 44
B*15:01	3, 600	3, 610	VQSTQWSLFF	0. 44
B*15:01	5, 732	5, 742	AQLPAPRTLL	0. 44
B*15:03	2, 500	2, 510	TVKNGSIHLY	0. 44
B*18:01	1, 027	1, 037	TIEVNSFSGY	0. 44
B*35:01	6, 627	6, 637	EAVKTQFNYY	0. 44

B*37:01	5,775	5,785	AEIVDTVSAL	0.44
B*38:01	5,954	5,964	PTHLSVDTKF	0.44
B*39:01	1,384	1,394	LAHAEETRKL	0.44
B*39:01	7,033	7,043	FWRNTNPIQL	0.44
B*40:01	998	1,008	GSEDNQTTTI	0.44
B*40:02	1,019	1,029	MELTPVVQTI	0.44
B*40:02	4,262	4,272	ATEVPANSTV	0.44
B*44:02	4,457	4,467	DEDDNLIDSY	0.44
B*44:03	375	385	NSEVGPEHSL	0.44
B*44:03	1,862	1,872	SEYKGPITDV	0.44
B*45:01	345	355	GTENLTKEGA	0.44
B*46:01	88	98	LVAELEGIQY	0.44
B*51:01	5,653	5,663	YLPIDKCSRI	0.44
B*57:01	3,153	3,163	ISTKHFYWFF	0.44
B*58:01	6,432	6,442	ISAGFSLWVY	0.44
C*01:02	5,322	5,332	YTPHTVLQAV	0.44
C*03:02	4,009	4,019	MADQAMTQMY	0.44
C*05:01	2,103	2,113	AYVDNSSLTI	0.44
C*05:01	6,777	6,787	VTIDYTEISF	0.44
C*08:01	2,469	2,479	FISDEVARDL	0.44
C*08:01	4,771	4,781	YAADPAMHAA	0.44
C*08:02	136	146	YGADLKSFDL	0.44
C*08:02	2,629	2,639	VSLDNVLSTF	0.44
C*14:02	2,517	2,527	TYERHSLSHF	0.44
C*14:02	3,657	3,667	MVYMPASWVM	0.44
C*14:02	4,989	4,999	SKFYGGWHNM	0.44
C*17:01	1,556	1,566	ITFDNLKTLL	0.44
C*17:03	1,556	1,566	ITFDNLKTLL	0.44
A*01:01	2,893	2,903	VFSAVGNICY	0.45
A*01:01	4,203	4,213	KSDGTGTIYT	0.45
A*01:01	6,118	6,128	HGFELTSMKY	0.45
A*02:01	6,664	6,674	SQMEIDFLEL	0.45
A*02:05	5,745	5,755	TLEPEYFNSV	0.45
A*02:06	1,663	1,673	YPQVNGLTSI	0.45
A*03:01	906	916	YLFDESGEFK	0.45
A*03:01	1,300	1,310	VLTAVVIPTK	0.45
A*03:01	4,909	4,919	ARLYYDSMSY	0.45
A*11:01	5,259	5,269	LAIDAYPLTK	0.45
A*23:01	2,960	2,970	VLMDGSIIQF	0.45
A*23:01	5,182	5,192	VLYYQNNVFM	0.45
A*24:02	2,334	2,344	LAYILFTRFF	0.45
A*24:02	2,792	2,802	FYLIIPVHVM	0.45
A*24:02	3,135	3,145	VMFTPLVPFW	0.45

A*24:02	6,224	6,234	IEYPIIGDEL	0.45
A*25:01	1,772	1,782	AVMYMGTLSY	0.45
A*25:01	5,291	5,301	ELTGHMLDMY	0.45
A*26:01	1,631	1,641	TLRVEAFEYY	0.45
A*26:01	5,644	5,654	DALCEKALKY	0.45
A*26:01	5,776	5,786	EIVDTVSALV	0.45
A*29:02	5,544	5,554	TTYKLNVGDY	0.45
A*31:01	15	25	QLSLPVLQVR	0.45
A*31:01	4,637	4,647	LLMPILTLTR	0.45
A*32:01	2,312	2,322	IQITISSFKW	0.45
A*32:01	6,441	6,451	YKQFDTYNLW	0.45
A*33:01	5,493	5,503	HLSWEVGKPR	0.45
A*68:02	2,951	2,961	ESLRPDTRYV	0.45
A*68:02	6,563	6,573	DIAKKPTETI	0.45
B*07:02	2,225	2,235	SPNFSKLINI	0.45
B*13:02	5,225	5,235	YLPYPDPSRI	0.45
B*27:05	4,965	4,975	NRQFHQKLLK	0.45
B*35:01	3,371	3,381	FVRIQPGQTF	0.45
B*35:01	5,819	5,829	RPQIGVVREF	0.45
B*35:03	5,228	5,238	YPDPSRILGA	0.45
B*35:03	5,972	5,982	IPGIPKDMTY	0.45
B*39:01	1,111	1,121	SGHNLAKHCL	0.45
B*40:01	862	872	VELGTEVNEF	0.45
B*40:01	5,061	5,071	SEMVMCGGSL	0.45
B*40:01	5,271	5,281	NQEYADVFHL	0.45
B*40:02	2,651	2,661	VETKDVVECL	0.45
B*40:02	5,496	5,506	WEVGKPRPPL	0.45
B*45:01	2,555	2,565	EESSAKSASV	0.45
B*46:01	4,930	4,940	RNVIPTITQM	0.45
B*49:01	3,432	3,442	HMELPTGVHA	0.45
B*51:01	6,864	6,874	VPYNMRVIHF	0.45
B*53:01	884	894	QPVSELLTPL	0.45
B*53:01	1,695	1,705	FNPPALQDAY	0.45
B*57:01	1,497	1,507	SSSKTPEEHF	0.45
B*57:01	2,930	2,940	ASGKPVPYCY	0.45
B*57:01	3,884	3,894	LRVESSSKLW	0.45
C*01:02	2,222	2,232	YLKSPNFSKL	0.45
C*03:02	2,557	2,567	SSAKSASVYY	0.45
C*03:02	5,003	5,013	YSDVENPHLM	0.45
C*05:01	4,361	4,371	CANDPVGFTL	0.45
C*06:02	6,217	6,227	VKRVDWTIEY	0.45
C*08:02	2,091	2,101	ITEEVGHTDL	0.45
C*12:02	5,143	5,153	YAYLRKHFSM	0.45

C*14:02	2,901	2,911	CYTPSKLIEY	0.45
A*01:01	3,468	3,478	ITVNVLAWLY	0.46
A*01:01	4,223	4,233	DTPKGPKVKY	0.46
A*01:01	5,741	5,751	LTKGTLEPEY	0.46
A*01:01	6,054	6,064	YVDTPDNTDF	0.46
A*02:01	6,963	6,973	KLALGGSVAI	0.46
A*02:02	5,745	5,755	TLEPEYFNSV	0.46
A*02:06	3,334	3,344	FLVQAGNVQL	0.46
A*03:01	1,360	1,370	ILPSIISNEK	0.46
A*03:01	2,104	2,114	YVDNSSLTIK	0.46
A*11:01	591	601	ATNNLVVMAY	0.46
A*11:01	675	685	VQTFFKLVNK	0.46
A*23:01	4,633	4,643	SYYSLLMPIL	0.46
A*24:02	676	686	QTFFKLVNKF	0.46
A*25:01	176	186	ELNGGAYTRY	0.46
A*25:01	1,012	1,022	EVQPQLEMEL	0.46
A*32:01	6,777	6,787	VTIDYTEISF	0.46
A*32:01	7,025	7,035	YVMHANYIFW	0.46
A*33:01	1,314	1,324	TTEMLAKALR	0.46
A*33:01	4,901	4,911	FPFNKWGKAR	0.46
A*68:01	4,398	4,408	SADAQSFLNR	0.46
A*68:02	3,465	3,475	DTTITVNVLA	0.46
A*68:02	5,925	5,935	NVATLQAENV	0.46
A*68:02	6,046	6,056	NLVAVPTGYV	0.46
A*68:02	6,525	6,535	EVKILNNLGV	0.46
B*07:02	284	294	IQPRVEKKKL	0.46
B*15:01	1,855	1,865	GALLTKSSEY	0.46
B*15:01	5,181	5,191	SVLYYQNNVF	0.46
B*15:01	5,741	5,751	LTKGTLEPEY	0.46
B*15:03	3,076	3,086	FGEYSHVVAF	0.46
B*15:03	6,104	6,114	LKNLSDRVVF	0.46
B*27:05	5,981	5,991	YRRLISMMGF	0.46
B*35:03	6,805	6,815	SQAWQPGVAM	0.46
B*37:01	6,224	6,234	IEYPIIGDEL	0.46
B*39:01	1,385	1,395	AHAEETRKLM	0.46
B*39:01	6,352	6,362	NKHAFHTPAF	0.46
B*40:01	2,622	2,632	EAELAKNVSL	0.46
B*40:02	3,612	3,622	YENAFLPFAM	0.46
B*40:02	3,951	3,961	SEFSSLPSYA	0.46
B*44:02	975	985	HLEEEQEEDW	0.46
B*44:03	538	548	SEAARVVRSI	0.46
B*44:03	975	985	HLEEEQEEDW	0.46
B*44:03	4,120	4,130	SMDNSPNLAW	0.46

B*44:03	4, 263	4, 273	TEVPANSTVL	0. 46
B*49:01	1, 805	1, 815	QESPFVMMSA	0. 46
B*57:01	4, 580	4, 590	RQALLKTVQF	0. 46
B*58:01	478	488	ASFSASTSAF	0. 46
B*58:01	5, 796	5, 806	KSAQCFKMFY	0. 46
B*58:01	6, 298	6, 308	YSYATHSDKF	0. 46
C*01:02	4, 127	4, 137	LAWPLIVTAL	0. 46
C*02:02	2, 500	2, 510	TVKNGSIHLY	0. 46
C*02:02	5, 994	6, 004	YQVNGYPNMF	0. 46
C*03:02	3, 904	3, 914	LLAKDTTEAF	0. 46
C*07:01	5, 309	5, 319	TSRYWEPEFY	0. 46
C*07:02	6, 693	6, 703	VYGDFSHSQL	0. 46
C*08:02	4, 771	4, 781	YAADPAMHAA	0. 46
C*14:02	5, 182	5, 192	VLYYQNNVFM	0. 46
C*17:01	4, 519	4, 529	YTMADLVYAL	0. 46
C*17:03	4, 519	4, 529	YTMADLVYAL	0. 46
A*01:01	5, 440	5, 450	TCDWTNAGDY	0. 47
A*02:01	2, 222	2, 232	YLKSPNFSKL	0. 47
A*02:01	2, 576	2, 586	LLDQALVSDV	0. 47
A*02:01	5, 474	5, 484	FKLSYGIATV	0. 47
A*02:01	6, 447	6, 457	YNLWNTFTRL	0. 47
A*03:01	6, 354	6, 364	HAFHTPAFDK	0. 47
A*11:01	3, 183	3, 193	AALCTFLLNK	0. 47
A*23:01	2, 175	2, 185	PYFFTLLLQL	0. 47
A*23:01	3, 179	3, 189	TFEEAALCTF	0. 47
A*23:01	3, 756	3, 766	VMFLARGIVF	0. 47
A*23:01	5, 226	5, 236	LPYPDPSRIL	0. 47
A*25:01	490	500	TVKGLDYKAF	0. 47
A*25:01	1, 642	1, 652	TTDPSFLGRY	0. 47
A*25:01	4, 867	4, 877	LFVVEVVDKY	0. 47
A*25:01	5, 384	5, 394	CDVTDVTQLY	0. 47
A*29:02	1, 035	1, 045	GYLKLTDNVY	0. 47
A*29:02	2, 431	2, 441	IVNGVRRSFY	0. 47
A*31:01	5, 493	5, 503	HLSWEVGKPR	0. 47
A*33:01	4, 406	4, 416	NRVCGVSAAR	0. 47
A*68:02	673	683	ESVQTFFKLV	0. 47
A*68:02	1, 275	1, 285	LVSDIDITFL	0. 47
B*07:02	3, 646	3, 656	LPSLATVAYF	0. 47
B*15:01	6, 045	6, 055	VNLVAVPTGY	0. 47
B*15:01	6, 642	6, 652	VVQQLPETYF	0. 47
B*27:05	642	652	LRDGWEIVKF	0. 47
B*37:01	1, 140	1, 150	YENFNQHEVL	0. 47
B*38:01	5, 955	5, 965	THLSVDTKFK	0. 47

B*39:01	5,618	5,628	SHFAIGLALY	0.47
B*40:01	3,612	3,622	YENAFLPFAM	0.47
B*40:01	5,318	5,328	YEAMYTPHTV	0.47
B*40:01	6,944	6,954	KENDSKEGFF	0.47
B*40:02	1,010	1,020	IVEVQPQLEM	0.47
B*44:02	112	122	GEIPVAYRKV	0.47
B*44:02	6,419	6,429	NEYRLYLDAY	0.47
B*44:03	1,019	1,029	MELTPVVQTI	0.47
B*45:01	938	948	EEEFEPSTQY	0.47
B*45:01	4,917	4,927	SYEDQDALFA	0.47
B*49:01	1,195	1,205	FLEMKSEKQV	0.47
B*51:01	3,841	3,851	DAFKLNIKLL	0.47
B*51:01	6,520	6,530	IKPVPEVKIL	0.47
B*57:01	4,986	4,996	IGTSKFYGGW	0.47
C*02:02	4,714	4,724	FSTVFPLTSF	0.47
C*02:02	5,537	5,547	AVVYRGTTTY	0.47
C*03:02	5,537	5,547	AVVYRGTTTY	0.47
C*04:01	5,312	5,322	YWEPEFYEAM	0.47
C*04:43	5,312	5,322	YWEPEFYEAM	0.47
C*05:01	874	884	VVADAVIKTL	0.47
C*05:01	1,342	1,352	YTVEEAKTVL	0.47
C*05:01	1,420	1,430	VVDYGARFYF	0.47
C*05:01	5,905	5,915	MSDRDLYDKL	0.47
C*06:02	2,518	2,528	YERHSLSHFV	0.47
C*07:02	3,809	3,819	FRLTLGVYDY	0.47
C*15:02	1,556	1,566	ITFDNLKTLL	0.47
A*01:01	176	186	ELNGGAYTRY	0.48
A*02:02	3,494	3,504	TLNDFNLVAM	0.48
A*02:02	3,644	3,654	FLLPSLATVA	0.48
A*11:01	3,674	3,684	MVDTSLSGFK	0.48
A*25:01	603	613	GGVVQLTSQW	0.48
A*25:01	5,391	5,401	QLYLGGMSYY	0.48
A*25:01	6,430	6,440	MMISAGFSLW	0.48
A*25:01	6,799	6,809	YPKLQSSQAW	0.48
A*26:01	539	549	EAARVVRSIF	0.48
A*26:01	1,823	1,833	HGTFTCASEY	0.48
A*26:01	3,468	3,478	ITVNVLAWLY	0.48
A*29:02	1,540	1,550	VYYTSNPTTF	0.48
A*29:02	2,349	2,359	AAIMQLFFSY	0.48
A*29:02	3,171	3,181	VFNGVSFSTF	0.48
A*29:02	4,734	4,744	GVPFVVSTGY	0.48
A*33:01	1,949	1,959	LTGYKKPASR	0.48
A*68:01	807	817	MVTNNTFTLK	0.48

A*68:01	5,538	5,548	VVYRGTTTYK	0.48
A*68:02	1,430	1,440	YTSKTTVASL	0.48
B*07:02	2,688	2,698	MTPRDLGACI	0.48
B*13:02	2,567	2,577	SQLMCQPILL	0.48
B*13:02	6,805	6,815	SQAWQPGVAM	0.48
B*14:02	5,143	5,153	YAYLRKHFSM	0.48
B*15:01	3,105	3,115	FLPGVYSVIY	0.48
B*15:01	5,116	5,126	YVRNLQHRLY	0.48
B*15:01	6,412	6,422	AVCRHHANEY	0.48
B*27:05	3,209	3,219	YNRYLALYNK	0.48
B*27:05	5,309	5,319	TSRYWEPEFY	0.48
B*35:03	2,101	2,111	MAAYVDNSSL	0.48
B*37:01	5,200	5,210	TDLTKGPHEF	0.48
B*40:01	656	666	ACEIVGGQIV	0.48
B*40:02	749	759	TEVLTEEVVL	0.48
B*44:02	911	921	SGEFKLASHM	0.48
B*45:01	6,601	6,611	TEGSVKGLQP	0.48
B*51:01	1,162	1,172	DPIHSLRVCV	0.48
B*51:01	5,410	5,420	FPLCANGQVF	0.48
B*53:01	6,288	6,298	DKAYKIEELF	0.48
B*53:01	7,038	7,048	NPIQLSSYSL	0.48
B*57:01	1,878	1,888	YTTTIKPVTY	0.48
C*03:02	4,076	4,086	MVVIPDYNTY	0.48
C*05:01	2,091	2,101	ITEEVGHTDL	0.48
C*07:02	1,333	1,343	TYPGQGLNGY	0.48
C*07:04	6,693	6,703	VYGDFSHSQL	0.48
C*08:01	2,091	2,101	ITEEVGHTDL	0.48
C*14:02	3,723	3,733	VYKVYYGNAL	0.48
C*14:02	4,690	4,700	TYHPNCVNCL	0.48
C*15:02	881	891	KTLQPVSELL	0.48
C*16:01	404	414	IAFGGCVFSY	0.48
A*02:01	3,120	3,130	YLTNDVSFLA	0.49
A*02:05	1,542	1,552	YTSNPTTFHL	0.49
A*02:05	1,625	1,635	VLPNDDTLRV	0.49
A*02:06	5,225	5,235	YLPYPDPSRI	0.49
A*03:01	5,123	5,133	RLYECLYRNR	0.49
A*11:01	1,300	1,310	VLTAVVIPTK	0.49
A*23:01	3,104	3,114	SFLPGVYSVI	0.49
A*25:01	5,675	5,685	FKVNSTLEQY	0.49
A*26:01	676	686	QTFFKLVNKF	0.49
A*26:01	3,027	3,037	DAVNLLTNMF	0.49
A*29:02	240	250	WYTERSEKSY	0.49
A*29:02	4,563	4,573	VENPDILRVY	0.49

A*31:01	6,628	6,638	AVKTQFNYYK	0.49
A*32:01	3,631	3,641	MMFVKHKHAF	0.49
A*68:01	1,624	1,634	YVLPNDDTLR	0.49
A*68:01	3,703	3,713	RTVYDDGARR	0.49
A*68:01	4,277	4,287	FAVDAAKAYK	0.49
A*68:01	5,591	5,601	FSSNVANYQK	0.49
B*15:01	61	71	LPQLEQPYVF	0.49
B*15:01	4,008	4,018	KMADQAMTQM	0.49
B*18:01	6,294	6,304	EELFYSYATH	0.49
B*35:03	747	757	LPTEVLTEEV	0.49
B*38:01	5,618	5,628	SHFAIGLALY	0.49
B*39:01	6,490	6,500	TKVDGVDVEL	0.49
B*40:01	2,092	2,102	TEEVGHTDLM	0.49
B*40:02	6,657	6,667	LQEFKPRSQM	0.49
B*44:02	1,019	1,029	MELTPVVQTI	0.49
B*44:02	5,006	5,016	VENPHLMGWD	0.49
B*44:03	3,076	3,086	FGEYSHVVAF	0.49
B*44:03	3,910	3,920	TEAFEKMVSL	0.49
B*46:01	4,626	4,636	SGVPVVDSYY	0.49
B*46:01	4,868	4,878	FVVEVVDKYF	0.49
B*49:01	1,199	1,209	KSEKQVEQKI	0.49
B*49:01	1,728	1,738	GELGDVRETM	0.49
B*51:01	1,287	1,297	DAPYIVGDVV	0.49
B*51:01	1,453	1,463	MPLGYVTHGL	0.49
B*51:01	5,569	5,579	LVPQEHYVRI	0.49
C*02:02	5,725	5,735	YVYIGDPAQL	0.49
C*03:02	2,629	2,639	VSLDNVLSTF	0.49
C*03:04	76	86	ARTAPHGHVM	0.49
C*08:02	4,221	4,231	VTDTPKGPKV	0.49
C*14:02	478	488	ASFSASTSAF	0.49
C*14:02	5,391	5,401	QLYLGGMSYY	0.49
C*15:02	5,021	5,031	RAMPNMLRIM	0.49
C*17:01	2,605	2,615	STFNVPMEKL	0.49
C*17:01	3,910	3,920	TEAFEKMVSL	0.49
C*17:03	2,605	2,615	STFNVPMEKL	0.49
C*17:03	3,910	3,920	TEAFEKMVSL	0.49
A*01:01	612	622	WLTNIFGTVY	0.5
A*01:01	5,272	5,282	QEYADVFHLY	0.5
A*02:05	1,442	1,452	TLNDLNETLV	0.5
A*02:05	1,663	1,673	YPQVNGLTSI	0.5
A*02:06	569	579	ILDGISQYSL	0.5
A*02:06	3,373	3,383	RIQPGQTFSV	0.5
A*02:06	6,765	6,775	SQDLSVVSKV	0.5

A*11:01	4,010	4,020	ADQAMTQMYK	0.5
A*11:01	5,797	5,807	SAQCFKMFYK	0.5
A*24:02	2,390	2,400	IFFASFYYVW	0.5
A*24:02	5,182	5,192	VLYYQNNVFM	0.5
A*25:01	1,507	1,517	IETISLAGSY	0.5
A*26:01	2,430	2,440	TIVNGVRRSF	0.5
A*29:02	6,641	6,651	GVVQQLPETY	0.5
A*31:01	162	172	NTKHSSGVTR	0.5
A*31:01	2,467	2,477	STFISDEVAR	0.5
A*31:01	6,880	6,890	GVAPGTAVLR	0.5
A*33:01	5,532	5,542	GDYGDAVVYR	0.5
A*68:01	4,651	4,661	ESHVDTDLTK	0.5
A*68:01	4,943	4,953	YAISAKNRAR	0.5
B*08:01	2,609	2,619	VPMEKLKTLV	0.5
B*13:02	454	464	LQKEKVNINI	0.5
B*13:02	3,074	3,084	RAFGEYSHVV	0.5
B*15:01	7,036	7,046	NTNPIQLSSY	0.5
B*27:05	4,576	4,586	GERVRQALLK	0.5
B*38:01	5,881	5,891	TAHSCNVNRF	0.5
B*39:01	6,805	6,815	SQAWQPGVAM	0.5
B*44:03	6,666	6,676	MEIDFLELAM	0.5
B*45:01	2,057	2,067	VENPTIQKDV	0.5
B*49:01	830	840	VIEVQGYKSV	0.5
B*51:01	5	15	VPGFNEKTHV	0.5
B*51:01	2,935	2,945	VPYCYDTNVL	0.5
B*53:01	731	741	MPLKAPKEII	0.5
B*53:01	1,652	1,662	MSALNHTKKW	0.5
B*53:01	2,379	2,389	MAPISAMVRM	0.5
B*53:01	2,473	2,483	EVARDLSLQF	0.5
B*53:01	3,617	3,627	LPFAMGIIAM	0.5
B*53:01	5,487	5,497	LSDRELHLSW	0.5
B*58:01	404	414	IAFGGCVFSY	0.5
B*58:01	567	577	ITILDGISQY	0.5
B*58:01	1,244	1,254	VTTTLEETKF	0.5
B*58:01	4,211	4,221	YTELEPPCRF	0.5
B*58:01	6,506	6,516	LPVNVAFELW	0.5
C*03:04	3,953	3,963	FSSLPSYAAF	0.5
C*05:01	843	853	FELDERIDKV	0.5
C*05:01	907	917	LFDESGEFKL	0.5
C*05:01	4,630	4,640	VVDSYYSLLM	0.5
C*07:01	5,626	5,636	LYYPSARIVY	0.5
C*07:02	1,903	1,913	NYYKKDNSYF	0.5
C*07:02	5,226	5,236	LPYPDPSRIL	0.5

C*08:02	1,556	1,566	ITFDNLKTLL	0.5
C*12:02	7,036	7,046	NTNPIQLSSY	0.5
C*12:03	3,371	3,381	FVRIQPGQTF	0.5
C*12:03	3,910	3,920	TEAFEKMVSL	0.5
C*15:02	3,579	3,589	RTIKGTHHWL	0.5
A*01:01	1,326	1,336	PTDNYITTYP	0.51
A*01:01	1,810	1,820	VMMSAPPAQY	0.51
A*01:01	4,916	4,926	MSYEDQDALF	0.51
A*01:01	5,304	5,314	LTNDNTSRYW	0.51
A*02:01	2,336	2,346	YILFTRFFYV	0.51
A*02:01	2,869	2,879	FVVPGLPGTI	0.51
A*02:02	2,661	2,671	KLSHQSDIEV	0.51
A*02:02	4,126	4,136	NLAWPLIVTA	0.51
A*02:02	6,113	6,123	FVLWAHGFEL	0.51
A*02:02	6,838	6,848	TLPKGIMMNV	0.51
A*02:05	445	455	GLNDNLLEIL	0.51
A*02:05	1,291	1,301	IVGDVVQEGV	0.51
A*02:06	1,202	1,212	KQVEQKIAEI	0.51
A*03:01	813	823	FTLKGGAPTK	0.51
A*03:01	6,737	6,747	ITDAQTGSSK	0.51
A*23:01	5,279	5,289	HLYLQYIRKL	0.51
A*24:02	3,119	3,129	FYLTNDVSFL	0.51
A*25:01	3,740	3,750	ALIISVTSNY	0.51
A*25:01	6,067	6,077	SAKPPPGDQF	0.51
A*26:01	2,557	2,567	SSAKSASVYY	0.51
A*29:02	1,895	1,905	TEIDPKLDNY	0.51
A*29:02	3,140	3,150	LVPFWITIAY	0.51
A*29:02	4,009	4,019	MADQAMTQMY	0.51
A*31:01	3,570	3,580	GVTFQSAVKR	0.51
A*31:01	4,049	4,059	ALNNIINNAR	0.51
A*32:01	3,944	3,954	ATLQAIASEF	0.51
B*13:02	560	570	RVLQKAAITI	0.51
B*13:02	2,062	2,072	IQKDVLECNV	0.51
B*14:02	5,572	5,582	QEHYVRITGL	0.51
B*15:01	1,803	1,813	VQQESPFVMM	0.51
B*15:01	2,327	2,337	GLVAEWFLAY	0.51
B*15:01	4,468	4,478	VVKRHTFSNY	0.51
B*15:03	5,792	5,802	AHKDKSAQCF	0.51
B*18:01	375	385	NSEVGPEHSL	0.51
B*18:01	1,140	1,150	YENFNQHEVL	0.51
B*18:01	1,503	1,513	EEHFIETISL	0.51
B*37:01	2,035	2,045	SEDAQGMDNL	0.51
B*37:01	2,721	2,731	KDFMSLSEQL	0.51

B*37:01	4,848	4,858	SDYDYYRYNL	0.51
B*37:01	6,716	6,726	KESPFELEDF	0.51
B*39:01	6,664	6,674	SQMEIDFLEL	0.51
B*40:01	112	122	GEIPVAYRKV	0.51
B*40:01	232	242	REHEHEIAWY	0.51
B*45:01	1,829	1,839	ASEYTGNYQC	0.51
B*45:01	3,937	3,947	EEMLDNRATL	0.51
B*46:01	4,909	4,919	ARLYYDSMSY	0.51
B*49:01	639	649	VEFLRDGWEI	0.51
B*51:01	3,141	3,151	VPFWITIAYI	0.51
B*53:01	3,140	3,150	LVPFWITIAY	0.51
B*53:01	4,639	4,649	MPILTLTRAL	0.51
B*53:01	5,005	5,015	DVENPHLMGW	0.51
B*57:01	3,950	3,960	ASEFSSLPSY	0.51
B*57:01	4,120	4,130	SMDNSPNLAW	0.51
C*02:02	1,542	1,552	YTSNPTTFHL	0.51
C*02:02	4,868	4,878	FVVEVVDKYF	0.51
C*02:02	5,303	5,313	MLTNDNTSRY	0.51
C*03:02	2,440	2,450	YVYANGGKGF	0.51
C*03:02	6,154	6,164	YACWHHSIGF	0.51
C*03:02	6,432	6,442	ISAGFSLWVY	0.51
C*03:02	6,651	6,661	FTQSRNLQEF	0.51
C*07:01	642	652	LRDGWEIVKF	0.51
C*07:01	2,953	2,963	LRPDTRYVLM	0.51
C*07:04	5,226	5,236	LPYPDPSRIL	0.51
C*07:04	5,288	5,298	LHDELTGHML	0.51
C*14:02	1,427	1,437	FYFYTSKTTV	0.51
C*14:02	2,172	2,182	NYMPYFFTLL	0.51
A*02:01	6,838	6,848	TLPKGIMMNV	0.52
A*02:02	1,275	1,285	LVSDIDITFL	0.52
A*02:02	2,109	2,119	SLTIKKPNEL	0.52
A*02:02	3,090	3,100	FLMSFTVLCL	0.52
A*02:02	4,135	4,145	ALRANSAVKL	0.52
A*02:06	874	884	VVADAVIKTL	0.52
A*02:06	3,643	3,653	LFLLPSLATV	0.52
A*03:01	1,403	1,413	IVSTIQRKYK	0.52
A*03:01	4,971	4,981	KLLKSIAATR	0.52
A*11:01	488	498	VETVKGLDYK	0.52
A*11:01	3,898	3,908	QLHNDILLAK	0.52
A*23:01	1,817	1,827	AQYELKHGTF	0.52
A*24:02	2,249	2,259	IYSTAALGVL	0.52
A*24:02	2,274	2,284	GYLNSTNVTI	0.52
A*26:01	404	414	IAFGGCVFSY	0.52

A*26:01	6,946	6,956	NDSKEGFFTY	0.52
A*29:02	4,517	4,527	TKYTMADLVY	0.52
A*29:02	4,661	4,671	PYIKWDLLKY	0.52
A*31:01	541	551	ARVVRSIFSR	0.52
A*68:01	1,120	1,130	LHVVGPNVNK	0.52
A*68:01	2,311	2,321	TIQITISSFK	0.52
A*68:02	5,276	5,286	DVFHLYLQYI	0.52
B*13:02	1,019	1,029	MELTPVVQTI	0.52
B*15:01	4,930	4,940	RNVIPTITQM	0.52
B*18:01	953	963	DDYQGKPLEF	0.52
B*27:05	2,386	2,396	VRMYIFFASF	0.52
B*27:05	7,033	7,043	FWRNTNPIQL	0.52
B*35:01	88	98	LVAELEGIQY	0.52
B*35:01	3,246	3,256	FSNSGSDVLY	0.52
B*35:01	5,077	5,087	TSSGDATTAY	0.52
B*35:01	7,036	7,046	NTNPIQLSSY	0.52
B*35:03	2,608	2,618	NVPMEKLKTL	0.52
B*37:01	242	252	TERSEKSYEL	0.52
B*37:01	6,547	6,557	RDAPAHISTI	0.52
B*38:01	5,129	5,139	YRNRDVDTDF	0.52
B*39:01	6,259	6,269	LHDIGNPKAI	0.52
B*40:02	268	278	GECPNFVFPL	0.52
B*40:02	911	921	SGEFKLASHM	0.52
B*44:02	1,503	1,513	EEHFIETISL	0.52
B*44:02	1,862	1,872	SEYKGPITDV	0.52
B*44:02	2,057	2,067	VENPTIQKDV	0.52
B*44:03	2,057	2,067	VENPTIQKDV	0.52
B*44:03	5,006	5,016	VENPHLMGWD	0.52
B*45:01	4,023	4,033	SEDKRAKVTS	0.52
B*46:01	2,500	2,510	TVKNGSIHLY	0.52
B*46:01	2,979	2,989	RVVTTFDSEY	0.52
B*46:01	5,303	5,313	MLTNDNTSRY	0.52
B*46:01	5,725	5,735	YVYIGDPAQL	0.52
B*49:01	4,454	4,464	QEKDEDDNLI	0.52
B*49:01	6,458	6,468	SLENVAFNVV	0.52
B*51:01	61	71	LPQLEQPYVF	0.52
B*51:01	4,639	4,649	MPILTLTRAL	0.52
B*53:01	2,380	2,390	APISAMVRMY	0.52
B*53:01	3,660	3,670	MPASWVMRIM	0.52
B*53:01	4,009	4,019	MADQAMTQMY	0.52
B*53:01	4,341	4,351	HPNPKGFCDL	0.52
B*53:01	4,628	4,638	VPVVDSYYSL	0.52
B*53:01	6,148	6,158	STASDTYACW	0.52

B*57:01	567	577	ITILDGISQY	0.52
B*57:01	6,432	6,442	ISAGFSLWVY	0.52
B*58:01	4,714	4,724	FSTVFPLTSF	0.52
B*58:01	6,882	6,892	APGTAVLRQW	0.52
C*01:02	2,173	2,183	YMPYFFTLLL	0.52
C*05:01	136	146	YGADLKSFDL	0.52
C*07:04	76	86	ARTAPHGHVM	0.52
C*08:02	907	917	LFDESGEFKL	0.52
C*08:02	2,582	2,592	VSDVGDSAEV	0.52
C*08:02	5,002	5,012	VYSDVENPHL	0.52
C*12:03	567	577	ITILDGISQY	0.52
C*14:02	2,841	2,851	TWFSQRGGSY	0.52
C*17:01	586	596	FTSDLATNNL	0.52
C*17:03	586	596	FTSDLATNNL	0.52
A*01:01	1,823	1,833	HGTFTCASEY	0.53
A*01:01	2,258	2,268	LMSNLGMPSY	0.53
A*02:01	1,990	2,000	LLHKPIVWHV	0.53
A*02:02	3,120	3,130	YLTNDVSFLA	0.53
A*02:05	6,250	6,260	ALLADKFPVL	0.53
A*02:05	6,615	6,625	KQASLNGVTL	0.53
A*03:01	5,168	5,178	ASQGLVASIK	0.53
A*23:01	1,623	1,633	FYVLPNDDTL	0.53
A*23:01	4,690	4,700	TYHPNCVNCL	0.53
A*23:01	5,910	5,920	LYDKLQFTSL	0.53
A*25:01	264	274	DIFNGECPNF	0.53
A*25:01	5,116	5,126	YVRNLQHRLY	0.53
A*25:01	5,619	5,629	HFAIGLALYY	0.53
A*26:01	1,274	1,284	TLVSDIDITF	0.53
A*26:01	1,880	1,890	TTIKPVTYKL	0.53
A*26:01	5,741	5,751	LTKGTLEPEY	0.53
A*29:02	3,107	3,117	PGVYSVIYLY	0.53
A*29:02	3,779	3,789	NTLQCIMLVY	0.53
A*31:01	1,699	1,709	ALQDAYYRAR	0.53
A*31:01	4,493	4,503	VAKHDFFKFR	0.53
A*32:01	1,186	1,196	NLYDKLVSSF	0.53
A*32:01	2,323	2,333	LTAFGLVAEW	0.53
A*32:01	3,466	3,476	TTITVNVLAW	0.53
A*33:01	2,185	2,195	CTFTRSTNSR	0.53
A*33:01	4,637	4,647	LLMPILTLTR	0.53
A*33:01	6,056	6,066	DTPDNTDFSR	0.53
A*68:01	536	546	FASEAARVVR	0.53
A*68:01	4,818	4,828	DFAVSKGFFK	0.53
A*68:02	4,094	4,104	FTYASALWEI	0.53

A*68:02	6,152	6,162	DTYACWHHSI	0.53
B*07:02	2,114	2,124	KPNELSRVLG	0.53
B*15:01	6,170	6,180	FMIDVQQWGF	0.53
B*15:01	6,651	6,661	FTQSRNLQEF	0.53
B*15:03	912	922	GEFKLASHMY	0.53
B*18:01	3,910	3,920	TEAFEKMVSL	0.53
B*18:01	5,469	5,479	ATEETFKLSY	0.53
B*35:03	6,271	6,281	VPQADVEWKF	0.53
B*38:01	3,430	3,440	MHHMELPTGV	0.53
B*39:01	1,584	1,594	LHTQVVDMSM	0.53
B*39:01	3,371	3,381	FVRIQPGQTF	0.53
B*44:03	1,507	1,517	IETISLAGSY	0.53
B*44:03	6,497	6,507	VELFENKTTL	0.53
B*46:01	2,964	2,974	GSIIQFPNTY	0.53
B*49:01	6,028	6,038	REAVGTNLPL	0.53
B*57:01	478	488	ASFSASTSAF	0.53
B*57:01	3,125	3,135	VSFLAHIQWM	0.53
B*57:01	3,601	3,611	QSTQWSLFFF	0.53
B*57:01	5,401	5,411	CKSHKPPISF	0.53
B*58:01	3,950	3,960	ASEFSSLPSY	0.53
C*02:02	1,615	1,625	HNSHEGKTFY	0.53
C*02:02	2,629	2,639	VSLDNVLSTF	0.53
C*03:02	478	488	ASFSASTSAF	0.53
C*03:02	5,619	5,629	HFAIGLALYY	0.53
C*03:04	6,067	6,077	SAKPPPGDQF	0.53
C*04:01	1,158	1,168	IFGADPIHSL	0.53
C*04:01	4,730	4,740	IFVDGVPFVV	0.53
C*04:43	1,158	1,168	IFGADPIHSL	0.53
C*04:43	4,730	4,740	IFVDGVPFVV	0.53
C*05:01	4,211	4,221	YTELEPPCRF	0.53
C*07:01	2,509	2,519	YFDKAGQKTY	0.53
C*07:02	5,129	5,139	YRNRDVDTDF	0.53
C*08:01	569	579	ILDGISQYSL	0.53
C*12:02	6,298	6,308	YSYATHSDKF	0.53
C*14:02	1,623	1,633	FYVLPNDDTL	0.53
C*14:02	5,273	5,283	EYADVFHLYL	0.53
C*14:02	5,910	5,920	LYDKLQFTSL	0.53
A*01:01	1,792	1,802	CTCGKQATKY	0.54
A*01:01	4,762	4,772	RLSFKELLVY	0.54
A*01:01	4,847	4,857	ISDYDYYRYN	0.54
A*01:01	5,544	5,554	TTYKLNVGDY	0.54
A*01:01	6,946	6,956	NDSKEGFFTY	0.54
A*02:01	2,661	2,671	KLSHQSDIEV	0.54

A*02:01	3, 187	3, 197	TFLLNKEMYL	0. 54
A*02:02	1, 801	1, 811	YLVQQESPFV	0. 54
A*02:02	6, 963	6, 973	KLALGGSVAI	0. 54
A*02:05	6, 879	6, 889	KGVAPGTAVL	0. 54
A*03:01	2, 221	2, 231	NYLKSPNFSK	0. 54
A*03:01	4, 814	4, 824	KDFYDFAVSK	0. 54
A*11:01	1, 856	1, 866	ALLTKSSEYK	0. 54
A*11:01	4, 933	4, 943	IPTITQMNLK	0. 54
A*23:01	134	144	HSYGADLKSF	0. 54
A*23:01	3, 112	3, 122	VIYLYLTFYL	0. 54
A*23:01	3, 119	3, 129	FYLTNDVSFL	0. 54
A*24:02	1, 549	1, 559	FHLDGEVITF	0. 54
A*24:02	2, 006	2, 016	ATYKPNTWCI	0. 54
A*24:02	4, 803	4, 813	AFQTVKPGNF	0. 54
A*26:01	4, 148	4, 158	ELSPVALRQM	0. 54
A*26:01	5, 243	5, 253	DIVKTDGTLM	0. 54
A*29:02	1, 766	1, 776	TLKGVEAVMY	0. 54
A*29:02	6, 045	6, 055	VNLVAVPTGY	0. 54
A*32:01	6, 491	6, 501	KVDGVDVELF	0. 54
A*68:01	68	78	YVFIKRSDAR	0. 54
A*68:01	1, 525	1, 535	STQLGIEFLK	0. 54
A*68:01	4, 845	4, 855	AAISDYDYYR	0. 54
A*68:01	5, 481	5, 491	ATVREVLSDR	0. 54
A*68:02	2, 157	2, 167	TTTNIVTRCL	0. 54
A*68:02	5, 586	5, 596	NISDEFSSNV	0. 54
A*68:02	5, 725	5, 735	YVYIGDPAQL	0. 54
B*07:02	6, 549	6, 559	APAHISTIGV	0. 54
B*13:02	4, 570	4, 580	RVYANLGERV	0. 54
B*13:02	4, 729	4, 739	KIFVDGVPFV	0. 54
B*13:02	5, 846	5, 856	SQNAVASKIL	0. 54
B*14:02	6, 416	6, 426	HHANEYRLYL	0. 54
B*15:01	7, 068	7, 078	GQINDMILSL	0. 54
B*27:05	847	857	ERIDKVLNEK	0. 54
B*27:05	4, 135	4, 145	ALRANSAVKL	0. 54
B*27:05	4, 470	4, 480	KRHTFSNYQH	0. 54
B*27:05	6, 517	6, 527	KRNIKPVPEV	0. 54
B*35:01	4, 734	4, 744	GVPFVVSTGY	0. 54
B*35:01	6, 805	6, 815	SQAWQPGVAM	0. 54
B*39:01	76	86	ARTAPHGHVM	0. 54
B*39:01	3, 430	3, 440	MHHMELPTGV	0. 54
B*39:01	6, 713	6, 723	KRFKESPFEL	0. 54
B*40:01	911	921	SGEFKLASHM	0. 54
B*40:01	1, 028	1, 038	IEVNSFSGYL	0. 54

B*40:02	862	872	VELGTEVNEF	0.54
B*40:02	1,016	1,026	QLEMELTPVV	0.54
B*40:02	7,066	7,076	KEGQINDMIL	0.54
B*44:02	55	65	EVEKGVLPQL	0.54
B*44:02	375	385	NSEVGPEHSL	0.54
B*44:02	524	534	GEQKSILSPL	0.54
B*44:02	1,465	1,475	EEAARYMRSL	0.54
B*44:02	2,517	2,527	TYERHSLSHF	0.54
B*44:03	327	337	GETSWQTGDF	0.54
B*49:01	524	534	GEQKSILSPL	0.54
B*49:01	2,620	2,630	TAEAELAKNV	0.54
B*51:01	5,319	5,329	EAMYTPHTVL	0.54
B*57:01	2,362	2,372	HFISNSWLMW	0.54
B*57:01	4,211	4,221	YTELEPPCRF	0.54
B*57:01	4,959	4,969	ICSTMTNRQF	0.54
B*57:01	5,040	5,050	TTCCSLSHRF	0.54
C*05:01	6,693	6,703	VYGDFSHSQL	0.54
C*07:01	397	407	LRKGGRTIAF	0.54
C*07:01	5,129	5,139	YRNRDVDTDF	0.54
C*07:02	599	609	AYITGGVVQL	0.54
C*12:02	5,003	5,013	YSDVENPHLM	0.54
C*16:01	2,557	2,567	SSAKSASVYY	0.54
C*16:01	5,003	5,013	YSDVENPHLM	0.54
C*17:01	1,342	1,352	YTVEEAKTVL	0.54
C*17:01	3,103	3,113	YSFLPGVYSV	0.54
C*17:03	1,342	1,352	YTVEEAKTVL	0.54
C*17:03	3,103	3,113	YSFLPGVYSV	0.54
A*01:01	1,276	1,286	VSDIDITFLK	0.55
A*01:01	5,531	5,541	KGDYGDAVVY	0.55
A*02:01	5,287	5,297	KLHDELTGHM	0.55
A*02:01	5,745	5,755	TLEPEYFNSV	0.55
A*02:02	1,095	1,105	YIATNGPLKV	0.55
A*02:02	1,990	2,000	LLHKPIVWHV	0.55
A*02:05	1,202	1,212	KQVEQKIAEI	0.55
A*02:05	2,062	2,072	IQKDVLECNV	0.55
A*02:06	1,625	1,635	VLPNDDTLRV	0.55
A*03:01	120	130	KVLLRKNGNK	0.55
A*03:01	789	799	MLLEIKDTEK	0.55
A*03:01	3,183	3,193	AALCTFLLNK	0.55
A*11:01	6,558	6,568	VCSMTDIAKK	0.55
A*23:01	3,610	3,620	FLYENAFLPF	0.55
A*23:01	4,803	4,813	AFQTVKPGNF	0.55
A*23:01	5,626	5,636	LYYPSARIVY	0.55

A*24:02	1,594	1,604	TYGQQFGPTY	0.55
A*25:01	1,060	1,070	TVVVNAANVY	0.55
A*25:01	5,544	5,554	TTYKLNVGDY	0.55
A*26:01	940	950	EFEPSTQYEY	0.55
A*26:01	1,482	1,492	VSSPDAVTAY	0.55
A*29:02	1,448	1,458	ETLVTMPLGY	0.55
A*31:01	6,813	6,823	AMPNLYKMQR	0.55
A*33:01	5,915	5,925	QFTSLEIPRR	0.55
A*68:01	504	514	ESCGNFKVTK	0.55
A*68:02	4,562	4,572	FVENPDILRV	0.55
B*08:01	1,053	1,063	EAKKVKPTVV	0.55
B*08:01	3,632	3,642	MFVKHKHAFL	0.55
B*08:01	6,844	6,854	MMNVAKYTQL	0.55
B*13:02	1,473	1,483	SLKVPATVSV	0.55
B*15:01	2,317	2,327	SSFKWDLTAF	0.55
B*15:03	700	710	LKALNLGETF	0.55
B*18:01	3,937	3,947	EEMLDNRATL	0.55
B*27:05	76	86	ARTAPHGHVM	0.55
B*27:05	3,271	3,281	FRKMAFPSGK	0.55
B*35:01	1,453	1,463	MPLGYVTHGL	0.55
B*35:01	4,276	4,286	AFAVDAAKAY	0.55
B*35:01	5,500	5,510	KPRPPLNRNY	0.55
B*35:01	5,663	5,673	IPARARVECF	0.55
B*37:01	6,944	6,954	KENDSKEGFF	0.55
B*40:01	1,873	1,883	YKENSYTTTI	0.55
B*40:01	3,180	3,190	FEEAALCTFL	0.55
B*40:01	4,211	4,221	YTELEPPCRF	0.55
B*44:02	3,076	3,086	FGEYSHVVAF	0.55
B*44:03	55	65	EVEKGVLPQL	0.55
B*45:01	100	110	SGETLGVLVP	0.55
B*45:01	112	122	GEIPVAYRKV	0.55
B*46:01	3,631	3,641	MMFVKHKHAF	0.55
B*46:01	5,994	6,004	YQVNGYPNMF	0.55
B*49:01	742	752	LEGETLPTEV	0.55
B*49:01	2,865	2,875	REVGFVVPGL	0.55
B*49:01	4,563	4,573	VENPDILRVY	0.55
B*53:01	3,466	3,476	TTITVNVLAW	0.55
B*53:01	4,189	4,199	LLSDLQDLKW	0.55
B*53:01	4,490	4,500	CPAVAKHDFF	0.55
B*53:01	6,430	6,440	MMISAGFSLW	0.55
B*57:01	722	732	KSREETGLLM	0.55
B*57:01	2,710	2,720	KSHNIALIWN	0.55
C*02:02	76	86	ARTAPHGHVM	0.55

C*12:02	134	144	HSYGADLKSF	0.55
C*14:02	1,519	1,529	WSYSGQSTQL	0.55
C*14:02	3,825	3,835	FRYMNSQGLL	0.55
C*14:02	4,670	4,680	YDFTEERLKL	0.55
C*14:02	7,044	7,054	SYSLFDMSKF	0.55
A*02:01	60	70	VLPQLEQPYV	0.56
A*02:01	3,866	3,876	KMSDVKCTSV	0.56
A*02:02	874	884	VVADAVIKTL	0.56
A*02:02	3,317	3,327	MLNPNYEDLL	0.56
A*02:05	3,336	3,346	VQAGNVQLRV	0.56
A*02:05	6,637	6,647	KKVDGVVQQL	0.56
A*02:06	3,051	3,061	SIVAGGIVAI	0.56
A*02:06	5,745	5,755	TLEPEYFNSV	0.56
A*03:01	3,839	3,849	SIDAFKLNIK	0.56
A*03:01	5,459	5,469	LKLFAAETLK	0.56
A*03:01	6,086	6,096	GLPWNVVRIK	0.56
A*03:01	6,583	6,593	RVDGQVDLFR	0.56
A*11:01	906	916	YLFDESGEFK	0.56
A*11:01	1,301	1,311	LTAVVIPTKK	0.56
A*11:01	6,060	6,070	NTDFSRVSAK	0.56
A*11:01	6,674	6,684	AMDEFIERYK	0.56
A*23:01	6,441	6,451	YKQFDTYNLW	0.56
A*25:01	3,578	3,588	KRTIKGTHHW	0.56
A*25:01	7,025	7,035	YVMHANYIFW	0.56
A*26:01	3,246	3,256	FSNSGSDVLY	0.56
A*26:01	4,510	4,520	HISRQRLTKY	0.56
A*26:01	6,357	6,367	HTPAFDKSAF	0.56
A*29:02	1,869	1,879	TDVFYKENSY	0.56
A*29:02	4,626	4,636	SGVPVVDSYY	0.56
A*31:01	3,223	3,233	SGAMDTTSYR	0.56
A*33:01	217	227	EQLDFIDTKR	0.56
A*33:01	533	543	LYAFASEAAR	0.56
A*33:01	4,146	4,156	NNELSPVALR	0.56
A*33:01	5,568	5,578	TLVPQEHYVR	0.56
A*68:01	2,427	2,437	ECTTIVNGVR	0.56
B*07:02	731	741	MPLKAPKEII	0.56
B*07:02	4,628	4,638	VPVVDSYYSL	0.56
B*07:02	5,611	5,621	GPPGTGKSHF	0.56
B*07:02	5,921	5,931	IPRRNVATLQ	0.56
B*07:02	6,814	6,824	MPNLYKMQRM	0.56
B*08:01	2,774	2,784	WLKQLIKVTL	0.56
B*08:01	3,980	3,990	VVLKKLKKSL	0.56
B*15:01	1,419	1,429	GVVDYGARFY	0.56

B*15:01	1,869	1,879	TDVFYKENSY	0.56
B*18:01	6,497	6,507	VELFENKTTL	0.56
B*27:05	6,421	6,431	YRLYLDAYNM	0.56
B*37:01	1,781	1,791	YEQFKKGVQI	0.56
B*38:01	3,997	4,007	DRDAAMQRKL	0.56
B*39:01	2,520	2,530	RHSLSHFVNL	0.56
B*40:01	248	258	SYELQTPFEI	0.56
B*40:01	632	642	EEKFKEGVEF	0.56
B*40:02	843	853	FELDERIDKV	0.56
B*40:02	6,675	6,685	MDEFIERYKL	0.56
B*44:02	3,910	3,920	TEAFEKMVSL	0.56
B*44:02	3,951	3,961	SEFSSLPSYA	0.56
B*44:03	5,702	5,712	FDEISMATNY	0.56
B*46:01	5,116	5,126	YVRNLQHRLY	0.56
B*49:01	1,139	1,149	AYENFNQHEV	0.56
B*51:01	6,563	6,573	DIAKKPTETI	0.56
B*53:01	3,371	3,381	FVRIQPGQTF	0.56
B*57:01	6,298	6,308	YSYATHSDKF	0.56
C*08:02	3,670	3,680	TWLDMVDTSL	0.56
C*12:03	1,812	1,822	MSAPPAQYEL	0.56
C*14:02	1,975	1,985	IDYKHYTPSF	0.56
C*14:02	3,163	3,173	SNYLKRRVVF	0.56
C*14:02	3,631	3,641	MMFVKHKHAF	0.56
C*14:02	3,705	3,715	VYDDGARRVW	0.56
C*15:02	2,881	2,891	TTNGDFLHFL	0.56
C*16:01	3,246	3,256	FSNSGSDVLY	0.56
C*16:01	5,021	5,031	RAMPNMLRIM	0.56
A*02:01	1,283	1,293	FLKKDAPYIV	0.57
A*02:01	2,785	2,795	FLFVAAIFYL	0.57
A*02:01	5,460	5,470	KLFAAETLKA	0.57
A*02:05	569	579	ILDGISQYSL	0.57
A*02:05	6,700	6,710	SQLGGLHLLI	0.57
A*02:06	843	853	FELDERIDKV	0.57
A*02:06	6,637	6,647	KKVDGVVQQL	0.57
A*02:06	6,754	6,764	LLLDDFVEII	0.57
A*03:01	4,035	4,045	QIMLFTMLRK	0.57
A*11:01	38	48	VLSEARQHLK	0.57
A*11:01	5,028	5,038	RIMASLVLAR	0.57
A*23:01	5,143	5,153	YAYLRKHFSM	0.57
A*24:02	3,104	3,114	SFLPGVYSVI	0.57
A*24:02	3,610	3,620	FLYENAFLPF	0.57
A*26:01	1,131	1,141	EDIQLLKSAY	0.57
A*26:01	6,571	6,581	TICAPLTVFF	0.57

A*26:01	6, 673	6, 683	LAMDEFIERY	0. 57
A*29:02	487	497	FVETVKGLDY	0. 57
A*29:02	5, 309	5, 319	TSRYWEPEFY	0. 57
A*32:01	2, 605	2, 615	STFNVPMEKL	0. 57
A*32:01	4, 092	4, 102	TTFTYASALW	0. 57
A*32:01	5, 600	5, 610	KVGMQKYSTL	0. 57
A*33:01	2, 378	2, 388	QMAPISAMVR	0. 57
A*33:01	4, 493	4, 503	VAKHDFFKFR	0. 57
A*68:01	3, 263	3, 273	TSAVLQSGFR	0. 57
A*68:01	5, 493	5, 503	HLSWEVGKPR	0. 57
B*35:03	781	791	TPVCINGLML	0. 57
B*35:03	2, 199	2, 209	MPTTIAKNTV	0. 57
B*38:01	5, 294	5, 304	GHMLDMYSVM	0. 57
B*38:01	6, 699	6, 709	HSQLGGLHLL	0. 57
B*40:01	2, 944	2, 954	LEGSVAYESL	0. 57
B*40:02	376	386	SEVGPEHSLA	0. 57
B*40:02	2, 622	2, 632	EAELAKNVSL	0. 57
B*44:02	9	19	NEKTHVQLSL	0. 57
B*44:03	524	534	GEQKSILSPL	0. 57
B*44:03	1, 131	1, 141	EDIQLLKSAY	0. 57
B*45:01	723	733	SREETGLLMP	0. 57
B*45:01	1, 214	1, 224	EEVKPFITES	0. 57
B*45:01	5, 495	5, 505	SWEVGKPRPP	0. 57
B*51:01	196	206	YPLECIKDLL	0. 57
B*51:01	3, 617	3, 627	LPFAMGIIAM	0. 57
B*53:01	1, 696	1, 706	NPPALQDAYY	0. 57
B*53:01	6, 520	6, 530	IKPVPEVKIL	0. 57
B*57:01	6, 441	6, 451	YKQFDTYNLW	0. 57
B*58:01	7, 036	7, 046	NTNPIQLSSY	0. 57
C*01:02	77	87	RTAPHGHVMV	0. 57
C*02:02	1, 812	1, 822	MSAPPAQYEL	0. 57
C*02:02	4, 930	4, 940	RNVIPTITQM	0. 57
C*02:02	5, 143	5, 153	YAYLRKHFSM	0. 57
C*02:02	6, 777	6, 787	VTIDYTEISF	0. 57
C*03:02	1, 812	1, 822	MSAPPAQYEL	0. 57
C*04:01	4, 107	4, 117	VDADSKIVQL	0. 57
C*04:01	4, 916	4, 926	MSYEDQDALF	0. 57
C*04:43	4, 107	4, 117	VDADSKIVQL	0. 57
C*04:43	4, 916	4, 926	MSYEDQDALF	0. 57
C*07:02	642	652	LRDGWEIVKF	0. 57
C*07:02	3, 825	3, 835	FRYMNSQGLL	0. 57
C*08:01	4, 008	4, 018	KMADQAMTQM	0. 57
C*14:02	1, 186	1, 196	NLYDKLVSSF	0. 57

C*14:02	1,951	1,961	GYKKPASREL	0.57
C*14:02	3,385	3,395	CYNGSPSGVY	0.57
C*14:02	5,143	5,153	YAYLRKHFSM	0.57
A*01:01	2,241	2,251	LSVCLGSLIY	0.58
A*02:01	1,095	1,105	YIATNGPLKV	0.58
A*02:06	1,291	1,301	IVGDVVQEGV	0.58
A*11:01	329	339	TSWQTGDFVK	0.58
A*11:01	1,508	1,518	ETISLAGSYK	0.58
A*11:01	3,223	3,233	SGAMDTTSYR	0.58
A*23:01	2,330	2,340	AEWFLAYILF	0.58
A*24:02	798	808	KYCALAPNMM	0.58
A*24:02	3,756	3,766	VMFLARGIVF	0.58
A*24:02	4,433	4,443	DIYNDKVAGF	0.58
A*25:01	3,200	3,210	SDVLLPLTQY	0.58
A*26:01	3,650	3,660	ATVAYFNMVY	0.58
A*26:01	6,772	6,782	SKVVKVTIDY	0.58
A*29:02	1,421	1,431	VDYGARFYFY	0.58
A*29:02	2,266	2,276	SYCTGYREGY	0.58
A*29:02	2,950	2,960	YESLRPDTRY	0.58
A*29:02	3,203	3,213	LLPLTQYNRY	0.58
A*29:02	6,438	6,448	LWVYKQFDTY	0.58
A*31:01	4,175	4,185	AYYNTTKGGR	0.58
A*68:01	2,155	2,165	VSTTTNIVTR	0.58
A*68:02	2,600	2,610	VNTFSSTFNV	0.58
A*68:02	2,869	2,879	FVVPGLPGTI	0.58
A*68:02	4,622	4,632	TTPGSGVPVV	0.58
B*07:02	2,572	2,582	QPILLLDQAL	0.58
B*07:02	3,660	3,670	MPASWVMRIM	0.58
B*14:02	76	86	ARTAPHGHVM	0.58
B*15:01	134	144	HSYGADLKSF	0.58
B*15:01	1,350	1,360	VLKKCKSAFY	0.58
B*15:01	4,276	4,286	AFAVDAAKAY	0.58
B*15:03	478	488	ASFSASTSAF	0.58
B*15:03	3,960	3,970	AAFATAQEAY	0.58
B*15:03	5,990	6,000	FKMNYQVNGY	0.58
B*15:03	6,067	6,077	SAKPPPGDQF	0.58
B*15:03	6,615	6,625	KQASLNGVTL	0.58
B*27:05	577	587	SLRLIDAMMF	0.58
B*27:05	3,006	3,016	SGRWVLNNDY	0.58
B*35:01	4,087	4,097	NTCDGTTFTY	0.58
B*35:01	6,168	6,178	NPFMIDVQQW	0.58
B*35:03	6,726	6,736	IPMDSTVKNY	0.58
B*35:03	6,799	6,809	YPKLQSSQAW	0.58

B*38:01	642	652	LRDGWEIVKF	0.58
B*40:01	4,478	4,488	QHEETIYNLL	0.58
B*40:02	3,937	3,947	EEMLDNRATL	0.58
B*40:02	4,211	4,221	YTELEPPCRF	0.58
B*44:02	242	252	TERSEKSYEL	0.58
B*44:02	327	337	GETSWQTGDF	0.58
B*44:02	1,131	1,141	EDIQLLKSAY	0.58
B*44:02	4,263	4,273	TEVPANSTVL	0.58
B*44:03	1,817	1,827	AQYELKHGTF	0.58
B*44:03	4,212	4,222	TELEPPCRFV	0.58
B*45:01	1,203	1,213	QVEQKIAEIP	0.58
B*45:01	4,392	4,402	REPMLQSADA	0.58
B*46:01	2,258	2,268	LMSNLGMPSY	0.58
B*51:01	730	740	LMPLKAPKEI	0.58
B*51:01	4,265	4,275	VPANSTVLSF	0.58
B*58:01	676	686	QTFFKLVNKF	0.58
B*58:01	2,205	2,215	KNTVKSVGKF	0.58
B*58:01	2,362	2,372	HFISNSWLMW	0.58
B*58:01	3,601	3,611	QSTQWSLFFF	0.58
B*58:01	3,944	3,954	ATLQAIASEF	0.58
B*58:01	6,067	6,077	SAKPPPGDQF	0.58
C*03:02	5,077	5,087	TSSGDATTAY	0.58
C*04:01	1,964	1,974	FFPDLNGDVV	0.58
C*04:43	1,964	1,974	FFPDLNGDVV	0.58
C*05:01	4,530	4,540	HFDEGNCDTL	0.58
C*07:02	2,598	2,608	AYVNTFSSTF	0.58
C*08:02	1,239	1,249	ACVEEVTTTL	0.58
C*08:02	5,905	5,915	MSDRDLYDKL	0.58
C*08:02	6,906	6,916	FVSDADSTLI	0.58
C*12:02	5,619	5,629	HFAIGLALYY	0.58
C*12:03	5,318	5,328	YEAMYTPHTV	0.58
C*14:02	1,863	1,873	EYKGPITDVF	0.58
C*15:02	164	174	KHSSGVTREL	0.58
C*16:01	4,276	4,286	AFAVDAAKAY	0.58
C*17:01	3,334	3,344	FLVQAGNVQL	0.58
C*17:03	3,334	3,344	FLVQAGNVQL	0.58
A*02:02	6,664	6,674	SQMEIDFLEL	0.59
A*03:01	2,551	2,561	KSKCEESSAK	0.59
A*11:01	518	528	KGAWNIGEQK	0.59
A*11:01	4,188	4,198	ALLSDLQDLK	0.59
A*26:01	1,766	1,776	TLKGVEAVMY	0.59
A*29:02	3,385	3,395	CYNGSPSGVY	0.59
A*29:02	5,077	5,087	TSSGDATTAY	0.59

A*31:01	4, 438	4, 448	KVAGFAKFLK	0. 59
A*31:01	5, 246	5, 256	KTDGTLMIER	0. 59
A*31:01	5, 481	5, 491	ATVREVLSDR	0. 59
A*32:01	1, 810	1, 820	VMMSAPPAQY	0. 59
A*33:01	2, 221	2, 231	NYLKSPNFSK	0. 59
A*68:02	832	842	EVQGYKSVNI	0. 59
A*68:02	877	887	DAVIKTLQPV	0. 59
A*68:02	3, 100	3, 110	TPVYSFLPGV	0. 59
A*68:02	5, 485	5, 495	EVLSDRELHL	0. 59
B*08:01	619	629	TVYEKLKPVL	0. 59
B*08:01	3, 910	3, 920	TEAFEKMVSL	0. 59
B*13:02	550	560	RTLETAQNSV	0. 59
B*13:02	4, 098	4, 108	SALWEIQQVV	0. 59
B*13:02	6, 630	6, 640	KTQFNYYKKV	0. 59
B*14:02	242	252	TERSEKSYEL	0. 59
B*15:01	5, 757	5, 767	LMKTIGPDMF	0. 59
B*15:03	1, 308	1, 318	TKKAGGTTEM	0. 59
B*15:03	1, 803	1, 813	VQQESPFVMM	0. 59
B*15:03	1, 864	1, 874	YKGPITDVFY	0. 59
B*15:03	3, 377	3, 387	GQTFSVLACY	0. 59
B*27:05	3, 825	3, 835	FRYMNSQGLL	0. 59
B*35:01	2, 509	2, 519	YFDKAGQKTY	0. 59
B*35:01	3, 360	3, 370	DTANPKTPKY	0. 59
B*38:01	6, 582	6, 592	GRVDGQVDLF	0. 59
B*40:01	1, 617	1, 627	SHEGKTFYVL	0. 59
B*44:02	1, 817	1, 827	AQYELKHGTF	0. 59
B*44:02	3, 313	3, 323	TSEDMLNPNY	0. 59
B*44:03	1, 465	1, 475	EEAARYMRSL	0. 59
B*44:03	3, 313	3, 323	TSEDMLNPNY	0. 59
B*45:01	6, 027	6, 037	TREAVGTNLP	0. 59
B*46:01	527	537	KSILSPLYAF	0. 59
B*46:01	3, 715	3, 725	TLMNVLTLVY	0. 59
B*49:01	1, 209	1, 219	AEIPKEEVKP	0. 59
B*53:01	5, 304	5, 314	LTNDNTSRYW	0. 59
B*57:01	3, 468	3, 478	ITVNVLAWLY	0. 59
B*58:01	2, 004	2, 014	NKATYKPNTW	0. 59
B*58:01	6, 491	6, 501	KVDGVDVELF	0. 59
C*02:02	4, 211	4, 221	YTELEPPCRF	0. 59
C*04:01	4, 338	4, 348	HIDHPNPKGF	0. 59
C*04:43	4, 338	4, 348	HIDHPNPKGF	0. 59
C*08:02	375	385	NSEVGPEHSL	0. 59
C*12:02	3, 953	3, 963	FSSLPSYAAF	0. 59
C*12:02	6, 805	6, 815	SQAWQPGVAM	0. 59

C*12:03	3,960	3,970	AAFATAQEAY	0.59
C*14:02	6,440	6,450	VYKQFDTYNL	0.59
C*15:02	4,107	4,117	VDADSKIVQL	0.59
A*02:02	1,253	1,263	FLTENLLLYI	0.6
A*02:02	4,592	4,602	AMRNAGIVGV	0.6
A*02:02	5,853	5,863	KILGLPTQTV	0.6
A*02:02	7,068	7,078	GQINDMILSL	0.6
A*02:05	5,732	5,742	AQLPAPRTLL	0.6
A*02:06	2,377	2,387	VQMAPISAMV	0.6
A*03:01	1,108	1,118	CVLSGHNLAK	0.6
A*03:01	1,276	1,286	VSDIDITFLK	0.6
A*03:01	2,144	2,154	ANYAKPFLNK	0.6
A*03:01	5,649	5,659	KALKYLPIDK	0.6
A*11:01	5,481	5,491	ATVREVLSDR	0.6
A*11:01	6,518	6,528	RNIKPVPEVK	0.6
A*24:02	2,354	2,364	LFFSYFAVHF	0.6
A*24:02	5,143	5,153	YAYLRKHFSM	0.6
A*25:01	707	717	ETFVTHSKGL	0.6
A*25:01	2,673	2,683	DSCNNYMLTY	0.6
A*25:01	5,272	5,282	QEYADVFHLY	0.6
A*25:01	6,571	6,581	TICAPLTVFF	0.6
A*25:01	6,648	6,658	ETYFTQSRNL	0.6
A*29:02	1,325	1,335	VPTDNYITTY	0.6
A*31:01	1,526	1,536	TQLGIEFLKR	0.6
A*32:01	5,853	5,863	KILGLPTQTV	0.6
A*68:01	5,568	5,578	TLVPQEHYVR	0.6
A*68:02	77	87	RTAPHGHVMV	0.6
A*68:02	1,876	1,886	NSYTTTIKPV	0.6
A*68:02	2,202	2,212	TIAKNTVKSV	0.6
A*68:02	7,006	7,016	EAFLIGCNYL	0.6
B*08:01	4,784	4,794	LLLDKRTTCF	0.6
B*14:02	1,841	1,851	YKHITSKETL	0.6
B*15:01	1,461	1,471	GLNLEEAARY	0.6
B*15:01	3,341	3,351	VQLRVIGHSM	0.6
B*15:01	4,537	4,547	DTLKEILVTY	0.6
B*18:01	790	800	LLEIKDTEKY	0.6
B*35:03	1,325	1,335	VPTDNYITTY	0.6
B*37:01	1,975	1,985	IDYKHYTPSF	0.6
B*37:01	4,263	4,273	TEVPANSTVL	0.6
B*37:01	5,207	5,217	HEFCSQHTML	0.6
B*39:01	4,825	4,835	FFKEGSSVEL	0.6
B*39:01	6,581	6,591	DGRVDGQVDL	0.6
B*40:01	1,145	1,155	QHEVLLAPLL	0.6

B*40:01	1,709	1,719	AGEAANFCAL	0.6
B*40:01	6,523	6,533	VPEVKILNNL	0.6
B*40:01	6,657	6,667	LQEFKPRSQM	0.6
B*40:02	248	258	SYELQTPFEI	0.6
B*40:02	656	666	ACEIVGGQIV	0.6
B*40:02	2,057	2,067	VENPTIQKDV	0.6
B*44:02	6,497	6,507	VELFENKTTL	0.6
B*45:01	4,212	4,222	TELEPPCRFV	0.6
B*46:01	5,003	5,013	YSDVENPHLM	0.6
B*46:01	5,077	5,087	TSSGDATTAY	0.6
B*51:01	2,969	2,979	FPNTYLEGSV	0.6
B*53:01	6,067	6,077	SAKPPPGDQF	0.6
B*57:01	2,382	2,392	ISAMVRMYIF	0.6
C*04:01	1,557	1,567	TFDNLKTLLS	0.6
C*04:43	1,557	1,567	TFDNLKTLLS	0.6
C*05:01	3,704	3,714	TVYDDGARRV	0.6
C*07:02	1,594	1,604	TYGQQFGPTY	0.6
C*08:01	6,490	6,500	TKVDGVDVEL	0.6
C*08:02	2,103	2,113	AYVDNSSLTI	0.6
C*14:02	5,545	5,555	TYKLNVGDYF	0.6
C*14:02	7,033	7,043	FWRNTNPIQL	0.6
A*01:01	3,960	3,970	AAFATAQEAY	0.61
A*01:01	5,116	5,126	YVRNLQHRLY	0.61
A*02:01	1,185	1,195	KNLYDKLVSS	0.61
A*02:01	2,960	2,970	VLMDGSIIQF	0.61
A*02:05	3,373	3,383	RIQPGQTFSV	0.61
A*02:05	5,287	5,297	KLHDELTGHM	0.61
A*02:05	5,853	5,863	KILGLPTQTV	0.61
A*02:06	4,246	4,256	MVLGSLAATV	0.61
A*03:01	5,466	5,476	TLKATEETFK	0.61
A*11:01	5,522	5,532	VQIGEYTFEK	0.61
A*11:01	5,591	5,601	FSSNVANYQK	0.61
A*25:01	2,324	2,334	TAFGLVAEWF	0.61
A*25:01	4,426	4,436	DVVYRAFDIY	0.61
A*25:01	5,861	5,871	TVDSSQGSEY	0.61
A*26:01	669	679	KEIKESVQTF	0.61
A*26:01	2,431	2,441	IVNGVRRSFY	0.61
A*26:01	4,930	4,940	RNVIPTITQM	0.61
A*26:01	5,275	5,285	ADVFHLYLQY	0.61
A*29:02	1,507	1,517	IETISLAGSY	0.61
A*29:02	2,979	2,989	RVVTTFDSEY	0.61
A*29:02	3,718	3,728	NVLTLVYKVY	0.61
A*29:02	6,181	6,191	GNLQSNHDLY	0.61

A*31:01	4,845	4,855	AAISDYDYYR	0.61
A*32:01	402	412	RTIAFGGCVF	0.61
A*33:01	3,476	3,486	LYAAVINGDR	0.61
A*68:01	281	291	IKTIQPRVEK	0.61
A*68:01	1,343	1,353	TVEEAKTVLK	0.61
A*68:01	6,482	6,492	SIINNTVYTK	0.61
A*68:02	772	782	EAVEAPLVGT	0.61
A*68:02	1,556	1,566	ITFDNLKTLL	0.61
B*07:02	6,799	6,809	YPKLQSSQAW	0.61
B*08:01	2,622	2,632	EAELAKNVSL	0.61
B*13:02	6,963	6,973	KLALGGSVAI	0.61
B*14:02	6,680	6,690	ERYKLEGYAF	0.61
B*15:01	232	242	REHEHEIAWY	0.61
B*15:01	2,598	2,608	AYVNTFSSTF	0.61
B*15:03	4,762	4,772	RLSFKELLVY	0.61
B*18:01	9	19	NEKTHVQLSL	0.61
B*18:01	5,207	5,217	HEFCSQHTML	0.61
B*27:05	3,073	3,083	RRAFGEYSHV	0.61
B*27:05	4,761	4,771	SRLSFKELLV	0.61
B*27:05	6,888	6,898	LRQWLPTGTL	0.61
B*35:01	1,179	1,189	YLAVFDKNLY	0.61
B*35:01	3,222	3,232	FSGAMDTTSY	0.61
B*39:01	1,144	1,154	NQHEVLLAPL	0.61
B*40:02	3,077	3,087	GEYSHVVAFN	0.61
B*44:03	5,207	5,217	HEFCSQHTML	0.61
B*46:01	591	601	ATNNLVVMAY	0.61
B*46:01	5,741	5,751	LTKGTLEPEY	0.61
B*46:01	6,154	6,164	YACWHHSIGF	0.61
B*49:01	245	255	SEKSYELQTP	0.61
B*49:01	866	876	TEVNEFACVV	0.61
B*49:01	960	970	LEFGATSAAL	0.61
B*51:01	1,394	1,404	MPVCVETKAI	0.61
B*51:01	2,225	2,235	SPNFSKLINI	0.61
B*57:01	404	414	IAFGGCVFSY	0.61
B*57:01	3,290	3,300	CGTTTLNGLW	0.61
B*58:01	2,964	2,974	GSIIQFPNTY	0.61
C*01:02	5,653	5,663	YLPIDKCSRI	0.61
C*02:02	5,675	5,685	FKVNSTLEQY	0.61
C*02:02	6,289	6,299	KAYKIEELFY	0.61
C*03:02	2,509	2,519	YFDKAGQKTY	0.61
C*03:04	874	884	VVADAVIKTL	0.61
C*05:01	3,670	3,680	TWLDMVDTSL	0.61
C*07:01	1,540	1,550	VYYTSNPTTF	0.61

C*07:02	1, 158	1, 168	IFGADPIHSL	0. 61
C*07:02	2, 172	2, 182	NYMPYFFTLL	0. 61
C*07:04	387	397	YHNESGLKTI	0. 61
C*08:01	1, 160	1, 170	GADPIHSLRV	0. 61
C*14:02	1, 646	1, 656	SFLGRYMSAL	0. 61
C*14:02	2, 266	2, 276	SYCTGYREGY	0. 61
C*15:02	722	732	KSREETGLLM	0. 61
C*15:02	4, 721	4, 731	TSFGPLVRKI	0. 61
C*15:02	4, 930	4, 940	RNVIPTITQM	0. 61
A*01:01	897	907	LDEWSMATYY	0. 62
A*01:01	6, 777	6, 787	VTIDYTEISF	0. 62
A*02:01	3, 195	3, 205	YLKLRSDVLL	0. 62
A*02:01	4, 099	4, 109	ALWEIQQVVD	0. 62
A*02:01	4, 915	4, 925	SMSYEDQDAL	0. 62
A*02:02	467	477	FKLNEEIAII	0. 62
A*02:06	6, 250	6, 260	ALLADKFPVL	0. 62
A*03:01	3, 188	3, 198	FLLNKEMYLK	0. 62
A*03:01	3, 361	3, 371	TANPKTPKYK	0. 62
A*11:01	216	226	SEQLDFIDTK	0. 62
A*11:01	1, 277	1, 287	SDIDITFLKK	0. 62
A*11:01	2, 467	2, 477	STFISDEVAR	0. 62
A*24:02	3, 748	3, 758	NYSGVVTTVM	0. 62
A*25:01	1, 027	1, 037	TIEVNSFSGY	0. 62
A*26:01	3, 818	3, 828	YLVSTQEFRY	0. 62
A*29:02	3, 377	3, 387	GQTFSVLACY	0. 62
A*29:02	4, 168	4, 178	CTDDNALAYY	0. 62
A*29:02	5, 573	5, 583	EHYVRITGLY	0. 62
A*29:02	6, 685	6, 695	EGYAFEHIVY	0. 62
A*29:02	6, 926	6, 936	KWDLIISDMY	0. 62
A*32:01	3, 168	3, 178	RRVVFNGVSF	0. 62
A*32:01	4, 756	4, 766	VNLHSSRLSF	0. 62
A*68:01	813	823	FTLKGGAPTK	0. 62
A*68:01	1, 466	1, 476	EAARYMRSLK	0. 62
A*68:01	6, 860	6, 870	LTLAVPYNMR	0. 62
A*68:01	7, 036	7, 046	NTNPIQLSSY	0. 62
A*68:02	3, 120	3, 130	YLTNDVSFLA	0. 62
B*07:02	5, 410	5, 420	FPLCANGQVF	0. 62
B*08:01	4, 825	4, 835	FFKEGSSVEL	0. 62
B*13:02	6, 387	6, 397	KQVVSDIDYV	0. 62
B*15:01	915	925	KLASHMYCSF	0. 62
B*15:01	3, 200	3, 210	SDVLLPLTQY	0. 62
B*15:01	4, 983	4, 993	TVVIGTSKFY	0. 62
B*15:01	5, 861	5, 871	TVDSSQGSEY	0. 62

B*15:03	61	71	LPQLEQPYVF	0.62
B*15:03	2,376	2,386	LVQMAPISAM	0.62
B*15:03	6,415	6,425	RHHANEYRLY	0.62
B*18:01	5,390	5,400	TQLYLGGMSY	0.62
B*35:01	4,844	4,854	NAAISDYDYY	0.62
B*35:01	5,644	5,654	DALCEKALKY	0.62
B*35:03	2,870	2,880	VVPGLPGTIL	0.62
B*39:01	5,294	5,304	GHMLDMYSVM	0.62
B*40:01	2,053	2,063	SEEVVENPTI	0.62
B*40:01	4,453	4,463	FQEKDEDDNL	0.62
B*40:02	4,670	4,680	YDFTEERLKL	0.62
B*40:02	6,944	6,954	KENDSKEGFF	0.62
B*46:01	378	388	VGPEHSLAEY	0.62
B*53:01	4,916	4,926	MSYEDQDALF	0.62
B*58:01	3,705	3,715	VYDDGARRVW	0.62
C*03:02	3,076	3,086	FGEYSHVVAF	0.62
C*05:01	824	834	TFGDDTVIEV	0.62
C*05:01	2,816	2,826	AIDGGVTRDI	0.62
C*05:01	2,910	2,920	YTDFATSACV	0.62
C*12:03	1,878	1,888	YTTTIKPVTY	0.62
C*14:02	2,249	2,259	IYSTAALGVL	0.62
C*14:02	6,422	6,432	RLYLDAYNMM	0.62
C*17:01	1,519	1,529	WSYSGQSTQL	0.62
C*17:03	1,519	1,529	WSYSGQSTQL	0.62
A*01:01	1,772	1,782	AVMYMGTLSY	0.63
A*02:02	1,036	1,046	YLKLTDNVYI	0.63
A*02:02	5,050	5,060	YRLANECAQV	0.63
A*02:02	5,295	5,305	HMLDMYSVML	0.63
A*02:02	5,711	5,721	YDLSVVNARL	0.63
A*03:01	1,193	1,203	SSFLEMKSEK	0.63
A*03:01	4,010	4,020	ADQAMTQMYK	0.63
A*03:01	4,779	4,789	AASGNLLLDK	0.63
A*03:01	6,060	6,070	NTDFSRVSAK	0.63
A*11:01	2,025	2,035	ETSNSFDVLK	0.63
A*11:01	2,144	2,154	ANYAKPFLNK	0.63
A*11:01	2,919	2,929	VLAAECTIFK	0.63
A*24:02	2,145	2,155	NYAKPFLNKV	0.63
A*25:01	1,402	1,412	AIVSTIQRKY	0.63
A*25:01	5,319	5,329	EAMYTPHTVL	0.63
A*29:02	176	186	ELNGGAYTRY	0.63
A*68:02	3,614	3,624	NAFLPFAMGI	0.63
A*68:02	4,262	4,272	ATEVPANSTV	0.63
B*08:01	2,707	2,717	QVAKSHNIAL	0.63

B*13:02	4,430	4,440	RAFDIYNDKV	0.63
B*15:01	1,274	1,284	TLVSDIDITF	0.63
B*15:03	500	510	KQIVESCGNF	0.63
B*27:05	4,512	4,522	SRQRLTKYTM	0.63
B*35:01	152	162	DPYEDFQENW	0.63
B*35:01	1,482	1,492	VSSPDAVTAY	0.63
B*35:03	5,322	5,332	YTPHTVLQAV	0.63
B*38:01	1,504	1,514	EHFIETISLA	0.63
B*39:01	3,997	4,007	DRDAAMQRKL	0.63
B*40:01	6,675	6,685	MDEFIERYKL	0.63
B*40:02	538	548	SEAARVVRSI	0.63
B*44:02	248	258	SYELQTPFEI	0.63
B*44:03	946	956	QYEYGTEDDY	0.63
B*44:03	2,622	2,632	EAELAKNVSL	0.63
B*46:01	173	183	LMRELNGGAY	0.63
B*46:01	4,009	4,019	MADQAMTQMY	0.63
B*46:01	4,916	4,926	MSYEDQDALF	0.63
B*49:01	6,716	6,726	KESPFELEDF	0.63
B*51:01	2,173	2,183	YMPYFFTLLL	0.63
B*51:01	6,322	6,332	YPANSIVCRF	0.63
B*53:01	1,274	1,284	TLVSDIDITF	0.63
B*53:01	7,025	7,035	YVMHANYIFW	0.63
B*57:01	608	618	LTSQWLTNIF	0.63
B*57:01	1,812	1,822	MSAPPAQYEL	0.63
B*57:01	4,656	4,666	TDLTKPYIKW	0.63
B*58:01	816	826	KGGAPTKVTF	0.63
C*02:02	6,154	6,164	YACWHHSIGF	0.63
C*03:02	6,777	6,787	VTIDYTEISF	0.63
C*06:02	4,593	4,603	MRNAGIVGVL	0.63
C*06:02	6,091	6,101	VVRIKIVQML	0.63
C*07:01	3,489	3,499	NRFTTTLNDF	0.63
C*07:01	5,576	5,586	VRITGLYPTL	0.63
C*07:02	1,639	1,649	YYHTTDPSFL	0.63
C*12:03	134	144	HSYGADLKSF	0.63
C*12:03	3,074	3,084	RAFGEYSHVV	0.63
C*15:02	1,160	1,170	GADPIHSLRV	0.63
C*17:01	2,222	2,232	YLKSPNFSKL	0.63
C*17:01	2,469	2,479	FISDEVARDL	0.63
C*17:03	2,222	2,232	YLKSPNFSKL	0.63
C*17:03	2,469	2,479	FISDEVARDL	0.63
A*01:01	1,252	1,262	KFLTENLLLY	0.64
A*01:01	2,485	2,495	PINPTDQSSY	0.64
A*01:01	2,827	2,837	STDTCFANKH	0.64

A*01:01	5,391	5,401	QLYLGGMSYY	0.64
A*02:02	1,684	1,694	ALLTLQQIEL	0.64
A*02:02	2,080	2,090	IILKPANNSL	0.64
A*02:02	2,377	2,387	VQMAPISAMV	0.64
A*02:02	3,643	3,653	LFLLPSLATV	0.64
A*02:02	3,875	3,885	VVLLSVLQQL	0.64
A*02:05	5,250	5,260	TLMIERFVSL	0.64
A*03:01	3,862	3,872	TVQSKMSDVK	0.64
A*03:01	5,828	5,838	FLTRNPAWRK	0.64
A*11:01	1,108	1,118	CVLSGHNLAK	0.64
A*23:01	1,594	1,604	TYGQQFGPTY	0.64
A*23:01	4,678	4,688	KLFDRYFKYW	0.64
A*25:01	3,134	3,144	MVMFTPLVPF	0.64
A*25:01	6,999	7,009	NVNASSSEAF	0.64
A*26:01	264	274	DIFNGECPNF	0.64
A*26:01	5,390	5,400	TQLYLGGMSY	0.64
A*26:01	5,469	5,479	ATEETFKLSY	0.64
A*31:01	2,196	2,206	KASMPTTIAK	0.64
A*31:01	3,876	3,886	VLLSVLQQLR	0.64
A*31:01	6,583	6,593	RVDGQVDLFR	0.64
A*31:01	6,860	6,870	LTLAVPYNMR	0.64
A*33:01	1,221	1,231	TESKPSVEQR	0.64
A*33:01	2,467	2,477	STFISDEVAR	0.64
B*07:02	6,380	6,390	SPCESHGKQV	0.64
B*07:02	6,613	6,623	GPKQASLNGV	0.64
B*08:01	6,864	6,874	VPYNMRVIHF	0.64
B*15:01	4,784	4,794	LLLDKRTTCF	0.64
B*15:01	5,538	5,548	VVYRGTTTYK	0.64
B*35:01	891	901	TPLGIDLDEW	0.64
B*39:01	7,089	7,099	NRVVISSDVL	0.64
B*44:02	2,622	2,632	EAELAKNVSL	0.64
B*44:02	4,212	4,222	TELEPPCRFV	0.64
B*44:02	6,666	6,676	MEIDFLELAM	0.64
B*44:03	897	907	LDEWSMATYY	0.64
B*45:01	669	679	KEIKESVQTF	0.64
B*45:01	1,052	1,062	EEAKKVKPTV	0.64
B*46:01	5,021	5,031	RAMPNMLRIM	0.64
B*53:01	1,501	1,511	TPEEHFIETI	0.64
B*53:01	3,105	3,115	FLPGVYSVIY	0.64
B*53:01	5,188	5,198	NVFMSEAKCW	0.64
B*57:01	669	679	KEIKESVQTF	0.64
B*57:01	1,244	1,254	VTTTLEETKF	0.64
B*57:01	4,714	4,724	FSTVFPLTSF	0.64

C*01:02	5,003	5,013	YSDVENPHLM	0.64
C*05:01	2,670	2,680	VTGDSCNNYM	0.64
C*07:02	2,792	2,802	FYLIIPVHVM	0.64
C*08:01	5,273	5,283	EYADVFHLYL	0.64
C*08:02	6,777	6,787	VTIDYTEISF	0.64
C*12:02	2,440	2,450	YVYANGGKGF	0.64
C*12:03	2,557	2,567	SSAKSASVYY	0.64
C*14:02	834	844	QGYKSVNITF	0.64
C*16:01	3,960	3,970	AAFATAQEAY	0.64
C*17:01	5,225	5,235	YLPYPDPSRI	0.64
C*17:03	5,225	5,235	YLPYPDPSRI	0.64
A*01:01	378	388	VGPEHSLAEY	0.65
A*01:01	2,901	2,911	CYTPSKLIEY	0.65
A*01:01	5,487	5,497	LSDRELHLSW	0.65
A*01:01	6,376	6,386	YYSDSPCESH	0.65
A*02:01	3,052	3,062	IVAGGIVAIV	0.65
A*02:01	3,373	3,383	RIQPGQTFSV	0.65
A*02:01	5,783	5,793	ALVYDNKLKA	0.65
A*02:01	7,068	7,078	GQINDMILSL	0.65
A*02:02	1,267	1,277	NLHPDSATLV	0.65
A*02:02	3,195	3,205	YLKLRSDVLL	0.65
A*02:02	6,082	6,092	LMYKGLPWNV	0.65
A*02:05	2,377	2,387	VQMAPISAMV	0.65
A*03:01	4,962	4,972	TMTNRQFHQK	0.65
A*23:01	2,274	2,284	GYLNSTNVTI	0.65
A*24:02	6,680	6,690	ERYKLEGYAF	0.65
A*25:01	404	414	IAFGGCVFSY	0.65
A*25:01	525	535	EQKSILSPLY	0.65
A*29:02	1,896	1,906	EIDPKLDNYY	0.65
A*29:02	4,653	4,663	HVDTDLTKPY	0.65
A*33:01	3,065	3,075	LAYYFMRFRR	0.65
A*68:02	2,047	2,057	EDLKPVSEEV	0.65
A*68:02	2,310	2,320	ETIQITISSF	0.65
A*68:02	6,933	6,943	DMYDPKTKNV	0.65
B*07:02	4,717	4,727	VFPLTSFGPL	0.65
B*08:01	679	689	FKLVNKFLAL	0.65
B*14:02	2,952	2,962	SLRPDTRYVL	0.65
B*15:01	1,024	1,034	VVQTIEVNSF	0.65
B*15:01	1,369	1,379	KQEILGTVSW	0.65
B*15:01	1,671	1,681	SIKWADNNCY	0.65
B*15:01	3,286	3,296	VQVTCGTTTL	0.65
B*15:03	1,895	1,905	TEIDPKLDNY	0.65
B*18:01	1,131	1,141	EDIQLLKSAY	0.65

B*35:03	731	741	MPLKAPKEII	0.65
B*38:01	95	105	IQYGRSGETL	0.65
B*38:01	2,129	2,139	THGLAAVNSV	0.65
B*39:01	1,145	1,155	QHEVLLAPLL	0.65
B*39:01	3,512	3,522	QDHVDILGPL	0.65
B*40:02	4,005	4,015	KLEKMADQAM	0.65
B*44:02	1,052	1,062	EEAKKVKPTV	0.65
B*44:03	248	258	SYELQTPFEI	0.65
B*45:01	3,950	3,960	ASEFSSLPSY	0.65
B*46:01	1,631	1,641	TLRVEAFEYY	0.65
B*46:01	2,430	2,440	TIVNGVRRSF	0.65
B*46:01	2,431	2,441	IVNGVRRSFY	0.65
B*46:01	5,777	5,787	IVDTVSALVY	0.65
B*49:01	6,665	6,675	QMEIDFLELA	0.65
B*51:01	1,021	1,031	LTPVVQTIEV	0.65
B*57:01	6,067	6,077	SAKPPPGDQF	0.65
B*58:01	1,542	1,552	YTSNPTTFHL	0.65
B*58:01	4,625	4,635	GSGVPVVDSY	0.65
C*02:02	3,222	3,232	FSGAMDTTSY	0.65
C*03:02	5,725	5,735	YVYIGDPAQL	0.65
C*05:01	1,275	1,285	LVSDIDITFL	0.65
C*07:01	242	252	TERSEKSYEL	0.65
C*07:01	3,070	3,080	MRFRRAFGEY	0.65
C*08:01	1,549	1,559	FHLDGEVITF	0.65
C*12:02	5,537	5,547	AVVYRGTTTY	0.65
C*15:02	76	86	ARTAPHGHVM	0.65
A*02:01	2,568	2,578	QLMCQPILLL	0.66
A*02:02	359	369	YLPQNAVVKI	0.66
A*02:02	814	824	TLKGGAPTKV	0.66
A*02:02	3,052	3,062	IVAGGIVAIV	0.66
A*02:02	4,256	4,266	RLQAGNATEV	0.66
A*02:05	4,396	4,406	LQSADAQSFL	0.66
A*23:01	3,796	3,806	TCYFGLFCLL	0.66
A*23:01	7,013	7,023	NYLGKPCEQI	0.66
A*24:02	5,084	5,094	TAYANSVFNI	0.66
A*25:01	5,776	5,786	EIVDTVSALV	0.66
A*29:02	1,615	1,625	HNSHEGKTFY	0.66
A*29:02	3,156	3,166	KHFYWFFSNY	0.66
A*31:01	4,520	4,530	TMADLVYALR	0.66
A*32:01	2,168	2,178	RVCTNYMPYF	0.66
A*68:01	1,276	1,286	VSDIDITFLK	0.66
A*68:02	6,041	6,051	FSTGVNLVAV	0.66
A*68:02	6,165	6,175	YVYNPFMIDV	0.66

B*07:02	5,842	5,852	SPYNSQNAVA	0.66
B*13:02	4,580	4,590	RQALLKTVQF	0.66
B*15:03	938	948	EEEFEPSTQY	0.66
B*35:01	2,473	2,483	EVARDLSLQF	0.66
B*35:01	6,864	6,874	VPYNMRVIHF	0.66
B*37:01	1,817	1,827	AQYELKHGTF	0.66
B*38:01	6,680	6,690	ERYKLEGYAF	0.66
B*40:01	744	754	GETLPTEVLT	0.66
B*44:02	5,207	5,217	HEFCSQHTML	0.66
B*44:03	2,517	2,527	TYERHSLSHF	0.66
B*44:03	4,262	4,272	ATEVPANSTV	0.66
B*44:03	4,656	4,666	TDLTKPYIKW	0.66
B*45:01	538	548	SEAARVVRSI	0.66
B*45:01	1,018	1,028	EMELTPVVQT	0.66
B*45:01	1,690	1,700	QIELKFNPPA	0.66
B*49:01	2,808	2,818	SSEIIGYKAI	0.66
B*49:01	3,950	3,960	ASEFSSLPSY	0.66
B*51:01	3,911	3,921	EAFEKMVSLL	0.66
B*53:01	359	369	YLPQNAVVKI	0.66
B*53:01	6,007	6,017	EEAIRHVRAW	0.66
B*57:01	5,188	5,198	NVFMSEAKCW	0.66
C*02:02	478	488	ASFSASTSAF	0.66
C*02:02	6,322	6,332	YPANSIVCRF	0.66
C*03:02	1,179	1,189	YLAVFDKNLY	0.66
C*03:02	4,795	4,805	VAALTNNVAF	0.66
C*03:02	7,036	7,046	NTNPIQLSSY	0.66
C*05:01	4,044	4,054	KLDNDALNNI	0.66
C*05:01	5,273	5,283	EYADVFHLYL	0.66
C*07:01	3,809	3,819	FRLTLGVYDY	0.66
C*07:01	4,593	4,603	MRNAGIVGVL	0.66
C*07:02	5,116	5,126	YVRNLQHRLY	0.66
C*07:04	5,910	5,920	LYDKLQFTSL	0.66
C*08:02	5,273	5,283	EYADVFHLYL	0.66
C*12:02	5,725	5,735	YVYIGDPAQL	0.66
C*12:03	4,276	4,286	AFAVDAAKAY	0.66
C*12:03	5,021	5,031	RAMPNMLRIM	0.66
C*14:02	4,722	4,732	SFGPLVRKIF	0.66
C*15:02	1,519	1,529	WSYSGQSTQL	0.66
C*15:02	6,699	6,709	HSQLGGLHLL	0.66
C*17:01	4,777	4,787	MHAASGNLLL	0.66
C*17:03	4,777	4,787	MHAASGNLLL	0.66
A*01:01	2,137	2,147	SVPWDTIANY	0.67
A*01:01	6,181	6,191	GNLQSNHDLY	0.67

A*02:01	359	369	YLPQNAVVKI	0.67
A*02:01	4,143	4,153	KLQNNELSPV	0.67
A*02:02	1,185	1,195	KNLYDKLVSS	0.67
A*02:02	2,230	2,240	KLINIIIWFL	0.67
A*02:05	881	891	KTLQPVSELL	0.67
A*02:05	4,771	4,781	YAADPAMHAA	0.67
A*02:06	6,387	6,397	KQVVSDIDYV	0.67
A*03:01	1,810	1,820	VMMSAPPAQY	0.67
A*03:01	6,880	6,890	GVAPGTAVLR	0.67
A*11:01	110	120	HVGEIPVAYR	0.67
A*11:01	3,626	3,636	MSAFAMMFVK	0.67
A*11:01	3,862	3,872	TVQSKMSDVK	0.67
A*11:01	6,606	6,616	KGLQPSVGPK	0.67
A*24:02	1,817	1,827	AQYELKHGTF	0.67
A*24:02	2,960	2,970	VLMDGSIIQF	0.67
A*25:01	37	47	EVLSEARQHL	0.67
A*25:01	1,508	1,518	ETISLAGSYK	0.67
A*25:01	1,630	1,640	DTLRVEAFEY	0.67
A*26:01	220	230	DFIDTKRGVY	0.67
A*26:01	1,615	1,625	HNSHEGKTFY	0.67
A*26:01	2,964	2,974	GSIIQFPNTY	0.67
A*26:01	5,863	5,873	DSSQGSEYDY	0.67
A*29:02	612	622	WLTNIFGTVY	0.67
A*29:02	3,816	3,826	YDYLVSTQEF	0.67
A*31:01	5,042	5,052	CCSLSHRFYR	0.67
A*32:01	2,362	2,372	HFISNSWLMW	0.67
A*32:01	3,134	3,144	MVMFTPLVPF	0.67
A*32:01	4,859	4,869	TMCDIRQLLF	0.67
A*32:01	6,306	6,316	KFTDGVCLFW	0.67
A*33:01	2,814	2,824	YKAIDGGVTR	0.67
A*33:01	5,277	5,287	VFHLYLQYIR	0.67
A*68:02	2,584	2,594	DVGDSAEVAV	0.67
A*68:02	4,437	4,447	DKVAGFAKFL	0.67
A*68:02	5,137	5,147	DFVNEFYAYL	0.67
A*68:02	6,912	6,922	STLIGDCATV	0.67
B*07:02	958	968	KPLEFGATSA	0.67
B*07:02	3,371	3,381	FVRIQPGQTF	0.67
B*07:02	5,269	5,279	HPNQEYADVF	0.67
B*07:02	5,841	5,851	ISPYNSQNAV	0.67
B*07:02	6,358	6,368	TPAFDKSAFV	0.67
B*15:03	5,391	5,401	QLYLGGMSYY	0.67
B*18:01	5,005	5,015	DVENPHLMGW	0.67
B*27:05	397	407	LRKGGRTIAF	0.67

B*35:01	3, 203	3, 213	LLPLTQYNRY	0. 67
B*35:01	3, 904	3, 914	LLAKDTTEAF	0. 67
B*35:01	4, 537	4, 547	DTLKEILVTY	0. 67
B*40:01	1, 016	1, 026	QLEMELTPVV	0. 67
B*44:03	9	19	NEKTHVQLSL	0. 67
B*44:03	242	252	TERSEKSYEL	0. 67
B*45:01	158	168	QENWNTKHSS	0. 67
B*46:01	3, 140	3, 150	LVPFWITIAY	0. 67
B*46:01	4, 762	4, 772	RLSFKELLVY	0. 67
B*46:01	5, 175	5, 185	SIKNFKSVLY	0. 67
B*46:01	6, 503	6, 513	KTTLPVNVAF	0. 67
B*53:01	3, 276	3, 286	FPSGKVEGCM	0. 67
B*57:01	2, 004	2, 014	NKATYKPNTW	0. 67
B*58:01	2, 557	2, 567	SSAKSASVYY	0. 67
B*58:01	2, 710	2, 720	KSHNIALIWN	0. 67
C*03:02	76	86	ARTAPHGHVM	0. 67
C*03:02	1, 586	1, 596	TQVVDMSMTY	0. 67
C*03:02	2, 941	2, 951	TNVLEGSVAY	0. 67
C*03:04	6, 836	6, 846	SATLPKGIMM	0. 67
C*06:02	5, 576	5, 586	VRITGLYPTL	0. 67
C*07:04	3, 371	3, 381	FVRIQPGQTF	0. 67
C*07:04	5, 002	5, 012	VYSDVENPHL	0. 67
C*14:02	295	305	GFMGRIRSVY	0. 67
C*14:02	2, 171	2, 181	TNYMPYFFTL	0. 67
C*14:02	6, 376	6, 386	YYSDSPCESH	0. 67
C*15:02	4, 430	4, 440	RAFDIYNDKV	0. 67
C*17:01	3, 973	3, 983	VANGDSEVVL	0. 67
C*17:01	4, 477	4, 487	YQHEETIYNL	0. 67
C*17:03	3, 973	3, 983	VANGDSEVVL	0. 67
C*17:03	4, 477	4, 487	YQHEETIYNL	0. 67
A*01:01	1, 586	1, 596	TQVVDMSMTY	0. 68
A*01:01	1, 827	1, 837	TCASEYTGNY	0. 68
A*01:01	5, 644	5, 654	DALCEKALKY	0. 68
A*01:01	5, 796	5, 806	KSAQCFKMFY	0. 68
A*02:01	1, 295	1, 305	VVQEGVLTAV	0. 68
A*02:02	1, 888	1, 898	KLDGVVCTEI	0. 68
A*02:02	5, 908	5, 918	RDLYDKLQFT	0. 68
A*02:02	6, 098	6, 108	QMLSDTLKNL	0. 68
A*03:01	1, 880	1, 890	TTIKPVTYKL	0. 68
A*24:02	4, 633	4, 643	SYYSLLMPIL	0. 68
A*25:01	83	93	HVMVELVAEL	0. 68
A*26:01	1, 576	1, 586	FTTVDNINLH	0. 68
A*26:01	5, 978	5, 988	DMTYRRLISM	0. 68

A*29:02	4,537	4,547	DTLKEILVTY	0.68
A*32:01	6,289	6,299	KAYKIEELFY	0.68
A*68:01	3,361	3,371	TANPKTPKYK	0.68
A*68:02	3,910	3,920	TEAFEKMVSL	0.68
A*68:02	4,324	4,334	ESFGGASCCL	0.68
B*13:02	5,819	5,829	RPQIGVVREF	0.68
B*15:01	591	601	ATNNLVVMAY	0.68
B*27:05	5,500	5,510	KPRPPLNRNY	0.68
B*35:01	1,333	1,343	TYPGQGLNGY	0.68
B*35:01	4,653	4,663	HVDTDLTKPY	0.68
B*35:03	1,812	1,822	MSAPPAQYEL	0.68
B*35:03	6,567	6,577	KPTETICAPL	0.68
B*40:02	6,523	6,533	VPEVKILNNL	0.68
B*46:01	6,289	6,299	KAYKIEELFY	0.68
B*49:01	3,993	4,003	KSEFDRDAAM	0.68
B*53:01	378	388	VGPEHSLAEY	0.68
B*53:01	6,441	6,451	YKQFDTYNLW	0.68
B*58:01	2,930	2,940	ASGKPVPYCY	0.68
B*58:01	4,580	4,590	RQALLKTVQF	0.68
C*02:02	5,021	5,031	RAMPNMLRIM	0.68
C*02:02	6,847	6,857	VAKYTQLCQY	0.68
C*03:02	1,772	1,782	AVMYMGTLSY	0.68
C*03:02	4,277	4,287	FAVDAAKAYK	0.68
C*03:04	5,560	5,570	TVMPLSAPTL	0.68
C*07:01	1,533	1,543	LKRGDKSVYY	0.68
C*07:02	3,705	3,715	VYDDGARRVW	0.68
C*07:04	2,952	2,962	SLRPDTRYVL	0.68
C*07:04	5,003	5,013	YSDVENPHLM	0.68
C*12:02	76	86	ARTAPHGHVM	0.68
C*12:02	1,812	1,822	MSAPPAQYEL	0.68
C*14:02	3,632	3,642	MFVKHKHAFL	0.68
C*16:01	2,928	2,938	KDASGKPVPY	0.68
C*16:01	6,416	6,426	HHANEYRLYL	0.68
A*01:01	220	230	DFIDTKRGVY	0.69
A*01:01	241	251	YTERSEKSYE	0.69
A*01:01	938	948	EEEFEPSTQY	0.69
A*01:01	3,212	3,222	YLALYNKYKY	0.69
A*01:01	4,605	4,615	DNQDLNGNWY	0.69
A*01:01	6,627	6,637	EAVKTQFNYY	0.69
A*02:06	1,801	1,811	YLVQQESPFV	0.69
A*02:06	5,063	5,073	MVMCGGSLYV	0.69
A*03:01	2,731	2,741	RKQIRSAAKK	0.69
A*03:01	5,904	5,914	IMSDRDLYDK	0.69

A*03:01	6,704	6,714	GLHLLIGLAK	0.69
A*11:01	2,723	2,733	FMSLSEQLRK	0.69
A*11:01	5,780	5,790	TVSALVYDNK	0.69
A*11:01	6,494	6,504	GVDVELFENK	0.69
A*24:02	3,727	3,737	YYGNALDQAI	0.69
A*26:01	553	563	ETAQNSVRVL	0.69
A*29:02	3,222	3,232	FSGAMDTTSY	0.69
A*31:01	4,814	4,824	KDFYDFAVSK	0.69
A*32:01	2,629	2,639	VSLDNVLSTF	0.69
A*33:01	2,332	2,342	WFLAYILFTR	0.69
A*33:01	3,801	3,811	LFCLLNRYFR	0.69
A*33:01	6,126	6,136	KYFVKIGPER	0.69
A*68:01	2,794	2,804	LIIPVHVMSK	0.69
B*13:02	5,865	5,875	SQGSEYDYVI	0.69
B*15:01	1,878	1,888	YTTTIKPVTY	0.69
B*15:01	3,134	3,144	MVMFTPLVPF	0.69
B*15:01	4,734	4,744	GVPFVVSTGY	0.69
B*15:03	5,272	5,282	QEYADVFHLY	0.69
B*18:01	4,263	4,273	TEVPANSTVL	0.69
B*35:01	196	206	YPLECIKDLL	0.69
B*35:01	1,086	1,096	NAMQVESDDY	0.69
B*35:01	4,916	4,926	MSYEDQDALF	0.69
B*35:01	6,118	6,128	HGFELTSMKY	0.69
B*35:01	6,158	6,168	HHSIGFDYVY	0.69
B*35:03	2,487	2,497	NPTDQSSYIV	0.69
B*40:01	5,272	5,282	QEYADVFHLY	0.69
B*40:02	1,130	1,140	GEDIQLLKSA	0.69
B*40:02	1,617	1,627	SHEGKTFYVL	0.69
B*44:02	4,262	4,272	ATEVPANSTV	0.69
B*44:03	1,249	1,259	EETKFLTENL	0.69
B*46:01	3,200	3,210	SDVLLPLTQY	0.69
B*46:01	5,143	5,153	YAYLRKHFSM	0.69
B*49:01	6,666	6,676	MEIDFLELAM	0.69
B*51:01	2,082	2,092	LKPANNSLKI	0.69
B*57:01	4,982	4,992	ATVVIGTSKF	0.69
B*57:01	7,036	7,046	NTNPIQLSSY	0.69
B*58:01	2,950	2,960	YESLRPDTRY	0.69
B*58:01	3,153	3,163	ISTKHFYWFF	0.69
C*05:01	1,239	1,249	ACVEEVTTTL	0.69
C*15:02	6,416	6,426	HHANEYRLYL	0.69
C*16:01	1,615	1,625	HNSHEGKTFY	0.69
A*01:01	5,154	5,164	ILSDDAVVCF	0.7
A*02:01	2,345	2,355	VLGLAAIMQL	0.7

A*02:01	5,908	5,918	RDLYDKLQFT	0.7
A*02:02	6,754	6,764	LLLDDFVEII	0.7
A*02:05	1,095	1,105	YIATNGPLKV	0.7
A*02:05	6,387	6,397	KQVVSDIDYV	0.7
A*02:06	445	455	GLNDNLLEIL	0.7
A*02:06	1,817	1,827	AQYELKHGTF	0.7
A*02:06	2,960	2,970	VLMDGSIIQF	0.7
A*02:06	5,560	5,570	TVMPLSAPTL	0.7
A*02:06	6,165	6,175	YVYNPFMIDV	0.7
A*02:06	6,615	6,625	KQASLNGVTL	0.7
A*03:01	641	651	FLRDGWEIVK	0.7
A*03:01	1,989	1,999	KLLHKPIVWH	0.7
A*03:01	3,716	3,726	LMNVLTLVYK	0.7
A*11:01	5,968	5,978	LCVDIPGIPK	0.7
A*24:02	5,626	5,636	LYYPSARIVY	0.7
A*29:02	946	956	QYEYGTEDDY	0.7
A*29:02	1,864	1,874	YKGPITDVFY	0.7
A*32:01	5,304	5,314	LTNDNTSRYW	0.7
A*32:01	7,056	7,066	KLRGTAVMSL	0.7
A*68:01	4,406	4,416	NRVCGVSAAR	0.7
A*68:02	840	850	NITFELDERI	0.7
B*07:02	1,643	1,653	TDPSFLGRYM	0.7
B*13:02	2,194	2,204	RIKASMPTTI	0.7
B*15:01	402	412	RTIAFGGCVF	0.7
B*15:01	3,325	3,335	LLIRKSNHNF	0.7
B*15:03	3,341	3,351	VQLRVIGHSM	0.7
B*35:01	1,878	1,888	YTTTIKPVTY	0.7
B*35:01	3,141	3,151	VPFWITIAYI	0.7
B*35:03	3,106	3,116	LPGVYSVIYL	0.7
B*38:01	6,302	6,312	THSDKFTDGV	0.7
B*38:01	6,889	6,899	RQWLPTGTLL	0.7
B*44:02	1,139	1,149	AYENFNQHEV	0.7
B*44:02	1,507	1,517	IETISLAGSY	0.7
B*44:02	3,705	3,715	VYDDGARRVW	0.7
B*44:02	5,572	5,582	QEHYVRITGL	0.7
B*44:03	254	264	PFEIKLAKKF	0.7
B*45:01	53	63	LVEVEKGVLP	0.7
B*45:01	2,997	3,007	SEAGVCVSTS	0.7
B*46:01	76	86	ARTAPHGHVM	0.7
B*46:01	2,349	2,359	AAIMQLFFSY	0.7
B*49:01	1,130	1,140	GEDIQLLKSA	0.7
B*51:01	294	304	DGFMGRIRSV	0.7
B*53:01	404	414	IAFGGCVFSY	0.7

B*53:01	2,324	2,334	TAFGLVAEWF	0.7
B*53:01	2,935	2,945	VPYCYDTNVL	0.7
B*53:01	4,076	4,086	MVVIPDYNTY	0.7
B*57:01	1,880	1,890	TTIKPVTYKL	0.7
B*58:01	2,594	2,604	KMFDAYVNTF	0.7
B*58:01	3,246	3,256	FSNSGSDVLY	0.7
C*01:02	6,130	6,140	KIGPERTCCL	0.7
C*02:02	669	679	KEIKESVQTF	0.7
C*03:04	1,430	1,440	YTSKTTVASL	0.7
C*03:04	3,947	3,957	QAIASEFSSL	0.7
C*07:01	3,168	3,178	RRVVFNGVSF	0.7
C*07:02	6,582	6,592	GRVDGQVDLF	0.7
C*08:01	6,805	6,815	SQAWQPGVAM	0.7
C*08:02	1,420	1,430	VVDYGARFYF	0.7
C*15:02	1,880	1,890	TTIKPVTYKL	0.7
C*16:01	6,673	6,683	LAMDEFIERY	0.7
A*01:01	5,265	5,275	PLTKHPNQEY	0.71
A*01:01	5,702	5,712	FDEISMATNY	0.71
A*02:01	1,266	1,276	GNLHPDSATL	0.71
A*02:02	2,785	2,795	FLFVAAIFYL	0.71
A*02:05	468	478	KLNEEIAIIL	0.71
A*02:05	1,817	1,827	AQYELKHGTF	0.71
A*02:05	2,126	2,136	TLATHGLAAV	0.71
A*02:06	984	994	WLDDDSQQTV	0.71
A*02:06	1,095	1,105	YIATNGPLKV	0.71
A*02:06	1,275	1,285	LVSDIDITFL	0.71
A*02:06	6,805	6,815	SQAWQPGVAM	0.71
A*03:01	5,390	5,400	TQLYLGGMSY	0.71
A*11:01	4,035	4,045	QIMLFTMLRK	0.71
A*11:01	4,721	4,731	TSFGPLVRKI	0.71
A*25:01	6,168	6,178	NPFMIDVQQW	0.71
A*26:01	1,012	1,022	EVQPQLEMEL	0.71
A*26:01	3,715	3,725	TLMNVLTLVY	0.71
A*29:02	3,610	3,620	FLYENAFLPF	0.71
A*31:01	2,378	2,388	QMAPISAMVR	0.71
A*31:01	3,447	3,457	GNFYGPFVDR	0.71
A*32:01	3,262	3,272	ITSAVLQSGF	0.71
A*33:01	2,806	2,816	DFSSEIIGYK	0.71
A*68:01	3,817	3,827	DYLVSTQEFR	0.71
A*68:01	4,561	4,571	DFVENPDILR	0.71
A*68:02	859	869	AYTVELGTEV	0.71
A*68:02	2,555	2,565	EESSAKSASV	0.71
A*68:02	5,164	5,174	NSTYASQGLV	0.71

A*68:02	6,478	6,488	EVPVSIINNT	0.71
A*68:02	6,906	6,916	FVSDADSTLI	0.71
B*07:02	1,953	1,963	KKPASRELKV	0.71
B*07:02	5,228	5,238	YPDPSRILGA	0.71
B*07:02	6,523	6,533	VPEVKILNNL	0.71
B*15:01	6,847	6,857	VAKYTQLCQY	0.71
B*27:05	722	732	KSREETGLLM	0.71
B*27:05	1,174	1,184	VRTNVYLAVF	0.71
B*27:05	7,081	7,091	GRLIIRENNR	0.71
B*35:01	2,590	2,600	EVAVKMFDAY	0.71
B*35:03	4,067	4,077	IPLTTAAKLM	0.71
B*37:01	3,816	3,826	YDYLVSTQEF	0.71
B*38:01	1,614	1,624	PHNSHEGKTF	0.71
B*38:01	6,384	6,394	SHGKQVVSDI	0.71
B*39:01	2,129	2,139	THGLAAVNSV	0.71
B*40:01	1,249	1,259	EETKFLTENL	0.71
B*44:03	932	942	EEGDCEEEEF	0.71
B*44:03	2,865	2,875	REVGFVVPGL	0.71
B*45:01	1,381	1,391	REMLAHAEET	0.71
B*45:01	3,556	3,566	EDEFTPFDVV	0.71
B*45:01	5,318	5,328	YEAMYTPHTV	0.71
B*46:01	1,402	1,412	AIVSTIQRKY	0.71
B*46:01	3,134	3,144	MVMFTPLVPF	0.71
B*46:01	5,972	5,982	IPGIPKDMTY	0.71
B*49:01	552	562	LETAQNSVRV	0.71
B*51:01	4,628	4,638	VPVVDSYYSL	0.71
B*53:01	2,004	2,014	NKATYKPNTW	0.71
B*53:01	6,054	6,064	YVDTPDNTDF	0.71
B*57:01	2,560	2,570	KSASVYYSQL	0.71
B*57:01	3,000	3,010	GVCVSTSGRW	0.71
B*57:01	5,985	5,995	ISMMGFKMNY	0.71
B*57:01	6,007	6,017	EEAIRHVRAW	0.71
B*57:01	6,347	6,357	GSLYVNKHAF	0.71
B*57:01	6,651	6,661	FTQSRNLQEF	0.71
B*58:01	4,265	4,275	VPANSTVLSF	0.71
C*02:02	1,817	1,827	AQYELKHGTF	0.71
C*02:02	2,324	2,334	TAFGLVAEWF	0.71
C*02:02	5,116	5,126	YVRNLQHRLY	0.71
C*04:01	30	40	GFGDSVEEVL	0.71
C*04:01	1,325	1,335	VPTDNYITTY	0.71
C*04:43	30	40	GFGDSVEEVL	0.71
C*04:43	1,325	1,335	VPTDNYITTY	0.71
C*07:01	1,549	1,559	FHLDGEVITF	0.71

C*07:01	5,272	5,282	QEYADVFHLY	0.71
C*07:02	1,863	1,873	EYKGPITDVF	0.71
C*08:01	5,725	5,735	YVYIGDPAQL	0.71
C*08:02	2,910	2,920	YTDFATSACV	0.71
C*12:03	5,003	5,013	YSDVENPHLM	0.71
C*14:02	2,343	2,353	FYVLGLAAIM	0.71
C*17:01	3,690	3,700	YASAVVLLIL	0.71
C*17:03	3,690	3,700	YASAVVLLIL	0.71
A*01:01	5,216	5,226	LVKQGDDYVY	0.72
A*02:01	4,635	4,645	YSLLMPILTL	0.72
A*02:02	60	70	VLPQLEQPYV	0.72
A*02:02	1,019	1,029	MELTPVVQTI	0.72
A*02:02	1,195	1,205	FLEMKSEKQV	0.72
A*02:02	5,051	5,061	RLANECAQVL	0.72
A*02:02	5,645	5,655	ALCEKALKYL	0.72
A*02:02	7,048	7,058	FDMSKFPLKL	0.72
A*02:05	1,434	1,444	TTVASLINTL	0.72
A*02:05	3,704	3,714	TVYDDGARRV	0.72
A*02:05	4,044	4,054	KLDNDALNNI	0.72
A*11:01	1,403	1,413	IVSTIQRKYK	0.72
A*11:01	1,776	1,786	MGTLSYEQFK	0.72
A*23:01	2,629	2,639	VSLDNVLSTF	0.72
A*23:01	6,680	6,690	ERYKLEGYAF	0.72
A*26:01	1,895	1,905	TEIDPKLDNY	0.72
A*26:01	4,468	4,478	VVKRHTFSNY	0.72
A*29:02	938	948	EEEFEPSTQY	0.72
A*29:02	3,420	3,430	IDYDCVSFCY	0.72
A*29:02	5,566	5,576	APTLVPQEHY	0.72
A*31:01	5,974	5,984	GIPKDMTYRR	0.72
A*32:01	2,359	2,369	FAVHFISNSW	0.72
A*32:01	2,709	2,719	AKSHNIALIW	0.72
B*07:02	3,834	3,844	LPPKNSIDAF	0.72
B*08:01	5,920	5,930	EIPRRNVATL	0.72
B*14:02	3,371	3,381	FVRIQPGQTF	0.72
B*35:03	4,294	4,304	QPITNCVKML	0.72
B*35:03	4,857	4,867	LPTMCDIRQL	0.72
B*38:01	1,817	1,827	AQYELKHGTF	0.72
B*38:01	5,206	5,216	PHEFCSQHTM	0.72
B*40:01	55	65	EVEKGVLPQL	0.72
B*40:01	843	853	FELDERIDKV	0.72
B*40:02	2,272	2,282	REGYLNSTNV	0.72
B*44:02	2,035	2,045	SEDAQGMDNL	0.72
B*44:03	3,181	3,191	EEAALCTFLL	0.72

B*44:03	5,054	5,064	NECAQVLSEM	0.72
B*44:03	5,572	5,582	QEHYVRITGL	0.72
B*45:01	3,885	3,895	RVESSSKLWA	0.72
B*46:01	1,812	1,822	MSAPPAQYEL	0.72
B*46:01	6,118	6,128	HGFELTSMKY	0.72
B*49:01	3,913	3,923	FEKMVSLLSV	0.72
B*51:01	1,625	1,635	VLPNDDTLRV	0.72
B*51:01	3,518	3,528	LGPLSAQTGI	0.72
B*53:01	6,673	6,683	LAMDEFIERY	0.72
B*58:01	1,642	1,652	TTDPSFLGRY	0.72
B*58:01	2,297	2,307	LSGLDSLDTY	0.72
C*01:02	306	316	VASPNECNQM	0.72
C*05:01	2,630	2,640	SLDNVLSTFI	0.72
C*07:02	4,825	4,835	FFKEGSSVEL	0.72
C*08:01	6,637	6,647	KKVDGVVQQL	0.72
C*08:02	1,010	1,020	IVEVQPQLEM	0.72
C*12:02	2,500	2,510	TVKNGSIHLY	0.72
C*12:02	3,222	3,232	FSGAMDTTSY	0.72
C*14:02	940	950	EFEPSTQYEY	0.72
A*01:01	1,420	1,430	VVDYGARFYF	0.73
A*02:02	3,126	3,136	SFLAHIQWMV	0.73
A*02:02	5,369	5,379	KLVLSVNPYV	0.73
A*02:05	1,441	1,451	NTLNDLNETL	0.73
A*03:01	5,396	5,406	GMSYYCKSHK	0.73
A*23:01	2,361	2,371	VHFISNSWLM	0.73
A*25:01	152	162	DPYEDFQENW	0.73
A*29:02	1,773	1,783	VMYMGTLSYE	0.73
A*29:02	2,277	2,287	NSTNVTIATY	0.73
A*29:02	4,859	4,869	TMCDIRQLLF	0.73
A*31:01	2,185	2,195	CTFTRSTNSR	0.73
A*31:01	4,957	4,967	VSICSTMTNR	0.73
A*31:01	5,532	5,542	GDYGDAVVYR	0.73
A*32:01	603	613	GGVVQLTSQW	0.73
A*32:01	2,387	2,397	RMYIFFASFY	0.73
A*32:01	4,656	4,666	TDLTKPYIKW	0.73
A*68:01	1,301	1,311	LTAVVIPTKK	0.73
A*68:02	4,368	4,378	FTLKNTVCTV	0.73
B*07:02	1,100	1,110	GPLKVGGSCV	0.73
B*15:01	2,431	2,441	IVNGVRRSFY	0.73
B*15:01	3,168	3,178	RRVVFNGVSF	0.73
B*15:01	3,246	3,256	FSNSGSDVLY	0.73
B*15:01	4,538	4,548	TLKEILVTYN	0.73
B*15:03	4,989	4,999	SKFYGGWHNM	0.73

B*18:01	3,557	3,567	DEFTPFDVVR	0.73
B*18:01	6,946	6,956	NDSKEGFFTY	0.73
B*27:05	5,667	5,677	ARVECFDKFK	0.73
B*27:05	7,056	7,066	KLRGTAVMSL	0.73
B*35:01	5,861	5,871	TVDSSQGSEY	0.73
B*35:03	1,981	1,991	TPSFKKGAKL	0.73
B*37:01	1,691	1,701	IELKFNPPAL	0.73
B*38:01	1,071	1,081	KHGGGVAGAL	0.73
B*40:01	384	394	LAEYHNESGL	0.73
B*40:01	639	649	VEFLRDGWEI	0.73
B*40:01	1,398	1,408	VETKAIVSTI	0.73
B*40:02	55	65	EVEKGVLPQL	0.73
B*44:02	1,199	1,209	KSEKQVEQKI	0.73
B*44:02	5,054	5,064	NECAQVLSEM	0.73
B*44:02	5,702	5,712	FDEISMATNY	0.73
B*49:01	4,211	4,221	YTELEPPCRF	0.73
B*51:01	3,559	3,569	FTPFDVVRQC	0.73
B*58:01	6,534	6,544	VDIAANTVIW	0.73
C*01:02	5,725	5,735	YVYIGDPAQL	0.73
C*06:02	5,403	5,413	SHKPPISFPL	0.73
C*07:02	5,273	5,283	EYADVFHLYL	0.73
C*08:01	1,542	1,552	YTSNPTTFHL	0.73
C*08:02	4,630	4,640	VVDSYYSLLM	0.73
C*12:02	3,076	3,086	FGEYSHVVAF	0.73
C*14:02	3,486	3,496	WFLNRFTTTL	0.73
C*15:02	1,030	1,040	VNSFSGYLKL	0.73
C*15:02	4,221	4,231	VTDTPKGPKV	0.73
A*01:01	3,779	3,789	NTLQCIMLVY	0.74
A*02:01	2,469	2,479	FISDEVARDL	0.74
A*02:01	3,103	3,113	YSFLPGVYSV	0.74
A*02:01	3,119	3,129	FYLTNDVSFL	0.74
A*02:01	4,592	4,602	AMRNAGIVGV	0.74
A*02:05	83	93	HVMVELVAEL	0.74
A*02:05	4,930	4,940	RNVIPTITQM	0.74
A*02:05	6,906	6,916	FVSDADSTLI	0.74
A*02:06	3,297	3,307	GLWLDDVVYC	0.74
A*02:06	5,250	5,260	TLMIERFVSL	0.74
A*11:01	116	126	VAYRKVLLRK	0.74
A*11:01	3,950	3,960	ASEFSSLPSY	0.74
A*23:01	4,433	4,443	DIYNDKVAGF	0.74
A*23:01	5,141	5,151	EFYAYLRKHF	0.74
A*23:01	6,224	6,234	IEYPIIGDEL	0.74
A*25:01	5,725	5,735	YVYIGDPAQL	0.74

A*29:02	6,946	6,956	NDSKEGFFTY	0.74
A*31:01	1,159	1,169	FGADPIHSLR	0.74
A*32:01	1,510	1,520	ISLAGSYKDW	0.74
A*32:01	2,598	2,608	AYVNTFSSTF	0.74
B*07:02	5,225	5,235	YLPYPDPSRI	0.74
B*14:02	1,549	1,559	FHLDGEVITF	0.74
B*14:02	3,824	3,834	EFRYMNSQGL	0.74
B*15:01	3,553	3,563	ALLEDEFTPF	0.74
B*15:01	5,120	5,130	LQHRLYECLY	0.74
B*15:01	6,289	6,299	KAYKIEELFY	0.74
B*15:01	6,830	6,840	LQNYGDSATL	0.74
B*27:05	6,654	6,664	SRNLQEFKPR	0.74
B*35:03	1,394	1,404	MPVCVETKAI	0.74
B*40:01	538	548	SEAARVVRSI	0.74
B*40:01	3,077	3,087	GEYSHVVAFN	0.74
B*44:02	1,016	1,026	QLEMELTPVV	0.74
B*44:02	3,181	3,191	EEAALCTFLL	0.74
B*49:01	3,076	3,086	FGEYSHVVAF	0.74
B*53:01	3,037	3,047	TPLIQPIGAL	0.74
B*53:01	3,203	3,213	LLPLTQYNRY	0.74
B*58:01	3,953	3,963	FSSLPSYAAF	0.74
B*58:01	5,021	5,031	RAMPNMLRIM	0.74
B*58:01	5,040	5,050	TTCCSLSHRF	0.74
C*02:02	4,009	4,019	MADQAMTQMY	0.74
C*02:02	4,076	4,086	MVVIPDYNTY	0.74
C*03:04	4,777	4,787	MHAASGNLLL	0.74
C*05:01	1,888	1,898	KLDGVVCTEI	0.74
C*05:01	6,778	6,788	TIDYTEISFM	0.74
C*06:02	5,116	5,126	YVRNLQHRLY	0.74
C*07:02	5,403	5,413	SHKPPISFPL	0.74
C*08:01	136	146	YGADLKSFDL	0.74
C*08:01	5,274	5,284	YADVFHLYLQ	0.74
C*08:02	4,009	4,019	MADQAMTQMY	0.74
C*12:03	76	86	ARTAPHGHVM	0.74
C*15:02	550	560	RTLETAQNSV	0.74
C*16:01	4,000	4,010	AAMQRKLEKM	0.74
C*17:01	4,361	4,371	CANDPVGFTL	0.74
C*17:03	4,361	4,371	CANDPVGFTL	0.74
A*01:01	4,563	4,573	VENPDILRVY	0.75
A*01:01	5,413	5,423	CANGQVFGLY	0.75
A*02:01	1,195	1,205	FLEMKSEKQV	0.75
A*02:01	3,126	3,136	SFLAHIQWMV	0.75
A*02:01	6,106	6,116	NLSDRVVFVL	0.75

A*02:02	2,725	2,735	SLSEQLRKQI	0.75
A*02:02	2,869	2,879	FVVPGLPGTI	0.75
A*02:05	1,556	1,566	ITFDNLKTLL	0.75
A*02:05	6,838	6,848	TLPKGIMMNV	0.75
A*02:06	5,928	5,938	TLQAENVTGL	0.75
A*03:01	3,570	3,580	GVTFQSAVKR	0.75
A*03:01	3,674	3,684	MVDTSLSGFK	0.75
A*03:01	4,637	4,647	LLMPILTLTR	0.75
A*24:02	1,187	1,197	LYDKLVSSFL	0.75
A*24:02	1,721	1,731	AYCNKTVGEL	0.75
A*24:02	2,175	2,185	PYFFTLLLQL	0.75
A*24:02	5,144	5,154	AYLRKHFSMM	0.75
A*24:02	5,279	5,289	HLYLQYIRKL	0.75
A*25:01	1,419	1,429	GVVDYGARFY	0.75
A*25:01	3,111	3,121	SVIYLYLTFY	0.75
A*25:01	4,148	4,158	ELSPVALRQM	0.75
A*25:01	6,946	6,956	NDSKEGFFTY	0.75
A*26:01	2,055	2,065	EVVENPTIQK	0.75
A*26:01	6,750	6,760	SVIDLLLDDF	0.75
A*29:02	3,681	3,691	GFKLKDCVMY	0.75
A*29:02	5,062	5,072	EMVMCGGSLY	0.75
A*31:01	1,181	1,191	AVFDKNLYDK	0.75
A*33:01	4,943	4,953	YAISAKNRAR	0.75
A*68:01	5,780	5,790	TVSALVYDNK	0.75
A*68:02	1,371	1,381	EILGTVSWNL	0.75
A*68:02	2,910	2,920	YTDFATSACV	0.75
B*15:03	525	535	EQKSILSPLY	0.75
B*15:03	6,637	6,647	KKVDGVVQQL	0.75
B*18:01	36	46	EEVLSEARQH	0.75
B*35:03	3,374	3,384	IQPGQTFSVL	0.75
B*38:01	6,490	6,500	TKVDGVDVEL	0.75
B*39:01	2,361	2,371	VHFISNSWLM	0.75
B*40:02	632	642	EEKFKEGVEF	0.75
B*40:02	1,873	1,883	YKENSYTTTI	0.75
B*40:02	2,035	2,045	SEDAQGMDNL	0.75
B*40:02	5,483	5,493	VREVLSDREL	0.75
B*44:02	254	264	PFEIKLAKKF	0.75
B*44:02	932	942	EEGDCEEEEF	0.75
B*44:02	1,140	1,150	YENFNQHEVL	0.75
B*44:02	1,249	1,259	EETKFLTENL	0.75
B*44:02	2,620	2,630	TAEAELAKNV	0.75
B*44:03	1,567	1,577	LREVRTIKVF	0.75
B*46:01	2,327	2,337	GLVAEWFLAY	0.75

B*51:01	3, 100	3, 110	TPVYSFLPGV	0. 75
B*53:01	3, 027	3, 037	DAVNLLTNMF	0. 75
B*57:01	322	332	KCDHCGETSW	0. 75
B*57:01	402	412	RTIAFGGCVF	0. 75
B*57:01	2, 765	2, 775	LKGGKIVNNW	0. 75
B*57:01	3, 705	3, 715	VYDDGARRVW	0. 75
B*58:01	402	412	RTIAFGGCVF	0. 75
B*58:01	5, 401	5, 411	CKSHKPPISF	0. 75
B*58:01	6, 651	6, 661	FTQSRNLQEF	0. 75
C*02:02	2, 317	2, 327	SSFKWDLTAF	0. 75
C*02:02	3, 103	3, 113	YSFLPGVYSV	0. 75
C*03:04	4, 127	4, 137	LAWPLIVTAL	0. 75
C*03:04	4, 361	4, 371	CANDPVGFTL	0. 75
C*05:01	722	732	KSREETGLLM	0. 75
C*05:01	4, 009	4, 019	MADQAMTQMY	0. 75
C*05:01	4, 502	4, 512	RIDGDMVPHI	0. 75
C*07:02	3, 489	3, 499	NRFTTTLNDF	0. 75
C*07:04	1, 540	1, 550	VYYTSNPTTF	0. 75
C*07:04	5, 273	5, 283	EYADVFHLYL	0. 75
C*12:02	4, 009	4, 019	MADQAMTQMY	0. 75
C*12:03	6, 673	6, 683	LAMDEFIERY	0. 75
C*14:02	2, 602	2, 612	TFSSTFNVPM	0. 75
C*16:01	3, 953	3, 963	FSSLPSYAAF	0. 75
C*16:01	6, 651	6, 661	FTQSRNLQEF	0. 75
A*01:01	173	183	LMRELNGGAY	0. 76
A*01:01	2, 629	2, 639	VSLDNVLSTF	0. 76
A*01:01	2, 964	2, 974	GSIIQFPNTY	0. 76
A*01:01	3, 715	3, 725	TLMNVLTLVY	0. 76
A*01:01	5, 175	5, 185	SIKNFKSVLY	0. 76
A*02:01	4, 484	4, 494	YNLLKDCPAV	0. 76
A*02:02	1, 149	1, 159	LLAPLLSAGI	0. 76
A*02:02	2, 960	2, 970	VLMDGSIIQF	0. 76
A*02:05	843	853	FELDERIDKV	0. 76
A*02:05	4, 008	4, 018	KMADQAMTQM	0. 76
A*02:06	468	478	KLNEEIAIIL	0. 76
A*02:06	3, 100	3, 110	TPVYSFLPGV	0. 76
A*02:06	3, 336	3, 346	VQAGNVQLRV	0. 76
A*02:06	4, 044	4, 054	KLDNDALNNI	0. 76
A*02:06	4, 930	4, 940	RNVIPTITQM	0. 76
A*02:06	6, 113	6, 123	FVLWAHGFEL	0. 76
A*02:06	6, 838	6, 848	TLPKGIMMNV	0. 76
A*03:01	2, 723	2, 733	FMSLSEQLRK	0. 76
A*23:01	95	105	IQYGRSGETL	0. 76

A*25:01	5,560	5,570	TVMPLSAPTL	0.76
A*26:01	59	69	GVLPQLEQPY	0.76
A*26:01	134	144	HSYGADLKSF	0.76
A*26:01	490	500	TVKGLDYKAF	0.76
A*26:01	3,094	3,104	FTVLCLTPVY	0.76
A*26:01	3,207	3,217	TQYNRYLALY	0.76
A*26:01	6,648	6,658	ETYFTQSRNL	0.76
A*29:02	220	230	DFIDTKRGVY	0.76
A*29:02	3,210	3,220	NRYLALYNKY	0.76
A*29:02	4,683	4,693	YFKYWDQTYH	0.76
A*29:02	5,972	5,982	IPGIPKDMTY	0.76
A*29:02	6,847	6,857	VAKYTQLCQY	0.76
A*32:01	2,516	2,526	KTYERHSLSH	0.76
A*33:01	2,511	2,521	DKAGQKTYER	0.76
A*68:02	1,147	1,157	EVLLAPLLSA	0.76
A*68:02	1,249	1,259	EETKFLTENL	0.76
A*68:02	6,796	6,806	ETFYPKLQSS	0.76
B*07:02	776	786	APLVGTPVCI	0.76
B*08:01	79	89	APHGHVMVEL	0.76
B*14:02	6,352	6,362	NKHAFHTPAF	0.76
B*15:01	3,944	3,954	ATLQAIASEF	0.76
B*15:01	4,563	4,573	VENPDILRVY	0.76
B*15:01	5,154	5,164	ILSDDAVVCF	0.76
B*15:01	5,287	5,297	KLHDELTGHM	0.76
B*15:03	1,549	1,559	FHLDGEVITF	0.76
B*35:01	2,935	2,945	VPYCYDTNVL	0.76
B*35:03	359	369	YLPQNAVVKI	0.76
B*35:03	2,113	2,123	KKPNELSRVL	0.76
B*35:03	6,479	6,489	VPVSIINNTV	0.76
B*37:01	672	682	KESVQTFFKL	0.76
B*39:01	6,242	6,252	VQHMVVKAAL	0.76
B*40:01	5,054	5,064	NECAQVLSEM	0.76
B*40:02	5,271	5,281	NQEYADVFHL	0.76
B*44:02	951	961	TEDDYQGKPL	0.76
B*46:01	2,598	2,608	AYVNTFSSTF	0.76
B*49:01	5,529	5,539	FEKGDYGDAV	0.76
B*57:01	2,594	2,604	KMFDAYVNTF	0.76
B*57:01	2,964	2,974	GSIIQFPNTY	0.76
B*57:01	4,492	4,502	AVAKHDFFKF	0.76
B*57:01	6,363	6,373	KSAFVNLKQL	0.76
B*58:01	5,188	5,198	NVFMSEAKCW	0.76
C*01:02	733	743	LKAPKEIIFL	0.76
C*01:02	5,920	5,930	EIPRRNVATL	0.76

C*02:02	2,964	2,974	GSIIQFPNTY	0.76
C*02:02	4,423	4,433	TSTDVVYRAF	0.76
C*02:02	6,432	6,442	ISAGFSLWVY	0.76
C*03:02	6,054	6,064	YVDTPDNTDF	0.76
C*07:01	206	216	ARAGKASCTL	0.76
C*08:02	5,274	5,284	YADVFHLYLQ	0.76
C*16:01	4,265	4,275	VPANSTVLSF	0.76
C*16:01	6,298	6,308	YSYATHSDKF	0.76
C*17:01	5,560	5,570	TVMPLSAPTL	0.76
C*17:03	5,560	5,570	TVMPLSAPTL	0.76
A*01:01	3,200	3,210	SDVLLPLTQY	0.77
A*02:01	14	24	VQLSLPVLQV	0.77
A*02:02	757	767	VLKTGDLQPL	0.77
A*02:05	77	87	RTAPHGHVMV	0.77
A*02:05	437	447	GVVGEGSEGL	0.77
A*02:06	1,603	1,613	YLDGADVTKI	0.77
A*02:06	6,474	6,484	GQQGEVPVSI	0.77
A*03:01	6,707	6,717	LLIGLAKRFK	0.77
A*11:01	872	882	ACVVADAVIK	0.77
A*23:01	2,335	2,345	AYILFTRFFY	0.77
A*23:01	2,885	2,895	DFLHFLPRVF	0.77
A*23:01	4,444	4,454	KFLKTNCCRF	0.77
A*23:01	4,859	4,869	TMCDIRQLLF	0.77
A*24:02	4,094	4,104	FTYASALWEI	0.77
A*25:01	4,734	4,744	GVPFVVSTGY	0.77
A*25:01	5,077	5,087	TSSGDATTAY	0.77
A*26:01	6,067	6,077	SAKPPPGDQF	0.77
A*26:01	6,999	7,009	NVNASSSEAF	0.77
A*29:02	1,461	1,471	GLNLEEAARY	0.77
A*31:01	4,013	4,023	AMTQMYKQAR	0.77
A*68:02	738	748	EIIFLEGETL	0.77
B*08:01	6,106	6,116	NLSDRVVFVL	0.77
B*13:02	6,830	6,840	LQNYGDSATL	0.77
B*15:01	1,179	1,189	YLAVFDKNLY	0.77
B*15:01	6,438	6,448	LWVYKQFDTY	0.77
B*15:01	6,664	6,674	SQMEIDFLEL	0.77
B*15:03	3,756	3,766	VMFLARGIVF	0.77
B*15:03	5,175	5,185	SIKNFKSVLY	0.77
B*35:01	2,557	2,567	SSAKSASVYY	0.77
B*35:01	3,058	3,068	VAIVVTCLAY	0.77
B*37:01	2,471	2,481	SDEVARDLSL	0.77
B*38:01	1,267	1,277	NLHPDSATLV	0.77
B*40:01	6,889	6,899	RQWLPTGTLL	0.77

B*40:02	232	242	REHEHEIAWY	0.77
B*44:02	2,808	2,818	SSEIIGYKAI	0.77
B*44:03	36	46	EEVLSEARQH	0.77
B*45:01	4,831	4,841	SVELKHFFFA	0.77
B*46:01	2,509	2,519	YFDKAGQKTY	0.77
B*49:01	773	783	AVEAPLVGTP	0.77
B*51:01	5,318	5,328	YEAMYTPHTV	0.77
B*53:01	2,486	2,496	INPTDQSSYI	0.77
C*01:02	4,622	4,632	TTPGSGVPVV	0.77
C*02:02	1,419	1,429	GVVDYGARFY	0.77
C*03:02	4,211	4,221	YTELEPPCRF	0.77
C*03:02	5,390	5,400	TQLYLGGMSY	0.77
C*03:04	1,158	1,168	IFGADPIHSL	0.77
C*07:01	5,718	5,728	ARLRAKHYVY	0.77
C*07:02	1,631	1,641	TLRVEAFEYY	0.77
C*07:04	6,330	6,340	RFDTRVLSNL	0.77
C*12:02	4,076	4,086	MVVIPDYNTY	0.77
C*16:01	3,910	3,920	TEAFEKMVSL	0.77
A*01:01	2,104	2,114	YVDNSSLTIK	0.78
A*01:01	5,537	5,547	AVVYRGTTTY	0.78
A*02:01	3,494	3,504	TLNDFNLVAM	0.78
A*02:02	1,291	1,301	IVGDVVQEGV	0.78
A*02:05	5,560	5,570	TVMPLSAPTL	0.78
A*02:06	279	289	SIIKTIQPRV	0.78
A*02:06	3,017	3,027	RSLPGVFCGV	0.78
A*03:01	175	185	RELNGGAYTR	0.78
A*03:01	2,387	2,397	RMYIFFASFY	0.78
A*11:01	4,899	4,909	AGFPFNKWGK	0.78
A*11:01	6,096	6,106	IVQMLSDTLK	0.78
A*23:01	3,371	3,381	FVRIQPGQTF	0.78
A*23:01	3,807	3,817	RYFRLTLGVY	0.78
A*23:01	5,144	5,154	AYLRKHFSMM	0.78
A*23:01	6,271	6,281	VPQADVEWKF	0.78
A*24:02	1,904	1,914	YYKKDNSYFT	0.78
A*24:02	2,396	2,406	YYVWKSYVHV	0.78
A*25:01	591	601	ATNNLVVMAY	0.78
A*25:01	1,434	1,444	TTVASLINTL	0.78
A*25:01	1,590	1,600	DMSMTYGQQF	0.78
A*25:01	3,477	3,487	YAAVINGDRW	0.78
A*29:02	2,930	2,940	ASGKPVPYCY	0.78
A*31:01	4,737	4,747	FVVSTGYHFR	0.78
A*32:01	2,131	2,141	GLAAVNSVPW	0.78
A*32:01	2,194	2,204	RIKASMPTTI	0.78

A*32:01	6,269	6,279	KCVPQADVEW	0.78
A*68:01	5,640	5,650	HAAVDALCEK	0.78
A*68:02	615	625	NIFGTVYEKL	0.78
B*07:02	4,150	4,160	SPVALRQMSC	0.78
B*07:02	5,322	5,332	YTPHTVLQAV	0.78
B*15:01	3,610	3,620	FLYENAFLPF	0.78
B*18:01	5,525	5,535	GEYTFEKGDY	0.78
B*27:05	206	216	ARAGKASCTL	0.78
B*27:05	1,631	1,641	TLRVEAFEYY	0.78
B*27:05	3,883	3,893	QLRVESSSKL	0.78
B*35:01	3,037	3,047	TPLIQPIGAL	0.78
B*35:01	4,341	4,351	HPNPKGFCDL	0.78
B*35:03	2,953	2,963	LRPDTRYVLM	0.78
B*35:03	4,066	4,076	IIPLTTAAKL	0.78
B*37:01	724	734	REETGLLMPL	0.78
B*38:01	5,732	5,742	AQLPAPRTLL	0.78
B*44:02	1,567	1,577	LREVRTIKVF	0.78
B*45:01	869	879	NEFACVVADA	0.78
B*46:01	1,179	1,189	YLAVFDKNLY	0.78
B*46:01	4,468	4,478	VVKRHTFSNY	0.78
B*49:01	724	734	REETGLLMPL	0.78
B*51:01	877	887	DAVIKTLQPV	0.78
B*53:01	4,120	4,130	SMDNSPNLAW	0.78
B*57:01	3,646	3,656	LPSLATVAYF	0.78
C*02:02	1,179	1,189	YLAVFDKNLY	0.78
C*02:02	1,642	1,652	TTDPSFLGRY	0.78
C*03:02	5,972	5,982	IPGIPKDMTY	0.78
C*04:01	1,603	1,613	YLDGADVTKI	0.78
C*04:43	1,603	1,613	YLDGADVTKI	0.78
C*05:01	5,274	5,284	YADVFHLYLQ	0.78
C*07:02	5,002	5,012	VYSDVENPHL	0.78
C*14:02	3,610	3,620	FLYENAFLPF	0.78
C*15:02	1,095	1,105	YIATNGPLKV	0.78
C*15:02	5,617	5,627	KSHFAIGLAL	0.78
C*17:01	2,965	2,975	SIIQFPNTYL	0.78
C*17:03	2,965	2,975	SIIQFPNTYL	0.78
A*01:01	1,461	1,471	GLNLEEAARY	0.79
A*01:01	4,630	4,640	VVDSYYSLLM	0.79
A*01:01	6,272	6,282	PQADVEWKFY	0.79
A*01:01	6,282	6,292	DAQPCSDKAY	0.79
A*01:01	6,289	6,299	KAYKIEELFY	0.79
A*01:01	6,341	6,351	LPGCDGGSLY	0.79
A*02:02	3,598	3,608	VLVQSTQWSL	0.79

A*02:05	2,605	2,615	STFNVPMEKL	0.79
A*02:05	6,105	6,115	KNLSDRVVFV	0.79
A*02:06	2,397	2,407	YVWKSYVHVV	0.79
A*03:01	518	528	KGAWNIGEQK	0.79
A*03:01	3,978	3,988	SEVVLKKLKK	0.79
A*03:01	5,596	5,606	ANYQKVGMQK	0.79
A*03:01	6,870	6,880	VIHFGAGSDK	0.79
A*23:01	2,396	2,406	YYVWKSYVHV	0.79
A*23:01	2,791	2,801	IFYLIIPVHV	0.79
A*23:01	6,713	6,723	KRFKESPFEL	0.79
A*24:02	2,972	2,982	TYLEGSVRVV	0.79
A*24:02	6,713	6,723	KRFKESPFEL	0.79
A*25:01	1,652	1,662	MSALNHTKKW	0.79
A*25:01	4,897	4,907	KSAGFPFNKW	0.79
A*26:01	3,950	3,960	ASEFSSLPSY	0.79
A*29:02	4,904	4,914	NKWGKARLYY	0.79
A*32:01	404	414	IAFGGCVFSY	0.79
A*32:01	6,271	6,281	VPQADVEWKF	0.79
A*68:01	2,378	2,388	QMAPISAMVR	0.79
A*68:02	2,490	2,500	DQSSYIVDSV	0.79
A*68:02	2,807	2,817	FSSEIIGYKA	0.79
B*15:01	1,615	1,625	HNSHEGKTFY	0.79
B*15:01	5,077	5,087	TSSGDATTAY	0.79
B*15:03	3,168	3,178	RRVVFNGVSF	0.79
B*15:03	6,830	6,840	LQNYGDSATL	0.79
B*37:01	6,028	6,038	REAVGTNLPL	0.79
B*38:01	242	252	TERSEKSYEL	0.79
B*38:01	1,842	1,852	KHITSKETLY	0.79
B*38:01	6,197	6,207	AHVASCDAIM	0.79
B*40:01	912	922	GEFKLASHMY	0.79
B*40:01	6,943	6,953	TKENDSKEGF	0.79
B*40:02	2,024	2,034	VETSNSFDVL	0.79
B*53:01	5,500	5,510	KPRPPLNRNY	0.79
C*02:02	1,586	1,596	TQVVDMSMTY	0.79
C*03:04	4,635	4,645	YSLLMPILTL	0.79
C*04:01	2,517	2,527	TYERHSLSHF	0.79
C*04:01	5,154	5,164	ILSDDAVVCF	0.79
C*04:43	2,517	2,527	TYERHSLSHF	0.79
C*04:43	5,154	5,164	ILSDDAVVCF	0.79
C*12:02	4,265	4,275	VPANSTVLSF	0.79
C*12:02	5,077	5,087	TSSGDATTAY	0.79
C*12:02	5,303	5,313	MLTNDNTSRY	0.79
C*12:03	1,482	1,492	VSSPDAVTAY	0.79

C*14:02	4,233	4,243	LYFIKGLNNL	0.79
C*16:01	4,423	4,433	TSTDVVYRAF	0.79
C*16:01	5,077	5,087	TSSGDATTAY	0.79
C*17:01	1,275	1,285	LVSDIDITFL	0.79
C*17:03	1,275	1,285	LVSDIDITFL	0.79
A*01:01	5,385	5,395	DVTDVTQLYL	0.8
A*01:01	6,145	6,155	TCFSTASDTY	0.8
A*02:02	83	93	HVMVELVAEL	0.8
A*02:02	1,566	1,576	SLREVRTIKV	0.8
A*02:02	2,336	2,346	YILFTRFFYV	0.8
A*02:02	5,111	5,121	KIADKYVRNL	0.8
A*02:02	5,460	5,470	KLFAAETLKA	0.8
A*02:05	3,644	3,654	FLLPSLATVA	0.8
A*02:06	4,126	4,136	NLAWPLIVTA	0.8
A*11:01	1,787	1,797	GVQIPCTCGK	0.8
A*11:01	6,557	6,567	GVCSMTDIAK	0.8
A*24:02	3,747	3,757	SNYSGVVTTV	0.8
A*26:01	6,726	6,736	IPMDSTVKNY	0.8
A*29:02	1,654	1,664	ALNHTKKWKY	0.8
A*29:02	4,465	4,475	SYFVVKRHTF	0.8
A*31:01	6,518	6,528	RNIKPVPEVK	0.8
A*32:01	3,756	3,766	VMFLARGIVF	0.8
A*32:01	5,617	5,627	KSHFAIGLAL	0.8
A*33:01	15	25	QLSLPVLQVR	0.8
A*33:01	1,382	1,392	EMLAHAEETR	0.8
A*68:01	217	227	EQLDFIDTKR	0.8
A*68:01	1,382	1,392	EMLAHAEETR	0.8
A*68:02	1,481	1,491	SVSSPDAVTA	0.8
A*68:02	2,473	2,483	EVARDLSLQF	0.8
B*07:02	4,124	4,134	SPNLAWPLIV	0.8
B*13:02	1,207	1,217	KIAEIPKEEV	0.8
B*15:01	612	622	WLTNIFGTVY	0.8
B*15:01	5,531	5,541	KGDYGDAVVY	0.8
B*15:01	5,598	5,608	YQKVGMQKYS	0.8
B*15:03	3,600	3,610	VQSTQWSLFF	0.8
B*27:05	5,666	5,676	RARVECFDKF	0.8
B*35:01	6,814	6,824	MPNLYKMQRM	0.8
B*35:03	2,173	2,183	YMPYFFTLLL	0.8
B*35:03	3,911	3,921	EAFEKMVSLL	0.8
B*37:01	524	534	GEQKSILSPL	0.8
B*37:01	6,287	6,297	SDKAYKIEEL	0.8
B*40:02	1,199	1,209	KSEKQVEQKI	0.8
B*40:02	4,212	4,222	TELEPPCRFV	0.8

B*40:02	5,061	5,071	SEMVMCGGSL	0.8
B*40:02	5,826	5,836	REFLTRNPAW	0.8
B*44:03	754	764	EEVVLKTGDL	0.8
B*44:03	1,010	1,020	IVEVQPQLEM	0.8
B*44:03	1,052	1,062	EEAKKVKPTV	0.8
B*51:01	5,975	5,985	IPKDMTYRRL	0.8
B*53:01	2,199	2,209	MPTTIAKNTV	0.8
B*53:01	4,067	4,077	IPLTTAAKLM	0.8
B*57:01	1,556	1,566	ITFDNLKTLL	0.8
C*02:02	6,805	6,815	SQAWQPGVAM	0.8
C*03:02	4,916	4,926	MSYEDQDALF	0.8
C*04:01	1,540	1,550	VYYTSNPTTF	0.8
C*04:43	1,540	1,550	VYYTSNPTTF	0.8
C*06:02	242	252	TERSEKSYEL	0.8
C*07:01	905	915	YYLFDESGEF	0.8
C*07:01	3,197	3,207	KLRSDVLLPL	0.8
C*07:04	907	917	LFDESGEFKL	0.8
C*08:02	5,288	5,298	LHDELTGHML	0.8
C*08:02	6,778	6,788	TIDYTEISFM	0.8
C*12:02	2,509	2,519	YFDKAGQKTY	0.8
C*12:02	3,910	3,920	TEAFEKMVSL	0.8
C*12:03	1,556	1,566	ITFDNLKTLL	0.8
C*14:02	2,849	2,859	SYTNDKACPL	0.8
C*14:02	3,230	3,240	SYREAACCHL	0.8
C*17:01	4,635	4,645	YSLLMPILTL	0.8
C*17:03	4,635	4,645	YSLLMPILTL	0.8
A*01:01	4,221	4,231	VTDTPKGPKV	0.81
A*01:01	4,457	4,467	DEDDNLIDSY	0.81
A*02:01	467	477	FKLNEEIAII	0.81
A*02:05	2,965	2,975	SIIQFPNTYL	0.81
A*02:05	3,253	3,263	VLYQPPQTSI	0.81
A*11:01	501	511	QIVESCGNFK	0.81
A*23:01	1,679	1,689	CYLATALLTL	0.81
A*24:02	3,112	3,122	VIYLYLTFYL	0.81
A*25:01	4,519	4,529	YTMADLVYAL	0.81
A*26:01	4,168	4,178	CTDDNALAYY	0.81
A*31:01	2,516	2,526	KTYERHSLSH	0.81
A*31:01	3,159	3,169	YWFFSNYLKR	0.81
A*31:01	3,211	3,221	RYLALYNKYK	0.81
A*68:01	4,220	4,230	FVTDTPKGPK	0.81
A*68:01	7,049	7,059	DMSKFPLKLR	0.81
B*07:02	2,199	2,209	MPTTIAKNTV	0.81
B*07:02	6,322	6,332	YPANSIVCRF	0.81

B*14:02	3,910	3,920	TEAFEKMVSL	0.81
B*15:01	3,620	3,630	AMGIIAMSAF	0.81
B*15:01	6,118	6,128	HGFELTSMKY	0.81
B*15:03	6,772	6,782	SKVVKVTIDY	0.81
B*27:05	3,372	3,382	VRIQPGQTFS	0.81
B*27:05	5,129	5,139	YRNRDVDTDF	0.81
B*35:03	5,841	5,851	ISPYNSQNAV	0.81
B*37:01	951	961	TEDDYQGKPL	0.81
B*37:01	2,057	2,067	VENPTIQKDV	0.81
B*40:01	774	784	VEAPLVGTPV	0.81
B*44:03	1,016	1,026	QLEMELTPVV	0.81
B*44:03	1,140	1,150	YENFNQHEVL	0.81
B*45:01	523	533	IGEQKSILSP	0.81
B*45:01	5,464	5,474	AETLKATEET	0.81
B*46:01	2,928	2,938	KDASGKPVPY	0.81
B*51:01	3,660	3,670	MPASWVMRIM	0.81
B*51:01	5,081	5,091	DATTAYANSV	0.81
B*53:01	5,619	5,629	HFAIGLALYY	0.81
B*58:01	3,290	3,300	CGTTTLNGLW	0.81
B*58:01	6,673	6,683	LAMDEFIERY	0.81
C*01:02	1,542	1,552	YTSNPTTFHL	0.81
C*03:02	3,058	3,068	VAIVVTCLAY	0.81
C*04:01	5,226	5,236	LPYPDPSRIL	0.81
C*04:43	5,226	5,236	LPYPDPSRIL	0.81
C*05:01	2,104	2,114	YVDNSSLTIK	0.81
C*05:01	4,771	4,781	YAADPAMHAA	0.81
C*05:01	5,287	5,297	KLHDELTGHM	0.81
C*05:01	6,638	6,648	KVDGVVQQLP	0.81
C*06:02	3,489	3,499	NRFTTTLNDF	0.81
C*07:01	1,706	1,716	RARAGEAANF	0.81
C*07:02	5,309	5,319	TSRYWEPEFY	0.81
C*07:02	6,291	6,301	YKIEELFYSY	0.81
C*12:02	5,675	5,685	FKVNSTLEQY	0.81
C*14:02	4,633	4,643	SYYSLLMPIL	0.81
C*14:02	6,864	6,874	VPYNMRVIHF	0.81
C*15:02	1,555	1,565	VITFDNLKTL	0.81
C*16:01	6,432	6,442	ISAGFSLWVY	0.81
C*17:01	2,091	2,101	ITEEVGHTDL	0.81
C*17:03	2,091	2,101	ITEEVGHTDL	0.81
A*01:01	4,076	4,086	MVVIPDYNTY	0.82
A*02:01	1,441	1,451	NTLNDLNETL	0.82
A*02:01	6,912	6,922	STLIGDCATV	0.82
A*02:02	5,783	5,793	ALVYDNKLKA	0.82

A*02:05	5, 111	5, 121	KIADKYVRNL	0. 82
A*03:01	1, 301	1, 311	LTAVVIPTKK	0. 82
A*03:01	2, 074	2, 084	TEVVGDIILK	0. 82
A*03:01	6, 433	6, 443	SAGFSLWVYK	0. 82
A*11:01	1, 029	1, 039	EVNSFSGYLK	0. 82
A*11:01	1, 813	1, 823	SAPPAQYELK	0. 82
A*11:01	3, 349	3, 359	SMQNCVLKFK	0. 82
A*23:01	2, 006	2, 016	ATYKPNTWCI	0. 82
A*23:01	6, 168	6, 178	NPFMIDVQQW	0. 82
A*24:02	1, 679	1, 689	CYLATALLTL	0. 82
A*24:02	5, 141	5, 151	EFYAYLRKHF	0. 82
A*25:01	1, 482	1, 492	VSSPDAVTAY	0. 82
A*25:01	6, 641	6, 651	GVVQQLPETY	0. 82
A*26:01	1, 325	1, 335	VPTDNYITTY	0. 82
A*26:01	1, 827	1, 837	TCASEYTGNY	0. 82
A*26:01	2, 979	2, 989	RVVTTFDSEY	0. 82
A*26:01	4, 846	4, 856	AISDYDYYRY	0. 82
A*29:02	3, 719	3, 729	VLTLVYKVYY	0. 82
A*31:01	6, 586	6, 596	GQVDLFRNAR	0. 82
A*32:01	22	32	QVRDVLVRGF	0. 82
A*32:01	5, 487	5, 497	LSDRELHLSW	0. 82
A*32:01	6, 615	6, 625	KQASLNGVTL	0. 82
A*68:01	15	25	QLSLPVLQVR	0. 82
A*68:02	33	43	DSVEEVLSEA	0. 82
A*68:02	1, 172	1, 182	DTVRTNVYLA	0. 82
A*68:02	2, 389	2, 399	YIFFASFYYV	0. 82
A*68:02	2, 965	2, 975	SIIQFPNTYL	0. 82
A*68:02	5, 995	6, 005	QVNGYPNMFI	0. 82
B*07:02	764	774	QPLEQPTSEA	0. 82
B*07:02	2, 484	2, 494	RPINPTDQSS	0. 82
B*13:02	2, 661	2, 671	KLSHQSDIEV	0. 82
B*13:02	2, 776	2, 786	KQLIKVTLVF	0. 82
B*14:02	95	105	IQYGRSGETL	0. 82
B*15:01	1, 252	1, 262	KFLTENLLLY	0. 82
B*35:03	4, 224	4, 234	TPKGPKVKYL	0. 82
B*35:03	5, 095	5, 105	QAVTANVNAL	0. 82
B*35:03	6, 523	6, 533	VPEVKILNNL	0. 82
B*44:03	2, 035	2, 045	SEDAQGMDNL	0. 82
B*45:01	232	242	REHEHEIAWY	0. 82
B*46:01	3, 657	3, 667	MVYMPASWVM	0. 82
B*46:01	4, 756	4, 766	VNLHSSRLSF	0. 82
B*46:01	6, 322	6, 332	YPANSIVCRF	0. 82
B*46:01	6, 641	6, 651	GVVQQLPETY	0. 82

B*49:01	862	872	VELGTEVNEF	0.82
B*49:01	1,200	1,210	SEKQVEQKIA	0.82
B*49:01	2,035	2,045	SEDAQGMDNL	0.82
B*49:01	3,555	3,565	LEDEFTPFDV	0.82
B*51:01	3,614	3,624	NAFLPFAMGI	0.82
B*57:01	816	826	KGGAPTKVTF	0.82
B*57:01	1,988	1,998	AKLLHKPIVW	0.82
B*57:01	3,953	3,963	FSSLPSYAAF	0.82
B*58:01	608	618	LTSQWLTNIF	0.82
B*58:01	3,000	3,010	GVCVSTSGRW	0.82
B*58:01	3,125	3,135	VSFLAHIQWM	0.82
B*58:01	3,468	3,478	ITVNVLAWLY	0.82
B*58:01	4,656	4,666	TDLTKPYIKW	0.82
B*58:01	4,959	4,969	ICSTMTNRQF	0.82
B*58:01	5,566	5,576	APTLVPQEHY	0.82
B*58:01	6,158	6,168	HHSIGFDYVY	0.82
C*03:02	3,950	3,960	ASEFSSLPSY	0.82
C*03:04	306	316	VASPNECNQM	0.82
C*05:01	1,010	1,020	IVEVQPQLEM	0.82
C*05:01	1,542	1,552	YTSNPTTFHL	0.82
C*06:02	5,830	5,840	TRNPAWRKAV	0.82
C*06:02	6,591	6,601	FRNARNGVLI	0.82
C*07:02	6,298	6,308	YSYATHSDKF	0.82
C*12:02	5,994	6,004	YQVNGYPNMF	0.82
C*14:02	3,688	3,698	VMYASAVVLL	0.82
C*14:02	4,530	4,540	HFDEGNCDTL	0.82
C*17:01	2,960	2,970	VLMDGSIIQF	0.82
C*17:03	2,960	2,970	VLMDGSIIQF	0.82
A*01:01	6,491	6,501	KVDGVDVELF	0.83
A*02:02	2,616	2,626	TLVATAEAEL	0.83
A*02:02	3,373	3,383	RIQPGQTFSV	0.83
A*02:05	967	977	AALQPENPHL	0.83
A*02:05	984	994	WLDDDSQQTV	0.83
A*02:05	3,459	3,469	AQAAGTDTTI	0.83
A*02:05	6,765	6,775	SQDLSVVSKV	0.83
A*02:06	550	560	RTLETAQNSV	0.83
A*03:01	1,787	1,797	GVQIPCTCGK	0.83
A*11:01	5,475	5,485	KLSYGIATVR	0.83
A*23:01	5,994	6,004	YQVNGYPNMF	0.83
A*25:01	4,982	4,992	ATVVIGTSKF	0.83
A*25:01	5,994	6,004	YQVNGYPNMF	0.83
A*25:01	6,651	6,661	FTQSRNLQEF	0.83
A*26:01	3,222	3,232	FSGAMDTTSY	0.83

A*26:01	4,276	4,286	AFAVDAAKAY	0.83
A*29:02	896	906	DLDEWSMATY	0.83
A*29:02	4,345	4,355	KGFCDLKGKY	0.83
A*31:01	5,538	5,548	VVYRGTTTYK	0.83
A*32:01	3,596	3,606	LLVLVQSTQW	0.83
A*33:01	2,416	2,426	MCYKRNRATR	0.83
A*68:02	534	544	YAFASEAARV	0.83
B*35:03	5,975	5,985	IPKDMTYRRL	0.83
B*38:01	6,789	6,799	WCKDGHVETF	0.83
B*38:01	7,033	7,043	FWRNTNPIQL	0.83
B*39:01	5,026	5,036	MLRIMASLVL	0.83
B*40:02	1,829	1,839	ASEYTGNYQC	0.83
B*44:02	672	682	KESVQTFFKL	0.83
B*44:03	2,620	2,630	TAEAELAKNV	0.83
B*45:01	1,912	1,922	FTEQPIDLVP	0.83
B*49:01	1,297	1,307	QEGVLTAVVI	0.83
B*49:01	6,684	6,694	LEGYAFEHIV	0.83
B*51:01	75	85	DARTAPHGHV	0.83
B*51:01	5,948	5,958	LHPTQAPTHL	0.83
B*51:01	6,395	6,405	YVPLKSATCI	0.83
B*53:01	4,092	4,102	TTFTYASALW	0.83
B*53:01	6,627	6,637	EAVKTQFNYY	0.83
B*57:01	5,566	5,576	APTLVPQEHY	0.83
B*58:01	3,646	3,656	LPSLATVAYF	0.83
B*58:01	5,677	5,687	VNSTLEQYVF	0.83
C*01:02	3,659	3,669	YMPASWVMRI	0.83
C*02:02	3,950	3,960	ASEFSSLPSY	0.83
C*03:04	967	977	AALQPENPHL	0.83
C*05:01	6,765	6,775	SQDLSVVSKV	0.83
C*07:04	3,253	3,263	VLYQPPQTSI	0.83
C*12:02	5,390	5,400	TQLYLGGMSY	0.83
C*12:03	5,619	5,629	HFAIGLALYY	0.83
C*14:02	798	808	KYCALAPNMM	0.83
C*14:02	2,893	2,903	VFSAVGNICY	0.83
C*17:01	6,664	6,674	SQMEIDFLEL	0.83
C*17:03	6,664	6,674	SQMEIDFLEL	0.83
A*01:01	2,670	2,680	VTGDSCNNYM	0.84
A*01:01	4,626	4,636	SGVPVVDSYY	0.84
A*02:02	5,555	5,565	VLTSHTVMPL	0.84
A*02:02	6,637	6,647	KKVDGVVQQL	0.84
A*02:05	5,676	5,686	KVNSTLEQYV	0.84
A*02:06	967	977	AALQPENPHL	0.84
A*03:01	2,055	2,065	EVVENPTIQK	0.84

A*03:01	5, 259	5, 269	LAIDAYPLTK	0. 84
A*03:01	5, 460	5, 470	KLFAAETLKA	0. 84
A*11:01	1, 851	1, 861	YCIDGALLTK	0. 84
A*24:02	1, 660	1, 670	KWKYPQVNGL	0. 84
A*24:02	3, 194	3, 204	MYLKLRSDVL	0. 84
A*25:01	2, 317	2, 327	SSFKWDLTAF	0. 84
A*25:01	4, 423	4, 433	TSTDVVYRAF	0. 84
A*25:01	4, 916	4, 926	MSYEDQDALF	0. 84
A*25:01	6, 441	6, 451	YKQFDTYNLW	0. 84
A*29:02	1, 027	1, 037	TIEVNSFSGY	0. 84
A*31:01	68	78	YVFIKRSDAR	0. 84
A*31:01	1, 314	1, 324	TTEMLAKALR	0. 84
A*31:01	3, 984	3, 994	KLKKSLNVAK	0. 84
A*31:01	4, 585	4, 595	KTVQFCDAMR	0. 84
A*32:01	3, 373	3, 383	RIQPGQTFSV	0. 84
A*32:01	3, 553	3, 563	ALLEDEFTPF	0. 84
A*32:01	6, 879	6, 889	KGVAPGTAVL	0. 84
A*68:02	3, 492	3, 502	TTTLNDFNLV	0. 84
A*68:02	5, 318	5, 328	YEAMYTPHTV	0. 84
A*68:02	6, 861	6, 871	TLAVPYNMRV	0. 84
B*13:02	3, 286	3, 296	VQVTCGTTTL	0. 84
B*14:02	1, 617	1, 627	SHEGKTFYVL	0. 84
B*15:03	76	86	ARTAPHGHVM	0. 84
B*35:01	2, 324	2, 334	TAFGLVAEWF	0. 84
B*39:01	95	105	IQYGRSGETL	0. 84
B*39:01	6, 302	6, 312	THSDKFTDGV	0. 84
B*40:01	1, 817	1, 827	AQYELKHGTF	0. 84
B*40:01	4, 649	4, 659	TAESHVDTDL	0. 84
B*40:02	639	649	VEFLRDGWEI	0. 84
B*40:02	5, 272	5, 282	QEYADVFHLY	0. 84
B*45:01	1, 503	1, 513	EEHFIETISL	0. 84
B*46:01	1, 817	1, 827	AQYELKHGTF	0. 84
B*46:01	2, 736	2, 746	SAAKKNNLPF	0. 84
B*46:01	4, 277	4, 287	FAVDAAKAYK	0. 84
B*46:01	6, 536	6, 546	IAANTVIWDY	0. 84
B*49:01	672	682	KESVQTFFKL	0. 84
B*53:01	2, 587	2, 597	DSAEVAVKMF	0. 84
B*53:01	3, 389	3, 399	SPSGVYQCAM	0. 84
B*57:01	4, 265	4, 275	VPANSTVLSF	0. 84
B*58:01	1, 956	1, 966	ASRELKVTFF	0. 84
C*01:02	6, 497	6, 507	VELFENKTTL	0. 84
C*02:02	3, 076	3, 086	FGEYSHVVAF	0. 84
C*02:02	5, 819	5, 829	RPQIGVVREF	0. 84

C*03:02	2, 317	2, 327	SSFKWDLTAF	0. 84
C*03:04	4, 519	4, 529	YTMADLVYAL	0. 84
C*03:04	4, 795	4, 805	VAALTNNVAF	0. 84
C*03:04	5, 226	5, 236	LPYPDPSRIL	0. 84
C*05:01	587	597	TSDLATNNLV	0. 84
C*07:02	1, 533	1, 543	LKRGDKSVYY	0. 84
C*07:02	6, 376	6, 386	YYSDSPCESH	0. 84
C*07:04	5, 403	5, 413	SHKPPISFPL	0. 84
C*12:03	3, 246	3, 256	FSNSGSDVLY	0. 84
C*14:02	2, 219	2, 229	SFNYLKSPNF	0. 84
C*15:02	6, 836	6, 846	SATLPKGIMM	0. 84
C*16:01	306	316	VASPNECNQM	0. 84
C*17:01	1, 549	1, 559	FHLDGEVITF	0. 84
C*17:03	1, 549	1, 559	FHLDGEVITF	0. 84
A*01:01	2, 941	2, 951	TNVLEGSVAY	0. 85
A*01:01	5, 390	5, 400	TQLYLGGMSY	0. 85
A*02:02	599	609	AYITGGVVQL	0. 85
A*02:02	3, 688	3, 698	VMYASAVVLL	0. 85
A*02:06	3, 253	3, 263	VLYQPPQTSI	0. 85
A*02:06	5, 287	5, 297	KLHDELTGHM	0. 85
A*03:01	1, 252	1, 262	KFLTENLLLY	0. 85
A*11:01	283	293	TIQPRVEKKK	0. 85
A*11:01	541	551	ARVVRSIFSR	0. 85
A*11:01	5, 844	5, 854	YNSQNAVASK	0. 85
A*23:01	1, 660	1, 670	KWKYPQVNGL	0. 85
A*23:01	5, 749	5, 759	EYFNSVCRLM	0. 85
A*25:01	4, 118	4, 128	EISMDNSPNL	0. 85
A*32:01	1, 758	1, 768	KTCGQQQTTL	0. 85
A*32:01	2, 539	2, 549	GSLPINVIVF	0. 85
A*32:01	3, 950	3, 960	ASEFSSLPSY	0. 85
A*33:01	2, 837	2, 847	ADFDTWFSQR	0. 85
A*68:02	375	385	NSEVGPEHSL	0. 85
B*07:02	4, 066	4, 076	IIPLTTAAKL	0. 85
B*07:02	7, 052	7, 062	KFPLKLRGTA	0. 85
B*15:01	4, 625	4, 635	GSGVPVVDSY	0. 85
B*15:03	6, 642	6, 652	VVQQLPETYF	0. 85
B*18:01	5, 140	5, 150	NEFYAYLRKH	0. 85
B*27:05	6, 889	6, 899	RQWLPTGTLL	0. 85
B*35:01	6, 145	6, 155	TCFSTASDTY	0. 85
B*44:02	2, 865	2, 875	REVGFVVPGL	0. 85
B*44:03	672	682	KESVQTFFKL	0. 85
B*44:03	951	961	TEDDYQGKPL	0. 85
B*44:03	3, 200	3, 210	SDVLLPLTQY	0. 85

B*44:03	3,993	4,003	KSEFDRDAAM	0.85
B*45:01	1,089	1,099	QVESDDYIAT	0.85
B*49:01	2,067	2,077	LECNVKTTEV	0.85
B*51:01	4,098	4,108	SALWEIQQVV	0.85
B*57:01	2,178	2,188	FTLLLQLCTF	0.85
B*58:01	4,986	4,996	IGTSKFYGGW	0.85
C*03:02	1,810	1,820	VMMSAPPAQY	0.85
C*05:01	375	385	NSEVGPEHSL	0.85
C*05:01	1,642	1,652	TTDPSFLGRY	0.85
C*05:01	4,167	4,177	ACTDDNALAY	0.85
C*07:04	242	252	TERSEKSYEL	0.85
C*07:04	4,477	4,487	YQHEETIYNL	0.85
C*12:02	6,651	6,661	FTQSRNLQEF	0.85
C*14:02	1,187	1,197	LYDKLVSSFL	0.85
C*14:02	2,317	2,327	SSFKWDLTAF	0.85
C*14:02	3,393	3,403	VYQCAMRPNF	0.85
C*15:02	4,562	4,572	FVENPDILRV	0.85
C*15:02	6,637	6,647	KKVDGVVQQL	0.85
C*16:01	165	175	HSSGVTRELM	0.85
C*17:01	1,430	1,440	YTSKTTVASL	0.85
C*17:01	2,869	2,879	FVVPGLPGTI	0.85
C*17:01	6,879	6,889	KGVAPGTAVL	0.85
C*17:03	1,430	1,440	YTSKTTVASL	0.85
C*17:03	2,869	2,879	FVVPGLPGTI	0.85
C*17:03	6,879	6,889	KGVAPGTAVL	0.85
A*01:01	1,281	1,291	ITFLKKDAPY	0.86
A*01:01	2,982	2,992	TTFDSEYCRH	0.86
A*01:01	5,619	5,629	HFAIGLALYY	0.86
A*02:02	3,187	3,197	TFLLNKEMYL	0.86
A*11:01	1,553	1,563	GEVITFDNLK	0.86
A*23:01	798	808	KYCALAPNMM	0.86
A*24:02	5,993	6,003	NYQVNGYPNM	0.86
A*29:02	1,832	1,842	YTGNYQCGHY	0.86
A*29:02	2,241	2,251	LSVCLGSLIY	0.86
A*29:02	5,216	5,226	LVKQGDDYVY	0.86
A*29:02	5,796	5,806	KSAQCFKMFY	0.86
A*32:01	3,151	3,161	ICISTKHFYW	0.86
A*33:01	235	245	EHEIAWYTER	0.86
A*68:02	1,570	1,580	VRTIKVFTTV	0.86
A*68:02	2,707	2,717	QVAKSHNIAL	0.86
A*68:02	2,754	2,764	QVVNVVTTKI	0.86
A*68:02	3,714	3,724	WTLMNVLTLV	0.86
A*68:02	3,838	3,848	NSIDAFKLNI	0.86

B*18:01	525	535	EQKSILSPLY	0.86
B*18:01	960	970	LEFGATSAAL	0.86
B*18:01	1,728	1,738	GELGDVRETM	0.86
B*18:01	3,313	3,323	TSEDMLNPNY	0.86
B*27:05	2,339	2,349	FTRFFYVLGL	0.86
B*35:01	4,223	4,233	DTPKGPKVKY	0.86
B*35:01	4,628	4,638	VPVVDSYYSL	0.86
B*35:03	4,718	4,728	FPLTSFGPLV	0.86
B*38:01	4,593	4,603	MRNAGIVGVL	0.86
B*39:01	2,952	2,962	SLRPDTRYVL	0.86
B*40:01	2,272	2,282	REGYLNSTNV	0.86
B*40:01	2,623	2,633	AELAKNVSLD	0.86
B*40:01	3,950	3,960	ASEFSSLPSY	0.86
B*40:02	1,145	1,155	QHEVLLAPLL	0.86
B*40:02	2,623	2,633	AELAKNVSLD	0.86
B*44:02	897	907	LDEWSMATYY	0.86
B*46:01	1,766	1,776	TLKGVEAVMY	0.86
B*49:01	911	921	SGEFKLASHM	0.86
B*49:01	3,556	3,566	EDEFTPFDVV	0.86
B*53:01	18	28	LPVLQVRDVL	0.86
B*53:01	747	757	LPTEVLTEEV	0.86
B*53:01	6,505	6,515	TLPVNVAFEL	0.86
B*57:01	2,975	2,985	EGSVRVVTTF	0.86
B*57:01	5,021	5,031	RAMPNMLRIM	0.86
B*58:01	2,560	2,570	KSASVYYSQL	0.86
B*58:01	4,982	4,992	ATVVIGTSKF	0.86
B*58:01	5,003	5,013	YSDVENPHLM	0.86
C*01:02	1,342	1,352	YTVEEAKTVL	0.86
C*01:02	5,569	5,579	LVPQEHYVRI	0.86
C*02:02	3,910	3,920	TEAFEKMVSL	0.86
C*03:04	1,556	1,566	ITFDNLKTLL	0.86
C*03:04	3,521	3,531	LSAQTGIAVL	0.86
C*03:04	5,318	5,328	YEAMYTPHTV	0.86
C*04:01	2,319	2,329	FKWDLTAFGL	0.86
C*04:43	2,319	2,329	FKWDLTAFGL	0.86
C*08:02	843	853	FELDERIDKV	0.86
C*12:02	1,817	1,827	AQYELKHGTF	0.86
C*12:02	4,277	4,287	FAVDAAKAYK	0.86
C*12:03	6,298	6,308	YSYATHSDKF	0.86
C*14:02	7	17	GFNEKTHVQL	0.86
C*14:02	3,179	3,189	TFEEAALCTF	0.86
C*17:01	1,095	1,105	YIATNGPLKV	0.86
C*17:03	1,095	1,105	YIATNGPLKV	0.86

A*01:01	5, 107	5, 117	TDGNKIADKY	0. 87
A*01:01	6, 390	6, 400	VSDIDYVPLK	0. 87
A*02:01	5, 050	5, 060	YRLANECAQV	0. 87
A*02:02	6, 078	6, 088	HLIPLMYKGL	0. 87
A*02:05	95	105	IQYGRSGETL	0. 87
A*02:05	2, 881	2, 891	TTNGDFLHFL	0. 87
A*02:05	4, 915	4, 925	SMSYEDQDAL	0. 87
A*02:06	4, 094	4, 104	FTYASALWEI	0. 87
A*02:06	6, 105	6, 115	KNLSDRVVFV	0. 87
A*03:01	2, 197	2, 207	ASMPTTIAKN	0. 87
A*03:01	2, 826	2, 836	ASTDTCFANK	0. 87
A*11:01	2, 737	2, 747	AAKKNNLPFK	0. 87
A*11:01	4, 362	4, 372	ANDPVGFTLK	0. 87
A*11:01	6, 954	6, 964	TYICGFIQQK	0. 87
A*25:01	5, 644	5, 654	DALCEKALKY	0. 87
A*25:01	5, 978	5, 988	DMTYRRLISM	0. 87
A*25:01	6, 322	6, 332	YPANSIVCRF	0. 87
A*26:01	708	718	TFVTHSKGLY	0. 87
A*26:01	905	915	YYLFDESGEF	0. 87
A*26:01	2, 324	2, 334	TAFGLVAEWF	0. 87
A*29:02	4, 553	4, 563	YFNKKDWYDF	0. 87
A*33:01	4, 667	4, 677	LLKYDFTEER	0. 87
A*68:01	253	263	TPFEIKLAKK	0. 87
A*68:01	1, 949	1, 959	LTGYKKPASR	0. 87
A*68:02	1, 479	1, 489	TVSVSSPDAV	0. 87
A*68:02	4, 179	4, 189	TTKGGRFVLA	0. 87
B*08:01	6, 659	6, 669	EFKPRSQMEI	0. 87
B*13:02	2, 594	2, 604	KMFDAYVNTF	0. 87
B*14:02	4, 593	4, 603	MRNAGIVGVL	0. 87
B*15:01	6, 271	6, 281	VPQADVEWKF	0. 87
B*15:01	6, 777	6, 787	VTIDYTEISF	0. 87
B*15:03	4, 475	4, 485	SNYQHEETIY	0. 87
B*27:05	6, 010	6, 020	IRHVRAWIGF	0. 87
B*35:01	884	894	QPVSELLTPL	0. 87
B*35:01	940	950	EFEPSTQYEY	0. 87
B*35:01	5, 303	5, 313	MLTNDNTSRY	0. 87
B*35:03	6, 814	6, 824	MPNLYKMQRM	0. 87
B*38:01	3, 883	3, 893	QLRVESSSKL	0. 87
B*39:01	1, 071	1, 081	KHGGGVAGAL	0. 87
B*40:01	442	452	GSEGLNDNLL	0. 87
B*40:01	2, 057	2, 067	VENPTIQKDV	0. 87
B*40:01	5, 732	5, 742	AQLPAPRTLL	0. 87
B*44:02	656	666	ACEIVGGQIV	0. 87

B*44:02	754	764	EEVVLKTGDL	0.87
B*44:02	946	956	QYEYGTEDDY	0.87
B*44:02	1,781	1,791	YEQFKKGVQI	0.87
B*44:02	3,200	3,210	SDVLLPLTQY	0.87
B*45:01	5,774	5,784	PAEIVDTVSA	0.87
B*45:01	7,086	7,096	RENNRVVISS	0.87
B*46:01	3,756	3,766	VMFLARGIVF	0.87
B*49:01	6,889	6,899	RQWLPTGTLL	0.87
B*49:01	6,944	6,954	KENDSKEGFF	0.87
B*53:01	2,572	2,582	QPILLLDQAL	0.87
B*53:01	3,705	3,715	VYDDGARRVW	0.87
B*57:01	1,758	1,768	KTCGQQQTTL	0.87
B*57:01	3,600	3,610	VQSTQWSLFF	0.87
B*58:01	722	732	KSREETGLLM	0.87
B*58:01	3,600	3,610	VQSTQWSLFF	0.87
C*02:02	5,077	5,087	TSSGDATTAY	0.87
C*03:04	3,334	3,344	FLVQAGNVQL	0.87
C*07:02	5,910	5,920	LYDKLQFTSL	0.87
C*12:02	1,586	1,596	TQVVDMSMTY	0.87
C*14:02	708	718	TFVTHSKGLY	0.87
C*14:02	2,103	2,113	AYVDNSSLTI	0.87
C*14:02	2,972	2,982	TYLEGSVRVV	0.87
C*15:02	5,365	5,375	STSHKLVLSV	0.87
A*01:01	3,203	3,213	LLPLTQYNRY	0.88
A*01:01	6,158	6,168	HHSIGFDYVY	0.88
A*02:02	52	62	GLVEVEKGVL	0.88
A*02:02	7,064	7,074	SLKEGQINDM	0.88
A*02:05	1,239	1,249	ACVEEVTTTL	0.88
A*02:05	6,805	6,815	SQAWQPGVAM	0.88
A*02:06	2,126	2,136	TLATHGLAAV	0.88
A*03:01	63	73	QLEQPYVFIK	0.88
A*03:01	4,939	4,949	MNLKYAISAK	0.88
A*03:01	4,981	4,991	GATVVIGTSK	0.88
A*11:01	2,551	2,561	KSKCEESSAK	0.88
A*11:01	3,347	3,357	GHSMQNCVLK	0.88
A*11:01	3,828	3,838	MNSQGLLPPK	0.88
A*11:01	6,810	6,820	PGVAMPNLYK	0.88
A*25:01	3,140	3,150	LVPFWITIAY	0.88
A*25:01	5,741	5,751	LTKGTLEPEY	0.88
A*25:01	6,444	6,454	FDTYNLWNTF	0.88
A*29:02	1,695	1,705	FNPPALQDAY	0.88
A*31:01	2,949	2,959	AYESLRPDTR	0.88
A*32:01	5,560	5,570	TVMPLSAPTL	0.88

A*32:01	6,571	6,581	TICAPLTVFF	0.88
A*33:01	3,538	3,548	ELLQNGMNGR	0.88
A*33:01	4,421	4,431	TGTSTDVVYR	0.88
A*33:01	5,652	5,662	KYLPIDKCSR	0.88
A*33:01	6,860	6,870	LTLAVPYNMR	0.88
A*68:01	4,957	4,967	VSICSTMTNR	0.88
A*68:02	482	492	ASTSAFVETV	0.88
A*68:02	1,294	1,304	DVVQEGVLTA	0.88
A*68:02	5,096	5,106	AVTANVNALL	0.88
A*68:02	5,848	5,858	NAVASKILGL	0.88
B*13:02	1,595	1,605	YGQQFGPTYL	0.88
B*13:02	3,831	3,841	QGLLPPKNSI	0.88
B*14:02	3,911	3,921	EAFEKMVSLL	0.88
B*15:01	1,079	1,089	ALNKATNNAM	0.88
B*15:01	5,972	5,982	IPGIPKDMTY	0.88
B*15:01	6,673	6,683	LAMDEFIERY	0.88
B*18:01	1,586	1,596	TQVVDMSMTY	0.88
B*18:01	3,496	3,506	NDFNLVAMKY	0.88
B*27:05	6,661	6,671	KPRSQMEIDF	0.88
B*35:01	1,274	1,284	TLVSDIDITF	0.88
B*35:01	6,506	6,516	LPVNVAFELW	0.88
B*35:03	5,692	5,702	LPETTADIVV	0.88
B*37:01	862	872	VELGTEVNEF	0.88
B*37:01	5,318	5,328	YEAMYTPHTV	0.88
B*44:02	5,061	5,071	SEMVMCGGSL	0.88
B*44:03	3,077	3,087	GEYSHVVAFN	0.88
B*44:03	5,471	5,481	EETFKLSYGI	0.88
B*45:01	1,895	1,905	TEIDPKLDNY	0.88
B*46:01	1,274	1,284	TLVSDIDITF	0.88
B*46:01	3,818	3,828	YLVSTQEFRY	0.88
B*49:01	375	385	NSEVGPEHSL	0.88
B*51:01	5,690	5,700	NALPETTADI	0.88
B*53:01	4,718	4,728	FPLTSFGPLV	0.88
B*57:01	672	682	KESVQTFFKL	0.88
B*57:01	6,534	6,544	VDIAANTVIW	0.88
B*58:01	2,131	2,141	GLAAVNSVPW	0.88
B*58:01	2,833	2,843	ANKHADFDTW	0.88
B*58:01	6,699	6,709	HSQLGGLHLL	0.88
C*02:02	5,881	5,891	TAHSCNVNRF	0.88
C*03:02	6,289	6,299	KAYKIEELFY	0.88
C*03:04	2,091	2,101	ITEEVGHTDL	0.88
C*03:04	5,021	5,031	RAMPNMLRIM	0.88
C*07:01	5,403	5,413	SHKPPISFPL	0.88

C*07:02	1,541	1,551	YYTSNPTTFH	0.88
C*07:02	2,901	2,911	CYTPSKLIEY	0.88
C*07:02	5,618	5,628	SHFAIGLALY	0.88
C*14:02	3,194	3,204	MYLKLRSDVL	0.88
C*14:02	4,475	4,485	SNYQHEETIY	0.88
A*01:01	1,549	1,559	FHLDGEVITF	0.89
A*01:01	2,910	2,920	YTDFATSACV	0.89
A*01:01	4,189	4,199	LLSDLQDLKW	0.89
A*02:02	3,051	3,061	SIVAGGIVAI	0.89
A*02:02	6,447	6,457	YNLWNTFTRL	0.89
A*02:02	6,912	6,922	STLIGDCATV	0.89
A*02:05	1,812	1,822	MSAPPAQYEL	0.89
A*02:05	3,866	3,876	KMSDVKCTSV	0.89
A*02:06	881	891	KTLQPVSELL	0.89
A*02:06	2,469	2,479	FISDEVARDL	0.89
A*02:06	3,875	3,885	VVLLSVLQQL	0.89
A*02:06	4,368	4,378	FTLKNTVCTV	0.89
A*03:01	1,343	1,353	TVEEAKTVLK	0.89
A*03:01	2,474	2,484	VARDLSLQFK	0.89
A*11:01	4,065	4,075	NIIPLTTAAK	0.89
A*11:01	4,277	4,287	FAVDAAKAYK	0.89
A*23:01	4,989	4,999	SKFYGGWHNM	0.89
A*24:02	4,444	4,454	KFLKTNCCRF	0.89
A*25:01	6,750	6,760	SVIDLLLDDF	0.89
A*26:01	2,380	2,390	APISAMVRMY	0.89
A*26:01	4,167	4,177	ACTDDNALAY	0.89
A*29:02	3,360	3,370	DTANPKTPKY	0.89
A*29:02	4,468	4,478	VVKRHTFSNY	0.89
A*29:02	4,903	4,913	FNKWGKARLY	0.89
A*29:02	6,412	6,422	AVCRHHANEY	0.89
A*29:02	6,975	6,985	TEHSWNADLY	0.89
A*31:01	3,335	3,345	LVQAGNVQLR	0.89
A*31:01	6,764	6,774	KSQDLSVVSK	0.89
A*68:01	541	551	ARVVRSIFSR	0.89
A*68:01	4,537	4,547	DTLKEILVTY	0.89
B*07:02	4,950	4,960	RARTVAGVSI	0.89
B*08:01	6,091	6,101	VVRIKIVQML	0.89
B*13:02	881	891	KTLQPVSELL	0.89
B*13:02	4,044	4,054	KLDNDALNNI	0.89
B*14:02	5,573	5,583	EHYVRITGLY	0.89
B*15:01	1,060	1,070	TVVVNAANVY	0.89
B*15:01	2,387	2,397	RMYIFFASFY	0.89
B*35:01	567	577	ITILDGISQY	0.89

B*35:01	896	906	DLDEWSMATY	0.89
B*37:01	3,993	4,003	KSEFDRDAAM	0.89
B*38:01	2,360	2,370	AVHFISNSWL	0.89
B*39:01	633	643	EKFKEGVEFL	0.89
B*40:02	1,398	1,408	VETKAIVSTI	0.89
B*44:02	5,200	5,210	TDLTKGPHEF	0.89
B*44:03	1,199	1,209	KSEKQVEQKI	0.89
B*45:01	1,019	1,029	MELTPVVQTI	0.89
B*45:01	2,620	2,630	TAEAELAKNV	0.89
B*46:01	1,956	1,966	ASRELKVTFF	0.89
B*46:01	3,149	3,159	YIICISTKHF	0.89
B*49:01	886	896	VSELLTPLGI	0.89
B*53:01	2,173	2,183	YMPYFFTLLL	0.89
B*53:01	2,310	2,320	ETIQITISSF	0.89
B*53:01	3,399	3,409	RPNFTIKGSF	0.89
B*53:01	6,814	6,824	MPNLYKMQRM	0.89
B*57:01	1,542	1,552	YTSNPTTFHL	0.89
B*57:01	5,677	5,687	VNSTLEQYVF	0.89
B*57:01	5,741	5,751	LTKGTLEPEY	0.89
B*57:01	6,977	6,987	HSWNADLYKL	0.89
B*58:01	6,363	6,373	KSAFVNLKQL	0.89
C*02:02	1,772	1,782	AVMYMGTLSY	0.89
C*03:02	5,021	5,031	RAMPNMLRIM	0.89
C*03:04	4,838	4,848	FFAQDGNAAI	0.89
C*03:04	6,879	6,889	KGVAPGTAVL	0.89
C*06:02	6,582	6,592	GRVDGQVDLF	0.89
C*08:02	1,812	1,822	MSAPPAQYEL	0.89
C*12:02	1,615	1,625	HNSHEGKTFY	0.89
C*12:02	4,714	4,724	FSTVFPLTSF	0.89
C*14:02	6,330	6,340	RFDTRVLSNL	0.89
C*14:02	6,713	6,723	KRFKESPFEL	0.89
C*16:01	134	144	HSYGADLKSF	0.89
C*16:01	3,371	3,381	FVRIQPGQTF	0.89
C*17:01	1,160	1,170	GADPIHSLRV	0.89
C*17:01	1,880	1,890	TTIKPVTYKL	0.89
C*17:03	1,160	1,170	GADPIHSLRV	0.89
C*17:03	1,880	1,890	TTIKPVTYKL	0.89
A*01:01	1,507	1,517	IETISLAGSY	0.9
A*01:01	4,120	4,130	SMDNSPNLAW	0.9
A*02:02	1,016	1,026	QLEMELTPVV	0.9
A*02:02	2,043	2,053	NLACEDLKPV	0.9
A*02:02	2,952	2,962	SLRPDTRYVL	0.9
A*02:05	2,960	2,970	VLMDGSIIQF	0.9

A*02:05	4, 793	4, 803	FSVAALTNNV	0. 9
A*02:05	5, 322	5, 332	YTPHTVLQAV	0. 9
A*02:05	5, 699	5, 709	IVVFDEISMA	0. 9
A*02:05	6, 389	6, 399	VVSDIDYVPL	0. 9
A*02:05	6, 889	6, 899	RQWLPTGTLL	0. 9
A*02:06	2, 336	2, 346	YILFTRFFYV	0. 9
A*03:01	4, 965	4, 975	NRQFHQKLLK	0. 9
A*03:01	6, 537	6, 547	AANTVIWDYK	0. 9
A*11:01	1, 215	1, 225	EVKPFITESK	0. 9
A*11:01	2, 500	2, 510	TVKNGSIHLY	0. 9
A*11:01	5, 596	5, 606	ANYQKVGMQK	0. 9
A*23:01	4, 094	4, 104	FTYASALWEI	0. 9
A*24:02	1, 333	1, 343	TYPGQGLNGY	0. 9
A*24:02	2, 791	2, 801	IFYLIIPVHV	0. 9
A*25:01	6, 673	6, 683	LAMDEFIERY	0. 9
A*26:01	3, 134	3, 144	MVMFTPLVPF	0. 9
A*26:01	6, 158	6, 168	HHSIGFDYVY	0. 9
A*29:02	897	907	LDEWSMATYY	0. 9
A*32:01	468	478	KLNEEIAIIL	0. 9
A*32:01	2, 560	2, 570	KSASVYYSQL	0. 9
A*32:01	3, 197	3, 207	KLRSDVLLPL	0. 9
A*68:02	867	877	EVNEFACVVA	0. 9
B*07:02	2, 952	2, 962	SLRPDTRYVL	0. 9
B*07:02	3, 276	3, 286	FPSGKVEGCM	0. 9
B*08:01	3, 363	3, 373	NPKTPKYKFV	0. 9
B*13:02	3, 540	3, 550	LQNGMNGRTI	0. 9
B*15:01	490	500	TVKGLDYKAF	0. 9
B*15:01	1, 414	1, 424	IKIQEGVVDY	0. 9
B*15:01	4, 510	4, 520	HISRQRLTKY	0. 9
B*15:03	2, 960	2, 970	VLMDGSIIQF	0. 9
B*15:03	3, 286	3, 296	VQVTCGTTTL	0. 9
B*15:03	3, 993	4, 003	KSEFDRDAAM	0. 9
B*15:03	5, 401	5, 411	CKSHKPPISF	0. 9
B*15:03	6, 158	6, 168	HHSIGFDYVY	0. 9
B*15:03	6, 651	6, 661	FTQSRNLQEF	0. 9
B*15:03	6, 847	6, 857	VAKYTQLCQY	0. 9
B*18:01	1, 817	1, 827	AQYELKHGTF	0. 9
B*35:03	3, 204	3, 214	LPLTQYNRYL	0. 9
B*35:03	3, 508	3, 518	EPLTQDHVDI	0. 9
B*37:01	953	963	DDYQGKPLEF	0. 9
B*39:01	206	216	ARAGKASCTL	0. 9
B*40:01	938	948	EEEFEPSTQY	0. 9
B*44:02	287	297	RVEKKKLDGF	0. 9

B*44:02	4, 454	4, 464	QEKDEDDNLI	0. 9
B*44:03	656	666	ACEIVGGQIV	0. 9
B*44:03	953	963	DDYQGKPLEF	0. 9
B*44:03	3, 496	3, 506	NDFNLVAMKY	0. 9
B*44:03	6, 657	6, 667	LQEFKPRSQM	0. 9
B*45:01	1, 367	1, 377	NEKQEILGTV	0. 9
B*45:01	4, 323	4, 333	QESFGGASCC	0. 9
B*45:01	6, 458	6, 468	SLENVAFNVV	0. 9
B*46:01	1, 419	1, 429	GVVDYGARFY	0. 9
B*46:01	2, 473	2, 483	EVARDLSLQF	0. 9
B*46:01	6, 946	6, 956	NDSKEGFFTY	0. 9
B*51:01	2, 146	2, 156	YAKPFLNKVV	0. 9
B*51:01	3, 043	3, 053	IGALDISASI	0. 9
B*57:01	2, 557	2, 567	SSAKSASVYY	0. 9
B*57:01	2, 833	2, 843	ANKHADFDTW	0. 9
C*01:02	2, 080	2, 090	IILKPANNSL	0. 9
C*02:02	4, 626	4, 636	SGVPVVDSYY	0. 9
C*02:02	4, 916	4, 926	MSYEDQDALF	0. 9
C*03:02	6, 847	6, 857	VAKYTQLCQY	0. 9
C*03:04	4, 771	4, 781	YAADPAMHAA	0. 9
C*04:01	984	994	WLDDDSQQTV	0. 9
C*04:43	984	994	WLDDDSQQTV	0. 9
C*07:02	3, 748	3, 758	NYSGVVTTVM	0. 9
C*07:04	569	579	ILDGISQYSL	0. 9
C*17:01	1, 158	1, 168	IFGADPIHSL	0. 9
C*17:03	1, 158	1, 168	IFGADPIHSL	0. 9
A*01:01	6, 737	6, 747	ITDAQTGSSK	0. 91
A*02:01	3, 188	3, 198	FLLNKEMYLK	0. 91
A*02:01	4, 256	4, 266	RLQAGNATEV	0. 91
A*02:01	4, 718	4, 728	FPLTSFGPLV	0. 91
A*02:01	5, 063	5, 073	MVMCGGSLYV	0. 91
A*02:05	7, 046	7, 056	SLFDMSKFPL	0. 91
A*02:06	1, 556	1, 566	ITFDNLKTLL	0. 91
A*02:06	3, 704	3, 714	TVYDDGARRV	0. 91
A*11:01	4, 814	4, 824	KDFYDFAVSK	0. 91
A*23:01	3, 631	3, 641	MMFVKHKHAF	0. 91
A*23:01	6, 435	6, 445	GFSLWVYKQF	0. 91
A*24:02	3, 658	3, 668	VYMPASWVMR	0. 91
A*24:02	3, 796	3, 806	TCYFGLFCLL	0. 91
A*24:02	3, 825	3, 835	FRYMNSQGLL	0. 91
A*24:02	4, 678	4, 688	KLFDRYFKYW	0. 91
A*24:02	6, 168	6, 178	NPFMIDVQQW	0. 91
A*25:01	1, 008	1, 018	QTIVEVQPQL	0. 91

A*25:01	1, 325	1, 335	VPTDNYITTY	0. 91
A*25:01	4, 211	4, 221	YTELEPPCRF	0. 91
A*26:01	6, 291	6, 301	YKIEELFYSY	0. 91
A*32:01	1, 652	1, 662	MSALNHTKKW	0. 91
A*32:01	4, 008	4, 018	KMADQAMTQM	0. 91
A*33:01	2, 682	2, 692	YNKVENMTPR	0. 91
A*33:01	6, 954	6, 964	TYICGFIQQK	0. 91
A*68:01	4, 235	4, 245	FIKGLNNLNR	0. 91
A*68:02	5, 088	5, 098	NSVFNICQAV	0. 91
B*07:02	781	791	TPVCINGLML	0. 91
B*08:01	5, 910	5, 920	LYDKLQFTSL	0. 91
B*08:01	6, 090	6, 100	NVVRIKIVQM	0. 91
B*13:02	6, 073	6, 083	GDQFKHLIPL	0. 91
B*15:01	1, 706	1, 716	RARAGEAANF	0. 91
B*15:01	4, 477	4, 487	YQHEETIYNL	0. 91
B*27:05	4, 501	4, 511	FRIDGDMVPH	0. 91
B*39:01	2, 798	2, 808	VHVMSKHTDF	0. 91
B*39:01	3, 825	3, 835	FRYMNSQGLL	0. 91
B*39:01	3, 883	3, 893	QLRVESSSKL	0. 91
B*39:01	6, 699	6, 709	HSQLGGLHLL	0. 91
B*40:01	3, 181	3, 191	EEAALCTFLL	0. 91
B*44:02	953	963	DDYQGKPLEF	0. 91
B*44:02	5, 471	5, 481	EETFKLSYGI	0. 91
B*45:01	1, 636	1, 646	AFEYYHTTDP	0. 91
B*46:01	2, 222	2, 232	YLKSPNFSKL	0. 91
B*46:01	3, 494	3, 504	TLNDFNLVAM	0. 91
B*49:01	5, 572	5, 582	QEHYVRITGL	0. 91
B*51:01	152	162	DPYEDFQENW	0. 91
C*02:02	2, 137	2, 147	SVPWDTIANY	0. 91
C*02:02	2, 431	2, 441	IVNGVRRSFY	0. 91
C*03:02	2, 594	2, 604	KMFDAYVNTF	0. 91
C*03:04	5, 553	5, 563	YFVLTSHTVM	0. 91
C*03:04	6, 298	6, 308	YSYATHSDKF	0. 91
C*04:01	4, 848	4, 858	SDYDYYRYNL	0. 91
C*04:43	4, 848	4, 858	SDYDYYRYNL	0. 91
C*06:02	5, 273	5, 283	EYADVFHLYL	0. 91
C*07:02	397	407	LRKGGRTIAF	0. 91
C*07:02	2, 171	2, 181	TNYMPYFFTL	0. 91
C*08:02	824	834	TFGDDTVIEV	0. 91
C*08:02	4, 825	4, 835	FFKEGSSVEL	0. 91
C*08:02	5, 861	5, 871	TVDSSQGSEY	0. 91
C*08:02	6, 067	6, 077	SAKPPPGDQF	0. 91
C*15:02	3, 017	3, 027	RSLPGVFCGV	0. 91

C*15:02	5,725	5,735	YVYIGDPAQL	0.91
C*16:01	6,805	6,815	SQAWQPGVAM	0.91
C*17:01	967	977	AALQPENPHL	0.91
C*17:03	967	977	AALQPENPHL	0.91
A*01:01	6,674	6,684	AMDEFIERYK	0.92
A*02:01	1,549	1,559	FHLDGEVITF	0.92
A*02:02	1,338	1,348	GLNGYTVEEA	0.92
A*02:02	3,682	3,692	FKLKDCVMYA	0.92
A*02:02	6,258	6,268	VLHDIGNPKA	0.92
A*02:05	6,861	6,871	TLAVPYNMRV	0.92
A*02:05	7,020	7,030	EQIDGYVMHA	0.92
A*02:06	5,699	5,709	IVVFDEISMA	0.92
A*24:02	2,361	2,371	VHFISNSWLM	0.92
A*24:02	2,629	2,639	VSLDNVLSTF	0.92
A*24:02	2,885	2,895	DFLHFLPRVF	0.92
A*24:02	4,709	4,719	NFNVLFSTVF	0.92
A*24:02	4,859	4,869	TMCDIRQLLF	0.92
A*24:02	6,848	6,858	AKYTQLCQYL	0.92
A*26:01	2,901	2,911	CYTPSKLIEY	0.92
A*26:01	3,960	3,970	AAFATAQEAY	0.92
A*26:01	6,282	6,292	DAQPCSDKAY	0.92
A*32:01	1,880	1,890	TTIKPVTYKL	0.92
A*32:01	2,137	2,147	SVPWDTIANY	0.92
A*32:01	4,728	4,738	RKIFVDGVPF	0.92
A*32:01	5,181	5,191	SVLYYQNNVF	0.92
A*33:01	3,202	3,212	VLLPLTQYNR	0.92
A*68:02	4,246	4,256	MVLGSLAATV	0.92
A*68:02	5,704	5,714	EISMATNYDL	0.92
B*07:02	5,772	5,782	RCPAEIVDTV	0.92
B*07:02	7,056	7,066	KLRGTAVMSL	0.92
B*08:01	114	124	IPVAYRKVLL	0.92
B*08:01	6,330	6,340	RFDTRVLSNL	0.92
B*13:02	112	122	GEIPVAYRKV	0.92
B*13:02	1,144	1,154	NQHEVLLAPL	0.92
B*14:02	2,608	2,618	NVPMEKLKTL	0.92
B*15:01	5,985	5,995	ISMMGFKMNY	0.92
B*15:03	4,682	4,692	RYFKYWDQTY	0.92
B*15:03	5,368	5,378	HKLVLSVNPY	0.92
B*15:03	6,352	6,362	NKHAFHTPAF	0.92
B*27:05	5,835	5,845	WRKAVFISPY	0.92
B*35:01	6,054	6,064	YVDTPDNTDF	0.92
B*35:03	2,622	2,632	EAELAKNVSL	0.92
B*35:03	5,003	5,013	YSDVENPHLM	0.92

B*35:03	5,819	5,829	RPQIGVVREF	0.92
B*37:01	843	853	FELDERIDKV	0.92
B*37:01	3,076	3,086	FGEYSHVVAF	0.92
B*38:01	6,920	6,930	TVHTANKWDL	0.92
B*39:01	1,267	1,277	NLHPDSATLV	0.92
B*40:01	4,918	4,928	YEDQDALFAY	0.92
B*40:02	1,819	1,829	YELKHGTFTC	0.92
B*44:02	36	46	EEVLSEARQH	0.92
B*44:03	388	398	HNESGLKTIL	0.92
B*44:03	6,224	6,234	IEYPIIGDEL	0.92
B*46:01	1,615	1,625	HNSHEGKTFY	0.92
B*46:01	4,008	4,018	KMADQAMTQM	0.92
B*49:01	232	242	REHEHEIAWY	0.92
B*49:01	2,426	2,436	VECTTIVNGV	0.92
B*51:01	18	28	LPVLQVRDVL	0.92
B*51:01	6,799	6,809	YPKLQSSQAW	0.92
B*57:01	2,950	2,960	YESLRPDTRY	0.92
C*02:02	1,556	1,566	ITFDNLKTLL	0.92
C*03:02	1,615	1,625	HNSHEGKTFY	0.92
C*03:02	5,994	6,004	YQVNGYPNMF	0.92
C*07:01	6,218	6,228	KRVDWTIEYP	0.92
C*07:01	7,036	7,046	NTNPIQLSSY	0.92
C*07:04	1,639	1,649	YYHTTDPSFL	0.92
C*12:02	1,542	1,552	YTSNPTTFHL	0.92
C*12:02	6,432	6,442	ISAGFSLWVY	0.92
C*16:01	7,036	7,046	NTNPIQLSSY	0.92
C*17:01	1,603	1,613	YLDGADVTKI	0.92
C*17:03	1,603	1,613	YLDGADVTKI	0.92
A*01:01	1,159	1,169	FGADPIHSLR	0.93
A*01:01	3,645	3,655	LLPSLATVAY	0.93
A*02:01	1,291	1,301	IVGDVVQEGV	0.93
A*02:02	586	596	FTSDLATNNL	0.93
A*02:02	3,127	3,137	FLAHIQWMVM	0.93
A*02:02	4,143	4,153	KLQNNELSPV	0.93
A*02:02	5,546	5,556	YKLNVGDYFV	0.93
A*02:05	2,080	2,090	IILKPANNSL	0.93
A*02:06	546	556	SIFSRTLETA	0.93
A*02:06	2,230	2,240	KLINIIIWFL	0.93
A*02:06	2,971	2,981	NTYLEGSVRV	0.93
A*02:06	6,082	6,092	LMYKGLPWNV	0.93
A*03:01	591	601	ATNNLVVMAY	0.93
A*23:01	2,145	2,155	NYAKPFLNKV	0.93
A*23:01	2,776	2,786	KQLIKVTLVF	0.93

A*24:02	4, 851	4, 861	DYYRYNLPTM	0. 93
A*24:02	5, 749	5, 759	EYFNSVCRLM	0. 93
A*24:02	6, 441	6, 451	YKQFDTYNLW	0. 93
A*25:01	3, 944	3, 954	ATLQAIASEF	0. 93
A*25:01	4, 120	4, 130	SMDNSPNLAW	0. 93
A*29:02	1, 281	1, 291	ITFLKKDAPY	0. 93
A*29:02	4, 816	4, 826	FYDFAVSKGF	0. 93
A*33:01	34	44	SVEEVLSEAR	0. 93
A*33:01	4, 034	4, 044	MQIMLFTMLR	0. 93
A*68:02	1, 577	1, 587	TTVDNINLHT	0. 93
A*68:02	5, 055	5, 065	ECAQVLSEMV	0. 93
B*07:02	2, 009	2, 019	KPNTWCIRCL	0. 93
B*08:01	844	854	ELDERIDKVL	0. 93
B*08:01	5, 502	5, 512	RPPLNRNYVF	0. 93
B*15:01	3, 222	3, 232	FSGAMDTTSY	0. 93
B*15:01	4, 728	4, 738	RKIFVDGVPF	0. 93
B*15:03	134	144	HSYGADLKSF	0. 93
B*15:03	1, 369	1, 379	KQEILGTVSW	0. 93
B*27:05	1, 321	1, 331	ALRKVPTDNY	0. 93
B*39:01	6, 356	6, 366	FHTPAFDKSA	0. 93
B*40:01	1, 139	1, 149	AYENFNQHEV	0. 93
B*40:01	6, 759	6, 769	FVEIIKSQDL	0. 93
B*40:02	774	784	VEAPLVGTPV	0. 93
B*40:02	2, 518	2, 528	YERHSLSHFV	0. 93
B*44:03	1, 145	1, 155	QHEVLLAPLL	0. 93
B*44:03	5, 271	5, 281	NQEYADVFHL	0. 93
B*45:01	1, 618	1, 628	HEGKTFYVLP	0. 93
B*46:01	3, 348	3, 358	HSMQNCVLKF	0. 93
B*49:01	1, 691	1, 701	IELKFNPPAL	0. 93
B*51:01	3, 037	3, 047	TPLIQPIGAL	0. 93
B*51:01	3, 508	3, 518	EPLTQDHVDI	0. 93
B*53:01	975	985	HLEEEQEEDW	0. 93
B*53:01	3, 596	3, 606	LLVLVQSTQW	0. 93
B*57:01	2, 393	2, 403	ASFYYVWKSY	0. 93
C*02:02	2, 594	2, 604	KMFDAYVNTF	0. 93
C*03:02	5, 303	5, 313	MLTNDNTSRY	0. 93
C*03:02	6, 836	6, 846	SATLPKGIMM	0. 93
C*03:04	1, 384	1, 394	LAHAEETRKL	0. 93
C*05:01	5, 861	5, 871	TVDSSQGSEY	0. 93
C*07:01	1, 631	1, 641	TLRVEAFEYY	0. 93
C*07:02	3, 070	3, 080	MRFRRAFGEY	0. 93
C*07:02	5, 576	5, 586	VRITGLYPTL	0. 93
C*07:04	3, 704	3, 714	TVYDDGARRV	0. 93

C*07:04	4,530	4,540	HFDEGNCDTL	0.93
C*07:04	5,725	5,735	YVYIGDPAQL	0.93
C*08:02	6,694	6,704	YGDFSHSQLG	0.93
C*14:02	3,756	3,766	VMFLARGIVF	0.93
C*16:01	5,318	5,328	YEAMYTPHTV	0.93
C*17:01	4,930	4,940	RNVIPTITQM	0.93
C*17:03	4,930	4,940	RNVIPTITQM	0.93
A*01:01	722	732	KSREETGLLM	0.94
A*01:01	5,062	5,072	EMVMCGGSLY	0.94
A*02:01	3,890	3,900	SKLWAQCVQL	0.94
A*02:02	45	55	HLKDGTCGLV	0.94
A*02:02	1,295	1,305	VVQEGVLTAV	0.94
A*02:02	2,214	2,224	FCLEASFNYL	0.94
A*02:05	2,294	2,304	SVCLSGLDSL	0.94
A*11:01	2,757	2,767	NVVTTKIALK	0.94
A*23:01	2,249	2,259	IYSTAALGVL	0.94
A*23:01	3,096	3,106	VLCLTPVYSF	0.94
A*23:01	3,157	3,167	HFYWFFSNYL	0.94
A*25:01	2,605	2,615	STFNVPMEKL	0.94
A*29:02	6,772	6,782	SKVVKVTIDY	0.94
A*32:01	1,274	1,284	TLVSDIDITF	0.94
A*32:01	3,564	3,574	VVRQCSGVTF	0.94
A*32:01	5,111	5,121	KIADKYVRNL	0.94
A*32:01	6,963	6,973	KLALGGSVAI	0.94
A*68:01	282	292	KTIQPRVEKK	0.94
A*68:01	4,655	4,665	DTDLTKPYIK	0.94
A*68:01	5,997	6,007	NGYPNMFITR	0.94
A*68:02	6,905	6,915	DFVSDADSTL	0.94
B*07:02	3,204	3,214	LPLTQYNRYL	0.94
B*15:03	397	407	LRKGGRTIAF	0.94
B*18:01	6,007	6,017	EEAIRHVRAW	0.94
B*18:01	6,224	6,234	IEYPIIGDEL	0.94
B*27:05	4,057	4,067	ARDGCVPLNI	0.94
B*27:05	4,592	4,602	AMRNAGIVGV	0.94
B*27:05	4,929	4,939	KRNVIPTITQ	0.94
B*27:05	5,505	5,515	LNRNYVFTGY	0.94
B*35:01	1,896	1,906	EIDPKLDNYY	0.94
B*35:01	5,777	5,787	IVDTVSALVY	0.94
B*38:01	3,512	3,522	QDHVDILGPL	0.94
B*40:01	5,826	5,836	REFLTRNPAW	0.94
B*40:01	6,805	6,815	SQAWQPGVAM	0.94
B*40:02	1,367	1,377	NEKQEILGTV	0.94
B*44:03	525	535	EQKSILSPLY	0.94

B*46:01	3, 168	3, 178	RRVVFNGVSF	0. 94
B*46:01	3, 207	3, 217	TQYNRYLALY	0. 94
B*46:01	5, 626	5, 636	LYYPSARIVY	0. 94
B*46:01	6, 412	6, 422	AVCRHHANEY	0. 94
B*51:01	3, 204	3, 214	LPLTQYNRYL	0. 94
B*57:01	2, 303	2, 313	LDTYPSLETI	0. 94
B*57:01	6, 699	6, 709	HSQLGGLHLL	0. 94
C*08:01	375	385	NSEVGPEHSL	0. 94
C*08:01	1, 158	1, 168	IFGADPIHSL	0. 94
C*14:02	953	963	DDYQGKPLEF	0. 94
C*14:02	5, 993	6, 003	NYQVNGYPNM	0. 94
C*14:02	6, 427	6, 437	AYNMMISAGF	0. 94
C*15:02	3, 910	3, 920	TEAFEKMVSL	0. 94
C*15:02	5, 815	5, 825	SAINRPQIGV	0. 94
C*17:01	6, 836	6, 846	SATLPKGIMM	0. 94
C*17:03	6, 836	6, 846	SATLPKGIMM	0. 94
A*02:01	4, 008	4, 018	KMADQAMTQM	0. 95
A*02:01	6, 637	6, 647	KKVDGVVQQL	0. 95
A*02:02	2, 568	2, 578	QLMCQPILLL	0. 95
A*02:05	546	556	SIFSRTLETA	0. 95
A*02:05	599	609	AYITGGVVQL	0. 95
A*02:05	1, 283	1, 293	FLKKDAPYIV	0. 95
A*02:05	6, 474	6, 484	GQQGEVPVSI	0. 95
A*02:06	5, 676	5, 686	KVNSTLEQYV	0. 95
A*03:01	1, 277	1, 287	SDIDITFLKK	0. 95
A*03:01	3, 882	3, 892	QQLRVESSSK	0. 95
A*11:01	567	577	ITILDGISQY	0. 95
A*11:01	690	700	ADSIIIGGAK	0. 95
A*11:01	1, 650	1, 660	RYMSALNHTK	0. 95
A*11:01	5, 064	5, 074	VMCGGSLYVK	0. 95
A*23:01	3, 748	3, 758	NYSGVVTTVM	0. 95
A*23:01	4, 709	4, 719	NFNVLFSTVF	0. 95
A*23:01	4, 851	4, 861	DYYRYNLPTM	0. 95
A*23:01	6, 170	6, 180	FMIDVQQWGF	0. 95
A*23:01	6, 430	6, 440	MMISAGFSLW	0. 95
A*25:01	3, 869	3, 879	DVKCTSVVLL	0. 95
A*25:01	4, 930	4, 940	RNVIPTITQM	0. 95
A*26:01	707	717	ETFVTHSKGL	0. 95
A*26:01	1, 587	1, 597	QVVDMSMTYG	0. 95
A*26:01	4, 982	4, 992	ATVVIGTSKF	0. 95
A*26:01	5, 276	5, 286	DVFHLYLQYI	0. 95
A*29:02	3, 118	3, 128	TFYLTNDVSF	0. 95
A*31:01	5, 138	5, 148	FVNEFYAYLR	0. 95

A*32:01	458	468	KVNINIVGDF	0.95
A*33:01	3,447	3,457	GNFYGPFVDR	0.95
A*68:02	552	562	LETAQNSVRV	0.95
A*68:02	3,463	3,473	GTDTTITVNV	0.95
B*08:01	4,025	4,035	DKRAKVTSAM	0.95
B*08:01	6,814	6,824	MPNLYKMQRM	0.95
B*15:01	4,982	4,992	ATVVIGTSKF	0.95
B*15:01	5,469	5,479	ATEETFKLSY	0.95
B*15:01	5,718	5,728	ARLRAKHYVY	0.95
B*15:01	6,432	6,442	ISAGFSLWVY	0.95
B*27:05	5,310	5,320	SRYWEPEFYE	0.95
B*35:01	2,138	2,148	VPWDTIANYA	0.95
B*35:01	4,795	4,805	VAALTNNVAF	0.95
B*35:03	3,645	3,655	LLPSLATVAY	0.95
B*35:03	3,947	3,957	QAIASEFSSL	0.95
B*37:01	1,728	1,738	GELGDVRETM	0.95
B*44:02	1,145	1,155	QHEVLLAPLL	0.95
B*44:02	2,509	2,519	YFDKAGQKTY	0.95
B*44:02	3,179	3,189	TFEEAALCTF	0.95
B*44:03	376	386	SEVGPEHSLA	0.95
B*45:01	774	784	VEAPLVGTPV	0.95
B*45:01	5,469	5,479	ATEETFKLSY	0.95
B*46:01	2,376	2,386	LVQMAPISAM	0.95
B*46:01	4,537	4,547	DTLKEILVTY	0.95
B*46:01	5,985	5,995	ISMMGFKMNY	0.95
B*51:01	2,608	2,618	NVPMEKLKTL	0.95
B*53:01	3,360	3,370	DTANPKTPKY	0.95
C*01:02	4,107	4,117	VDADSKIVQL	0.95
C*02:02	5,741	5,751	LTKGTLEPEY	0.95
C*03:04	4,140	4,150	SAVKLQNNEL	0.95
C*03:04	6,054	6,064	YVDTPDNTDF	0.95
C*03:04	6,116	6,126	WAHGFELTSM	0.95
C*07:02	240	250	WYTERSEKSY	0.95
C*08:02	4,211	4,221	YTELEPPCRF	0.95
C*12:03	165	175	HSSGVTRELM	0.95
C*15:02	6,363	6,373	KSAFVNLKQL	0.95
C*17:01	6,416	6,426	HHANEYRLYL	0.95
C*17:01	6,699	6,709	HSQLGGLHLL	0.95
C*17:03	6,416	6,426	HHANEYRLYL	0.95
C*17:03	6,699	6,709	HSQLGGLHLL	0.95
A*01:01	2,805	2,815	TDFSSEIIGY	0.96
A*02:02	1,259	1,269	LLYIDINGNL	0.96
A*02:05	6,190	6,200	YCQVHGNAHV	0.96

A*02:06	1,990	2,000	LLHKPIVWHV	0.96
A*02:06	4,771	4,781	YAADPAMHAA	0.96
A*03:01	1,949	1,959	LTGYKKPASR	0.96
A*03:01	2,111	2,121	TIKKPNELSR	0.96
A*03:01	4,933	4,943	IPTITQMNLK	0.96
A*03:01	5,246	5,256	KTDGTLMIER	0.96
A*03:01	6,390	6,400	VSDIDYVPLK	0.96
A*11:01	4,421	4,431	TGTSTDVVYR	0.96
A*24:02	2,330	2,340	AEWFLAYILF	0.96
A*24:02	4,226	4,236	KGPKVKYLYF	0.96
A*26:01	6,651	6,661	FTQSRNLQEF	0.96
A*29:02	1,638	1,648	EYYHTTDPSF	0.96
A*29:02	3,058	3,068	VAIVVTCLAY	0.96
A*32:01	3,371	3,381	FVRIQPGQTF	0.96
A*33:01	4,192	4,202	DLQDLKWARF	0.96
A*68:01	2,500	2,510	TVKNGSIHLY	0.96
A*68:01	2,544	2,554	NVIVFDGKSK	0.96
A*68:01	3,359	3,369	VDTANPKTPK	0.96
A*68:01	6,433	6,443	SAGFSLWVYK	0.96
A*68:01	6,976	6,986	EHSWNADLYK	0.96
A*68:01	7,043	7,053	SSYSLFDMSK	0.96
A*68:02	2,303	2,313	LDTYPSLETI	0.96
A*68:02	4,126	4,136	NLAWPLIVTA	0.96
B*07:02	61	71	LPQLEQPYVF	0.96
B*07:02	6,879	6,889	KGVAPGTAVL	0.96
B*13:02	5,318	5,328	YEAMYTPHTV	0.96
B*14:02	3,825	3,835	FRYMNSQGLL	0.96
B*35:01	1,772	1,782	AVMYMGTLSY	0.96
B*35:01	1,965	1,975	FPDLNGDVVA	0.96
B*35:03	2,305	2,315	TYPSLETIQI	0.96
B*35:03	5,663	5,673	IPARARVECF	0.96
B*35:03	5,725	5,735	YVYIGDPAQL	0.96
B*37:01	334	344	GDFVKATCEF	0.96
B*37:01	1,019	1,029	MELTPVVQTI	0.96
B*40:01	1,770	1,780	VEAVMYMGTL	0.96
B*40:01	4,005	4,015	KLEKMADQAM	0.96
B*40:02	2,330	2,340	AEWFLAYILF	0.96
B*40:02	2,721	2,731	KDFMSLSEQL	0.96
B*44:02	1,010	1,020	IVEVQPQLEM	0.96
B*44:03	2,808	2,818	SSEIIGYKAI	0.96
B*46:01	3,624	3,634	IAMSAFAMMF	0.96
B*46:01	3,910	3,920	TEAFEKMVSL	0.96
B*46:01	4,032	4,042	SAMQIMLFTM	0.96

B*46:01	4,734	4,744	GVPFVVSTGY	0.96
B*49:01	1,010	1,020	IVEVQPQLEM	0.96
B*51:01	4,066	4,076	IIPLTTAAKL	0.96
B*53:01	5,975	5,985	IPKDMTYRRL	0.96
B*57:01	5,465	5,475	ETLKATEETF	0.96
B*58:01	165	175	HSSGVTRELM	0.96
B*58:01	700	710	LKALNLGETF	0.96
B*58:01	1,880	1,890	TTIKPVTYKL	0.96
B*58:01	6,977	6,987	HSWNADLYKL	0.96
C*02:02	4,477	4,487	YQHEETIYNL	0.96
C*02:02	5,390	5,400	TQLYLGGMSY	0.96
C*03:02	1,542	1,552	YTSNPTTFHL	0.96
C*03:02	5,861	5,871	TVDSSQGSEY	0.96
C*03:04	375	385	NSEVGPEHSL	0.96
C*03:04	586	596	FTSDLATNNL	0.96
C*05:01	4,562	4,572	FVENPDILRV	0.96
C*06:02	206	216	ARAGKASCTL	0.96
C*08:02	2,509	2,519	YFDKAGQKTY	0.96
C*08:02	2,670	2,680	VTGDSCNNYM	0.96
C*08:02	4,167	4,177	ACTDDNALAY	0.96
C*12:03	7,036	7,046	NTNPIQLSSY	0.96
C*14:02	3,157	3,167	HFYWFFSNYL	0.96
C*17:01	1,595	1,605	YGQQFGPTYL	0.96
C*17:01	6,054	6,064	YVDTPDNTDF	0.96
C*17:03	1,595	1,605	YGQQFGPTYL	0.96
C*17:03	6,054	6,064	YVDTPDNTDF	0.96
A*01:01	3,105	3,115	FLPGVYSVIY	0.97
A*01:01	3,186	3,196	CTFLLNKEMY	0.97
A*01:01	4,510	4,520	HISRQRLTKY	0.97
A*01:01	5,985	5,995	ISMMGFKMNY	0.97
A*01:01	6,412	6,422	AVCRHHANEY	0.97
A*02:05	5,775	5,785	AEIVDTVSAL	0.97
A*03:01	283	293	TIQPRVEKKK	0.97
A*24:02	5,994	6,004	YQVNGYPNMF	0.97
A*29:02	1,842	1,852	KHITSKETLY	0.97
A*29:02	2,960	2,970	VLMDGSIIQF	0.97
A*29:02	4,510	4,520	HISRQRLTKY	0.97
A*29:02	4,625	4,635	GSGVPVVDSY	0.97
A*31:01	2,144	2,154	ANYAKPFLNK	0.97
A*31:01	4,667	4,677	LLKYDFTEER	0.97
A*32:01	2,134	2,144	AVNSVPWDTI	0.97
A*32:01	5,732	5,742	AQLPAPRTLL	0.97
A*32:01	6,080	6,090	IPLMYKGLPW	0.97

A*68:01	2,493	2,503	SYIVDSVTVK	0.97
A*68:01	2,982	2,992	TTFDSEYCRH	0.97
A*68:02	1,002	1,012	NQTTTIQTIV	0.97
B*13:02	1,002	1,012	NQTTTIQTIV	0.97
B*15:01	527	537	KSILSPLYAF	0.97
B*15:01	938	948	EEEFEPSTQY	0.97
B*15:03	905	915	YYLFDESGEF	0.97
B*18:01	6,943	6,953	TKENDSKEGF	0.97
B*35:01	5,228	5,238	YPDPSRILGA	0.97
B*35:01	6,685	6,695	EGYAFEHIVY	0.97
B*38:01	79	89	APHGHVMVEL	0.97
B*38:01	5,576	5,586	VRITGLYPTL	0.97
B*40:01	6,664	6,674	SQMEIDFLEL	0.97
B*44:03	743	753	EGETLPTEVL	0.97
B*46:01	59	69	GVLPQLEQPY	0.97
B*46:01	1,706	1,716	RARAGEAANF	0.97
B*46:01	4,211	4,221	YTELEPPCRF	0.97
B*46:01	4,983	4,993	TVVIGTSKFY	0.97
B*53:01	1,878	1,888	YTTTIKPVTY	0.97
B*53:01	5,133	5,143	DVDTDFVNEF	0.97
B*57:01	22	32	QVRDVLVRGF	0.97
B*57:01	4,721	4,731	TSFGPLVRKI	0.97
B*58:01	1,988	1,998	AKLLHKPIVW	0.97
C*01:02	4,825	4,835	FFKEGSSVEL	0.97
C*02:02	88	98	LVAELEGIQY	0.97
C*03:02	5,553	5,563	YFVLTSHTVM	0.97
C*03:04	3,079	3,089	YSHVVAFNTL	0.97
C*05:01	1,812	1,822	MSAPPAQYEL	0.97
C*05:01	5,288	5,298	LHDELTGHML	0.97
C*06:02	1,540	1,550	VYYTSNPTTF	0.97
C*07:04	2,222	2,232	YLKSPNFSKL	0.97
C*12:03	485	495	SAFVETVKGL	0.97
C*14:02	3,807	3,817	RYFRLTLGVY	0.97
C*14:02	5,619	5,629	HFAIGLALYY	0.97
C*15:02	2,109	2,119	SLTIKKPNEL	0.97
C*15:02	2,560	2,570	KSASVYYSQL	0.97
C*16:01	567	577	ITILDGISQY	0.97
C*17:01	1,555	1,565	VITFDNLKTL	0.97
C*17:01	6,113	6,123	FVLWAHGFEL	0.97
C*17:03	1,555	1,565	VITFDNLKTL	0.97
C*17:03	6,113	6,123	FVLWAHGFEL	0.97
A*01:01	906	916	YLFDESGEFK	0.98
A*02:01	2,389	2,399	YIFFASFYYV	0.98

A*02:01	2,630	2,640	SLDNVLSTFI	0.98
A*02:01	5,645	5,655	ALCEKALKYL	0.98
A*02:02	5,691	5,701	ALPETTADIV	0.98
A*02:06	2,389	2,399	YIFFASFYYV	0.98
A*03:01	3,740	3,750	ALIISVTSNY	0.98
A*23:01	6,669	6,679	DFLELAMDEF	0.98
A*24:02	1,259	1,269	LLYIDINGNL	0.98
A*24:02	2,717	2,727	IWNVKDFMSL	0.98
A*24:02	3,631	3,641	MMFVKHKHAF	0.98
A*24:02	5,312	5,322	YWEPEFYEAM	0.98
A*25:01	938	948	EEEFEPSTQY	0.98
A*25:01	2,869	2,879	FVVPGLPGTI	0.98
A*26:01	5,994	6,004	YQVNGYPNMF	0.98
A*29:02	2,380	2,390	APISAMVRMY	0.98
A*29:02	2,389	2,399	YIFFASFYYV	0.98
A*29:02	2,637	2,647	TFISAARQGF	0.98
A*29:02	3,094	3,104	FTVLCLTPVY	0.98
A*31:01	4,398	4,408	SADAQSFLNR	0.98
A*31:01	4,676	4,686	RLKLFDRYFK	0.98
A*31:01	5,568	5,578	TLVPQEHYVR	0.98
A*32:01	6,642	6,652	VVQQLPETYF	0.98
A*33:01	1,463	1,473	NLEEAARYMR	0.98
A*33:01	2,524	2,534	SHFVNLDNLR	0.98
A*68:01	1,062	1,072	VVNAANVYLK	0.98
A*68:01	5,712	5,722	DLSVVNARLR	0.98
A*68:02	1,330	1,340	YITTYPGQGL	0.98
A*68:02	3,651	3,661	TVAYFNMVYM	0.98
B*13:02	5,271	5,281	NQEYADVFHL	0.98
B*14:02	12	22	THVQLSLPVL	0.98
B*15:01	3,987	3,997	KSLNVAKSEF	0.98
B*15:03	6,716	6,726	KESPFELEDF	0.98
B*35:01	134	144	HSYGADLKSF	0.98
B*35:01	2,572	2,582	QPILLLDQAL	0.98
B*35:01	5,225	5,235	YLPYPDPSRI	0.98
B*35:01	5,390	5,400	TQLYLGGMSY	0.98
B*35:01	6,067	6,077	SAKPPPGDQF	0.98
B*35:03	1,695	1,705	FNPPALQDAY	0.98
B*39:01	836	846	YKSVNITFEL	0.98
B*39:01	4,530	4,540	HFDEGNCDTL	0.98
B*40:02	1,817	1,827	AQYELKHGTF	0.98
B*45:01	866	876	TEVNEFACVV	0.98
B*45:01	4,263	4,273	TEVPANSTVL	0.98
B*46:01	1,010	1,020	IVEVQPQLEM	0.98

B*46:01	3,697	3,707	LILMTARTVY	0.98
B*46:01	5,154	5,164	ILSDDAVVCF	0.98
B*51:01	5,972	5,982	IPGIPKDMTY	0.98
B*58:01	2,178	2,188	FTLLLQLCTF	0.98
B*58:01	5,465	5,475	ETLKATEETF	0.98
C*03:02	905	915	YYLFDESGEF	0.98
C*03:02	5,777	5,787	IVDTVSALVY	0.98
C*03:04	6,416	6,426	HHANEYRLYL	0.98
C*04:01	905	915	YYLFDESGEF	0.98
C*04:43	905	915	YYLFDESGEF	0.98
C*05:01	6,067	6,077	SAKPPPGDQF	0.98
C*07:02	3,168	3,178	RRVVFNGVSF	0.98
C*07:04	824	834	TFGDDTVIEV	0.98
C*08:01	6,067	6,077	SAKPPPGDQF	0.98
C*08:02	722	732	KSREETGLLM	0.98
C*08:02	2,104	2,114	YVDNSSLTIK	0.98
C*12:02	1,772	1,782	AVMYMGTLSY	0.98
C*12:02	6,777	6,787	VTIDYTEISF	0.98
C*12:02	6,847	6,857	VAKYTQLCQY	0.98
C*14:02	1,455	1,465	LGYVTHGLNL	0.98
C*14:02	5,312	5,322	YWEPEFYEAM	0.98
C*17:01	3,253	3,263	VLYQPPQTSI	0.98
C*17:01	4,008	4,018	KMADQAMTQM	0.98
C*17:01	5,021	5,031	RAMPNMLRIM	0.98
C*17:03	3,253	3,263	VLYQPPQTSI	0.98
C*17:03	4,008	4,018	KMADQAMTQM	0.98
C*17:03	5,021	5,031	RAMPNMLRIM	0.98
A*01:01	1,402	1,412	AIVSTIQRKY	0.99
A*01:01	1,654	1,664	ALNHTKKWKY	0.99
A*02:01	5,546	5,556	YKLNVGDYFV	0.99
A*02:02	5,706	5,716	SMATNYDLSV	0.99
A*02:02	7,056	7,066	KLRGTAVMSL	0.99
A*02:05	1,158	1,168	IFGADPIHSL	0.99
A*02:06	83	93	HVMVELVAEL	0.99
A*03:01	1,321	1,331	ALRKVPTDNY	0.99
A*03:01	1,851	1,861	YCIDGALLTK	0.99
A*03:01	2,737	2,747	AAKKNNLPFK	0.99
A*11:01	2,137	2,147	SVPWDTIANY	0.99
A*24:02	2,335	2,345	AYILFTRFFY	0.99
A*24:02	3,807	3,817	RYFRLTLGVY	0.99
A*25:01	5,175	5,185	SIKNFKSVLY	0.99
A*25:01	5,243	5,253	DIVKTDGTLM	0.99
A*26:01	37	47	EVLSEARQHL	0.99

A*26:01	5,725	5,735	YVYIGDPAQL	0.99
A*29:02	4,934	4,944	PTITQMNLKY	0.99
A*29:02	5,902	5,912	LCIMSDRDLY	0.99
A*29:02	6,626	6,636	GEAVKTQFNY	0.99
A*31:01	4,235	4,245	FIKGLNNLNR	0.99
A*31:01	5,889	5,899	RFNVAITRAK	0.99
A*68:01	3,360	3,370	DTANPKTPKY	0.99
A*68:02	647	657	EIVKFISTCA	0.99
B*07:02	543	553	VVRSIFSRTL	0.99
B*07:02	5,582	5,592	YPTLNISDEF	0.99
B*08:01	3,486	3,496	WFLNRFTTTL	0.99
B*13:02	1,803	1,813	VQQESPFVMM	0.99
B*13:02	3,747	3,757	SNYSGVVTTV	0.99
B*15:01	127	137	GNKGAGGHSY	0.99
B*15:01	4,005	4,015	KLEKMADQAM	0.99
B*15:01	6,298	6,308	YSYATHSDKF	0.99
B*15:03	5,626	5,636	LYYPSARIVY	0.99
B*15:03	6,664	6,674	SQMEIDFLEL	0.99
B*35:03	3,973	3,983	VANGDSEVVL	0.99
B*35:03	5,560	5,570	TVMPLSAPTL	0.99
B*35:03	5,628	5,638	YPSARIVYTA	0.99
B*38:01	1,640	1,650	YHTTDPSFLG	0.99
B*38:01	4,494	4,504	AKHDFFKFRI	0.99
B*39:01	2,834	2,844	NKHADFDTWF	0.99
B*40:01	4,562	4,572	FVENPDILRV	0.99
B*40:02	1,709	1,719	AGEAANFCAL	0.99
B*40:02	2,908	2,918	IEYTDFATSA	0.99
B*44:02	6,028	6,038	REAVGTNLPL	0.99
B*44:03	1,781	1,791	YEQFKKGVQI	0.99
B*44:03	4,454	4,464	QEKDEDDNLI	0.99
B*45:01	2,035	2,045	SEDAQGMDNL	0.99
B*46:01	5,216	5,226	LVKQGDDYVY	0.99
B*49:01	6,657	6,667	LQEFKPRSQM	0.99
B*51:01	3,646	3,656	LPSLATVAYF	0.99
C*01:02	6,340	6,350	NLPGCDGGSL	0.99
C*02:02	4,762	4,772	RLSFKELLVY	0.99
C*03:02	2,324	2,334	TAFGLVAEWF	0.99
C*03:02	5,675	5,685	FKVNSTLEQY	0.99
C*03:04	6,615	6,625	KQASLNGVTL	0.99
C*05:01	2,509	2,519	YFDKAGQKTY	0.99
C*05:01	4,120	4,130	SMDNSPNLAW	0.99
C*17:01	3,371	3,381	FVRIQPGQTF	0.99
C*17:03	3,371	3,381	FVRIQPGQTF	0.99
